# Supplementary material for: Multiscale analysis reveals that diet-dependent midgut plasticity emerges from alterations in both stem cell niche coupling and enterocyte size
Source: eLife. 2021 Sep 23;10:e64125. doi: 10.7554/eLife.64125 (PMC8528489; doi:10.7554/eLife.64125)
Supplement: Supplementary file 1. [file elife-64125-supp1.docx]

Table1: list of diets

| Main diets | | | |
| --- | --- | --- | --- |
| **Ingredients** | Pre-experiment diet | High Sugar | High Yeast |
| Brewer’s yeast (g/L) | 50 | 11.85 | 105.9 |
| Sucrose (g/L) | 40 | 168.1 | 74.1 |
| Fly Agar (g/L) | 7 | 15 | 15 |
| Acid Mix (ml/L) | 12 | 10 | 10 |
| Cornmeal (g/L) | 60 |  |  |
| Moldex (ml/L) | 26.5 |  |  |

| Nutritional geometry. Isocaloric group 1 (69.65 calories per L) | | | | | |
| --- | --- | --- | --- | --- | --- |
| **Ingredients** | No yeast | Y:S = 1:14 | Y:S = 1:1.59 | Y:S = 1:0.7 | No sugar |
| Brewer’s yeast (g/L) | 0 | 1.185 | 6.96 | 10.59 | 18 |
| Sucrose (g/L) | 18 | 16.81 | 11.04 | 7.41 | 0 |
| Fly Agar (g/L) | 15 | 15 | 15 | 15 | 15 |
| Acid Mix (ml/L) | 10 | 10 | 10 | 10 | 10 |

| Nutritional geometry. Isocaloric group 2 (306.98 calories per L) | | | | | |
| --- | --- | --- | --- | --- | --- |
| **Ingredients** | No yeast | Y:S = 1:14 | Y:S = 1:1.59 | Y:S = 1:0.7 | No sugar |
| Brewer’s yeast (g/L) | 0 | 5.22 | 30.66 | 46.67 | 79.32 |
| Sucrose (g/L) | 79.32 | 74.1 | 48.67 | 32.65 | 0 |
| Fly Agar (g/L) | 15 | 15 | 15 | 15 | 15 |
| Acid Mix (ml/L) | 10 | 10 | 10 | 10 | 10 |

| Nutritional geometry. Isocaloric group 3 (696.53 calories per L) | | | | | | | |
| --- | --- | --- | --- | --- | --- | --- | --- |
| **Ingredients** | No yeast | Y:S = 1:14 | Extra | Extra | Y:S = 1:1.59 | Y:S = 1:0.7 | No sugar |
| Brewer’s yeast (g/L) | 0 | 11.85 | 25 | 46.67 | 69.57 | 46.67 | 79.32 |
| Sucrose (g/L) | 179.98 | 168.1 | 155 | 133 | 110.43 | 32.65 | 0 |
| Fly Agar (g/L) | 15 | 15 | 15 | 15 | 15 | 15 | 15 |
| Acid Mix (ml/L) | 10 | 10 | 10 | 10 | 10 | 10 | 10 |

| Nutritional geometry. Isocaloric group 4 (1060.38 calories per L) | | | | | |
| --- | --- | --- | --- | --- | --- |
| **Ingredients** | No yeast | Y:S = 1:14 | Y:S = 1:1.59 | Y:S = 1:0.7 | No sugar |
| Brewer’s yeast (g/L) | 0 | 18.04 | 105.9 | 161.20 | 274 |
| Sucrose (g/L) | 274 | 255.96 | 168.1 | 112.8 | 0 |
| Fly Agar (g/L) | 15 | 15 | 15 | 15 | 15 |
| Acid Mix (ml/L) | 10 | 10 | 10 | 10 | 10 |

| Nutritional geometry. Extra diets | | | | | | |
| --- | --- | --- | --- | --- | --- | --- |
| **Ingredients** |  |  |  |  |  |  |
| Brewer’s yeast (g/L) | 46.67 | 46.67 | 46.67 | 105.9 | 161.20 | 161.20 |
| Sucrose (g/L) | 74.1 | 168.1 | 220 | 220 | 168.1 | 255.96 |
| Fly Agar (g/L) | 15 | 15 | 15 | 15 | 15 | 15 |
| Acid Mix (ml/L) | 10 | 10 | 10 | 10 | 10 | 10 |

| Figure 2E diets (Base/proteins/AAs) | | | | | |
| --- | --- | --- | --- | --- | --- |
| **Ingredients** | S74Y0 (A), base diet | A+Cas2 | A+Cas4 | A+AA | A+AA2 |
| Brewer’s yeast (g/L) | 0 | 0 | 0 | 0 | 0 |
| Sucrose (g/L) | 74.1 | 74.1 | 74.1 | 74.1 | 74.1 |
| Fly Agar (g/L) | 15 | 15 | 15 | 15 | 15 |
| Acid Mix (ml/L) | 10 | 10 | 10 | 10 | 10 |
| Additional Components Proteins/AA (g/L) |  | 90 Casein | 180 Casein | 94 AA mix | 188 AA mix |

| Figure 2E diets (Lipids/Cholesterol) | | | | |
| --- | --- | --- | --- | --- |
| **Ingredients** | A+L4 | A+Ch0.4 | A+L2+Ch0.2 | A+L4+Ch0.4 |
| Brewer’s yeast (g/L) | 0 | 0 | 0 | 0 |
| Sucrose (g/L) | 74.1 | 74.1 | 74.1 | 74.1 |
| Fly Agar (g/L) | 15 | 15 | 15 | 15 |
| Acid Mix (ml/L) | 10 | 10 | 10 | 10 |
| Additional Components Lard (g/L) | 40 lard |  | 20 lard | 40 lard |
| Additional Components Cholesterol (g/L) |  | 0.4 Cholesterol | 0.2 Cholesterol | 0.4 cholesterol |

| Figure 2E diets (Vitamins/All ingredients) | | | | |
| --- | --- | --- | --- | --- |
| **Ingredients** | A+V2 | A+V4 | A+C4+L4+Ch0.4 | A+C4+L4+Ch0.4+V2 |
| Brewer’s yeast (g/L) | 0 | 0 | 0 | 0 |
| Sucrose (g/L) | 74.1 | 74.1 | 74.1 | 74.1 |
| Fly Agar (g/L) | 15 | 15 | 15 | 15 |
| Acid Mix (ml/L) | 10 | 10 | 10 | 10 |
| Additional Components Proteins/AA (g/L) |  |  | 180 Casein | 180 Casein |
| Additional Components Lard (g/L) |  |  | 40 lard | 40 lard |
| Additional Components Cholesterol (g/L) |  |  | 0.4 Cholesterol | 0.4 Cholesterol |
| Additional Components Vitamin mix (g/L) | 12.8 Vitamin/mineral Mix | 25.6 Vitamin/mineral Mix |  | 12.8 Vitamin/mineral Mix |

| Figure 2F | | | |
| --- | --- | --- | --- |
| **Ingredients** | Yeast:Lipid 0:1 | Yeast:Lipid 1:14 | Yeast:Lipid 1:0.7 |
| Brewer’s yeast (g/L) | 0 | 11.85 | 105.9 |
| Sucrose (g/L) | 0 | 0 | 0 |
| Fly Agar (g/L) | 15 | 15 | 15 |
| Acid Mix (ml/L) | 10 | 10 | 10 |
| Additional Components Lard (g/L) | 75.45 (Lard) | 70.48 (Lard) | 31.07 (Lard) |

| Figure 2 - figure supplement 2A (1) | | | | | |
| --- | --- | --- | --- | --- | --- |
| **Ingredients** | HS + inulin | HS + pectin | HS + pectin2 | HS + cellulose | HS + cellulose2 |
| Brewer’s yeast (g/L) | 11.85 | 11.85 | 11.85 | 11.85 | 11.85 |
| Sucrose (g/L) | 168.1 | 168.1 | 168.1 | 168.1 | 168.1 |
| Fly Agar (g/L) | 15 | 15 | 15 | 15 | 15 |
| Acid Mix (ml/L) | 10 | 10 | 10 | 10 | 10 |
| Additional Components Fibers (g/L) | 28.59 inulin | 28.59 pectin | 57.19 pectin | 28.59 cellulose | 57.19 cellulose |

| Figure 2 - figure supplement 2A (2) | | | | |
| --- | --- | --- | --- | --- |
| **Ingredients** | HS + inulin + pectin + cellulose (All fibers, AF) | HS + Pectin2 + Cellulose2 | HY + AF | HY + PC2 |
| Brewer’s yeast (g/L) | 11.85 | 11.85 | 105.9 | 105.9 |
| Sucrose (g/L) | 168.1 | 168.1 | 74.1 | 74.1 |
| Fly Agar (g/L) | 15 | 15 | 15 | 15 |
| Acid Mix (ml/L) | 10 | 10 | 10 | 10 |
| Additional Components Fibers (g/L) | 19.06 of each fiber | 28.59 of each fiber | 19.06 each | 28.59 of each fiber |

| Figure 2 - figure supplement 2B (1) | | | | |
| --- | --- | --- | --- | --- |
| **Ingredients** | HS agar 0.5% | HS Agar 1% | HS (1.5%) | HS Agar 3% |
| Brewer’s yeast (g/L) | 11.85 | 11.85 | 11.85 | 11.85 |
| Sucrose (g/L) | 168.1 | 168.1 | 168.1 | 168.1 |
| Fly Agar (g/L) | 5 | 10 | 15 | 30 |
| Acid Mix (ml/L) | 10 | 10 | 10 | 10 |

| Figure 2 - figure supplement 2B (2) | | | | |
| --- | --- | --- | --- | --- |
| **Ingredients** | HY agar 0.5% | HY Agar 1% | HY (1.5%) | HY Agar 3% |
| Brewer’s yeast (g/L) | 105.9 | 105.9 | 105.9 | 105.9 |
| Sucrose (g/L) | 74.1 | 74.1 | 74.1 | 74.1 |
| Fly Agar (g/L) | 5 | 10 | 15 | 30 |
| Acid Mix (ml/L) | 10 | 10 | 10 | 10 |
