# Supplementary material for: Multiscale analysis reveals that diet-dependent midgut plasticity emerges from alterations in both stem cell niche coupling and enterocyte size
Source: eLife. 2021 Sep 23;10:e64125. doi: 10.7554/eLife.64125 (PMC8528489; doi:10.7554/eLife.64125)
Supplement: Supplementary file 2. [file elife-64125-supp2.zip › Bonfini_script_GutPlasticity_diet.html]

 

 

 

 
 
 


 Bonfini et al, 2021 - Multiscale analysis reveals that diet-dependent gut plasticity emerges from alterations in both stem cell niche coupling and enterocyte size. 

 
 
 
 
 
 
 
 
 
 
 
 
 
 
 

 


 
 
 


 


 


 

 

 
 


 


 

 


 


 
 
 
 
 
 

 


 

 
  Code     
 
  Show All Code  
  Hide All Code  
 
 


 Bonfini et al, 2021 - Multiscale analysis reveals that diet-dependent gut plasticity emerges from alterations in both stem cell niche coupling and enterocyte size. 

 


 
 
 
 Table of contents 
 
 
 Library, Data import and reformatting 
 
 Library 
       library (devtools) 
    library (reshape2) 
    library (lattice) 
    library (MASS) 
    library (car) 
    library (lmtest) 
    library (ggplot2) 
    library (survival) 
    library (plotrix) 
    library (grid) 
    library (gridExtra) 
    library (agricolae) 
    library (nparLD) 
    library (psych) 
    library (doBy) 
    library (xlsxjars) 
    library (xlsx) 
    library (dplyr) 
    library (stringr) 
    library (scales) 
    library (tidyr) 
    library (phia) 
    library (data.table) 
    library (spaMM) 
    library (lme4) 
    library (fields) 
    library (EBImage) 
    library (gplots) 
    library (RColorBrewer) 
    library (gridGraphics) 
    library (fields) 
    library (multcomp) 
    library (ggrepel) 
    library (metR) 
    library (forcats) 
    library (ggh4x) #remotes::install_github("teunbrand/ggh4x")  
    library (GenomicRanges) 
    library (DESeq)    
  ## Error in library(DESeq): there is no package called 'DESeq'  
       library (RColorBrewer) 
    library (coxme) 
    library (ggplotify) 
    library (base2grob) 
    library (knitr) 
    library (kableExtra) 
    library (plotfunctions) 
    library (ggsignif) 
    #Function to include factor that are NOT in a list  
    '%!in%'   =   function (x,y) ! ( '%in%' (x,y)) 
    
    #Function to Grab graph and display it as a ggplot graph  
   grab_grob  =   function (){ 
      grid.echo () 
      grid.grab () 
   } 
    
    
    #Function to calculate standard deviaion   
   sd  =   function (x)  sqrt ( var (x, na.rm= T)) 
    
    
    #Function to calculate standard error   
   se  =   function (x)  sqrt ( var (x, na.rm= T) /  length (x)) 
    
    # Function to graph survival with ggplot and displaying the checkpoints  
   ggplotprep2  <-   function (x, times){ 
      #spreading the surfit dataframe into dataframe per day.   
     d  <-   data.frame ( condition=  rep ( names (x $ strata), x $ strata),  time= x $ time,  survival= x $ surv,  upper= x $ upper,  lower= x $ lower) 
      # function to add time point 0   
     fillup0  <-   function (s)  rbind ( c ( condition= s,  time=  0 ,  survival=  1 ,  upper=  1 ,  lower=  1 ), d[d $ condition == s, ],  deparse.level =   0 ) 
      
      # function to determine the missing time points  
     indexes  <-   function (x, time) { 
        if (x %in% time)  return (x) 
        return (time[ which.min ( abs (time[time < x] - x))]) 
     } 
      #Function to complete the missing time points  
     fillup  <-   function (s) { 
       d.temp  <-  d[d $ condition == s, ] 
       time  <-   as.numeric (d.temp $ time) 
       id  <-   sapply (times, indexes,  time= time) 
       d.temp  <-  d.temp[ match (id, time), ] 
       d.temp $ time  <-  times 
        return (d.temp) 
     } 
      
      if (times[ 1 ] ==  0 ) d  <-   do.call ( "rbind" ,  sapply ( names (x $ strata), fillup0,  simplify= F)) 
     d  <-   do.call ( "rbind" ,  sapply ( names (x $ strata), fillup,  simplify= F)) 
     clean.name  <-   function (name)  unlist ( lapply ( strsplit ( as.character (name),  split=  "=" ),  function (x) x[ 2 ])) 
     d  <-   data.frame ( Condition=  clean.name (d $ condition),  Time=  as.numeric (d $ time),  Survival=  as.numeric (d $ survival),  upper=  as.numeric (d $ upper),  lower=  as.numeric (d $ lower)) 
      return (d) 
   }   
    
    
    #function to select colours for GF-style plot (function mapping colors)  
   seeMahPal  <-   function (x, pal){ 
       pal[ round (x)] 
   } 
    
        #a function to take x,y,z  
        #and return a GF-style plot with points per diet  
   geomPlotta  <-   function (x,y,z,alf,...){ 
       dat  <-   data.frame ( x= x,  y= y,  z= z) 
       d.means  <-   aggregate (z  ~  x  *  y, dat, mean) 
       surf.te  <-   Tps ( cbind (dat $ x, dat $ y), dat $ z,  lambda =   0 )  
    
       experiColours  <-   data.frame ( z= d.means $ z,  rank=  rank (d.means $ z),  rnd=  round (d.means $ z),  rankRnd=  rank ( round (d.means $ z))) 
       mahPal  <-   colorRampPalette ( c ( "darkblue" ,  "blue" ,  "turquoise" ,  "yellow" ,  "orange" ,  "red" ,  "darkred" ))( max (experiColours $ rank))  #Decide colors  
    
   d.means $ colour  <-   seeMahPal ( x= d.means $ z,  pal= mahPal) 
    
    surface ( predictSurface (surf.te,  extrap= F),  col=  alpha (mahPal, alf), ...) 
    points (d.means $ x, d.means $ y,  bg=  seeMahPal ( x= experiColours $ rank,  pal= mahPal),  col=  "white" ,  pch=  21 ,  cex=  1 , ...) 
   } 
    
    
   left  =   function (text, num_char) { 
      substr (text,  1 , num_char) 
   } 
     
   mid  =   function (text, start_num, num_char) { 
      substr (text, start_num, start_num  +  num_char  -   1 ) 
   } 
     
   right  =   function (text, num_char) { 
      substr (text,  nchar (text)  -  (num_char -1 ),  nchar (text)) 
   }    
 
 
 Font size and palettes 
      SuperSmallfont =   6  
   xSmallfont  =   8  
   Smallfont =   10  
   Mediumfont =   12  
   Largefont =   14  
   verylargefont  =   16  
   pointsize =   0.7  
   linesize =  0.35  
   meansize  =   1.5  
   Margin =  c ( 0 , 0 , 0 , 0 ) 
    
   fontsizeaxes  =   14  
   fontsizeaxes2  =   10  
    
   palette_diet_2  =   c ( "#FFB4B4" ,  "#C3E6FC" ) 
   palette_component_3  =   c ( "#f4ead0" , "#2d5ad7" , "gold" ) 
   palette_mean  =   c ( "yellow" , "green" , "red" , "white" , "magenta" , "skyblue" ,  "blue" ,  "deeppink" ,  "gold" ) 
   cbbPalette_4  =   c ( "#BDE6BD" ,  "#C3E6FC" ,  "#FFE5E5" ,  "#E5E5FF" )  #Green eclosion, HY, HYtoHS, HStoHY  
   cbbHS_HStoHY  =   c ( "#FFB4B4" , "#E5E5FF" ) 
   cbbHY_HYtoHS  =   c ( "#C3E6FC" , "#FFE5E5" )    
 
 
 Import dataset 
      path.to.data  =   "D:/Dropbox/z_ Ale Shared work/z_Nutrition Paper Markdown/Ale/Revision/"  
    
    rm (d,path) 
   d  =   list () 
   path  =   list () 
    for (f  in   list.files ( path= path.to.data, pattern=  "*.csv$" , recursive= T, full.names= T)) { 
     nom  =   gsub ( ".*/(.*).csv" , "  \\  1" ,f)     
      cat (nom, "  \n  " ) 
     path[[nom]]  =   gsub ( "(.*)/.*csv" , "  \\  1/" ,f) 
     d[[nom]]  =   read.table (f, header= T, sep=  "," ) 
   }    
 
 
 Data meaning - DGRP GWAS 
      wolb  =  d[[ "DGRP_wolbachia_DFD" ]] 
    colnames (wolb)  =   c ( "dgrp_id" ,  "wolbachia" ) 
   wolb $ dgrp_id  =   gsub ( "line_" ,  "DGRP-" , wolb $ dgrp_id) 
    
   weight  =  d[[ "weights" ]] 
   decode  =  d[[ "stockDecode" ]] 
    
   weight $ mg  =  weight $ weightPerFlyGram  *   1000  
   weight $ diet  =   tolower (weight $ diet) 
   decode $ shortID  =   as.factor ( as.character (decode $ shortID)) 
   weight $ stockNumber  =   as.factor ( as.character (weight $ dgrp)) 
   weight  =   merge (weight, decode,  by.x=  "stockNumber" ,  by.y=  "shortID" ) 
    colnames (weight)  =   tolower ( colnames (weight)) 
   weight  =  weight[, which ( !  colnames (weight)  %in%   c ( "stockNumber" ,  "dgrp.x" ))] 
    colnames (weight)[ which ( colnames (weight)  ==   "dgrp.y" )]  =   "dgrp"  
   weight $ dgrp_number  =   substr ( as.character (weight $ dgrp),  1 ,  3 ) 
   weight $ dgrpDiet  =   factor ( paste (weight $ dgrp_number, weight $ diet,  sep=  "_" )) 
    
   tab_GWAS_gut  =  d[[ "1F - G" ]] 
    
    #edit the data  
    table ( complete.cases (tab_GWAS_gut)) 
    #str(tab_GWAS_gut)  
   tab_GWAS_gut =   mutate_if (tab_GWAS_gut,is.integer,as.factor) 
    
    colnames (tab_GWAS_gut)  =   tolower ( colnames (tab_GWAS_gut)) 
   tab_GWAS_gut  =  tab_GWAS_gut[, !  colnames (tab_GWAS_gut)  %in%   c ( "notes" ,  "image" ,  "bloomington_id" )] 
    
    #remove the samples that subsequently proved crazy  
   tab_GWAS_gut  =   subset (tab_GWAS_gut, anteriorwidth  <   1000 ) 
   tab_GWAS_gut  =   subset (tab_GWAS_gut, middlelength  <   1500 ) 
    
    #remove lines that don't appear in both diets  
   dgrpLines  =   levels (tab_GWAS_gut $ dgrp_number) 
   yDat  =   droplevels ( subset (tab_GWAS_gut, diet ==  "y" )) 
   xDat  =   droplevels ( subset (tab_GWAS_gut, diet ==  "x" )) 
    length (dgrpLines) 
   dgrpLines  =  dgrpLines[dgrpLines  %in%  yDat $ dgrp_number] 
    #length(dgrpLines)  
   dgrpLines  =  dgrpLines[dgrpLines  %in%  xDat $ dgrp_number] 
    #length(dgrpLines)  
    
   tab_GWAS_gut  =   droplevels ( subset (tab_GWAS_gut, dgrp_number  %in%  dgrpLines)) 
   yDat  =   droplevels ( subset (yDat, dgrp_number  %in%  dgrpLines)) 
   xDat  =   droplevels ( subset (xDat, dgrp_number  %in%  dgrpLines)) 
    
    #link up Wolbachia  
   tab_GWAS_gut  =   merge (tab_GWAS_gut, wolb,  by=  "dgrp_id" ) 
   tab_GWAS_gut =   mutate_if (tab_GWAS_gut,is.character,as.factor)    
 
 
 
  1  Figure 1. Diet composition affects size and regional allometry of the midgut 
 
  1.1  Figure 1 - main 
 
  1.1.1  Figure 1A 
 
  Illustration of general dietary treatment design.  Flies were reared on pre-experiment diet during development. At eclosion, flies were allocated to either HS or HY before midgut dissection at 5 days post eclosion. 
 
      img1A  =   readImage ( "D:/Dropbox/z_ Ale Shared work/z_Nutrition Paper Markdown/Ale/Revision/1A.jpg" )  
   gob_imageFig1A  =   rasterGrob (img1A) 
    grid.draw (gob_imageFig1A)    
   
 
 
  1.1.2  Figure 1B 
 
  Nutritional composition  (proteins, carbohydrates, and lipids) of the two isocaloric diets used as a basis for this study as calories per liter of food: enriched in sugars (High sugar, HS) or yeast (High yeast, HY). 
 
      general_info_diet  = d[[ "1B" ]] 
   general_info_diet  =   mutate_if (general_info_diet,is.character,as.factor) 
    
   general_info_diet $ Component  <-   factor (general_info_diet $ Component,  levels =   c ( "Lipids" , "Proteins" , "Carbohydrates" )) 
   Limits  =   c ( "Lipids" , "Proteins" , "Carbohydrates" ) 
   Labels  =   c ( "Lipids" , "Proteins" , "Carbohydrates" ) 
    
   Plot_Fig1B =  
    ggplot (general_info_diet, aes ( x= Diet, y= Calories.contributed)) +  
    geom_bar ( stat=  "identity" , aes ( fill= Component), color=  "black" , width= . 90 ) +  
      scale_fill_manual ( limits= Limits, 
                          values= palette_component_3, 
                          labels= Labels) +  
      scale_x_discrete ( "" , 
                         limits=  c ( "HS" ,  "HY" ), 
                         breaks=  c ( "HS" ,  "HY" )) +  
      scale_y_continuous ( "Calories/L of food" , 
                         breaks=  c ( seq ( 0 , 650 , by=  200 ), 696 )) +  
      theme ( axis.title.x =   element_text ( size= Smallfont, colour=  "black" ), 
            axis.title.y =    element_text ( size= Smallfont, colour=  "black" ), 
            axis.line.x =   element_line ( colour=  "black" ), 
            axis.line.y =   element_line ( colour=  "black" ), 
            axis.ticks.x =   element_line (), 
            axis.ticks.y =   element_line (), 
            axis.text.x =   element_text ( size= Smallfont, colour=  "black" ), 
            axis.text.y =   element_text ( size= Smallfont, colour=  "black" ), 
            panel.grid =   element_blank (), 
            plot.margin =   unit ( c ( 0 , 0 , 0 , 0 ),  "cm" ), 
            legend.direction =   "vertical" ,  
            legend.box =   "vertical" , 
            legend.position =   c ( 0.5 , -  0.3 ), 
            legend.key.height =   unit ( 0.3 ,  "cm" ), 
            legend.key.width=   unit ( 0.3 ,  "cm" ), 
            legend.margin=  margin ( t=  -  0.9 ,  r=  -  0 ,  b=  -  0 ,  l=  -  0 ,  unit=  "cm" ), 
            legend.title =   element_blank (),  
            legend.key =   element_rect ( colour =   'white' ,  fill =   "white" ,  linetype=  'dashed' ), 
            legend.text =   element_text ( size= xSmallfont), 
            legend.background =   element_rect ( fill=  NA ), 
            strip.text.x =   element_text ( size = Smallfont,  colour =   "black" , face=  "italic" ), 
            strip.text.y =   element_text ( size = Smallfont,  colour =   "black" , face=  "italic" ), 
            strip.background =   element_rect ( fill=  NA ,  colour=  "black" ), 
            strip.placement=  "outside" , 
            panel.background =   element_rect ( fill=  "transparent" )) +  
      guides ( fill=  guide_legend ( ncol=  1 )) 
   Plot_Fig1B    
   
 
 
  1.1.3  Figure 1C-D 
 
 Canton S (Cs) flies fed on HS diet (C, first image) have shorter midguts than flies on HY (D, Second image).  Complete graphical annotation can be found in manuscript figures  
 
   
   
 
 
  1.1.4  Figure 1E 
 
 Quantification of midgut length for HS vs HY at 5 days post eclosion. 
 
      Length_HSHY  =   
     d[[ "1E - 1S1A" ]] %>%  
      mutate_at ( vars ( starts_with ( "Total" )), ~ . /  1000 ) %>%  
      mutate_if (is.character,as.factor) %>%  
      mutate_if (is.integer,as.factor) %>%  
     dplyr ::  rename ( Total_Length_mm= Total.L, 
                    Total_width_mm= Total.W, 
                    Day_of_treatment= Day) 
    
   Sample_size =  
     Length_HSHY %>%  
      group_by (Diet) %>%  
      summarise ( Sample_size=  n ()) 
    
   Averages  <-   summarise ( group_by (Length_HSHY, Diet),  mean =   mean (Total_Length_mm,  na.rm =   TRUE )) 
    
    ###Stats  
   mod.gen  =   fitme ( log (Total_Length_mm)  ~   Diet  +  ( 1   |  Repeat),  data =  Length_HSHY) 
    shapiro.test ( residuals (mod.gen))     
  ## 
##  Shapiro-Wilk normality test
## 
## data:  residuals(mod.gen)
## W = 0.98954, p-value = 0.9657  
       bptest ( log (Total_Length_mm)  ~  Diet  +  ( 1   /  Repeat),  data =  Length_HSHY)     
  ## 
##  studentized Breusch-Pagan test
## 
## data:  log(Total_Length_mm) ~ Diet + (1/Repeat)
## BP = 0.64174, df = 1, p-value = 0.4231  
      mod.gen1  =   fitme ( log (Total_Length_mm)  ~   1   +  ( 1   |  Repeat),  data =  Length_HSHY)  
   test  =   anova (mod.gen, mod.gen1)  
   Chi2_LRT_growth  =   2  * (mod.gen $ APHLs[[ "p_v" ]] - mod.gen1 $ APHLs[[ "p_v" ]]) 
    
    #Now we make a tab with the results  
   tab_stat  =   data.frame ( Variable =   as.character ( "HS vs HY" ), 
                          Rep =   nlevels (Length_HSHY $ Repeat), 
                          chi2_LR =   format ( as.numeric (test $ basicLRT $ chi2_LR),  digits =   2 ), 
                          intercept =   format (mod.gen $ fixef[ 1 ], digits=  3 ), 
                          estimate =   format (mod.gen $ fixef[ 2 ], digits=  3 ), 
                          df =   as.numeric (test $ basicLRT $ df), 
                          Pvalue =   as.numeric ( format ( pchisq (Chi2_LRT_growth, df=  1 , lower.tail =  F), digits=  2 ))) 
   tab_stat $ sig  =   ifelse (tab_stat $ Pvalue  <   0.05   &  tab_stat $ Pvalue  >   0.01 ,  "*" , 
                          ifelse (tab_stat $ Pvalue  <   0.01   &  tab_stat $ Pvalue  >   0.001 ,  "**" , 
                                 ifelse (tab_stat $ Pvalue  <   0.001 ,  "***" ,  "" ))) 
    
   tab_stat %>%  
      kable ( col.names =   c ( "Comparison" ,  "Replicates" ,  "Chi2" , "Intercept" , "Estimate" , "df"  , "p-value" , "Signif." ), row.names =   FALSE )  %>%  
      add_header_above ( c ( "log(Total_Length_mm) ~  Diet + (1 | Repeat)"   =   8 )) %>%  
      kable_styling ( bootstrap_options =   c ( "striped" ,  "hover" ,  "condensed" ),  full_width =  F)    
 
 
 
 
 
log(Total_Length_mm) ~ Diet + (1 | Repeat)
 
 
 
 
 
Comparison
 
 
Replicates
 
 
Chi2
 
 
Intercept
 
 
Estimate
 
 
df
 
 
p-value
 
 
Signif.
 
 
 
 
 
 
HS vs HY
 
 
3
 
 
35
 
 
1.45
 
 
0.266
 
 
1
 
 
0
 
 
***
 
 
 
 
       ### Plot  
   Limits  =   c ( "HS" ,  "HY" ) 
   z =   max (Length_HSHY $ Total_Length_mm) 
   Plot_Fig1E =  
      ggplot (Length_HSHY,  aes ( x =  Diet,  y =  Total_Length_mm)) +   
      geom_violin ( aes ( fill =  Diet),  draw_quantiles =   c ( 0.25 ,  0.5 ,  0.75 ),  colour =   "black" ,  size =   0.2 , adjust =   0.8 )  +  
      geom_dotplot (  colour =   "black" ,  fill =   "white" ,  binaxis =   "y" ,  stackdir =   "center" ,  binwidth =  z /  50 )  +   
      geom_text ( data =  Sample_size,  mapping =   aes ( x =  Diet,  y =   2.3 ,  label =   paste ( "(" ,Sample_size, ")" , sep=  "" )), size=  3 ) +  
      geom_signif ( annotation =   formatC ( paste ( "p=" ,tab_stat $ Pvalue),  digits =   2 ),  textsize =   3 ,  y_position =   7.3 ,  xmin =   1 ,  xmax =   2 ,  tip_length =   c ( 0.02 ,  0.02 ),  vjust =   -  0.2 ) +  
      scale_fill_manual ( limits= Limits, 
                        values= palette_diet_2) +  
      scale_x_discrete ( "" , 
                       limits=  c ( "HS" ,  "HY" ), 
                       labels=  c ( "HS" ,  "HY" )) +  
      scale_y_continuous ( "Midgut length (mm)" , 
                         limits=  c ( 2 , 8 ), 
                         breaks=  seq ( 2 , 8 , by=  1 ), 
                         minor_breaks =   seq ( 3 ,  7 , by=   1 )) +  
      stat_summary ( fun =  mean,  geom =   "point" ,  size =   3 ,  shape =   18 ,  colour =   "black" ,  aes ( group =  Repeat))  +  
      stat_summary ( fun =  mean,  geom =   "point" ,  size =   2 ,  shape =   18 ,  aes ( group =  Repeat,  colour =  Repeat))  +  
      scale_color_manual ( values =  palette_mean)  +  
      theme ( aspect.ratio=  2 , 
            panel.grid.major.y =   element_line ( colour =   grey ( 0.45 ),  linetype =   "dashed" ,  size =   0.2 ), 
            panel.background =   element_blank (), 
            axis.title.x =   element_text ( size= Smallfont, colour=  "black" ), 
            axis.title.y =   element_text ( size= Smallfont, colour=  "black" ),  
            axis.line.x =   element_line ( colour=  "black" , size=  0.75 ), 
            axis.line.y =   element_line ( colour=  "black" , size=  0.75 ), 
            axis.ticks.x =   element_line ( size =   0.75 ), 
            axis.ticks.y =   element_line ( size =   0.75 ), 
            axis.text.x =   element_text ( size= Smallfont, colour=  "black" ), 
            axis.text.y =   element_text ( size= Smallfont, colour=  "black" ), 
            plot.margin =   unit (Margin,  "cm" ), 
            legend.direction =   "vertical" ,  
            legend.box =   "horizontal" , 
            legend.position =   "none" , 
            legend.key.height =   unit ( 0.4 ,  "cm" ), 
            legend.key.width=   unit ( 0.6 ,  "cm" ), 
            legend.title =   element_text ( face=  "italic" , size= Smallfont),  
            legend.key =   element_rect ( colour =   'white' ,  fill =   "white" ,  linetype=  'dashed' ), 
            legend.text =   element_text ( size= SuperSmallfont), 
            legend.background =   element_rect ( fill=  NA )) 
    
        
      
    
    
    
    #+  
     # annotate("segment", x = 1, xend = 2, y = 7.2, yend = 7.2,  
      #colour = "black", size =1.5)  
    
    
    
   Plot_Fig1E    
   
 
 
  1.1.5  Figure 1F 
 
  Midgut length response to diet is strongly variable across the DGRP , with HY being generally longer than HS (i.e. the ratio length on HY/length on HS is between 1 and 1.4). 
 
      tab_GWAS_gut_mean  =   
     tab_GWAS_gut %>%  
      group_by (dgrp_number,diet) %>%  
      summarise ( mean_gut_length=  mean (totallength, na.rm= T)) %>%  
      spread (diet,mean_gut_length) %>%  
     dplyr ::  rename ( Mean_length_HS= x, 
             Mean_length_HY= y) %>%  
      mutate ( Ratio =  Mean_length_HY / Mean_length_HS) 
    
   tab_GWAS_gut_se  =   
     tab_GWAS_gut %>%  
      group_by (dgrp_number,diet) %>%  
      summarise ( se_gut_length =   se (totallength)) %>%  
      spread (diet,se_gut_length) %>%  
     dplyr ::  rename ( SE_length_HS= x, 
             SE_length_HY= y) 
    
   tab_GWAS_gut_mean =   left_join (tab_GWAS_gut_mean,tab_GWAS_gut_se) 
    
   colors =  c ( "HS"  =  "#FFB4B4" , "HY"  =  "#C3E6FC" , "Ratio"  =  "black" )  
    
   plot_ratio_DGRP =  
    ggplot (tab_GWAS_gut_mean, aes ( x =   reorder (dgrp_number,Ratio)))  +   
      geom_point ( aes ( y= Mean_length_HS /  1000 , colour=  "HS" ), stat=  "identity" , size=  0.7 , shape=  16 ) +  
      geom_errorbar ( aes ( ymax =  (Mean_length_HS +  SE_length_HS) /  1000  ,  ymin =  (Mean_length_HS  -  SE_length_HS) /  1000  , colour=  "HS" ), width=  0.1 ,  show.legend=  FALSE ) +  
      geom_point ( aes ( y= Mean_length_HY /  1000 , colour=  "HY" ), stat=  "identity" , size=  0.7 , shape=  16 ) +  
      geom_errorbar ( aes ( ymax =  (Mean_length_HY +  SE_length_HY) /  1000  ,  ymin =  (Mean_length_HY  -  SE_length_HY) /  1000  , colour=  "HY" ), width=  0.1 ,  show.legend=  FALSE ) +  
      geom_point ( aes ( y= Ratio *  3.9 , colour=  "Ratio" ), shape=  17 , size=  0.7 ) +  
      geom_hline ( yintercept=  3.9 , linetype=  2 ) +  
      scale_y_continuous ( "Midgut length (mm)  \n   [mean \u00B1 se]" , 
                         limits=  c ( 3 , 7.2 ), 
                         sec.axis =   sec_axis ( ~ . /  3.9 ,  name =   "Ratio (HY/HS)" ,  breaks =   seq ( 0.8 , 1.8 , 0.2 ))) +  
      scale_x_discrete ( "DGRP lines" , expand=  c ( 0.03 , 0.03 )) +  
      scale_color_manual ( values =  colors ) +  
      theme ( panel.background =   element_blank (), 
           ( panel.border =   element_blank ()), 
            axis.title.x =   element_text ( size= Smallfont, colour=  "black" ), 
            axis.title.y =   element_text ( size= Smallfont, colour=  "black" ),  
            axis.line.x =   element_line ( colour=  "black" , size=  0.75 ), 
            axis.line.y =   element_line ( colour=  "black" , size=  0.75 ), 
            axis.ticks.x =   element_blank (), 
            axis.ticks.y =   element_line ( size =   0.75 ), 
            axis.text.x =   element_blank (), 
            axis.text.y =   element_text ( size= Smallfont, colour=  "black" ), 
            plot.margin =   unit (Margin,  "cm" ), 
            legend.direction =   "horizontal" ,  
            legend.box =   "horizontal" , 
            legend.position =   c ( 0.25 , 0.98 ), 
            legend.key.height =   unit ( 0.4 ,  "cm" ), 
            legend.key.width=   unit ( 0.4 ,  "cm" ), 
            legend.title =   element_blank (),  
            legend.key =   element_rect ( colour =   'white' ,  fill =   "white" ,  linetype=  'dashed' ), 
            legend.text =   element_text ( size= Smallfont), 
            legend.background =   element_rect ( fill=  NA )) +  
      guides ( color=  guide_legend ( ncol=  3 )) 
    
   plot_ratio_DGRP    
   
      list_lines  =   unique (tab_GWAS_gut $ dgrp_id) 
   Tab  =   NULL  
    for (i  in  list_lines){ 
     tmp =   subset (tab_GWAS_gut,dgrp_id == i) 
   sample_size =  tmp  %>%   group_by (diet) %>%  summarize ( n=  n ()) 
          
   test =   t.test (totallength ~ diet , data= tmp)   
   Tab  =   rbind (Tab,  c (i,test $ parameter,test $ statistic,test $ p.value,test $ estimate,sample_size[ 1 , 2 ],sample_size[ 2 , 2 ])) 
   } 
    
    colnames (Tab) =  c ( "Line" , "df" , "t" , "Pvalue" , "Mean_HS" , "Mean_HY" , "Sample_size_HS" , "Sample_size_HY" ) 
   Tab  =   as.data.frame (Tab) %>%  
      mutate ( Pvalue=  as.numeric (Pvalue), 
             Mean_HS =  as.numeric (Mean_HS), 
             Mean_HY =  as.numeric (Mean_HY), 
             Difference =  Mean_HY - Mean_HS ) 
    
   Tab $ Pv_adjust  =   p.adjust (Tab $ Pvalue, method =   "BH" )  # Here I control   
    
    length ( which (Tab $ Pv_adjust >  0.05 )) 
    # 56 lines have no significant difference in size between diets  
    length ( which (Tab $ Pv_adjust <=  0.05 )) 
    #132 lines have a significant difference in size between diets  
    
   Tab_sign  =   subset (Tab,Pv_adjust <=  0.05 ) 
    
    length ( which (Tab_sign $ Difference <=  0 )) 
    # 0 line was significantly smaller on HY  
    
    length ( which (Tab_sign $ Difference >  0 )) 
    # 132 lines (i.e. all of those that were different) were significantly larger on HY     
 
 
  1.1.6  Figure 1G 
 
  Midgut re-sizing is allometric between regions of the midgut.  Posterior midguts of flies fed HY exhibit a greater increase than anterior regions. 
 
      tab_GWAS_gut =  
     tab_GWAS_gut %>%  
      mutate ( allometry= posteriorlength / anteriorlength) 
    
   tab_GWAS_gut_allometry_mean =  
     tab_GWAS_gut %>%  
      group_by (diet,dgrp_number) %>%  
      summarise ( mean_allometry=  mean (allometry, na.rm= T)) 
    
   Sample_size =  
     tab_GWAS_gut_allometry_mean %>%  
      group_by (diet) %>%  
      summarise ( Sample_size=  n ()) 
    
    ###Stats  
    
   mod.gen  =   fitme (mean_allometry  ~   diet  +  ( 1   |  dgrp_number) ,  data =  tab_GWAS_gut_allometry_mean) 
    shapiro.test ( residuals (mod.gen))     
  ## 
##  Shapiro-Wilk normality test
## 
## data:  residuals(mod.gen)
## W = 0.9809, p-value = 7.05e-05  
       bptest (mean_allometry  ~  diet  +  ( 1   /  dgrp_number) ,  data =  tab_GWAS_gut_allometry_mean)     
  ## 
##  studentized Breusch-Pagan test
## 
## data:  mean_allometry ~ diet + (1/dgrp_number)
## BP = 0.051149, df = 1, p-value = 0.8211  
      mod.gen1  =   fitme (mean_allometry  ~   1   +  ( 1   |  dgrp_number),  data =  tab_GWAS_gut_allometry_mean)  
   test  =   anova (mod.gen, mod.gen1)  
   Chi2_LRT_growth  =   2  * (mod.gen $ APHLs[[ "p_v" ]] - mod.gen1 $ APHLs[[ "p_v" ]]) 
    
    #Now we make a tab with the results  
   tab_stat  =   data.frame ( Variable =   as.character ( paste ( "HS vs HY" )), 
                                   Rep =   1 , 
                                   chi2_LR =   round ( as.numeric (test $ basicLRT $ chi2_LR),  digits =   2 ), 
                                   intercept =   format (mod.gen $ fixef[ 1 ], digits=  3 ), 
                                   estimate =   format (mod.gen $ fixef[ 2 ], digits=  3 ), 
                                   df =   as.numeric (test $ basicLRT $ df), 
                                   Pvalue =   as.numeric ( format ( pchisq (Chi2_LRT_growth, df=  1 , lower.tail =  F), digits=  2 ))) 
   tab_stat $ sig  =   ifelse (tab_stat $ Pvalue  <   0.05   &  tab_stat $ Pvalue  >   0.01 ,  "*" , 
                 ifelse (tab_stat $ Pvalue  <   0.01   &  tab_stat $ Pvalue  >   0.001 ,  "**" , 
                  ifelse (tab_stat $ Pvalue  <   0.001 ,  "***" ,  "" ))) 
    
   tab_stat %>%  
      kable ( col.names =   c ( "Comparison" ,  "Replicates" ,  "Chi2" , "Intercept" , "Estimate" , "df"  , "p-value" , "Signif." ), row.names =   FALSE )  %>%     add_header_above ( c ( "mean_allometry ~  diet + (1 | dgrp_number)"   =   8 )) %>%  
      kable_styling ( bootstrap_options =   c ( "striped" ,  "hover" ,  "condensed" ),  full_width =  F)    
 
 
 
 
 
mean_allometry ~ diet + (1 | dgrp_number)
 
 
 
 
 
Comparison
 
 
Replicates
 
 
Chi2
 
 
Intercept
 
 
Estimate
 
 
df
 
 
p-value
 
 
Signif.
 
 
 
 
 
 
HS vs HY
 
 
1
 
 
128.95
 
 
0.861
 
 
0.101
 
 
1
 
 
0
 
 
***
 
 
 
 
       ### Plot  
    
   Plot_Fig1G =  
      ggplot (tab_GWAS_gut_allometry_mean,  aes (diet,mean_allometry,  group= dgrp_number, color= diet))  +  
      geom_path ( size=  0.3 , color=  grey ( 0.65 )) +  
      geom_point ( shape=  16 , size=   1 ) +  
      scale_x_discrete ( "" , 
                       expand=  c ( 0.1 , 0.1 ), 
                       limits=  c ( "x" , "y" ), 
                       labels=  c ( "HS" ,  "HY" )) +  
      scale_y_continuous ( "Posterior / anterior length" , 
                         limits=  c ( 0.6 ,  1.4 ), 
                         breaks=  c ( c ( seq ( 0.5 , 1.4 , by=  0.1 )))) +  
      scale_color_manual ( limits=  c ( "x" , "y" ), 
                        values= palette_diet_2) +  
      annotate ( "text" ,  label=  paste ( "p=" ,tab_stat $ Pvalue, sep=  "" ),  x=   1.5 ,  y=  1.4 , size=  3 ) +  
      stat_summary ( fun =  mean,  aes ( group =   1 ), geom =   "point" ,  colour =   "black" ,  fill =   "yellow" ,  size =   3 ,  shape =   23 )  +  
      stat_summary ( fun= mean,  colour=  "black" ,  geom=  "line" ,  aes ( group =   1 ), size=  1 , linetype=  2 ) +  
      theme ( 
        axis.title.x =   element_text ( size= Smallfont), 
        axis.title.y =   element_text ( size= Smallfont), 
        axis.line.x =   element_line ( colour=  "black" , size=  0.75 ), 
        axis.line.y =   element_line ( colour=  "black" , size=  0.75 ), 
        axis.ticks.x =   element_line ( size =   0.75 ), 
        axis.ticks.y =   element_line ( size =   0.75 ), 
        axis.text.x =   element_text ( size= Smallfont, colour=  "black" ), 
        axis.text.y =   element_text ( size= Smallfont, colour=  "black" ), 
        legend.position =   "none" , 
        panel.background =   element_blank ()) 
    
   Plot_Fig1G    
   
 ##Export Figure 1 
 
 
 
  1.2  Figure 1 - figure supplement 1 
 
  1.2.1  Figure 1S1A 
 
 Canton S (Cs) flies fed HS diet have narrower midguts than those fed HY diet. Width was measured in three point along the gut (Region 2, 3 and 4, as visible in the yellow annotation in Figure 1C,D) and the sum of these three measurement was used as proxy for midgut width. Measurements are from the same guts as in Figure 1E 
 
      Length_HSHY  =   
     d[[ "1E - 1S1A" ]] %>%  
      mutate_at ( vars ( starts_with ( "Total" )), ~ . /  1000 ) %>%  
      mutate_if (is.character,as.factor) %>%  
      mutate_if (is.integer,as.factor) %>%  
     dplyr ::  rename ( Total_Length_mm= Total.L, 
             Total_width_mm= Total.W, 
             Day_of_treatment= Day) 
    
   Sample_size =  
     Length_HSHY %>%  
      group_by (Diet) %>%  
      summarise ( Sample_size=  n ()) 
    
    ###Stats  
    
   mod.gen  =   fitme ( log (Total_width_mm)  ~   Diet  +  ( 1   |  Repeat),  data =  Length_HSHY) 
    shapiro.test ( residuals (mod.gen))     
  ## 
##  Shapiro-Wilk normality test
## 
## data:  residuals(mod.gen)
## W = 0.95797, p-value = 0.1335  
       bptest ( log (Total_width_mm)  ~  Diet  +  ( 1   /  Repeat),  data =  Length_HSHY)     
  ## 
##  studentized Breusch-Pagan test
## 
## data:  log(Total_width_mm) ~ Diet + (1/Repeat)
## BP = 0.17388, df = 1, p-value = 0.6767  
      mod.gen1  =   fitme ( log (Total_width_mm)  ~   1   +  ( 1   |  Repeat),  data =  Length_HSHY)  
   test  =   anova (mod.gen, mod.gen1)  
   Chi2_LRT_growth  =   2  * (mod.gen $ APHLs[[ "p_v" ]] - mod.gen1 $ APHLs[[ "p_v" ]]) 
    
    #Now we make a tab with the results  
   tab_stat  =   data.frame ( Variable =   as.character ( paste ( "HS vs HY" )), 
                                   Rep =   nlevels (Length_HSHY $ Repeat), 
                                   chi2_LR =   round ( as.numeric (test $ basicLRT $ chi2_LR),  digits =   2 ), 
                                   intercept =   format (mod.gen $ fixef[ 1 ], digits=  3 ), 
                                   estimate =   format (mod.gen $ fixef[ 2 ], digits=  3 ), 
                                   df =   as.numeric (test $ basicLRT $ df), 
                                   Pvalue =   as.numeric ( format ( pchisq (Chi2_LRT_growth, df=  1 , lower.tail =  F), digits=  2 ))) 
   tab_stat $ sig  =   ifelse (tab_stat $ Pvalue  <   0.05   &  tab_stat $ Pvalue  >   0.01 ,  "*" , 
                 ifelse (tab_stat $ Pvalue  <   0.01   &  tab_stat $ Pvalue  >   0.001 ,  "**" , 
                  ifelse (tab_stat $ Pvalue  <   0.001 ,  "***" ,  "" ))) 
    
   tab_stat %>%  
      kable ( col.names =   c ( "Comparison" ,  "Replicates" ,  "Chi2" , "Intercept" , "Estimate" , "df"  , "p-value" , "Signif." ), row.names =   FALSE )  %>%  
      add_header_above ( c ( "log(Total_width_mm) ~  Diet + (1 | Repeat)"   =   8 )) %>%  
      kable_styling ( bootstrap_options =   c ( "striped" ,  "hover" ,  "condensed" ),  full_width =  F)    
 
 
 
 
 
log(Total_width_mm) ~ Diet + (1 | Repeat)
 
 
 
 
 
Comparison
 
 
Replicates
 
 
Chi2
 
 
Intercept
 
 
Estimate
 
 
df
 
 
p-value
 
 
Signif.
 
 
 
 
 
 
HS vs HY
 
 
3
 
 
55.48
 
 
-0.754
 
 
0.374
 
 
1
 
 
0
 
 
***
 
 
 
 
       ### Plot  
   Limits  =   c ( "HS" ,  "HY" ) 
   z  =   max (Length_HSHY $ Total_width_mm) 
    
   Plot_Fig1S1A =  
    ggplot (Length_HSHY,  aes ( x =  Diet,  y =  Total_width_mm)) +   
      geom_violin ( aes ( fill =  Diet),  draw_quantiles =   c ( 0.25 ,  0.5 ,  0.75 ),  colour =   "black" ,  size =   0.2 , adjust =   0.6 )  +  
      geom_dotplot (  colour =   "black" ,  fill =   "white" ,  binaxis =   "y" ,  stackdir =   "center" ,  binwidth =  z /  50 )  +   
      geom_text ( data =  Sample_size,  mapping =   aes ( x =  Diet,  y =   0.25 ,  label =   paste ( "(" ,Sample_size, ")" , sep=  "" )), size=  3 ) +  
      geom_signif ( annotation =   formatC ( paste ( "p=" ,tab_stat $ Pvalue),  digits =   2 ),  textsize =   3 ,  y_position =   0.93 ,  xmin =   1 ,  xmax =   2 ,  tip_length =   c ( 0.02 ,  0.02 ),  vjust =   -  0.2 ) +  
      scale_fill_manual ( limits= Limits, 
                        values= palette_diet_2) +  
      scale_x_discrete ( "" , 
                       limits=  c ( "HS" ,  "HY" ), 
                       breaks=  c ( "HS" ,  "HY" )) +  
      scale_y_continuous ( "Midgut width (mm)" , 
                         limits=  c ( 0.2 , 1 ), 
                         breaks=  seq ( 0.2 , 1 , by=  0.1 )) +  
      stat_summary ( fun =  mean,  geom =   "point" ,  size =   3 ,  shape =   18 , colour =   "black" , aes ( group= Repeat))  +  
      stat_summary ( fun =  mean,  geom =   "point" ,  size =   2 ,  shape =   18 , aes ( group= Repeat,  colour =  Repeat))  +  
      scale_color_manual ( values= palette_mean) +  
      theme ( panel.background =   element_blank (), 
            panel.grid.major.y =   element_line ( colour =   grey ( 0.45 ),  linetype =   "dashed" ,  size =   0.2 ), 
                    axis.title.x =   element_text ( size= Smallfont, colour=  "black" ), 
                    axis.title.y =   element_text ( size= Smallfont, colour=  "black" ),  
                    axis.line.x =   element_line ( colour=  "black" , size=  0.75 ), 
                    axis.line.y =   element_line ( colour=  "black" , size=  0.75 ), 
                    axis.ticks.x =   element_line ( size =   0.75 ), 
                    axis.ticks.y =   element_line ( size =   0.75 ), 
                    axis.text.x =   element_text ( size= Smallfont, colour=  "black" ), 
                    axis.text.y =   element_text ( size= Smallfont, colour=  "black" ), 
                    plot.margin =   unit (Margin,  "cm" ), 
                    legend.direction =   "vertical" ,  
                    legend.box =   "horizontal" , 
                    legend.position =   "none" , 
                    legend.key.height =   unit ( 0.4 ,  "cm" ), 
                    legend.key.width=   unit ( 0.6 ,  "cm" ), 
                    legend.title =   element_text ( face=  "italic" , size= Smallfont),  
                    legend.key =   element_rect ( colour =   'white' ,  fill =   "white" ,  linetype=  'dashed' ), 
                    legend.text =   element_text ( size= SuperSmallfont), 
                    legend.background =   element_rect ( fill=  NA )) 
    
   Plot_Fig1S1A    
   
 
 
  1.2.2  Figure 1S1B 
 
 Length of midguts on HY diet is similar to standard diets used in the field (Bloomington [Bl] cornmeal and Bl molasses). 
 
      tab_stddiets_rev  =  
     d[[ "1 - S1B" ]] %>%  
      mutate ( Total_Length_mm = Total.L /  1000 ) %>%  
      mutate_if (is.character,as.factor) %>%  
      mutate_if (is.integer,as.factor) %>%  
      mutate ( Sugar=  fct_relevel (Diet, "HS" , "HY" ,  "BL Cornmeal" ,  "BL Molasses" )) 
    
   Sample_size =  
     tab_stddiets_rev %>%  
      group_by (Diet) %>%  
      summarise ( Sample_size=  n ()) 
    
    ###Stats  
    
   mod.gen  =   fitme ((Total_Length_mm)  ~   Diet  +  ( 1   |  Repeat),  data =  tab_stddiets_rev) 
    shapiro.test ( residuals (mod.gen))     
  ## 
##  Shapiro-Wilk normality test
## 
## data:  residuals(mod.gen)
## W = 0.96479, p-value = 0.00686  
       bptest ((Total_Length_mm)  ~  Diet  +  ( 1   /  Repeat),  data =  tab_stddiets_rev)     
  ## 
##  studentized Breusch-Pagan test
## 
## data:  (Total_Length_mm) ~ Diet + (1/Repeat)
## BP = 1.7773, df = 3, p-value = 0.6199  
      mod.gen1  =   fitme ((Total_Length_mm)  ~   1   +  ( 1   |  Repeat),  data =  tab_stddiets_rev)  
   test  =   anova (mod.gen, mod.gen1)  
   Chi2_LRT_growth  =   2  * (mod.gen $ APHLs[[ "p_v" ]] - mod.gen1 $ APHLs[[ "p_v" ]]) 
    
    #Now we make a tab with the results  
   tab_stat  =   data.frame ( Variable =   as.character ( paste ( "Anova diets" )), 
                                   Rep =   nlevels (tab_stddiets_rev $ Repeat), 
                                   chi2_LR =   round ( as.numeric (test $ basicLRT $ chi2_LR),  digits =   2 ), 
                                   intercept =   format (mod.gen $ fixef[ 1 ], digits=  3 ), 
                                   estimate =   format (mod.gen $ fixef[ 2 ], digits=  3 ), 
                                   df =   as.numeric (test $ basicLRT $ df), 
                                   Pvalue =   as.numeric ( format ( pchisq (Chi2_LRT_growth, df=  1 , lower.tail =  F), digits=  2 ))) 
   tab_stat $ sig  =   ifelse (tab_stat $ Pvalue  <   0.05   &  tab_stat $ Pvalue  >   0.01 ,  "*" , 
                 ifelse (tab_stat $ Pvalue  <   0.01   &  tab_stat $ Pvalue  >   0.001 ,  "**" , 
                  ifelse (tab_stat $ Pvalue  <   0.001 ,  "***" ,  "" ))) 
    
   tab_stat %>%  
      kable ( col.names =   c ( "Comparison" ,  "Replicates" ,  "Chi2" , "Intercept" , "Estimate" , "df"  , "p-value" , "Signif." ), row.names =   FALSE )  %>%     add_header_above ( c ( "(Total_Length_mm) ~  Diet + (1 | Repeat)"   =   8 )) %>%  
      kable_styling ( bootstrap_options =   c ( "striped" ,  "hover" ,  "condensed" ),  full_width =  F)    
 
 
 
 
 
(Total_Length_mm) ~ Diet + (1 | Repeat)
 
 
 
 
 
Comparison
 
 
Replicates
 
 
Chi2
 
 
Intercept
 
 
Estimate
 
 
df
 
 
p-value
 
 
Signif.
 
 
 
 
 
 
Anova diets
 
 
3
 
 
53.44
 
 
5.07
 
 
0.435
 
 
3
 
 
0
 
 
***
 
 
 
 
      mod.gen  =   lmer (Total_Length_mm  ~   Diet  +  ( 1   |  Repeat),  data =  tab_stddiets_rev) 
   multcomp  =   glht (mod.gen,  linfct=  mcp ( Diet=  "Tukey" )) 
   tmp  =   cld (multcomp) 
    
   letter_position  =   aggregate ( data= tab_stddiets_rev,Total_Length_mm  ~  Diet, max) 
    
   tab_letter  =    as.data.frame (tmp $ mcletters $ Letters) 
   tab_letter $ Diet =  rownames (tab_letter) 
    colnames (tab_letter)[ 1 ]  =   "Letter"  
   tab_letter  =   left_join (tab_letter,letter_position) 
    
   Limits  =  c ( "HS" , "HY" ,  "BL Cornmeal" ,  "BL Molasses" ) 
   Labels  =  c ( "HS" , "HY" ,  "Bl cornmeal" ,  "Bl molasses" ) 
   cbbPalette  =   c ( "#FFB4B4" , "#C3E6FC" ,  "#f6efe5" ,  "#f6efe5" ) 
    
   z  =   max (tab_stddiets_rev $ Total_Length_mm,  na.rm =   TRUE ) 
    
   Plot_Fig1S1B =  
      ggplot (tab_stddiets_rev,  aes ( x =  Diet,  y =  Total_Length_mm)) +   
      geom_violin ( aes ( fill =  Diet),  draw_quantiles =   c ( 0.25 ,  0.5 ,  0.75 ),  colour =   "black" ,  size =   0.2 , adjust =   0.8 ,  alpha =   0.5 )  +  
      geom_dotplot (  colour =   "black" ,  fill =   "white" ,  binaxis =   "y" ,  stackdir =   "center" ,  binwidth =  z /  35 )  +   
      geom_text ( data =  Sample_size,  mapping =   aes ( x =  Diet,  y =   1.8 ,  label =   paste ( "(" ,Sample_size, ")" , sep=  "" )), size=  3 ) +  
      geom_text ( data =  tab_stat,  mapping =   aes ( x =   2.5 ,  y =   7.5 ,  label =   paste ( "p=" , format (Pvalue, digits=  2 ))), size=  3 ) +  
      geom_text ( data =  tab_letter,  mapping =   aes ( x =  Diet,  y =  Total_Length_mm +0.6 ,  label =  Letter), size=  3 ) +  
      scale_fill_manual ( limits= Limits, 
                        values= cbbPalette) +  
      scale_x_discrete ( "" , 
                       limits= Limits, 
                       labels= Labels) +  
      scale_y_continuous ( "Midgut length (mm)" , 
                         limits=  c ( 1.6 , 8 ), 
                         breaks=  seq ( 2 , 7 , by=  1 )) +  
       stat_summary ( fun =  mean,  geom =   "point" ,  size =   3 ,  shape =   18 ,  colour =   "black" ,  aes ( group =  Repeat))  +  
                         stat_summary ( fun =  mean,  geom =   "point" ,  size =   2 ,  shape =   18 ,  aes ( group =  Repeat,  colour =  Repeat))  +  
                         scale_color_manual ( values =  palette_mean)  +  
      theme ( panel.background =   element_blank (), 
            panel.grid.major.y =   element_line ( colour =   grey ( 0.45 ),  linetype =   "dashed" ,  size =   0.2 ), 
            axis.title.x =   element_text ( size= Smallfont, colour=  "black" ), 
            axis.title.y =   element_text ( size= Smallfont, colour=  "black" ),  
            axis.line.x =   element_line ( colour=  "black" , size=  0.75 ), 
            axis.line.y =   element_line ( colour=  "black" , size=  0.75 ), 
            axis.ticks.x =   element_line ( size =   0.75 ), 
            axis.ticks.y =   element_line ( size =   0.75 ), 
            axis.text.x =   element_text ( size= Smallfont, colour=  "black" ), 
            axis.text.y =   element_text ( size= Smallfont, colour=  "black" ), 
            plot.margin =   unit ( c ( 0 , 0 , 0 , 0.5 ),  "cm" ), 
            legend.direction =   "vertical" ,  
            legend.box =   "horizontal" , 
            legend.position =   "none" , 
            legend.key.height =   unit ( 0.4 ,  "cm" ), 
            legend.key.width=   unit ( 0.6 ,  "cm" ), 
            legend.title =   element_text ( face=  "italic" , size= Smallfont),  
            legend.key =   element_rect ( colour =   'white' ,  fill =   "white" ,  linetype=  'dashed' ), 
            legend.text =   element_text ( size= SuperSmallfont), 
            legend.background =   element_rect ( fill=  NA )) 
    
   Plot_Fig1S1B    
   
 
 
  1.2.3  Figure 1S1C 
 
  Un-mated females and mated males have lower response to diet compared to mated female flies.  Statistics: comparison of the interaction between diet and mating status/sex.  Full annotation on figure present in manuscript  
 
      tab_MUmM_rev  =   
     d[[ "1 - S1C" ]] %>%  
      mutate_at ( vars ( !  starts_with ( "Total" )),as.factor) %>%  
      mutate ( Total_Length_mm= Total.L /  1000 ) %>%  
      mutate ( Treatment=  fct_relevel (Treatment, c ( "Mated females" , "Un-mated females" , "Males" ))) 
    
   Sample_size =  
     tab_MUmM_rev %>%  
      group_by (Diet,Treatment) %>%  
      summarise ( Sample_size=  n ()) 
    
    ###Stats  
    #Un-mated  
   tmp  =   subset (tab_MUmM_rev, Treatment %in%  c ( "Mated females" , "Un-mated females" )) 
   mod.gen  =   fitme ((Total_Length_mm)  ~   Diet  *  Treatment  +  ( 1   |  Repeat), data =  tmp) 
    shapiro.test ( residuals (mod.gen))     
  ## 
##  Shapiro-Wilk normality test
## 
## data:  residuals(mod.gen)
## W = 0.98312, p-value = 0.2426  
       bptest ((Total_Length_mm)  ~   Diet  +  Treatment  +  ( 1   /  Repeat), data =  tmp)    
  ## 
##  studentized Breusch-Pagan test
## 
## data:  (Total_Length_mm) ~ Diet + Treatment + (1/Repeat)
## BP = 0.27838, df = 2, p-value = 0.8701  
      mod.gen1  =   fitme ((Total_Length_mm)  ~   Diet  +  Treatment  +  ( 1   |  Repeat), data =  tmp) 
   test  =   anova (mod.gen, mod.gen1)  
   Chi2_LRT_growth  =   2  * (mod.gen $ APHLs[[ "p_v" ]] - mod.gen1 $ APHLs[[ "p_v" ]]) 
    
   tab_stat  =   data.frame ( Treatment =   as.character ( paste ( "Mated vs Un-mated females" )), 
                                   Rep =   nlevels (tmp $ Repeat), 
                                   chi2_LR =   round ( as.numeric (test $ basicLRT $ chi2_LR),  digits =   2 ), 
                                   intercept =   format (mod.gen $ fixef[ 1 ], digits=  3 ), 
                                   estimate =   format (mod.gen $ fixef[ 2 ], digits=  3 ), 
                                   df =   as.numeric (test $ basicLRT $ df), 
                                   Pvalue =   as.numeric ( format ( pchisq (Chi2_LRT_growth, df=  1 , lower.tail =  F), digits=  1 , scientific= F))) 
   tab_stat_Un_mated = tab_stat 
    
    
    
    
    
    #Male  
   tmp  =   subset (tab_MUmM_rev, Treatment %in%  c ( "Mated females" , "Males" )) 
   mod.gen  =   fitme ( log (Total_Length_mm)  ~   Diet  *  Treatment  +  ( 1   |  Repeat), data =  tmp) 
    shapiro.test ( residuals (mod.gen))     
  ## 
##  Shapiro-Wilk normality test
## 
## data:  residuals(mod.gen)
## W = 0.97901, p-value = 0.115  
       bptest ( log (Total_Length_mm)  ~   Diet  +  Treatment  +  ( 1   /  Repeat), data =  tmp)    
  ## 
##  studentized Breusch-Pagan test
## 
## data:  log(Total_Length_mm) ~ Diet + Treatment + (1/Repeat)
## BP = 6.2807, df = 2, p-value = 0.04327  
      mod.gen1  =   fitme ( log (Total_Length_mm)  ~   Diet  +  Treatment  +  ( 1   |  Repeat), data =  tmp) 
   test  =   anova (mod.gen, mod.gen1)  
   Chi2_LRT_growth  =   2  * (mod.gen $ APHLs[[ "p_v" ]] - mod.gen1 $ APHLs[[ "p_v" ]]) 
    
   tab_stat  =   data.frame ( Treatment =   as.character ( paste ( "Mated vs Males" )), 
                                   Rep =   nlevels (tmp $ Repeat), 
                                   chi2_LR =   round ( as.numeric (test $ basicLRT $ chi2_LR),  digits =   2 ), 
                                   intercept =   format (mod.gen $ fixef[ 1 ], digits=  3 ), 
                                   estimate =   format (mod.gen $ fixef[ 2 ], digits=  3 ), 
                                   df =   as.numeric (test $ basicLRT $ df), 
                                   Pvalue =   as.numeric ( format ( pchisq (Chi2_LRT_growth, df=  1 , lower.tail =  F), digits=  1 , scientific= F))) 
   tab_stat_Male = tab_stat 
    
   tab_stat =  rbind (tab_stat_Male,tab_stat_Un_mated) 
   tab_stat $ sig  =   ifelse (tab_stat $ Pvalue  <   0.05   &  tab_stat $ Pvalue  >   0.009 ,  "*" ,  #changed to 0.009 because Un-mated is exactle 0.01  
                 ifelse (tab_stat $ Pvalue  <   0.01   &  tab_stat $ Pvalue  >   0.001 ,  "**" , 
                  ifelse (tab_stat $ Pvalue  <   0.001 ,  "***" ,  "" ))) 
    
    
    
   tab_stat %>%  
      kable ( col.names =   c ( "Variable" ,  "Replicates" ,  "Chi2" , "Intercept" , "Estimate" , "df"  , "p-value" , "Signif." ), row.names =   FALSE )  %>%  
      add_header_above ( c ( "log(Total_Length_mm) ~  Diet + Treatment + Diet : Treatment + (1 | Repeat)"   =   8 )) %>%  
      kable_styling ( bootstrap_options =   c ( "striped" ,  "hover" ,  "condensed" ),  full_width =  F)    
 
 
 
 
 
log(Total_Length_mm) ~ Diet + Treatment + Diet : Treatment + (1 | Repeat)
 
 
 
 
 
Variable
 
 
Replicates
 
 
Chi2
 
 
Intercept
 
 
Estimate
 
 
df
 
 
p-value
 
 
Signif.
 
 
 
 
 
 
Mated vs Males
 
 
3
 
 
23.35
 
 
1.46
 
 
0.229
 
 
1
 
 
1e-06
 
 
***
 
 
 
 
Mated vs Un-mated females
 
 
3
 
 
6.57
 
 
4.35
 
 
1.11
 
 
1
 
 
1e-02
 
 
 
  
 
 
  
 
      tab_stat $ Treatment  =   as.factor (tab_stat $ Treatment) 
   tab    
  ## Error in eval(expr, envir, enclos): object 'tab' not found  
       ### Plot  
   tab_stat_1S1C = tab_stat 
    
   Plot_Fig1S1C =  
      ggplot (tab_MUmM_rev,  aes ( x =  Diet,  y =  Total_Length_mm)) +   
      geom_violin ( aes ( fill =  Diet),  draw_quantiles =   c ( 0.25 ,  0.5 ,  0.75 ),  colour =   "black" ,  size =   0.2 , adjust =   0.8 )  +  
      geom_dotplot (  colour =   "black" ,  fill =   "white" ,  binaxis =   "y" ,  stackdir =   "center" ,  binwidth =   0.15 )  +   
      geom_text ( data =  Sample_size,  mapping =   aes ( x =  Diet,  y =   1.7 ,  label =   paste ( "(" ,Sample_size, ")" , sep=  "" )), size=  3 ) +  
      facet_grid (. ~  Treatment) +  
      scale_fill_manual ( values= palette_diet_2) +  
      scale_x_discrete ( "" , 
                       limits=  c ( "HS" , "HY" ), 
                       labels=  c ( "HS" , "HY" )) +  
      scale_y_continuous ( "Midgut length (mm)" , 
                         limits=  c ( 1.5 , 8 ), 
                         breaks=  seq ( 2 , 6 , by=  1 )) +  
      stat_summary ( fun =  mean,  geom =   "point" ,  size =   3 ,  shape =   18 ,  colour =   "black" ,  aes ( group =  Repeat))  +  
      stat_summary ( fun =  mean,  geom =   "point" ,  size =   2 ,  shape =   18 ,  aes ( group =  Repeat,  colour =  Repeat))  +  
      stat_summary ( fun =  mean,  colour =   "black" ,  geom =   "line" ,  aes ( group =  Repeat))  +  
      scale_color_manual ( values =  palette_mean)  +  
      theme ( panel.grid.major.y =   element_line ( colour =   grey ( 0.45 ),  linetype =   "dashed" ,  size =   0.2 ), 
            panel.background =   element_blank (), 
            axis.title.x =   element_text ( size= Smallfont, colour=  "black" ), 
            axis.title.y =   element_text ( size= Smallfont, colour=  "black" ),  
            axis.line.x =   element_line ( colour=  "black" , size=  0.75 ), 
            axis.line.y =   element_line ( colour=  "black" , size=  0.75 ), 
            axis.ticks.x =   element_line ( size =   0.75 ), 
            axis.ticks.y =   element_line ( size =   0.75 ), 
            axis.text.x =   element_text ( size= Smallfont, colour=  "black" ), 
            axis.text.y =   element_text ( size= Smallfont, colour=  "black" ), 
            plot.margin =   unit (Margin,  "cm" ), 
            legend.direction =   "vertical" ,  
            legend.box =   "horizontal" , 
            legend.position =   "none" , 
            legend.key.height =   unit ( 0.4 ,  "cm" ), 
            legend.key.width=   unit ( 0.6 ,  "cm" ), 
            legend.title =   element_text ( face=  "italic" , size= Smallfont),  
            legend.key =   element_rect ( colour =   'white' ,  fill =   "white" ,  linetype=  'dashed' ), 
            legend.text =   element_text ( size= SuperSmallfont), 
            legend.background =   element_rect ( fill=  NA ), 
            strip.text.x =   element_text ( size =  Smallfont,  colour =   "black" ,  margin =   margin ( t =   2 ,  r =   0 ,  b =   2 ,  l =   0 )), 
            strip.text.y =   element_text ( size =  Smallfont,  colour =   "black" ,  margin =   margin ( t =   2 ,  r =   0 ,  b =   2 ,  l =   0 )), 
            strip.background =   element_rect ( fill=  NA ,  colour=  "black" ), 
            strip.placement=  "outside" ) 
    
   Plot_Fig1S1C    
   
 
 
  1.2.4  Figure 1S1D 
 
  Feeding assay shows higher dietary intake on HS than on HY diet.  Absorbance measured after 1 day of assay, each day along a 5-day period from eclosion, for a total of 5 times per condition/repeat 
 
      Tab_absorbance  =   
     d[[ "1 - S1D" ]] %>%  
      mutate_if (is.character,as.factor) %>%  
      mutate_if (is.integer,as.factor) %>%  
     dplyr ::  rename ( Diet= Food) 
    
   Sample_size =  
     Tab_absorbance %>%  
      group_by (Diet) %>%  
      summarise ( Sample_size=  n ()) 
    
    ###Stats  
    
   mod.gen  =   fitme (Absorbance  ~   Diet  +  ( 1   |  Repeat),  data =  Tab_absorbance) 
    shapiro.test ( residuals (mod.gen))     
  ## 
##  Shapiro-Wilk normality test
## 
## data:  residuals(mod.gen)
## W = 0.91089, p-value = 0.01567  
       bptest (Absorbance  ~  Diet  +  ( 1   /  Repeat),  data =  Tab_absorbance)     
  ## 
##  studentized Breusch-Pagan test
## 
## data:  Absorbance ~ Diet + (1/Repeat)
## BP = 5.6989, df = 1, p-value = 0.01698  
      mod.gen1  =   fitme (Absorbance  ~   1   +  ( 1   |  Repeat),  data =  Tab_absorbance)  
   test  =   anova (mod.gen, mod.gen1)  
   Chi2_LRT_growth  =   2  * (mod.gen $ APHLs[[ "p_v" ]] - mod.gen1 $ APHLs[[ "p_v" ]]) 
    
    #Now we make a tab with the results  
   tab_stat  =   data.frame ( Variable =   as.character ( paste ( "HS vs HY" )), 
                                   Rep =   nlevels (Tab_absorbance $ Repeat), 
                                   chi2_LR =   round ( as.numeric (test $ basicLRT $ chi2_LR),  digits =   2 ), 
                                   intercept =   format (mod.gen $ fixef[ 1 ], digits=  3 ), 
                                   estimate =   format (mod.gen $ fixef[ 2 ], digits=  3 ), 
                                   df =   as.numeric (test $ basicLRT $ df), 
                                   Pvalue =   as.numeric ( format ( pchisq (Chi2_LRT_growth, df=  1 , lower.tail =  F), digits=  2 ))) 
   tab_stat $ sig  =   ifelse (tab_stat $ Pvalue  <   0.05   &  tab_stat $ Pvalue  >   0.01 ,  "*" , 
                 ifelse (tab_stat $ Pvalue  <   0.01   &  tab_stat $ Pvalue  >   0.001 ,  "**" , 
                  ifelse (tab_stat $ Pvalue  <   0.001 ,  "***" ,  "" ))) 
    
   tab_stat %>%  
      kable ( col.names =   c ( "Comparison" ,  "Replicates" ,  "Chi2" , "Intercept" , "Estimate" , "df"  , "p-value" , "Signif." ), row.names =   FALSE )  %>%     add_header_above ( c ( "Absorbance ~  Diet + (1 | Repeat)"   =   8 )) %>%  
      kable_styling ( bootstrap_options =   c ( "striped" ,  "hover" ,  "condensed" ),  full_width =  F)    
 
 
 
 
 
Absorbance ~ Diet + (1 | Repeat)
 
 
 
 
 
Comparison
 
 
Replicates
 
 
Chi2
 
 
Intercept
 
 
Estimate
 
 
df
 
 
p-value
 
 
Signif.
 
 
 
 
 
 
HS vs HY
 
 
3
 
 
30.71
 
 
0.404
 
 
-0.256
 
 
1
 
 
0
 
 
***
 
 
 
 
       ##Plot  
   Limits  =   c ( "HS" ,  "HY" ) 
   z  =   max (Tab_absorbance $ Absorbance) 
    
   Plot_Fig1S1D =  
    ggplot (Tab_absorbance,  aes ( x =  Diet,  y =  Absorbance)) +   
      geom_violin ( aes ( fill =  Diet),  draw_quantiles =   c ( 0.25 ,  0.5 ,  0.75 ),  colour =   "black" ,  size =   0.2 , adjust =   0.8 )  +  
      geom_dotplot (  colour =   "black" ,  fill =   "white" ,  binaxis =   "y" ,  stackdir =   "center" ,  binwidth =  z /  40 )  +   
      geom_text ( data =  Sample_size,  mapping =   aes ( x =  Diet,  y =   0.02 ,  label =   paste ( "(" ,Sample_size, ")" , sep=  "" )), size=  3 ) +  
      geom_signif ( annotation =   formatC ( paste ( "p=" ,tab_stat $ Pvalue),  digits =   2 ),  textsize =   3 ,  y_position =   0.58 ,  xmin =   1 ,  xmax =   2 ,  tip_length =   c ( 0.02 ,  0.02 ),  vjust =   -  0.2 ) +  
    
      scale_fill_manual ( limits= Limits, 
                        values= palette_diet_2) +  
      scale_x_discrete ( "" , 
                       limits=  c ( "HS" ,  "HY" ), 
                       breaks=  c ( "HS" ,  "HY" )) +  
      scale_y_continuous ( "Absorbance" , 
                         limits=  c ( 0 , 0.6 ), 
                         breaks=  seq ( 0 , 0.5 , by=  0.1 )) +  
      stat_summary ( fun =  mean,  geom =   "point" ,  size =   3 ,  shape =   18 , colour =   "black" , aes ( group= Repeat))  +  
      stat_summary ( fun =  mean,  geom =   "point" ,  size =   2 ,  shape =   18 , aes ( group= Repeat,  colour =  Repeat))  +  
      scale_color_manual ( values= palette_mean) +  
      theme ( panel.background =   element_blank (), 
            panel.grid.major.y =   element_line ( colour =   grey ( 0.45 ),  linetype =   "dashed" ,  size =   0.2 ), 
                    axis.title.x =   element_text ( size= Smallfont, colour=  "black" ), 
                    axis.title.y =   element_text ( size= Smallfont, colour=  "black" ),  
                    axis.line.x =   element_line ( colour=  "black" , size=  0.75 ), 
                    axis.line.y =   element_line ( colour=  "black" , size=  0.75 ), 
                    axis.ticks.x =   element_line ( size =   0.75 ), 
                    axis.ticks.y =   element_line ( size =   0.75 ), 
                    axis.text.x =   element_text ( size= Smallfont, colour=  "black" ), 
                    axis.text.y =   element_text ( size= Smallfont, colour=  "black" ), 
                    plot.margin =   unit (Margin,  "cm" ), 
                    legend.direction =   "vertical" ,  
                    legend.box =   "horizontal" , 
                    legend.position =   "none" , 
                    legend.key.height =   unit ( 0.4 ,  "cm" ), 
                    legend.key.width=   unit ( 0.6 ,  "cm" ), 
                    legend.title =   element_text ( face=  "italic" , size= Smallfont),  
                    legend.key =   element_rect ( colour =   'white' ,  fill =   "white" ,  linetype=  'dashed' ), 
                    legend.text =   element_text ( size= SuperSmallfont), 
                    legend.background =   element_rect ( fill=  NA )) 
    
   Plot_Fig1S1D    
   
 
 
  1.2.5  Figure 1S1E 
 
  Microbes are not required for the difference in size observed between HS and HY fed flies.  Germ-free flies exhibit similar diet-induced increase in size as conventionally reared flies at both 7- and 14-days post eclosion (statistics: comparison of the interaction between diets and conv. reared/germ free treatment). Of note, at 7-days post eclosion we observed longer guts in germ free flies compared to conventionally reared flies (significant on HS diet). This difference was lost at 14-days post eclosion (Post hoc Tukey test from GLMM summarized by letter at the bottom of chart).  Full statistical annotation on figure present in manuscript  
 
      Length_HSHY_germfree  =   
     d[[ "1 - S1E" ]] %>%  
      mutate_at ( vars ( starts_with ( "Total" )), ~ . /  1000 ) %>%  
      mutate_if (is.character,as.factor) %>%  
      mutate_if (is.integer,as.factor) %>%  
     dplyr ::  rename ( Total_Length_mm= Total.L, 
             Day_of_treatment= Day) 
    
    
   Sample_size =  
     Length_HSHY_germfree %>%  
      group_by (Diet,Treatment, Day_of_treatment) %>%  
      summarise ( Sample_size=  n ()) 
    
   Sample_size $ Sample_size  <-   as.numeric (Sample_size $ Sample_size) 
    
    
    
    
    
    ###Stats Day 7 interaction  
   Length_HSHY_germfree_Day7  =   subset (Length_HSHY_germfree, Day_of_treatment  ==   "7" ) 
   mod.gen  =   fitme ( log (Total_Length_mm)  ~   Diet  +  Treatment  +  Diet  :  Treatment  +  ( 1   |  Repeat),  data =  Length_HSHY_germfree_Day7) 
    shapiro.test ( residuals (mod.gen))     
  ## 
##  Shapiro-Wilk normality test
## 
## data:  residuals(mod.gen)
## W = 0.98828, p-value = 0.5513  
       bptest ( log (Total_Length_mm)  ~  Diet   +  Treatment  +   ( 1   /  Repeat),  data =  Length_HSHY_germfree_Day7)     
  ## 
##  studentized Breusch-Pagan test
## 
## data:  log(Total_Length_mm) ~ Diet + Treatment + (1/Repeat)
## BP = 5.3166, df = 2, p-value = 0.07007  
      mod.gen1  =   fitme ( log (Total_Length_mm)  ~  Diet  +  Treatment  +  ( 1   |  Repeat),  data =  Length_HSHY_germfree_Day7)  
   test  =   anova (mod.gen, mod.gen1)  
   Chi2_LRT_growth  =   2  * (mod.gen $ APHLs[[ "p_v" ]] - mod.gen1 $ APHLs[[ "p_v" ]]) 
    
    
    
    
    #Now we make a tab with the results  
   tab_stat7  =   data.frame ( Variable =   as.character ( paste ( "Response to diet Day7" )), 
                                   Rep =   as.numeric ( nlevels (Length_HSHY_germfree_Day7 $ Repeat)), 
                                   chi2_LR =   round ( as.numeric (test $ basicLRT $ chi2_LR),  digits =   2 ), 
                                   intercept =   format (mod.gen $ fixef[ 1 ], digits=  3 ), 
                                   estimate =   format (mod.gen $ fixef[ 4 ], digits=  3 ), 
                                   df =   as.numeric (test $ basicLRT $ df), 
                                   Pvalue =   as.numeric ( format ( pchisq (Chi2_LRT_growth, df=  1 , lower.tail =  F), digits=  2 ))) 
   tab_stat7 $ sig  =   ifelse (tab_stat7 $ Pvalue  <   0.05   &  tab_stat7 $ Pvalue  >   0.01 ,  "*" , 
                 ifelse (tab_stat7 $ Pvalue  <   0.01   &  tab_stat7 $ Pvalue  >   0.001 ,  "**" , 
                  ifelse (tab_stat7 $ Pvalue  <   0.001 ,  "***" ,  "" ))) 
    
   tab_stat7 %>%  
      kable ( col.names =   c ( "Response to diet Day7" ,  "Replicates" ,  "Chi2" , "Intercept" , "Estimate" , "df"  , "p-value" , "Signif." ), row.names =   FALSE )  %>%     add_header_above ( c ( "log(Total_Length_mm) ~  Diet + Treatment + Diet : Treatment + (1 | Repeat)"   =   8 )) %>%  
      kable_styling ( bootstrap_options =   c ( "striped" ,  "hover" ,  "condensed" ),  full_width =  F)    
 
 
 
 
 
log(Total_Length_mm) ~ Diet + Treatment + Diet : Treatment + (1 | Repeat)
 
 
 
 
 
Response to diet Day7
 
 
Replicates
 
 
Chi2
 
 
Intercept
 
 
Estimate
 
 
df
 
 
p-value
 
 
Signif.
 
 
 
 
 
 
Response to diet Day7
 
 
3
 
 
0.15
 
 
1.35
 
 
-0.015
 
 
1
 
 
0.7
 
 
 
 
 
 
      tab_stat_int_GF7 = tab_stat7 
    
    
    
    ###Stats Day 14 interaction  
   Length_HSHY_germfree_Day14  =   subset (Length_HSHY_germfree, Day_of_treatment  ==   "14" ) 
   mod.gen  =   fitme ( log (Total_Length_mm)  ~   Diet  +  Treatment  +  Diet  :  Treatment  +  ( 1   |  Repeat),  data =  Length_HSHY_germfree_Day14) 
    shapiro.test ( residuals (mod.gen))     
  ## 
##  Shapiro-Wilk normality test
## 
## data:  residuals(mod.gen)
## W = 0.98625, p-value = 0.4111  
       bptest ( log (Total_Length_mm)  ~  Diet   +  Treatment  +   ( 1   /  Repeat),  data =  Length_HSHY_germfree_Day14)     
  ## 
##  studentized Breusch-Pagan test
## 
## data:  log(Total_Length_mm) ~ Diet + Treatment + (1/Repeat)
## BP = 0.62752, df = 2, p-value = 0.7307  
      mod.gen1  =   fitme ( log (Total_Length_mm)  ~  Diet  +  Treatment  +  ( 1   |  Repeat),  data =  Length_HSHY_germfree_Day14)  
   test  =   anova (mod.gen, mod.gen1)  
   Chi2_LRT_growth  =   2  * (mod.gen $ APHLs[[ "p_v" ]] - mod.gen1 $ APHLs[[ "p_v" ]]) 
    
    
    
    
    #Now we make a tab with the results  
   tab_stat14  =   data.frame ( Variable =   as.character ( paste ( "Response to diet Day14" )), 
                                   Rep =   as.numeric ( nlevels (Length_HSHY_germfree_Day14 $ Repeat)), 
                                   chi2_LR =   round ( as.numeric (test $ basicLRT $ chi2_LR),  digits =   2 ), 
                                   intercept =   format (mod.gen $ fixef[ 1 ], digits=  3 ), 
                                   estimate =   format (mod.gen $ fixef[ 4 ], digits=  3 ), 
                                   df =   as.numeric (test $ basicLRT $ df), 
                                   Pvalue =   as.numeric ( format ( pchisq (Chi2_LRT_growth, df=  1 , lower.tail =  F), digits=  2 ))) 
   tab_stat14 $ sig  =   ifelse (tab_stat14 $ Pvalue  <   0.05   &  tab_stat14 $ Pvalue  >   0.01 ,  "*" , 
                 ifelse (tab_stat14 $ Pvalue  <   0.01   &  tab_stat14 $ Pvalue  >   0.001 ,  "**" , 
                  ifelse (tab_stat14 $ Pvalue  <   0.001 ,  "***" ,  "" ))) 
    
   tab_stat14 %>%  
      kable ( col.names =   c ( "Response to diet Day14" ,  "Replicates" ,  "Chi2" , "Intercept" , "Estimate" , "df"  , "p-value" , "Signif." ), row.names =   FALSE )  %>%     add_header_above ( c ( "log(Total_Length_mm) ~  Diet + Treatment + Diet : Treatment + (1 | Repeat)"   =   8 )) %>%  
      kable_styling ( bootstrap_options =   c ( "striped" ,  "hover" ,  "condensed" ),  full_width =  F)    
 
 
 
 
 
log(Total_Length_mm) ~ Diet + Treatment + Diet : Treatment + (1 | Repeat)
 
 
 
 
 
Response to diet Day14
 
 
Replicates
 
 
Chi2
 
 
Intercept
 
 
Estimate
 
 
df
 
 
p-value
 
 
Signif.
 
 
 
 
 
 
Response to diet Day14
 
 
3
 
 
3.75
 
 
1.29
 
 
-0.0976
 
 
1
 
 
0.053
 
 
 
 
 
 
      tab_stat_int_GF14 = tab_stat14 
    
    
    #Model including all samples and Post HOC test  
    
   Length_HSHY_germfree $ Treat_Diet_Day  =   as.factor ( paste (Length_HSHY_germfree $ Treatment, Length_HSHY_germfree $ Diet, Length_HSHY_germfree $ Day_of_treatment,  sep=  "_" )) 
    
    
    
    
   mod.gen  =   lmer ( log (Total_Length_mm)  ~  Treat_Diet_Day  +  ( 1   |  Repeat),  data =  Length_HSHY_germfree) 
    
    shapiro.test ( residuals (mod.gen))    
  ## 
##  Shapiro-Wilk normality test
## 
## data:  residuals(mod.gen)
## W = 0.99294, p-value = 0.4774  
       bptest ( log (Total_Length_mm)  ~  Treat_Diet_Day  +  ( 1  /  Repeat),  data =  Length_HSHY_germfree)    
  ## 
##  studentized Breusch-Pagan test
## 
## data:  log(Total_Length_mm) ~ Treat_Diet_Day + (1/Repeat)
## BP = 16.062, df = 7, p-value = 0.02455  
      multcomp  =   glht (mod.gen,  linfct=  mcp ( Treat_Diet_Day=  "Tukey" )) 
   tmp  =   cld (multcomp) 
    
    
    
   letter_position  =   aggregate ( data= Length_HSHY_germfree,Total_Length_mm  ~  Treat_Diet_Day, min) 
    
   tab_letter  =    as.data.frame (tmp $ mcletters $ Letters) 
   tab_letter $ Treat_Diet_Day =  rownames (tab_letter) 
    colnames (tab_letter)[ 1 ]  =   "Letter"  
   tab_letter  =   left_join (tab_letter,letter_position) 
   tab_letter $ Treat_Diet_Day =   as.factor (tab_letter $ Treat_Diet_Day) 
   tab_letter $ Day_of_treatment  =   as.factor ( mid (tab_letter $ Treat_Diet_Day, 7 ,  2 )) 
   tab_letter $ Diet  =   as.factor ( mid (tab_letter $ Treat_Diet_Day,  4 , 2 )) 
   tab_letter $ Treatment  =   as.factor ( left (tab_letter $ Treat_Diet_Day,  2 )) 
    
    
    
    ### Plot  
   Limits  =   c ( "HS" ,  "HY" ) 
   z =   max (Length_HSHY_germfree $ Total_Length_mm) 
   Treatment.status  =   c ( "Conv. reared" ,  "Germ free" ) 
    names (Treatment.status)  =   c ( "CR" ,  "GF" ) 
    
   Plot_Fig1S1E =  
      ggplot (Length_HSHY_germfree,  aes ( x =  Diet,  y =  Total_Length_mm)) +   
      geom_violin ( aes ( fill =  Diet),  draw_quantiles =   c ( 0.25 ,  0.5 ,  0.75 ),  colour =   "black" ,  size =   0.2 , adjust =   0.8 )  +  
      geom_dotplot (  colour =   "black" ,  fill =   "white" ,  binaxis =   "y" ,  stackdir =   "center" ,  binwidth =  z /  50 )  +  
      geom_text ( data =  tab_letter,  mapping =   aes ( x =  Diet,  y =  Total_Length_mm -0.4 ,  label =  Letter), size=  3 ) +  
      facet_wrap (Day_of_treatment ~ Treatment, labeller=  labeller ( Treatment= Treatment.status),  nrow =   1 ) +  
      geom_text ( data =  Sample_size,  mapping =   aes ( x =  Diet,  y =   1.35 ,  label =   paste ( "(" ,Sample_size, ")" , sep=  "" )), size=  3 ) +  
      scale_fill_manual ( limits= Limits, 
                        values= palette_diet_2) +  
      scale_x_discrete ( "" , 
                       limits=  c ( "HS" ,  "HY" ), 
                       breaks=  c ( "HS" ,  "HY" )) +  
      scale_y_continuous ( "Midgut length (mm)" , 
                         limits=  c ( 1 , 8 ), 
                         breaks=  seq ( 2 , 8 , by=  1 )) +  
      stat_summary ( fun =  mean,  geom =   "point" ,  size =   3 ,  shape =   18 , colour =   "black" , aes ( group= Repeat))  +  
      stat_summary ( fun =  mean,  geom =   "point" ,  size =   2 ,  shape =   18 , aes ( group= Repeat,  colour =  Repeat))  +  
      stat_summary ( fun= mean,  colour=  "black" ,  geom=  "line" , aes ( group= Repeat)) +  
      scale_color_manual ( values= palette_mean) +  
      theme ( panel.background =   element_blank (), 
            panel.grid.major.y =   element_line ( colour =   grey ( 0.45 ),  linetype =   "dashed" ,  size =   0.2 ), 
            axis.title.x =   element_text ( size= Smallfont, colour=  "black" ), 
            axis.title.y =   element_text ( size= Smallfont, colour=  "black" ),  
            axis.line.x =   element_line ( colour=  "black" , size=  0.75 ), 
            axis.line.y =   element_line ( colour=  "black" , size=  0.75 ), 
            axis.ticks.x =   element_line ( size =   0.75 ), 
            axis.ticks.y =   element_line ( size =   0.75 ), 
            axis.text.x =   element_text ( size= Smallfont, colour=  "black" ), 
            axis.text.y =   element_text ( size= Smallfont, colour=  "black" ), 
            plot.margin =   unit (Margin,  "cm" ), 
            legend.direction =   "vertical" ,  
            legend.box =   "horizontal" , 
            legend.position =   "none" , 
            legend.key.height =   unit ( 0.4 ,  "cm" ), 
            legend.key.width=   unit ( 0.6 ,  "cm" ), 
            legend.title =   element_text ( face=  "italic" , size= Smallfont),  
            legend.key =   element_rect ( colour =   'white' ,  fill =   "white" ,  linetype=  'dashed' ), 
            legend.text =   element_text ( size= SuperSmallfont), 
            legend.background =   element_rect ( fill=  NA ), 
            strip.text.x =   element_text ( size = Smallfont,  colour =   "black" ,  margin =   margin ( t =   2 ,  r =   0 ,  b =   2 ,  l =   0 )), 
            strip.text.y =   element_text ( size = Smallfont,  colour =   "black" ,  margin =   margin ( t =   2 ,  r =   0 ,  b =   2 ,  l =   0 )), 
            strip.background =   element_rect ( fill=  NA ,  colour=  "black" ), 
            strip.placement=  "outside" ) 
   Plot_Fig1S1E    
   
 ##Export Figure S1 
 
 
 
  1.3  Figure 1 - figure supplement 2 
 
  1.3.1  Figure 1S2A 
 
  Scheme depicting regional organization of the gut.  The gut comprises three main anatomical regions: foregut (comprising the crop), midgut and hindgut. The midgut itself can be divided in anterior (blue), middle (green) and posterior (purple). Additional subregions have been described (Buchon et al., 2013; Marianes and Spradling, 2013). 
 
       img1S2A  =   readImage ( "D:/Dropbox/z_ Ale Shared work/z_Nutrition Paper Markdown/Ale/Revision/1- S2A.jpg" )  
    gob_imageFig1S2A  =   rasterGrob (img1S2A) 
     grid.draw (gob_imageFig1S2A)    
   
 
 
  1.3.2  Figure 1S2B 
 
 All regions of the midgut (x-axis) respond variably to diet composition, but the response of the posterior midgut length more closely reflects the response of the total midgut length. Red lines represent linear regression and black dashed lines are the lines of equivalence. 
 
      tab_GWAS_gut $ dgrpDiet  =   factor ( paste (tab_GWAS_gut $ dgrp_id, tab_GWAS_gut $ diet),  ordered= T) 
    
   meansMat  =   aggregate ( as.matrix (tab_GWAS_gut[, c ( "anteriorlength" ,  "middlelength" ,  "posteriorlength" ,   "totallength" )])  ~  diet  *  dgrp_id, tab_GWAS_gut, mean) 
    rownames (meansMat)  =   paste (meansMat $ dgrp_id, meansMat $ diet,  sep=  "_" ) 
    colnames (meansMat)  <-   c ( "diet" , "dgrp_id" ,  "Anterior Length" ,  "Middle Length" ,  "Posterior Length" ,  "Total Length" ) 
   meansMatX  =   subset (meansMat, diet ==  "x" ) 
   meansMatY  =   subset (meansMat, diet ==  "y" ) 
    all (meansMatX $ dgrp_id  ==  meansMatY $ dgrp_id) 
   meansMatX  =  meansMatX[, !  colnames (meansMatX)  %in%   c ( "dgrp_id" ,  "diet" )] 
   meansMatY  =  meansMatY[, !  colnames (meansMatY)  %in%   c ( "dgrp_id" ,  "diet" )] 
    
   meansMatY =  
   meansMatY  %>%  
      setNames ( str_to_sentence ( names (.))) 
    
   meansMatX =  
   meansMatX  %>%  
      setNames ( str_to_sentence ( names (.))) 
    
   RIs  <-  meansMatY  /  meansMatX 
   RIs  <-  RIs[, !  grepl ( "width" ,  colnames (RIs))] 
   plotRegress  <-   function (x,y, datRange, textCex, ...){ 
       regn  <-   lm (y  ~  x) 
        plot (y  ~  x,  xlim= datRange,  ylim= datRange, ...) 
        abline ( a=  0 , b=  1 ,  col=  alpha ( 1 ,  0.5 ),  lty=  2 ) 
        abline ( a=  coef (regn)[ 1 ],  b=  coef (regn)[ 2 ], col=  2 ) 
        
       val =  round ( summary (regn) $ adj.r.squared,  1 ) 
        text ( y=  max (datRange),  x=  min (datRange),  
        labels=  bquote (R ^  2   ~  "="  ~  .(val)),  adj=  0 ,  cex= textCex) 
        
        text ( y=  max (datRange)  -  ( 0.1   *   diff ( range (RIs))),  x=  min (datRange),  
        labels=  paste ( "p =" ,  signif ( summary (regn) $ coefficients[ 2 , 4 ],  2 )),  adj=  0 ,  cex= textCex) 
        
        text ( y=  max (datRange)  -  ( 0.2   *   diff ( range (RIs))),  x=  min (datRange),  
        labels=  paste ( "y = " ,  signif ( coef (regn)[ 2 ],  2 ),  "x" ,  " + " ,  signif ( coef (regn)[ 1 ],  2 ),  sep=  "" ),  adj=  0 ,  cex= textCex) 
   } 
    
    jpeg ( filename =    "Plot_Fig1S2B.jpeg" , 
          res =   300 , 
          width =   9 ,  height =   3 ,  units =   'in'  ) 
    
     par ( bty=  "n" ,  mfrow=  c ( 1 , 3 ),  cex.main=  1.4 ,  cex.lab=  1.4 ,  cex.axis=  1.4 ) 
    
    for (i  in   1  :  3 ){ 
     plotRegress ( x= RIs[,i],  y= RIs[, 4 ],  xlab=  paste ( c ( "Anterior" ,  "Middle" ,  "Posterior" )[i],  "HY length / HS length" ),  ylab=  "Total HY length / HS length" ,  bty=  "n" ,  cex=  0.75 ,  pch=  16 ,  datRange=  range (RIs),  las=  1 ,  asp =  1 ,  textCex=  1.4 ) 
    }  
     
    dev.off ()    
       img1S2B  =   readImage ( "D:/Dropbox/z_ Ale Shared work/z_Nutrition Paper Markdown/Ale/Revision/Plot_Fig1S2B.jpeg" )  
    gob_imageFig1S2B  =   rasterGrob (img1S2B) 
     grid.draw (gob_imageFig1S2B)    
   
 ##Export Figure 1S2 
 
 
 
  1.4  Figure 1 - figure supplement 3 
 
  1.4.1  Figure 1S3A 
 
 Variation in impact of diet on midgut length in the DGRP maps to genes with functions connected to epithelial turnover. The Manhattan plot summarizes the p-value per chromosomal locus (grey bars) associated with GWAS analysis. Highlighted genes have been selected based on their statistical significance, their function, and the effect of the genetic variation (e.g. non-synonymous mutation, etc.). 
 
      img1S3A  =   readImage ( "D:/Dropbox/z_ Ale Shared work/z_Nutrition Paper Markdown/Ale/Revision/1 - S3.jpg" )  
   gob_imageFig1S3A  =   rasterGrob (img1S3A) 
    grid.draw (gob_imageFig1S3A)    
   
 ##Export Figure 
 
 
 
 
  2  Figure 2. Sugar antagonizes yeast-induced increase of midgut length 
 
  2.1  Figure 2 - main 
 
  2.1.1  Figure 2A 
 
  Midgut length is maximized at specific points in diet space.  Adult flies were maintained for 5 days from eclosion on one of 28 diets based on different caloric concentration and yeast to sucrose ratios (see figure 1-figure supplement 1A for scheme on diets used and sample size). The list of recipes can be found in Table1. The figure shows contours of a thin-plate spline (Generalized Additive Model) of length (mm, coded by colors) as a function of yeast and sucrose in diet. Colored dots represent mean of samples in a particular diet. 
 
      tab_nutri_geo  =   
     d[[ "2A" ]] %>%  
        mutate ( Total.Lmm =  Total.L  /   1000 ) 
    
   tab_nutri_geo $ YSdiet  <-   with (tab_nutri_geo, (Yeast.in.Diet) / (Sucrose.in.Diet)) 
       tab_nutri_geo $ YSingested  <-   with (tab_nutri_geo, (Yeast.ingested) / (Sucrose.ingested)) 
    
    jpeg ( filename =    "D:/Dropbox/z_ Ale Shared work/z_Nutrition Paper Markdown/Ale/Revision/Plot_Fig2A.jpeg" , 
         res =   600 , 
         width =   5 ,  height =   4 ,  units =   'in'  ) 
    par ( cex=  1 ,  mar =   c ( 4.5 ,  4.5 ,  1 ,  3 )) 
    with (tab_nutri_geo,  geomPlotta ( x =  Sucrose.in.Diet,  y =  Yeast.in.Diet,  z =  Total.Lmm,  alf =   1 ,  xlim =   c ( -  10 ,  300 ),  ylim =   c ( -  10 ,  300 ),  xlab =   "Sucrose in diet (g/L)" ,  ylab =   "Yeast in diet (g/L)" ,  frame.plot=   FALSE ,  cex.lab=  1.2 ,  cex.axis =  1 ,  las=  1 ,  labcex=  1 ,  asp=  1 ))    
      img2A  =   readImage ( "D:/Dropbox/z_ Ale Shared work/z_Nutrition Paper Markdown/Ale/Revision/Plot_Fig2A.jpeg" ) 
   gob_imageFig2A  =   rasterGrob (img2A) 
    grid.draw (gob_imageFig2A)    
   
 
 
  2.1.2  Figure 2B 
 
 Plot show an increase in midgut length with increased amount of yeast ingested. 
 
      tab_nutri_geo  =   
     d[[ "2A" ]] %>%  
        mutate ( Total.Lmm =  Total.L  /   1000 ) 
    
   tab_nutri_geo $ title1  <-   "Midgut length vs yeast ingested"  
    
   graph2  <-   ggplot (tab_nutri_geo,  aes ( x= Yeast.ingested,  y= Total.Lmm)) 
    
   Plot_Fig2B =  
     graph2  +   geom_point ( size=  2 , shape=  16 )  +   geom_smooth ( span=  1 ,  size=  1 ,  color =   "blue" )  +  
      scale_y_continuous ( "Midgut length (mm)" )  +  
      scale_x_continuous ( "Yeast ingested (g/L x absorbance)" )  +  
        theme ( panel.background =   element_blank (), 
            panel.grid.major.y =   element_line ( colour =   grey ( 0.45 ),  linetype =   "dashed" ,  size =   0.2 ), 
            axis.title.x =   element_text ( size= Smallfont, colour=  "black" ), 
            axis.title.y =   element_text ( size= Smallfont, colour=  "black" ), 
            axis.line.x =   element_line ( colour=  "black" , size=  0.75 ), 
            axis.line.y =   element_line ( colour=  "black" , size=  0.75 ), 
            axis.ticks.x =   element_line ( size =   0.75 ), 
            axis.ticks.y =   element_line ( size =   0.75 ), 
            axis.text.x =   element_text ( size= Smallfont, colour=  "black" ), 
            axis.text.y =   element_text ( size= Smallfont, colour=  "black" ), 
            plot.margin =   unit (Margin,  "cm" )) 
    
   Plot_Fig2B    
   
 
 
  2.1.3  Figure 2C 
 
 Plots show a decrease in midgut length with increased amount of sucrose ingested. 
 
      tab_nutri_geo  =   
     d[[ "2A" ]] %>%  
        mutate ( Total.Lmm =  Total.L  /   1000 ) 
    
   graph3  <-   ggplot (tab_nutri_geo,  aes ( x= Sucrose.ingested,  y= Total.Lmm)) 
    
   Plot_Fig2C =  
     graph3  +   geom_point ( size=  2 , shape=  16 )  +   geom_smooth ( span=  1 ,  size=  1 ,  color =   "red" ) +  
      scale_y_continuous ( "Midgut length (mm)" )  +  
      scale_x_continuous ( "Sucrose ingested (g/L x absorbance)" )  +  
      theme ( panel.background =   element_blank (), 
            panel.grid.major.y =   element_line ( colour =   grey ( 0.45 ),  linetype =   "dashed" ,  size =   0.2 ), 
            axis.title.x =   element_text ( size= Smallfont, colour=  "black" ), 
            axis.title.y =   element_text ( size= Smallfont, colour=  "black" ), 
            axis.line.x =   element_line ( colour=  "black" , size=  0.75 ), 
            axis.line.y =   element_line ( colour=  "black" , size=  0.75 ), 
            axis.ticks.x =   element_line ( size =   0.75 ), 
            axis.ticks.y =   element_line ( size =   0.75 ), 
            axis.text.x =   element_text ( size= Smallfont, colour=  "black" ), 
            axis.text.y =   element_text ( size= Smallfont, colour=  "black" ), 
            plot.margin =   unit (Margin,  "cm" )) 
    
   Plot_Fig2C    
   
 
 
  2.1.4  Figure 2D 
 
 Plot show an increase in midgut length with ratio of yeast to sucrose ingested. 
 
      tab_nutri_geo  =   
     d[[ "2A" ]] %>%  
        mutate ( Total.Lmm =  Total.L  /   1000 ) 
    
   graph4  <-   ggplot (tab_nutri_geo,  aes ( x= (Yeast.ingested / Sucrose.ingested),  y= Total.Lmm)) 
    
   Plot_Fig2D =  
      
   graph4  +   geom_point ( size=  2 , shape=  16 )  +   geom_smooth ( span=  1 ,  size=  1 ,  color =   "green" ) +  
      scale_y_continuous ( "Midgut length (mm)" )  +  
      scale_x_continuous ( "Y:S ingested" )  +  
      theme ( panel.background =   element_blank (), 
            panel.grid.major.y =   element_line ( colour =   grey ( 0.45 ),  linetype =   "dashed" ,  size =   0.2 ), 
            axis.title.x =   element_text ( size= Smallfont, colour=  "black" ), 
            axis.title.y =   element_text ( size= Smallfont, colour=  "black" ), 
            axis.line.x =   element_line ( colour=  "black" , size=  0.75 ), 
            axis.line.y =   element_line ( colour=  "black" , size=  0.75 ), 
            axis.ticks.x =   element_line ( size =   0.75 ), 
            axis.ticks.y =   element_line ( size =   0.75 ), 
            axis.text.x =   element_text ( size= Smallfont, colour=  "black" ), 
            axis.text.y =   element_text ( size= Smallfont, colour=  "black" ), 
            plot.margin =   unit (Margin,  "cm" )) 
    
   Plot_Fig2D    
   
 
 
  2.1.5  Figure 2E 
 
  Several nutrients from yeast (proteins, lipids, vitamins/minerals) are required to increase midgut length.  Nutrients from yeast (proteins, amino acids, lipids, cholesterol, vitamins/minerals) were added against a base diet of only the amount of sucrose found in HY and devoid of yeast. Letters above violin plots represent grouping by statistical differences (Post hoc Tukey on GLMM). Bars beneath the main plot describe caloric content provided by the different components.  Proper label annotation (in line with the chart) can be found in manuscript figures  
 
      tab_lenght_complement  =   
     d[[ "2E" ]] %>%  
      mutate_if (is.character,as.factor) %>%  
      mutate_if (is.integer,as.factor) %>%  
     dplyr ::  rename ( Diet= Food, 
            Total_Length_mm =  Total.Lmm) 
    
   tab_lenght_complement $ Diet  =   factor (tab_lenght_complement $ Diet,  levels =   c ( "HS" , 
    "S74Y0 (A)" , 
    "A+Cas2" , 
    "A+Cas4" , 
    "A+AA" , 
    "A+AA2" , 
    "A+L4" , 
    "A+Ch0.4" , 
    "A+L2+Ch0.2" , 
    "A+L4+Ch0.4" , 
    "A+V2" , 
    "A+V4" , 
    "A+C4+L4+Ch0.4" , 
    "A+C4+L4+Ch0.4+V2" , 
    "HY" ))  #Order Diets  
    
   Sample_size =  
     tab_lenght_complement %>%  
      group_by (Diet) %>%  
      summarise ( Sample_size=  n ()) 
    
    ###Stats  
    
   mod.gen  =   fitme ( log (Total_Length_mm)  ~   Diet  +  ( 1   |  Repeat),  data =  tab_lenght_complement) 
    shapiro.test ( residuals (mod.gen))     
  ## 
##  Shapiro-Wilk normality test
## 
## data:  residuals(mod.gen)
## W = 0.99393, p-value = 0.1361  
       bptest ( log (Total_Length_mm)  ~  Diet  +  ( 1   /  Repeat),  data =  tab_lenght_complement)     
  ## 
##  studentized Breusch-Pagan test
## 
## data:  log(Total_Length_mm) ~ Diet + (1/Repeat)
## BP = 41.593, df = 14, p-value = 0.0001434  
      mod.gen1  =   fitme ( log (Total_Length_mm)  ~   1   +  ( 1   |  Repeat),  data =  tab_lenght_complement)  
   test  =   anova (mod.gen, mod.gen1) 
   Chi2_LRT_growth  =   2  * (mod.gen $ APHLs[[ "p_v" ]] - mod.gen1 $ APHLs[[ "p_v" ]]) 
    
    #Now we make a tab with the results  
   tab_stat  =   data.frame ( Variable =   as.character ( paste ( "Anova diets" )), 
                                   Rep =   nlevels (tab_lenght_complement $ Repeat), 
                                   chi2_LR =   round ( as.numeric (test $ basicLRT $ chi2_LR),  digits =   2 ), 
                                   intercept =   format (mod.gen $ fixef[ 1 ], digits=  3 ), 
                                   estimate =   format (mod.gen $ fixef[ 2 ], digits=  3 ), 
                                   df =   as.numeric (test $ basicLRT $ df), 
                                   Pvalue =   as.numeric ( format ( pchisq (Chi2_LRT_growth, df=  1 , lower.tail =  F), digits=  2 ))) 
   tab_stat $ sig  =   ifelse (tab_stat $ Pvalue  <   0.05   &  tab_stat $ Pvalue  >   0.01 ,  "*" , 
                 ifelse (tab_stat $ Pvalue  <   0.01   &  tab_stat $ Pvalue  >   0.001 ,  "**" , 
                  ifelse (tab_stat $ Pvalue  <   0.001 ,  "***" ,  "" ))) 
    
   tab_stat %>%  
      kable ( col.names =   c ( "Comparison" ,  "Replicates" ,  "Chi2" , "Intercept" , "Estimate" , "df"  , "p-value" , "Signif." ), row.names =   FALSE )  %>%     add_header_above ( c ( "log(Total_Length_mm) ~  Diet + (1 | Repeat)"   =   8 )) %>%  
      kable_styling ( bootstrap_options =   c ( "striped" ,  "hover" ,  "condensed" ),  full_width =  F)    
 
 
 
 
 
log(Total_Length_mm) ~ Diet + (1 | Repeat)
 
 
 
 
 
Comparison
 
 
Replicates
 
 
Chi2
 
 
Intercept
 
 
Estimate
 
 
df
 
 
p-value
 
 
Signif.
 
 
 
 
 
 
Anova diets
 
 
3
 
 
313.48
 
 
1.45
 
 
-0.0461
 
 
14
 
 
0
 
 
***
 
 
 
 
      mod.gen  =   lmer (Total_Length_mm  ~   Diet  +  ( 1   |  Repeat),  data =  tab_lenght_complement) 
   multcomp  =   glht (mod.gen,  linfct=  mcp ( Diet=  "Tukey" )) 
    
   tmp  =   cld (multcomp) 
    
   letter_position  =   aggregate ( data= tab_lenght_complement,Total_Length_mm  ~  Diet, max) 
    
   tab_letter  =    as.data.frame (tmp $ mcletters $ Letters) 
   tab_letter $ Diet =  rownames (tab_letter) 
    colnames (tab_letter)[ 1 ]  =   "Letter"  
   tab_letter  =   left_join (tab_letter,letter_position) 
    
    ### Plot  
    
   Limits =   c ( "HS" ,  "S74Y0 (A)" ,  "A+Cas2" ,  "A+Cas4" ,  "A+AA" ,  "A+AA2" ,  "A+L4" ,  "A+Ch0.4" ,  "A+L2+Ch0.2" ,  "A+L4+Ch0.4" ,  "A+V2" ,  "A+V4" ,  "A+C4+L4+Ch0.4" , "A+C4+L4+Ch0.4+V2" ,  "HY" ) 
    
   cbbPalette  =   c ( "#FFB4B4" ,  "#f6efe5" ,  "#f6efe5" ,  "#f6efe5" ,  "#f6efe5" ,  "#f6efe5" ,  "#f6efe5" ,  "#f6efe5" ,  "#f6efe5" ,  "#f6efe5" ,  "#f6efe5" ,  "#f6efe5" ,  "#f6efe5" ,  "#f6efe5" ,  "#C3E6FC" )  
    
    
   z  =   max (tab_lenght_complement $ Total_Length_mm,  na.rm=  TRUE ) 
    
   Plot_Fig2E =  
      ggplot (tab_lenght_complement,  aes ( x =  Diet,  y =  Total_Length_mm)) +   
      geom_violin ( aes ( fill =  Diet),  draw_quantiles =   c ( 0.25 ,  0.5 ,  0.75 ),  colour =   "black" ,  size =   0.2 , adjust =   0.8 )  +  
      geom_dotplot (  colour =   "black" ,  fill =   "white" ,  binaxis =   "y" ,  stackdir =   "center" ,  binwidth =  z  /   60 )  +   
      geom_text ( data =  Sample_size,  mapping =   aes ( x =  Diet,  y =   2.2 ,  label =   paste ( "(" ,Sample_size, ")" , sep=  "" )), size=  3 ) +  
      geom_text ( data =  tab_letter,  mapping =   aes ( x =  Diet,  y =  Total_Length_mm +0.4 ,  label =  Letter), size=  3 ) +  
      geom_text ( data =  tab_stat,  mapping =   aes ( x =   2 ,  y =   7.5 ,  label =   paste ( "p=" ,Pvalue)), size=  3 ) +  
      scale_fill_manual ( limits= Limits, 
                        values= cbbPalette) +  
      scale_x_discrete ( "" , 
                       limits= Limits) +  
      scale_y_continuous ( "Midgut length (mm)" , 
                         limits=  c ( 2 , 8 ), 
                         breaks=  seq ( 3 , 7 , by=  1 )) +  
                         stat_summary ( fun =  mean,  geom =   "point" ,  size =   2 ,  shape =   18 ,  colour =   "black" ,  aes ( group =  Repeat))  +  
                         stat_summary ( fun =  mean,  geom =   "point" ,  size =   1 ,  shape =   18 ,  aes ( group =  Repeat,  colour =  Repeat))  +  
                         scale_color_manual ( values =  palette_mean)  +  
      theme ( panel.background =   element_blank (), 
            panel.grid.major.y =   element_line ( colour =   grey ( 0.45 ),  linetype =   "dashed" ,  size =   0.2 ), 
            axis.title.x =   element_blank (), 
            axis.title.y =   element_text ( size= Smallfont, colour=  "black" ),  
            axis.line.x =   element_line ( colour=  "black" , size=  0.75 ), 
            axis.line.y =   element_line ( colour=  "black" , size=  0.75 ), 
            axis.ticks.x =   element_line ( size =   0.75 ), 
            axis.ticks.y =   element_line ( size =   0.75 ), 
            axis.text.x =   element_blank (), 
            axis.text.y =   element_text ( size= Smallfont, colour=  "black" ), 
            plot.margin =   unit ( c ( 0 , 0 , 0 , 0.5 ),  "cm" ), 
            legend.direction =   "vertical" ,  
            legend.box =   "horizontal" , 
            legend.position =   "none" , 
            legend.key.height =   unit ( 0.4 ,  "cm" ), 
            legend.key.width=   unit ( 0.5 ,  "cm" ), 
            legend.title =   element_text ( face=  "italic" , size= Smallfont),  
            legend.key =   element_rect ( colour =   'white' ,  fill =   "white" ,  linetype=  'dashed' ), 
            legend.text =   element_text ( size= SuperSmallfont), 
            legend.background =   element_rect ( fill=  NA )) 
    
   tab_component_calories =   mutate_if (d[[ "2E - calories" ]],is.character,as.factor) 
   tab_component_calories $ Component  <-   factor (tab_component_calories $ Component,  levels =   c ( "Lipids" ,  "Proteins" ,  "Carbohydrates" )) 
    
   Limits_2  =   c ( "Lipids" , "Proteins" , "Carbohydrates" ) 
   Labels  =   c ( "Lipids" , "Proteins" , "Carbohydrates" ) 
    
   Plot_Fig2E_bis =  
    ggplot (tab_component_calories, aes ( x= Diet, y= Calories.contributed)) +  
    geom_bar ( stat=  "identity" , aes ( fill= Component), color=  "black" , width= . 90 ) +  
      scale_fill_manual ( limits= Limits_2, 
                          values= palette_component_3, 
                          labels= Labels) +  
      scale_x_discrete ( "" , 
                         limits= Limits) +  
      scale_y_reverse ( "Calories" , 
                         breaks=  c ( seq ( 0 , 1400 , by=  300 ))) +  
      theme ( axis.title.x =   element_blank (), 
            axis.title.y =    element_text ( size= Smallfont, colour=  "black" ), 
            axis.line.x =   element_line ( colour=  "white" ), 
            axis.line.y =   element_line ( colour=  "black" ), 
            axis.ticks.x =   element_line ( colour=  "white" ), 
            axis.ticks.y =   element_line (), 
            axis.text.x =   element_blank (), 
            axis.text.y =   element_text ( size= Smallfont, colour=  "black" ), 
            panel.grid =   element_blank (), 
            plot.margin =   unit ( c ( 0 , 0 , 0 , 0 ),  "cm" ), 
            legend.direction =   "horizontal" ,  
            legend.box =   "horizontal" , 
            legend.position =   "bottom" , 
            legend.margin=  margin ( t=  0.1 ,  r=  0.2 ,  b=  0.1 ,  l=  0 ,  unit=  "cm" ), 
            legend.title =   element_blank (),  
            legend.key =   element_rect ( colour =   'white' ,  fill =   "white" ,  linetype=  'dashed' ), 
            legend.text =   element_text ( size= Smallfont), 
            legend.background =   element_rect ( fill=  "white" ,  colour=  "black" ), 
            legend.key.size =   unit ( 0.5 , "cm" ), 
          
            strip.text.x =   element_text ( size = Mediumfont,  colour =   "black" , face=  "italic" ), 
            strip.text.y =   element_text ( size = Mediumfont,  colour =   "black" , face=  "italic" ), 
            strip.background =   element_rect ( fill=  NA ,  colour=  "black" ), 
            strip.placement=  "outside" , 
            panel.background =   element_rect ( fill=  "transparent" )) +  
      guides ( fill=  guide_legend ( ncol=  3 )) 
    
    
   plot_2E  =   grid.arrange (Plot_Fig2E, 
                          Plot_Fig2E_bis +   theme ( legend.position=  "none" ), 
                           grid.text ( "HS" , x=  0.15 ,  y=  0.2 ,  just=  "left" , gp =   gpar ( fontsize= Smallfont, fontface=  "bold" )), 
                           grid.text ( "HY" , x=  0.95 ,  y=  1 ,  just=  "left" , gp =   gpar ( fontsize= Smallfont, fontface=  "bold" )), 
                           grid.text ( "Sucrose in HY completed with:" , x=  0.21 ,  y=  0.9 , just=  "left" , gp =   gpar ( fontsize= Smallfont, fontface=  "bold" )), 
                           grid.text ( "Casein       x0  x2  x4                                            x4 x4" ,  x=  0.02 ,  y=  0.52 , just=  "left" , gp =   gpar ( fontsize= Smallfont, fontface=  "bold" )), 
                           grid.text ( "AAs            x0            x1 x2" ,  x=  0.02 ,  y=  0.51 , just=  "left" , gp =   gpar ( fontsize= Smallfont, fontface=  "bold" )), 
                           
                           grid.text ( "Lard           x0                       x4       x2 x4            x4  x4" ,  x=  0.02 ,  y=  0.50 , just=  "left" , gp =   gpar ( fontsize= Smallfont, fontface=  "bold" )), 
                           grid.text ( "Chol.          x0                            x1  x1 x1            x1  x1" ,  x=  0.02 ,  y=  0.49 , just=  "left" , gp =   gpar ( fontsize= Smallfont, fontface=  "bold" )), 
                           
                           grid.text ( "Vit.             x0                                            x2  x4       x2" ,  x=  0.02 ,  y=  0.48 , just=  "left" , gp =   gpar ( fontsize= Smallfont, fontface=  "bold" )), 
                         
                           ncol =   1 ,  heights =   c ( 2 , 1 , 0.2 , 0.15 ,  0.15 , 0.15 , 0.15 , 0.15 , 0.15 , 0.2 ))    
   
 
 
  2.1.6  Figure 2F 
 
  Midgut size is antagonized by sugar, but not other added calories.  Diet with only lipids, isocaloric with HS and HY diets, results in midguts of lengths comparable to those on HS diet. Substitution of sucrose from HS diet with isocaloric lipids (Lipids HS) results in midguts as long as those on HY. Midguts of flies reared on a diet substituting sucrose in HY diet with lipids (Lipids HY) are also similar in length to those of flies fed HY. Letters above violin plots represent grouping by statistical differences (Post hoc Tukey on GLMM). Bottom part of the chart (bar graph) describes caloric content provided by the different components. 
 
      tab_lipids  =  
     d[[ "2F" ]] %>%  
      mutate ( Total_Length_mm = Total.L /  1000 ) %>%  
      mutate_if (is.character,as.factor) %>%  
      mutate_if (is.integer,as.factor) %>%  
     dplyr ::  rename ( Diet= Food) 
    
   Sample_size =  
     tab_lipids %>%  
      group_by (Diet) %>%  
      summarise ( Sample_size=  n ()) 
    
    ###Stats  
    
   mod.gen  =   fitme ( log (Total_Length_mm)  ~   Diet  +  ( 1   |  Repeat),  data =  tab_lipids) 
    shapiro.test ( residuals (mod.gen))     
  ## 
##  Shapiro-Wilk normality test
## 
## data:  residuals(mod.gen)
## W = 0.98586, p-value = 0.2715  
       bptest ( log (Total_Length_mm)  ~  Diet  +  ( 1   /  Repeat),  data =  tab_lipids)     
  ## 
##  studentized Breusch-Pagan test
## 
## data:  log(Total_Length_mm) ~ Diet + (1/Repeat)
## BP = 6.0006, df = 4, p-value = 0.1991  
      mod.gen1  =   fitme ( log (Total_Length_mm)  ~   1   +  ( 1   |  Repeat),  data =  tab_lipids)  
   test  =   anova (mod.gen, mod.gen1)  
   Chi2_LRT_growth  =   2  * (mod.gen $ APHLs[[ "p_v" ]] - mod.gen1 $ APHLs[[ "p_v" ]]) 
    
    #Now we make a tab with the results  
   tab_stat  =   data.frame ( Variable =   as.character ( paste ( "Anova diets" )), 
                                   Rep =   nlevels (tab_lipids $ Repeat), 
                                   chi2_LR =   round ( as.numeric (test $ basicLRT $ chi2_LR),  digits =   2 ), 
                                   intercept =   format (mod.gen $ fixef[ 1 ], digits=  3 ), 
                                   estimate =   format (mod.gen $ fixef[ 2 ], digits=  3 ), 
                                   df =   as.numeric (test $ basicLRT $ df), 
                                   Pvalue =   as.numeric ( format ( pchisq (Chi2_LRT_growth, df=  1 , lower.tail =  F), digits=  2 ))) 
   tab_stat $ sig  =   ifelse (tab_stat $ Pvalue  <   0.05   &  tab_stat $ Pvalue  >   0.01 ,  "*" , 
                 ifelse (tab_stat $ Pvalue  <   0.01   &  tab_stat $ Pvalue  >   0.001 ,  "**" , 
                  ifelse (tab_stat $ Pvalue  <   0.001 ,  "***" ,  "" ))) 
    
   tab_stat %>%  
      kable ( col.names =   c ( "Comparison" ,  "Replicates" ,  "Chi2" , "Intercept" , "Estimate" , "df"  , "p-value" , "Signif." ), row.names =   FALSE )  %>%     add_header_above ( c ( "log(Total_Length_mm) ~  Diet + (1 | Repeat)"   =   8 )) %>%  
      kable_styling ( bootstrap_options =   c ( "striped" ,  "hover" ,  "condensed" ),  full_width =  F)    
 
 
 
 
 
log(Total_Length_mm) ~ Diet + (1 | Repeat)
 
 
 
 
 
Comparison
 
 
Replicates
 
 
Chi2
 
 
Intercept
 
 
Estimate
 
 
df
 
 
p-value
 
 
Signif.
 
 
 
 
 
 
Anova diets
 
 
3
 
 
112.99
 
 
1.59
 
 
0.224
 
 
4
 
 
0
 
 
***
 
 
 
 
      mod.gen  =   lmer (Total_Length_mm  ~   Diet  +  ( 1   |  Repeat),  data =  tab_lipids) 
   multcomp  =   glht (mod.gen,  linfct=  mcp ( Diet=  "Tukey" )) 
   tmp  =   cld (multcomp) 
    
   letter_position  =   aggregate ( data= tab_lipids,Total_Length_mm  ~  Diet, max) 
    
   tab_letter  =    as.data.frame (tmp $ mcletters $ Letters) 
   tab_letter $ Diet =  rownames (tab_letter) 
    colnames (tab_letter)[ 1 ]  =   "Letter"  
   tab_letter  =   left_join (tab_letter,letter_position) 
    
   Limits  =  c ( "Lard Only" , "HS" , "HS Lard sub" , "HY Lard sub" , "HY" ) 
   Labels  =  c ( "Yeast:Lipid 0:1" , "Yeast:Sugar 1:14 (HS)" , "Yeast:Lipid 1:14" , "Yeast:Lipid 1:0.7" , "Yeast:Sugar 1:0.7 (HY)" ) 
   cbbPalette  =   c ( "#f6efe5" ,  "#FFB4B4" ,  "#f6efe5" ,  "#f6efe5" ,  "#C3E6FC" ) 
    
   z  =   max (tab_lipids $ Total_Length_mm,  na.rm =   TRUE ) 
    
   Plot_Fig2F =  
      ggplot (tab_lipids,  aes ( x =  Diet,  y =  Total_Length_mm)) +   
      geom_violin ( aes ( fill =  Diet),  draw_quantiles =   c ( 0.25 ,  0.5 ,  0.75 ),  colour =   "black" ,  size =   0.2 , adjust =   0.8 ,  alpha =   0.5 )  +  
      geom_dotplot (  colour =   "black" ,  fill =   "white" ,  binaxis =   "y" ,  stackdir =   "center" ,  binwidth =  z /  60 )  +   
      geom_text ( data =  Sample_size,  mapping =   aes ( x =  Diet,  y =   2.5 ,  label =   paste ( "(" ,Sample_size, ")" , sep=  "" )), size=  3 ) +  
      geom_text ( data =  tab_stat,  mapping =   aes ( x =   1.7 ,  y =   7.5 ,  label =   paste ( "p=" , format (Pvalue, digits=  2 ))), size=  3 ) +  
      geom_text ( data =  tab_letter,  mapping =   aes ( x =  Diet,  y =  Total_Length_mm +0.4 ,  label =  Letter), size=  3 ) +  
      scale_fill_manual ( limits= Limits, 
                        values= cbbPalette) +  
      scale_x_discrete ( "" , 
                       limits= Limits, 
                       labels= Labels) +  
      scale_y_continuous ( "Midgut length (mm)" , 
                         limits=  c ( 2 , 8.2 ), 
                         breaks=  seq ( 2 , 8 , by=  1 )) +  
       stat_summary ( fun =  mean,  geom =   "point" ,  size =   3 ,  shape =   18 ,  colour =   "black" ,  aes ( group =  Repeat))  +  
                         stat_summary ( fun =  mean,  geom =   "point" ,  size =   2 ,  shape =   18 ,  aes ( group =  Repeat,  colour =  Repeat))  +  
                         scale_color_manual ( values =  palette_mean)  +  
      theme ( panel.background =   element_blank (), 
            panel.grid.major.y =   element_line ( colour =   grey ( 0.45 ),  linetype =   "dashed" ,  size =   0.2 ), 
            axis.title.x =   element_blank (), 
            axis.title.y =   element_text ( size= Smallfont, colour=  "black" ),  
            axis.line.x =   element_line ( colour=  "black" , size=  0.75 ), 
            axis.line.y =   element_line ( colour=  "black" , size=  0.75 ), 
            axis.ticks.x =   element_line ( size =   0.75 ), 
            axis.ticks.y =   element_line ( size =   0.75 ), 
            axis.text.x =   element_blank (), 
            axis.text.y =   element_text ( size= Smallfont, colour=  "black" ), 
            plot.margin =   unit ( c ( 0 , 0 , 0 , 0.5 ),  "cm" ), 
            legend.direction =   "vertical" ,  
            legend.box =   "horizontal" , 
            legend.position =   "none" , 
            legend.key.height =   unit ( 0.4 ,  "cm" ), 
            legend.key.width=   unit ( 0.6 ,  "cm" ), 
            legend.title =   element_text ( face=  "italic" , size= Smallfont),  
            legend.key =   element_rect ( colour =   'white' ,  fill =   "white" ,  linetype=  'dashed' ), 
            legend.text =   element_text ( size= SuperSmallfont), 
            legend.background =   element_rect ( fill=  NA )) 
    
   tab_component_calories_2F =   mutate_if (d[[ "2F - calories" ]],is.character,as.factor) 
   Limits_1  =  c ( "Lard Only" , "HS" , "HS Lard" , "HY Lard" , "HY" ) 
   Labels_1  =  c ( "Yeast:Lipid 0:1" , "Yeast:Sugar 1:14 (HS)" , "Yeast:Lipid 1:14" , "Yeast:Lipid 1:0.7" , "Yeast:Sugar 1:0.7 (HY)" ) 
    
   tab_component_calories_2F $ Component  <-   factor (tab_component_calories_2F $ Component,  levels =   c ( "Lipids" ,  "Proteins" ,  "Carbohydrates" )) 
    
   Limits_2  =   c ( "Lipids" , "Proteins" , "Carbohydrates" ) 
   Labels_2  =   c ( "Lipids" , "Proteins" , "Carbohydrates" ) 
    
   Plot_Fig2F_bis =  
    ggplot (tab_component_calories_2F, aes ( x= Diet, y= Calories.contributed)) +  
    geom_bar ( stat=  "identity" , aes ( fill= Component), color=  "black" , width= . 90 ) +  
      scale_x_discrete ( "" , 
                         limits= Limits_1, 
                       labels= Labels_1) +  
      scale_y_reverse ( "Calories" , 
                         breaks=  c ( seq ( 0 , 600 , by=  200 ))) +  
      scale_fill_manual ( limits= Limits_2, 
                          values= palette_component_3, 
                          labels= Labels_2) +  
      theme ( axis.title.x =   element_blank (), 
            axis.title.y =    element_text ( size= Smallfont, colour=  "black" ), 
            axis.line.x =   element_line ( colour=  "white" ), 
            axis.line.y =   element_line ( colour=  "black" ), 
            axis.ticks.x =   element_line ( colour=  "white" ), 
            axis.ticks.y =   element_line (), 
            axis.text.x =   element_text ( size= Smallfont, colour=  "black" , angle=  45 , hjust=  1 ), 
            axis.text.y =   element_text ( size= Smallfont, colour=  "black" ), 
            panel.grid =   element_blank (), 
            plot.margin =   unit ( c ( 0 , 0 , 0 , 0 ),  "cm" ), 
            legend.direction =   "horizontal" ,  
            legend.box =   "horizontal" , 
            legend.position =   "bottom" , 
            legend.key.height =   unit ( 0.3 ,  "cm" ), 
            legend.key.width=   unit ( 0.3 ,  "cm" ), 
            legend.margin=  margin ( t=  0 ,  r=  0 ,  b=  0 ,  l=  0 ,  unit=  "cm" ), 
            legend.title =   element_blank (),  
            legend.key =   element_rect ( colour =   'white' ,  fill =   "white" ,  linetype=  'dashed' ), 
            legend.text =   element_text ( size= Smallfont), 
            legend.background =   element_rect ( fill=  NA ), 
            strip.text.x =   element_text ( size = Mediumfont,  colour =   "black" , face=  "italic" ), 
            strip.text.y =   element_text ( size = Mediumfont,  colour =   "black" , face=  "italic" ), 
            strip.background =   element_rect ( fill=  NA ,  colour=  "black" ), 
            strip.placement=  "outside" , 
            panel.background =   element_rect ( fill=  "transparent" )) +  
      guides ( fill=  guide_legend ( ncol=  3 )) 
    
   g_legend =  function (a.gplot){ 
     tmp  =   ggplot_gtable ( ggplot_build (a.gplot)) 
     leg  =   which ( sapply (tmp $ grobs,  function (x) x $ name)  ==   "guide-box" ) 
     legend  =  tmp $ grobs[[leg]] 
      return (legend)} 
    
   mylegend =  g_legend (Plot_Fig2F_bis) 
    
   plot_2F  =   grid.arrange (Plot_Fig2F, 
                          Plot_Fig2F_bis +   theme ( legend.position=  "none" ), 
                           ncol =   1 ,  heights =   c ( 2 , 2 ))    
   
 
 
  2.1.7  Figure 2G 
 
  Antagonism by sugar of yeast-induced growth is not specific to sucrose.  Statistical comparisons were performed with HS vs HY for each sugar. 
 
      tab_sugars  =  
     d[[ "2G" ]] %>%  
      mutate ( Total_Length_mm = Total.L /  1000 ) %>%  
      mutate_if (is.character,as.factor) %>%  
      mutate_if (is.integer,as.factor) %>%  
     dplyr ::  rename ( Day_of_Treatment= Day) 
    
   tab_sugars $ Sugar  =   factor ( c ( "Sucrose" , "Fructose" , "Glucose" , "Maltose" ),  levels =   c ( "Sucrose" ,  "Glucose" ,  "Fructose" ,  "Maltose" )) 
    
   Sample_size =  
     tab_sugars %>%  
      group_by (Diet,Sugar) %>%  
      summarise ( Sample_size=  n ()) 
    
    ###Stats  
    # Sucrose:  
   mod.gen  =   fitme ( log (Total_Length_mm)  ~   Diet  +  ( 1   |  Repeat),  data =   subset (tab_sugars,Sugar ==  "Sucrose" )) 
    shapiro.test ( residuals (mod.gen))     
  ## 
##  Shapiro-Wilk normality test
## 
## data:  residuals(mod.gen)
## W = 0.98398, p-value = 0.9186  
       bptest ( log (Total_Length_mm)  ~  Diet  +  ( 1   /  Repeat),  data =   subset (tab_sugars,Sugar ==  "Sucrose" ))     
  ## 
##  studentized Breusch-Pagan test
## 
## data:  log(Total_Length_mm) ~ Diet + (1/Repeat)
## BP = 0.52599, df = 1, p-value = 0.4683  
      mod.gen1  =   fitme ( log (Total_Length_mm)  ~   1   +  ( 1   |  Repeat),  data =   subset (tab_sugars,Sugar ==  "Sucrose" ))  
   test  =   anova (mod.gen, mod.gen1)  
   Chi2_LRT_growth  =   2  * (mod.gen $ APHLs[[ "p_v" ]] - mod.gen1 $ APHLs[[ "p_v" ]]) 
    
   tab_stat  =   data.frame ( Variable =   as.character ( paste ( "HS vs HY sucrose" )), 
                                   Rep =   nlevels (tab_lipids $ Repeat), 
                                   chi2_LR =   round ( as.numeric (test $ basicLRT $ chi2_LR),  digits =   2 ), 
                                   intercept =   format (mod.gen $ fixef[ 1 ], digits=  3 ), 
                                   estimate =   format (mod.gen $ fixef[ 2 ], digits=  3 ), 
                                   df =   as.numeric (test $ basicLRT $ df), 
                                   Pvalue =   as.numeric ( format ( pchisq (Chi2_LRT_growth, df=  1 , lower.tail =  F), digits=  2 ))) 
   tab_stat_sucrose  = tab_stat 
    
    # Glucose:  
   mod.gen  =   fitme ( log (Total_Length_mm)  ~   Diet  +  ( 1   |  Repeat),  data =   subset (tab_sugars,Sugar ==  "Glucose" )) 
    shapiro.test ( residuals (mod.gen))     
  ## 
##  Shapiro-Wilk normality test
## 
## data:  residuals(mod.gen)
## W = 0.94939, p-value = 0.1628  
       bptest ( log (Total_Length_mm)  ~  Diet  +  ( 1   /  Repeat),  data =   subset (tab_sugars,Sugar ==  "Glucose" ))     
  ## 
##  studentized Breusch-Pagan test
## 
## data:  log(Total_Length_mm) ~ Diet + (1/Repeat)
## BP = 0.92415, df = 1, p-value = 0.3364  
      mod.gen1  =   fitme ( log (Total_Length_mm)  ~   1   +  ( 1   |  Repeat),  data =   subset (tab_sugars,Sugar ==  "Glucose" ))  
   test  =   anova (mod.gen, mod.gen1)  
   Chi2_LRT_growth  =   2  * (mod.gen $ APHLs[[ "p_v" ]] - mod.gen1 $ APHLs[[ "p_v" ]]) 
    
   tab_stat  =   data.frame ( Variable =   as.character ( paste ( "HS vs HY glucose" )), 
                                   Rep =   nlevels (tab_lipids $ Repeat), 
                                   chi2_LR =   round ( as.numeric (test $ basicLRT $ chi2_LR),  digits =   2 ), 
                                   intercept =   format (mod.gen $ fixef[ 1 ], digits=  3 ), 
                                   estimate =   format (mod.gen $ fixef[ 2 ], digits=  3 ), 
                                   df =   as.numeric (test $ basicLRT $ df), 
                                   Pvalue =   as.numeric ( format ( pchisq (Chi2_LRT_growth, df=  1 , lower.tail =  F), digits=  2 ))) 
   tab_stat_Glucose = tab_stat 
    
    # Fructose:  
   mod.gen  =   fitme ( log (Total_Length_mm)  ~   Diet  +  ( 1   |  Repeat),  data =   subset (tab_sugars,Sugar ==  "Fructose" )) 
    shapiro.test ( residuals (mod.gen))     
  ## 
##  Shapiro-Wilk normality test
## 
## data:  residuals(mod.gen)
## W = 0.96101, p-value = 0.3101  
       bptest ( log (Total_Length_mm)  ~  Diet  +  ( 1   /  Repeat),  data =   subset (tab_sugars,Sugar ==  "Fructose" ))     
  ## 
##  studentized Breusch-Pagan test
## 
## data:  log(Total_Length_mm) ~ Diet + (1/Repeat)
## BP = 0.80035, df = 1, p-value = 0.371  
      mod.gen1  =   fitme ( log (Total_Length_mm)  ~   1   +  ( 1   |  Repeat),  data =   subset (tab_sugars,Sugar ==  "Fructose" ))  
   test  =   anova (mod.gen, mod.gen1)  
   Chi2_LRT_growth  =   2  * (mod.gen $ APHLs[[ "p_v" ]] - mod.gen1 $ APHLs[[ "p_v" ]]) 
    
   tab_stat  =   data.frame ( Variable =   as.character ( paste ( "HS vs HY fructose" )), 
                                   Rep =   nlevels (tab_lipids $ Repeat), 
                                   chi2_LR =   round ( as.numeric (test $ basicLRT $ chi2_LR),  digits =   2 ), 
                                   intercept =   format (mod.gen $ fixef[ 1 ], digits=  3 ), 
                                   estimate =   format (mod.gen $ fixef[ 2 ], digits=  3 ), 
                                   df =   as.numeric (test $ basicLRT $ df), 
                                   Pvalue =   as.numeric ( format ( pchisq (Chi2_LRT_growth, df=  1 , lower.tail =  F), digits=  2 ))) 
   tab_stat_Fructose  =  tab_stat 
    
    # Maltose:  
   mod.gen  =   fitme ( log (Total_Length_mm)  ~   Diet  +  ( 1   |  Repeat),  data =   subset (tab_sugars,Sugar ==  "Maltose" )) 
    shapiro.test ( residuals (mod.gen))     
  ## 
##  Shapiro-Wilk normality test
## 
## data:  residuals(mod.gen)
## W = 0.94774, p-value = 0.1352  
       bptest ( log (Total_Length_mm)  ~  Diet  +  ( 1   /  Repeat),  data =   subset (tab_sugars,Sugar ==  "Maltose" ))     
  ## 
##  studentized Breusch-Pagan test
## 
## data:  log(Total_Length_mm) ~ Diet + (1/Repeat)
## BP = 0.080366, df = 1, p-value = 0.7768  
      mod.gen1  =   fitme ( log (Total_Length_mm)  ~   1   +  ( 1   |  Repeat),  data =   subset (tab_sugars,Sugar ==  "Maltose" ))  
   test  =   anova (mod.gen, mod.gen1) 
   Chi2_LRT_growth  =   2  * (mod.gen $ APHLs[[ "p_v" ]] - mod.gen1 $ APHLs[[ "p_v" ]]) 
    
   tab_stat  =   data.frame ( Variable =   as.character ( paste ( "HS vs HY maltose" )), 
                                   Rep =   nlevels (tab_lipids $ Repeat), 
                                   chi2_LR =   round ( as.numeric (test $ basicLRT $ chi2_LR),  digits =   2 ), 
                                   intercept =   format (mod.gen $ fixef[ 1 ], digits=  3 ), 
                                   estimate =   format (mod.gen $ fixef[ 2 ], digits=  3 ), 
                                   df =   as.numeric (test $ basicLRT $ df), 
                                   Pvalue =   as.numeric ( format ( pchisq (Chi2_LRT_growth, df=  1 , lower.tail =  F), digits=  2 ))) 
   tab_stat_Maltose =  tab_stat 
    
   tab_stat  =   rbind (tab_stat_sucrose,tab_stat_Glucose,tab_stat_Fructose,tab_stat_Maltose) 
   tab_stat $ sig  =   ifelse (tab_stat $ Pvalue  <   0.05   &  tab_stat $ Pvalue  >   0.01 ,  "*" , 
                 ifelse (tab_stat $ Pvalue  <   0.01   &  tab_stat $ Pvalue  >   0.001 ,  "**" , 
                  ifelse (tab_stat $ Pvalue  <   0.001 ,  "***" ,  "" ))) 
   tab_stat $ Sugar  =   c ( "Sucrose" , "Glucose" , "Fructose" , "Maltose" ) 
   tab_stat $ Sugar  =  as.factor (tab_stat $ Sugar) 
    
   tab_stat %>%  
      kable ( col.names =   c ( "Comparison" ,  "Replicates" ,  "Chi2" , "Intercept" , "Estimate" , "df"  , "p-value" , "Signif." , "Sugar" ), row.names =   FALSE )  %>%     add_header_above ( c ( "log(Total_Length_mm) ~  Diet + (1 | Repeat)"   =   9 )) %>%  
      kable_styling ( bootstrap_options =   c ( "striped" ,  "hover" ,  "condensed" ),  full_width =  F)    
 
 
 
 
 
log(Total_Length_mm) ~ Diet + (1 | Repeat)
 
 
 
 
 
Comparison
 
 
Replicates
 
 
Chi2
 
 
Intercept
 
 
Estimate
 
 
df
 
 
p-value
 
 
Signif.
 
 
Sugar
 
 
 
 
 
 
HS vs HY sucrose
 
 
3
 
 
42.83
 
 
1.56
 
 
0.333
 
 
1
 
 
0.0e+00
 
 
***
 
 
Sucrose
 
 
 
 
HS vs HY glucose
 
 
3
 
 
16.16
 
 
1.6
 
 
0.218
 
 
1
 
 
5.8e-05
 
 
***
 
 
Glucose
 
 
 
 
HS vs HY fructose
 
 
3
 
 
39.14
 
 
1.53
 
 
0.32
 
 
1
 
 
0.0e+00
 
 
***
 
 
Fructose
 
 
 
 
HS vs HY maltose
 
 
3
 
 
20.75
 
 
1.54
 
 
0.244
 
 
1
 
 
5.2e-06
 
 
***
 
 
Maltose
 
 
 
 
      Limits  =   c ( "HS" , "HY" ) 
   Labels =   c ( "HS" , "HY" ) 
    
   z  =   max (tab_sugars $ Total_Length_mm,  na.rm =   TRUE ) 
    
   Plot_Fig2G =  
      ggplot (tab_sugars,  aes ( x =  Diet,  y =  Total_Length_mm)) +   
      geom_violin ( aes ( fill =  Diet),  draw_quantiles =   c ( 0.25 ,  0.5 ,  0.75 ),  colour =   "black" ,  size =   0.2 , adjust =   0.8 )  +  
      geom_dotplot ( colour =   "black" ,  fill =   "white" ,  binaxis =   "y" ,  stackdir =   "center" ,  binwidth =  z  /   60 )  +   
      geom_text ( data =  Sample_size,  mapping =   aes ( x =  Diet,  y =   2.5 ,  label =   paste ( "(" ,Sample_size, ")" , sep=  "" )), size=  3 ) +  
      geom_signif ( data =  tab_stat, aes ( xmin =   1 ,  xmax =   2 ,  annotations =   formatC ( paste ( "p=" ,Pvalue),  digits =   2 ),  y_position =   8.5 ),  textsize =   3 ,  vjust =   -  0.2 ,  manual =   TRUE ) +  
      facet_grid (. ~ Sugar) +  
      scale_fill_manual ( limits= Limits, 
                        values= palette_diet_2) +  
      scale_x_discrete ( "" , 
                       limits= Limits, 
                       labels= Labels) +  
      scale_y_continuous ( "Midgut length (mm)" , 
                         limits=  c ( 2 , 9 ), 
                         breaks=  seq ( 2 , 8 , by=  1 )) +  
       stat_summary ( fun =  mean,  geom =   "point" ,  size =   3 ,  shape =   18 ,  colour =   "black" ,  aes ( group =  Repeat))  +  
                         stat_summary ( fun =  mean,  geom =   "point" ,  size =   2 ,  shape =   18 ,  aes ( group =  Repeat,  colour =  Repeat))  +  
                         scale_color_manual ( values =  palette_mean)  +  
      theme ( panel.background =   element_blank (), 
            panel.grid.major.y =   element_line ( colour =   grey ( 0.45 ),  linetype =   "dashed" ,  size =   0.2 ), 
            axis.title.x =   element_blank (), 
            axis.title.y =   element_text ( size= Smallfont, colour=  "black" ),  
            axis.line.x =   element_line ( colour=  "black" , size=  0.75 ), 
            axis.line.y =   element_line ( colour=  "black" , size=  0.75 ), 
            axis.ticks.x =   element_line ( size =   0.75 ), 
            axis.ticks.y =   element_line ( size =   0.75 ), 
            axis.text.x =   element_text ( size= Smallfont, colour=  "black" ),  
            axis.text.y =   element_text ( size= Smallfont, colour=  "black" ), 
            plot.margin =   unit ( c ( 0 , 0 , 0 , 0.5 ),  "cm" ), 
            legend.direction =   "vertical" ,  
            legend.box =   "horizontal" , 
            legend.position =   "none" , 
            legend.key.height =   unit ( 0.4 ,  "cm" ), 
            legend.key.width=   unit ( 0.6 ,  "cm" ), 
            legend.title =   element_text ( face=  "italic" , size= Smallfont),  
            legend.key =   element_rect ( colour =   'white' ,  fill =   "white" ,  linetype=  'dashed' ), 
            legend.text =   element_text ( size= SuperSmallfont), 
            legend.background =   element_rect ( fill=  NA ), 
            strip.text.x =   element_text ( size =  Smallfont,  colour =   "black" ,  margin =   margin ( t =   2 ,  r =   0 ,  b =   2 ,  l =   0 )), 
            strip.text.y =   element_text ( size =  Smallfont,  colour =   "black" ,  margin =   margin ( t =   2 ,  r =   0 ,  b =   2 ,  l =   0 )), 
            strip.background =   element_rect ( fill=  NA ,  colour=  "black" ), 
            strip.placement=  "outside" ) 
    
   Plot_Fig2G    
   
 ##Export Figure 2 
 
 
 
  2.2  Figure 2 - figure supplement 1 
 
  2.2.1  Figure 2S1A 
 
 Set of diets utilized for the nutritional geometry experiment. Numbers on dots denote sample sizes for figure 2A (pool of three independent biological replicates). We utilized 28 different diets, varying either caloric content or the yeast to sucrose ratio. The complete list of recipes can be found in Table 1.  Complete numbers can be found in manuscript figure  
 
      tab_nutri_geo_design  =   
     d[[ "2 - S1A" ]] %>%  
     dplyr ::  rename ( Yeast.in.Diet= Yeast, 
             Sucrose.in.Diet= Sugar) 
    
   tab_raw_2A  =   
     d[[ "2A" ]] %>%  
      mutate_if (is.character,as.factor) %>%  
      mutate_if (is.integer,as.factor) %>%  
      mutate ( Conc =   paste ( round (Sucrose.in.Diet, digits=  2 ), "x" , round (Yeast.in.Diet, digits=  2 ), sep=  "" )) %>%  
      group_by (Yeast.in.Diet,Sucrose.in.Diet) %>%  
      summarise ( Sample_size=  n ()) 
    
   tab_nutri_geo_design  =   left_join (tab_nutri_geo_design,tab_raw_2A) 
    
   Plot_Fig2S1A =  
      ggplot (tab_nutri_geo_design,  aes ( x =  Sucrose.in.Diet,  y =  Yeast.in.Diet, label= Sample_size)) +   
      geom_point ( aes ( size= Calories),  colour =   "black" )  +   
      scale_x_continuous ( "Sucrose in diet (g/L)" , 
                         limits=  c ( -  5 , 300 ), 
                         breaks=  seq ( 0 , 300 , by=  100 )) +  
      scale_y_continuous ( "Yeast in diet (g/L)" , 
                         limits=  c ( -  5 , 300 ), 
                         breaks=  seq ( 0 , 300 , by=  100 )) +  
      geom_text ( data=  subset (tab_nutri_geo_design,Yeast.in.Diet >=  30   |  Sucrose.in.Diet >=  30 ), color=  "white" , size=  3 ) +  
      scale_size_continuous ( range =   c ( 1 , 13 ))  +  
      theme ( panel.background =   element_blank (), 
            panel.grid.major =   element_line ( colour =   "black" , linetype=  3 ), 
            axis.title.x =   element_text ( size= Smallfont, colour=  "black" ),  
            axis.title.y =   element_text ( size= Smallfont, colour=  "black" ),  
            axis.line.x =   element_line ( colour=  "black" , size=  0.75 ), 
            axis.line.y =   element_line ( colour=  "black" , size=  0.75 ), 
            axis.ticks.x =   element_line ( size =   0.75 ), 
            axis.ticks.y =   element_line ( size =   0.75 ), 
            axis.text.x =   element_text ( size= Smallfont, colour=  "black" ),  
            axis.text.y =   element_text ( size= Smallfont, colour=  "black" ), 
            plot.margin =   unit ( c ( 0 , 0 , 0 , 0.5 ),  "cm" ), 
            legend.direction =   "horizontal" ,  
            legend.box =   "horizontal" , 
            legend.position =   "top" , 
            legend.key.height =   unit ( 0.4 ,  "cm" ), 
            legend.key.width=   unit ( 0.6 ,  "cm" ), 
            legend.title =   element_text ( face=  "italic" , size= Smallfont),  
            legend.key =   element_rect ( colour =   'white' ,  fill =   "white" ,  linetype=  'dashed' ), 
            legend.text =   element_text ( size= SuperSmallfont), 
            legend.background =   element_rect ( fill=  NA ), 
            strip.text.x =   element_text ( size = Smallfont,  colour =   "black" , face=  "italic" ), 
            strip.text.y =   element_text ( size = Smallfont,  colour =   "black" , face=  "italic" ), 
            strip.background =   element_rect ( fill=  NA ,  colour=  "black" ), 
            strip.placement=  "outside" ) +  
      guides ( size=  guide_legend ( ncol=  4 )) 
    
   Plot_Fig2S1A    
   
 
 HS and HY diet compared to standard diets used for Drosophila 
 
 
 
  2.2.2  Figure 2S1B 
 
 FD&C1 blue transit assay (feeding assay) showing amount of food defecated, and by inference ingested, in the nutritional geometry experiment. The scale maps color to units. The graph indicates compensatory feeding at lower nutrient densities, especially low yeast. These data were used to calculate the total amount of yeast and sucrose ingested on each diet in Figure 2-supplemental figure 1C. 
 
      tab_geom_fecal  =   
     d[[ "2 - S1B" ]] 
    
    jpeg ( filename =   "D:/Dropbox/z_ Ale Shared work/z_Nutrition Paper Markdown/Ale/Revision/Plot_Fig2-S1B.jpeg" , 
         res =   600 , 
         width =   5 ,  height =   4 ,  units =   'in'  ) 
    par ( cex=  1 ,  mar =   c ( 4 ,  4 ,  1 ,  3 )) 
    with (tab_geom_fecal,  geomPlotta ( x =  Sucrose.in.Diet,  y =  Yeast.in.Diet,  z =  (Absorbance *  10 ),  alf =   1 ,  xlim =   c ( -  10 ,  300 ),  ylim =   c ( -  10 ,  300 ),  xlab =   "Sucrose in diet (g/L)" ,  ylab =   "Yeast in diet (g/L)" ,  frame.plot=   FALSE ,  cex.lab=  1 ,  cex.axis =  1 ,  las=  1 ,  labcex=  1 ,  asp=  1 ))    
      img2S1B  =   readImage ( "D:/Dropbox/z_ Ale Shared work/z_Nutrition Paper Markdown/Ale/Revision/Plot_Fig2-S1B.jpeg" )  
   gob_imageFig2S1B  =   rasterGrob (img2S1B) 
    grid.draw (gob_imageFig2S1B)    
   
 
 
  2.2.3  Figure 2S1C 
 
 Yeast and sucrose have mutually antagonistic impacts on midgut length. Plots show midgut length as a function of sucrose or yeast ingested (g/L x Absorbance from Figure 2-supplemental figure 1B), or their ratio multiplied by ingestion per diet. 
 
      tab_nutri_geo  =   
     d[[ "2A" ]] %>%  
        mutate ( Total.Lmm =  Total.L  /   1000 ) 
    
   tab_nutri_geo2  <-  tab_nutri_geo  %>%  
        group_by (concatenate)  %>%  
        summarize ( Calories.ingested =   mean (Calories.ingested), 
                  Midgut.length =   mean (Total.Lmm), 
                  Yeast.ingested =   mean (Yeast.ingested), 
                  Sucrose.ingested =   mean (Sucrose.ingested)) 
    
   graph  <-   ggplot (tab_nutri_geo2,  aes ( x= Sucrose.ingested,  y= Yeast.ingested)) 
    
   Plot_Fig2S1C =  
     graph  +   geom_point ( aes ( size= Calories.ingested,  fill= Midgut.length),  stroke=  1.5 ,  shape=  21 ,   color=  "black" )  +  
      scale_size ( range =   c ( 1 , 5 ))  +  
      scale_fill_viridis_c ()  +  
      theme ( plot.title=   element_text ( hjust =   0.5 )) +  
      scale_x_continuous ( "Sucrose ingested (g/L x Absorbance)" , 
                         limits=  c ( -  5 , 160 ), 
                         breaks=  seq ( 0 , 160 , by=  25 )) +  
      scale_y_continuous ( "Yeast ingested (g/L x Absorbance)" , 
                         limits=  c ( -  5 , 50 ), 
                         breaks=  seq ( 0 , 50 , by=  10 )) +  
      scale_size_continuous ( range =   c ( 1 , 10 ))  +  
      theme ( panel.background =   element_blank (), 
            panel.grid.major =   element_line ( colour =   "black" , linetype=  3 ), 
            axis.title.x =   element_text ( size= Smallfont, colour=  "black" ),  
            axis.title.y =   element_text ( size= Smallfont, colour=  "black" ),  
            axis.line.x =   element_line ( colour=  "black" , size=  0.75 ), 
            axis.line.y =   element_line ( colour=  "black" , size=  0.75 ), 
            axis.ticks.x =   element_line ( size =   0.75 ), 
            axis.ticks.y =   element_line ( size =   0.75 ), 
            axis.text.x =   element_text ( size= Smallfont, colour=  "black" ),  
            axis.text.y =   element_text ( size= Smallfont, colour=  "black" ), 
            plot.margin =   unit ( c ( 0 , 0 , 0 , 0.5 ),  "cm" ), 
            legend.direction =   "horizontal" ,  
            legend.box =   "vertical" , 
            legend.position =   c ( 0.79 , 0.79 ), 
            legend.key.height =   unit ( 0.3 ,  "cm" ), 
            legend.key.width=   unit ( 0.4 ,  "cm" ), 
            legend.title =   element_text ( face=  "italic" , size= Smallfont),  
            legend.key =   element_rect ( colour =   'white' ,  fill =   "white" ,  linetype=  'dashed' ), 
            legend.text =   element_text ( size= SuperSmallfont), 
            strip.text.x =   element_text ( size = Smallfont,  colour =   "black" , face=  "italic" ), 
            strip.text.y =   element_text ( size = Smallfont,  colour =   "black" , face=  "italic" ), 
            strip.background =   element_rect ( fill=  NA ,  colour=  "black" ), 
            strip.placement=  "outside" ) +  
      labs ( fill =   "Midgut length (mm)" ,  size =   "Calories ingested" ) 
    
   Plot_Fig2S1C    
   
 ##Export Figure 2S1 
 
 
 
  2.3  Figure 2 - figure supplement 2 
 
  2.3.1  Figure 2S2A 
 
  Food texture does not explain the differential effects of HS and HY diets on midgut length.  Addition of inulin (inu), pectin (pect), cellulose (cell), and all previous fibers mixed (AF) or pectin + cellulose (PC) to HS does not increase midgut length. Addition of fibers to HY (HY + AF) and HY + pectin + cellulose (HY + PC2) does not affect midgut length. 
 
      tab_fiber  =   
     d[[ "2 - S2A" ]] %>%  
      mutate_if (is.character,as.factor) %>%  
      mutate_if (is.integer,as.factor) %>%  
      mutate ( Total_Length_mm =  Total.L /  1000 ) %>%  
     dplyr ::  rename ( Diet= Food) 
    
   tab_fiber $ Diet  =   factor (tab_fiber $ Diet,  levels =   c ( "HS" ,  "HS + Inu" ,  "HS + Pect" ,  "HS + Pect2" ,  "HS + Cell" ,  "HS + Cell2" ,  "HS + AF" ,  "HS + PC2" ,  "HY + AF" ,  "HY + PC2" ,  "HY" ))  
    
   Sample_size =  
     tab_fiber %>%  
      group_by (Diet) %>%  
      summarise ( Sample_size=  n ()) 
    
    ###Stats  
    
   mod.gen  =   fitme ( log (Total_Length_mm)  ~   Diet  +  ( 1   |  Repeat),  data =  tab_fiber) 
    shapiro.test ( residuals (mod.gen))     
  ## 
##  Shapiro-Wilk normality test
## 
## data:  residuals(mod.gen)
## W = 0.9942, p-value = 0.4463  
       bptest ( log (Total_Length_mm)  ~  Diet  +  ( 1   /  Repeat),  data =  tab_fiber)     
  ## 
##  studentized Breusch-Pagan test
## 
## data:  log(Total_Length_mm) ~ Diet + (1/Repeat)
## BP = 7.5012, df = 10, p-value = 0.6774  
      mod.gen1  =   fitme ( log (Total_Length_mm)  ~   1   +  ( 1   |  Repeat),  data =  tab_fiber)  
   test  =   anova (mod.gen, mod.gen1)  
    
   Chi2_LRT_growth  =   2  * (mod.gen $ APHLs[[ "p_v" ]] - mod.gen1 $ APHLs[[ "p_v" ]]) 
    
    #Now we make a tab with the results  
   tab_stat  =   data.frame ( Variable =   as.character ( paste ( "Any difference" )), 
                                   Rep =   nlevels (tab_fiber $ Repeat), 
                                   chi2_LR =   round ( as.numeric (test $ basicLRT $ chi2_LR),  digits =   2 ), 
                                   intercept =   format (mod.gen $ fixef[ 1 ], digits=  3 ), 
                                   estimate =   format (mod.gen $ fixef[ 2 ], digits=  3 ), 
                                   df =   as.numeric (test $ basicLRT $ df), 
                                   Pvalue =   as.numeric ( format ( pchisq (Chi2_LRT_growth, df=  1 , lower.tail =  F), digits=  2 ))) 
   tab_stat $ sig  =   ifelse (tab_stat $ Pvalue  <   0.05   &  tab_stat $ Pvalue  >   0.01 ,  "*" , 
                 ifelse (tab_stat $ Pvalue  <   0.01   &  tab_stat $ Pvalue  >   0.001 ,  "**" , 
                  ifelse (tab_stat $ Pvalue  <   0.001 ,  "***" ,  "" ))) 
    
   tab_stat %>%  
      kable ( col.names =   c ( "Comparison" ,  "Replicates" ,  "Chi2" , "Intercept" , "Estimate" , "df"  , "p-value" , "Signif." ), row.names =   FALSE )  %>%     add_header_above ( c ( "log(Total_Length_mm) ~  Diet + (1 | Repeat)"   =   8 )) %>%  
      kable_styling ( bootstrap_options =   c ( "striped" ,  "hover" ,  "condensed" ),  full_width =  F)    
 
 
 
 
 
log(Total_Length_mm) ~ Diet + (1 | Repeat)
 
 
 
 
 
Comparison
 
 
Replicates
 
 
Chi2
 
 
Intercept
 
 
Estimate
 
 
df
 
 
p-value
 
 
Signif.
 
 
 
 
 
 
Any difference
 
 
3
 
 
122.76
 
 
1.55
 
 
-0.0361
 
 
10
 
 
0
 
 
***
 
 
 
 
      mod.gen  =   lmer (Total_Length_mm  ~   Diet  +  ( 1   |  Repeat),  data =  tab_fiber) 
   multcomp  =   glht (mod.gen,  linfct=  mcp ( Diet=  "Tukey" )) 
    
   tmp  =   cld (multcomp) 
    
   letter_position  =   aggregate ( data=  subset (tab_fiber, !  is.na (Total_Length_mm)),Total_Length_mm  ~  Diet, max) 
    
   tab_letter  =    as.data.frame (tmp $ mcletters $ Letters) 
   tab_letter $ Diet =  rownames (tab_letter) 
    colnames (tab_letter)[ 1 ]  =   "Letter"  
   tab_letter  =   left_join (tab_letter,letter_position) 
    
    ### Plot  
    
   Limits =   c ( "HS" ,  "HS + Inu" ,  "HS + Pect" ,  "HS + Pect2" ,  "HS + Cell" ,  "HS + Cell2" ,  "HS + AF" ,  "HS + PC2" ,  "HY + AF" ,  "HY + PC2" ,  "HY" ) 
    
   cbbPalette  =   c ( "#FFB4B4" ,  "#f6efe5" ,  "#f6efe5" ,  "#f6efe5" ,  "#f6efe5" ,  "#f6efe5" ,  "#f6efe5" , "#f6efe5" ,  "#f6efe5" ,  "#f6efe5" ,  "#C3E6FC" ) 
   z  =   max (tab_fiber $ Total_Length_mm,  na.rm =   TRUE ) 
    
   Plot_Fig2S2A =  
      ggplot (tab_fiber,  aes ( x =  Diet,  y =  Total_Length_mm)) +   
      geom_violin ( aes ( fill =  Diet),  draw_quantiles =   c ( 0.25 ,  0.5 ,  0.75 ),  colour =   "black" ,  size =   0.2 , adjust =   0.8 )  +  
      geom_dotplot (  colour =   "black" ,  fill =   "white" ,  binaxis =   "y" ,  stackdir =   "center" ,  binwidth =  z /  60 )  +   
      geom_text ( data =  Sample_size,  mapping =   aes ( x =  Diet,  y =   2.5 ,  label =   paste ( "(" ,Sample_size, ")" , sep=  "" )), size=  3 ) +  
      geom_text ( data =  tab_letter,  mapping =   aes ( x =  Diet,  y =  Total_Length_mm +0.4 ,  label =  Letter), size=  3 ) +  
      geom_text ( data =  tab_stat,  mapping =   aes ( x =   1.5 ,  y =   7.5 ,  label =   paste ( "p=" , format (Pvalue, digits=  2 ))), size=  3 ) +  
      scale_fill_manual ( limits= Limits, 
                        values= cbbPalette) +  
      scale_x_discrete ( "" , 
                       limits= Limits) +  
      scale_y_continuous ( "Midgut length (mm)" , 
                         limits=  c ( 2 , 8 ), 
                         breaks=  seq ( 2 , 8 , by=  1 )) +  
      stat_summary ( fun =  mean,  geom =   "point" ,  size =   3 ,  shape =   18 ,  colour =   "black" ,  aes ( group =  Repeat))  +  
      stat_summary ( fun =  mean,  geom =   "point" ,  size =   2 ,  shape =   18 ,  aes ( group =  Repeat,  colour =  Repeat))  +  
      scale_color_manual ( values =  palette_mean)  +  
      theme ( panel.background =   element_blank (), 
            panel.grid.major.y =   element_line ( colour =   grey ( 0.45 ),  linetype =   "dashed" ,  size =   0.2 ), 
            axis.title.x =   element_blank (), 
            axis.title.y =   element_text ( size= Smallfont, colour=  "black" ),  
            axis.line.x =   element_line ( colour=  "black" , size=  0.75 ), 
            axis.line.y =   element_line ( colour=  "black" , size=  0.75 ), 
            axis.ticks.x =   element_line ( size =   0.75 ), 
            axis.ticks.y =   element_line ( size =   0.75 ), 
            axis.text.x =   element_text ( size= Smallfont, colour=  "black" , angle=  45 , hjust=  1 ), 
            axis.text.y =   element_text ( size= Smallfont, colour=  "black" ), 
            plot.margin =   unit ( c ( 0 , 0 , 0 , 0.5 ),  "cm" ), 
            legend.direction =   "vertical" ,  
            legend.box =   "horizontal" , 
            legend.position =   "none" , 
            legend.key.height =   unit ( 0.4 ,  "cm" ), 
            legend.key.width=   unit ( 0.6 ,  "cm" ), 
            legend.title =   element_text ( face=  "italic" , size= Smallfont),  
            legend.key =   element_rect ( colour =   'white' ,  fill =   "white" ,  linetype=  'dashed' ), 
            legend.text =   element_text ( size= SuperSmallfont), 
            legend.background =   element_rect ( fill=  NA )) 
    
   Plot_Fig2S2A    
   
 
 
  2.3.2  Figure 2S2B 
 
  Changes in food texture due to variation in agar concentration can affect midgut length but do not explain the effect of the diet treatment.  Changes in agar concentration do not change midgut length on HS. Either increasing or decreasing agar concentration reduces midgut length on HY. 
 
      tab_agar  =    
     d[[ "2 - S2B" ]] %>%  
      mutate_if (is.character,as.factor) %>%  
      mutate_if (is.integer,as.factor) %>%  
      mutate ( Total_Length_mm =  Total.L /  1000 ) %>%  
     dplyr ::  rename ( Diet= Food) 
    
    levels (tab_agar $ Diet)[ levels (tab_agar $ Diet) ==  "HS" ]  =  "HS (original, 1.5%)"  
    levels (tab_agar $ Diet)[ levels (tab_agar $ Diet) ==  "HY" ]  =  "HY (original, 1.5%)"  
    
   Sample_size =  
     tab_agar %>%  
      group_by (Diet) %>%  
      summarise ( Sample_size=  n ()) 
    
    ###Stats  
    
   mod.gen  =   fitme ( log (Total_Length_mm)  ~   Diet  +  ( 1   |  Repeat),  data =  tab_agar) 
    shapiro.test ( residuals (mod.gen))     
  ## 
##  Shapiro-Wilk normality test
## 
## data:  residuals(mod.gen)
## W = 0.99568, p-value = 0.4148  
       bptest ( log (Total_Length_mm)  ~  Diet  +  ( 1   /  Repeat),  data =  tab_agar)     
  ## 
##  studentized Breusch-Pagan test
## 
## data:  log(Total_Length_mm) ~ Diet + (1/Repeat)
## BP = 5.8743, df = 7, p-value = 0.5545  
      mod.gen1  =   fitme ( log (Total_Length_mm)  ~   1   +  ( 1   |  Repeat),  data =  tab_agar)  
   test  =   anova (mod.gen, mod.gen1) 
    
   Chi2_LRT_growth  =   2  * (mod.gen $ APHLs[[ "p_v" ]] - mod.gen1 $ APHLs[[ "p_v" ]]) 
    
    #Now we make a tab with the results  
   tab_stat  =   data.frame ( Variable =   as.character ( paste ( "Any difference" )), 
                                   Rep =   nlevels (tab_agar $ Repeat), 
                                   chi2_LR =   round ( as.numeric (test $ basicLRT $ chi2_LR),  digits =   2 ), 
                                   intercept =   format (mod.gen $ fixef[ 1 ], digits=  3 ), 
                                   estimate =   format (mod.gen $ fixef[ 2 ], digits=  3 ), 
                                   df =   as.numeric (test $ basicLRT $ df), 
                                   Pvalue =   as.numeric ( format ( pchisq (Chi2_LRT_growth, df=  1 , lower.tail =  F), digits=  2 ))) 
   tab_stat $ sig  =   ifelse (tab_stat $ Pvalue  <   0.05   &  tab_stat $ Pvalue  >   0.01 ,  "*" , 
                 ifelse (tab_stat $ Pvalue  <   0.01   &  tab_stat $ Pvalue  >   0.001 ,  "**" , 
                  ifelse (tab_stat $ Pvalue  <   0.001 ,  "***" ,  "" ))) 
    
   tab_stat %>%  
      kable ( col.names =   c ( "Comparison" ,  "Replicates" ,  "Chi2" , "Intercept" , "Estimate" , "df"  , "p-value" , "Signif." ), row.names =   FALSE )  %>%     add_header_above ( c ( "log(Total_Length_mm) ~  Diet + (1 | Repeat)"   =   8 )) %>%  
      kable_styling ( bootstrap_options =   c ( "striped" ,  "hover" ,  "condensed" ),  full_width =  F)    
 
 
 
 
 
log(Total_Length_mm) ~ Diet + (1 | Repeat)
 
 
 
 
 
Comparison
 
 
Replicates
 
 
Chi2
 
 
Intercept
 
 
Estimate
 
 
df
 
 
p-value
 
 
Signif.
 
 
 
 
 
 
Any difference
 
 
6
 
 
201.35
 
 
1.49
 
 
-5.9e-05
 
 
7
 
 
0
 
 
***
 
 
 
 
      mod.gen  =   lmer (Total_Length_mm  ~   Diet  +  ( 1   |  Repeat),  data =  tab_agar) 
   multcomp  =   glht (mod.gen,  linfct=  mcp ( Diet=  "Tukey" )) 
    
   tmp  =   cld (multcomp) 
    
   letter_position  =   aggregate ( data=  subset (tab_agar, !  is.na (Total_Length_mm)),Total_Length_mm  ~  Diet, max) 
    
   tab_letter  =    as.data.frame (tmp $ mcletters $ Letters) 
   tab_letter $ Diet =  rownames (tab_letter) 
    colnames (tab_letter)[ 1 ]  =   "Letter"  
   tab_letter  =   left_join (tab_letter,letter_position) 
    
    ### Plot  
    
   Limits =   c ( "HS Agar 0.5%" ,  "HS Agar 1%" ,  "HS (original, 1.5%)" ,  "HS Agar 3%" ,  "HY Agar 0.5%" ,  "HY Agar 1%" ,  "HY (original, 1.5%)" ,  "HY Agar 3%" ) 
    
   cbbPalette  =   c ( "#FFB4B4" ,  "#FFB4B4" ,  "#FFB4B4" ,  "#FFB4B4" ,  "#C3E6FC" ,  "#C3E6FC" ,  "#C3E6FC" ,  "#C3E6FC" ) 
   z  =   max (tab_agar $ Total_Length_mm,  na.rm =   TRUE ) 
    
   Plot_Fig2S2B =  
      ggplot (tab_agar,  aes ( x =  Diet,  y =  Total_Length_mm)) +   
      geom_violin ( aes ( fill =  Diet),  draw_quantiles =   c ( 0.25 ,  0.5 ,  0.75 ),  colour =   "black" ,  size =   0.2 , adjust =   0.8 )  +  
      geom_dotplot (  colour =   "black" ,  fill =   "white" ,  binaxis =   "y" ,  stackdir =   "center" ,  binwidth =  z /  60 )  +   
      geom_text ( data =  Sample_size,  mapping =   aes ( x =  Diet,  y =   2.5 ,  label =   paste ( "(" ,Sample_size, ")" , sep=  "" )), size=  3 ) +  
      geom_text ( data =  tab_letter,  mapping =   aes ( x =  Diet,  y =  Total_Length_mm +0.4 ,  label =  Letter), size=  3 ) +  
      geom_text ( data =  tab_stat,  mapping =   aes ( x =   1.5 ,  y =   7.5 ,  label =   paste ( "p=" ,Pvalue)), size=  3 ) +  
      scale_fill_manual ( limits= Limits, 
                        values= cbbPalette) +  
      scale_x_discrete ( "" , 
                       limits= Limits) +  
      scale_y_continuous ( "Midgut length (mm)" , 
                         limits=  c ( 2 , 8 ), 
                         breaks=  seq ( 2 , 8 , by=  1 )) +  
      stat_summary ( fun =  mean,  geom =   "point" ,  size =   3 ,  shape =   18 ,  colour =   "black" ,  aes ( group =  Repeat))  +  
      stat_summary ( fun =  mean,  geom =   "point" ,  size =   2 ,  shape =   18 ,  aes ( group =  Repeat,  colour =  Repeat))  +  
      scale_color_manual ( values =  palette_mean)  +  
      theme ( panel.background =   element_blank (), 
            panel.grid.major.y =   element_line ( colour =   grey ( 0.45 ),  linetype =   "dashed" ,  size =   0.2 ), 
            axis.title.x =   element_blank (), 
            axis.title.y =   element_text ( size= Smallfont, colour=  "black" ),  
            axis.line.x =   element_line ( colour=  "black" , size=  0.75 ), 
            axis.line.y =   element_line ( colour=  "black" , size=  0.75 ), 
            axis.ticks.x =   element_line ( size =   0.75 ), 
            axis.ticks.y =   element_line ( size =   0.75 ), 
            axis.text.x =   element_text ( size= Smallfont, colour=  "black" , angle=  34 , hjust=  1 ), 
            axis.text.y =   element_text ( size= Smallfont, colour=  "black" ), 
            plot.margin =   unit ( c ( 0 , 0 , 0 , 0.5 ),  "cm" ), 
            legend.direction =   "vertical" ,  
            legend.box =   "horizontal" , 
            legend.position =   "none" , 
            legend.key.height =   unit ( 0.4 ,  "cm" ), 
            legend.key.width=   unit ( 0.6 ,  "cm" ), 
            legend.title =   element_text ( face=  "italic" , size= Smallfont),  
            legend.key =   element_rect ( colour =   'white' ,  fill =   "white" ,  linetype=  'dashed' ), 
            legend.text =   element_text ( size= SuperSmallfont), 
            legend.background =   element_rect ( fill=  NA )) 
    
   Plot_Fig2S2B    
   
 
 
  2.3.3  Figure 2S2C 
 
 Sorbitol, a nutritious but not palatable sugar, has increased size on HY compared to HS, while Arabinose, a palatable but not nutritious sugar, results in death of flies before reaching dissection day on HS, and decreased size of midguts on HY diet. Statistical analysis is HS vs HY for each sugar. 
 
      tab_xtrsugars_rev  =  
     d[[ "2S2C" ]] %>%  
      mutate ( Total_Length_mm = Total.L /  1000 ) %>%  
      mutate_if (is.character,as.factor) %>%  
      mutate_if (is.integer,as.factor) %>%  
     dplyr ::  rename ( Day_of_Treatment= Day) %>%  
      mutate ( Sugar=  fct_relevel (Sugar, "Sucrose" , "Sorbitol" ,  "Arabinose" )) 
    
    #tab_xtrsugars_rev$Sugar = factor(c("Sucrose","Sorbitol","Arabinose"), levels = c("Sucrose", "Sorbitol", "Arabinose"))  
    
   Sample_size =  
     tab_xtrsugars_rev %>%  
      group_by (Diet,Sugar) %>%  
      summarise ( Sample_size=  n ()) 
    
    ###Stats  
    # Sucrose:  
   mod.gen  =   fitme ((Total_Length_mm)  ~   Diet  +  ( 1   |  Repeat),  data =   subset (tab_xtrsugars_rev,Sugar ==  "Sucrose" )) 
    shapiro.test ( residuals (mod.gen))     
  ## 
##  Shapiro-Wilk normality test
## 
## data:  residuals(mod.gen)
## W = 0.96327, p-value = 0.07213  
       bptest ((Total_Length_mm)  ~  Diet  +  ( 1   /  Repeat),  data =   subset (tab_xtrsugars_rev,Sugar ==  "Sucrose" ))     
  ## 
##  studentized Breusch-Pagan test
## 
## data:  (Total_Length_mm) ~ Diet + (1/Repeat)
## BP = 0.77017, df = 1, p-value = 0.3802  
      mod.gen1  =   fitme ((Total_Length_mm)  ~   1   +  ( 1   |  Repeat),  data =   subset (tab_xtrsugars_rev,Sugar ==  "Sucrose" ))  
   test  =   anova (mod.gen, mod.gen1)  
   Chi2_LRT_growth  =   2  * (mod.gen $ APHLs[[ "p_v" ]] - mod.gen1 $ APHLs[[ "p_v" ]]) 
    
   tab_stat  =   data.frame ( Variable =   as.character ( paste ( "HS vs HY Sucrose" )), 
                                   Rep =   nlevels (tab_lipids $ Repeat), 
                                   chi2_LR =   round ( as.numeric (test $ basicLRT $ chi2_LR),  digits =   2 ), 
                                   intercept =   format (mod.gen $ fixef[ 1 ], digits=  3 ), 
                                   estimate =   format (mod.gen $ fixef[ 2 ], digits=  3 ), 
                                   df =   as.numeric (test $ basicLRT $ df), 
                                   Pvalue =   as.numeric ( format ( pchisq (Chi2_LRT_growth, df=  1 , lower.tail =  F), digits=  2 ))) 
   tab_stat_sucrose  = tab_stat 
    
    # Sorbitol:  
   mod.gen  =   fitme ((Total_Length_mm)  ~   Diet  +  ( 1   |  Repeat),  data =   subset (tab_xtrsugars_rev,Sugar ==  "Sorbitol" )) 
    shapiro.test ( residuals (mod.gen))     
  ## 
##  Shapiro-Wilk normality test
## 
## data:  residuals(mod.gen)
## W = 0.97561, p-value = 0.3359  
       bptest ((Total_Length_mm)  ~  Diet  +  ( 1   /  Repeat),  data =   subset (tab_xtrsugars_rev,Sugar ==  "Sorbitol" ))     
  ## 
##  studentized Breusch-Pagan test
## 
## data:  (Total_Length_mm) ~ Diet + (1/Repeat)
## BP = 0.129, df = 1, p-value = 0.7195  
      mod.gen1  =   fitme ((Total_Length_mm)  ~   1   +  ( 1   |  Repeat),  data =   subset (tab_xtrsugars_rev,Sugar ==  "Sorbitol" ))  
   test  =   anova (mod.gen, mod.gen1)  
   Chi2_LRT_growth  =   2  * (mod.gen $ APHLs[[ "p_v" ]] - mod.gen1 $ APHLs[[ "p_v" ]]) 
    
   tab_stat  =   data.frame ( Variable =   as.character ( paste ( "HS vs HY Sorbitol" )), 
                                   Rep =   nlevels (tab_lipids $ Repeat), 
                                   chi2_LR =   round ( as.numeric (test $ basicLRT $ chi2_LR),  digits =   2 ), 
                                   intercept =   format (mod.gen $ fixef[ 1 ], digits=  3 ), 
                                   estimate =   format (mod.gen $ fixef[ 2 ], digits=  3 ), 
                                   df =   as.numeric (test $ basicLRT $ df), 
                                   Pvalue =   as.numeric ( format ( pchisq (Chi2_LRT_growth, df=  1 , lower.tail =  F), digits=  2 ))) 
   tab_stat_Sorbitol = tab_stat 
    
    
    
   tab_stat  =   rbind (tab_stat_sucrose,tab_stat_Sorbitol) 
   tab_stat $ sig  =   ifelse (tab_stat $ Pvalue  <   0.05   &  tab_stat $ Pvalue  >   0.01 ,  "*" , 
                 ifelse (tab_stat $ Pvalue  <   0.01   &  tab_stat $ Pvalue  >   0.001 ,  "**" , 
                  ifelse (tab_stat $ Pvalue  <   0.001 ,  "***" ,  "" ))) 
   tab_stat $ Sugar  =   c ( "Sucrose" , "Sorbitol" ) 
   tab_stat $ Sugar =  as.factor (tab_stat $ Sugar) 
    
   tab_stat %>%  
      kable ( col.names =   c ( "Comparison" ,  "Replicates" ,  "Chi2" , "Intercept" , "Estimate" , "df"  , "p-value" , "Signif." , "Sugar" ), row.names =   FALSE )  %>%     add_header_above ( c ( "log(Total_Length_mm) ~  Diet + (1 | Repeat)"   =   9 )) %>%  
      kable_styling ( bootstrap_options =   c ( "striped" ,  "hover" ,  "condensed" ),  full_width =  F)    
 
 
 
 
 
log(Total_Length_mm) ~ Diet + (1 | Repeat)
 
 
 
 
 
Comparison
 
 
Replicates
 
 
Chi2
 
 
Intercept
 
 
Estimate
 
 
df
 
 
p-value
 
 
Signif.
 
 
Sugar
 
 
 
 
 
 
HS vs HY Sucrose
 
 
3
 
 
31.6
 
 
4.19
 
 
1.18
 
 
1
 
 
0.0e+00
 
 
***
 
 
Sucrose
 
 
 
 
HS vs HY Sorbitol
 
 
3
 
 
22.7
 
 
4.41
 
 
0.913
 
 
1
 
 
1.9e-06
 
 
***
 
 
Sorbitol
 
 
 
 
      Limits  =   c ( "HS" , "HY" ) 
   Labels =   c ( "HS" , "HY" ) 
    
   z  =   max (tab_xtrsugars_rev $ Total_Length_mm,  na.rm =   TRUE ) 
    
   Plot_Fig2S2C =  
      ggplot (tab_xtrsugars_rev,  aes ( x =  Diet,  y =  Total_Length_mm)) +   
      geom_violin ( aes ( fill =  Diet),  draw_quantiles =   c ( 0.25 ,  0.5 ,  0.75 ),  colour =   "black" ,  size =   0.2 , adjust =   0.8 )  +  
      geom_dotplot (  colour =   "black" ,  fill =   "white" ,  binaxis =   "y" ,  stackdir =   "center" ,  binwidth =  z  /   60 )  +   
      facet_grid (. ~ Sugar) +  
      geom_text ( data =  Sample_size,  mapping =   aes ( x =  Diet,  y =   1.8 ,  label =   paste ( "(" ,Sample_size, ")" , sep=  "" )), size=  3 ) +  
        geom_signif ( data =  tab_stat, aes ( xmin =   1 ,  xmax =   2 ,  annotations =   formatC ( paste ( "p=" ,Pvalue),  digits =   2 ),  y_position =   7.4 ),  textsize =   3 ,  vjust =   -  0.2 ,  manual =   TRUE ) +  
      scale_fill_manual ( limits= Limits, 
                        values= palette_diet_2) +  
      scale_x_discrete ( "" , 
                       limits= Limits, 
                       labels= Labels) +  
      scale_y_continuous ( "Midgut length (mm)" , 
                         limits=  c ( 1.7 , 7.5 ), 
                         breaks=  seq ( 2 , 8 , by=  1 )) +  
       stat_summary ( fun =  mean,  geom =   "point" ,  size =   3 ,  shape =   18 ,  colour =   "black" ,  aes ( group =  Repeat))  +  
                         stat_summary ( fun =  mean,  geom =   "point" ,  size =   2 ,  shape =   18 ,  aes ( group =  Repeat,  colour =  Repeat))  +  
                         scale_color_manual ( values =  palette_mean)  +  
      theme ( panel.background =   element_blank (), 
            panel.grid.major.y =   element_line ( colour =   grey ( 0.45 ),  linetype =   "dashed" ,  size =   0.2 ), 
            axis.title.x =   element_blank (), 
            axis.title.y =   element_text ( size= Smallfont, colour=  "black" ),  
            axis.line.x =   element_line ( colour=  "black" , size=  0.75 ), 
            axis.line.y =   element_line ( colour=  "black" , size=  0.75 ), 
            axis.ticks.x =   element_line ( size =   0.75 ), 
            axis.ticks.y =   element_line ( size =   0.75 ), 
            axis.text.x =   element_text ( size= Smallfont, colour=  "black" ),  
            axis.text.y =   element_text ( size= Smallfont, colour=  "black" ), 
            plot.margin =   unit ( c ( 0 , 0 , 0 , 0.5 ),  "cm" ), 
            legend.direction =   "vertical" ,  
            legend.box =   "horizontal" , 
            legend.position =   "none" , 
            legend.key.height =   unit ( 0.4 ,  "cm" ), 
            legend.key.width=   unit ( 0.6 ,  "cm" ), 
            legend.title =   element_text ( face=  "italic" , size= Smallfont),  
            legend.key =   element_rect ( colour =   'white' ,  fill =   "white" ,  linetype=  'dashed' ), 
            legend.text =   element_text ( size= SuperSmallfont), 
            legend.background =   element_rect ( fill=  NA ), 
            strip.text.x =   element_text ( size =  Smallfont,  colour =   "black" ,  margin =   margin ( t =   2 ,  r =   0 ,  b =   2 ,  l =   0 )), 
            strip.text.y =   element_text ( size =  Smallfont,  colour =   "black" ,  margin =   margin ( t =   2 ,  r =   0 ,  b =   2 ,  l =   0 )), 
            strip.background =   element_rect ( fill=  NA ,  colour=  "black" ), 
            strip.placement=  "outside" ) 
    
   Plot_Fig2S2C    
   
 ##Export Figure 2S2 
 
 
 
 
  3  Figure 3. Diet composition affects both cell number and enterocyte size in the midgut 
 
  3.1  Figure 3 - main 
 
  3.1.1  Figure 3A - B 
 
 Representative pictures of midguts from flies kept on HS (A) or HY (B) diet. Green arrows indicate intestinal stem cells (ISCs), marked only by GFP (green), red arrows mark enteroblasts (EBs), marked by GFP and GBE Su(H)-lacZ (red), and white arrow indicate enteroendocrine (EE) cells, marked with anti-Prospero antibody (white). All nuclei are stained with DAPI (blue).  Complete graphical annotation can be found in manuscript figures  
 
 
 
   
 
 
   
 
 
 
 
  3.1.2  Figure 3C 
 
 Quantification of total cell numbers in the posterior midgut (R4) for HS and HY 
 
      Tab_cellnumber  =   
     d[[ "3C" ]] %>%  
         mutate ( across ( c (Diet,Line,Day,Repeat,GutNumber,Region),as.factor)) %>%  
         mutate ( across ( c (ISC.AL,EB.AL,EE.AL,EC.AL),round, 0 )) 
    
    ###Stats  
    #### ISC  
   mod.gen  =   fitme ( log (ISC.AL)  ~   Diet  +  ( 1   |  Repeat),  data =  Tab_cellnumber) 
    shapiro.test ( residuals (mod.gen))     
  ## 
##  Shapiro-Wilk normality test
## 
## data:  residuals(mod.gen)
## W = 0.96619, p-value = 0.01821  
       bptest ( log (ISC.AL)  ~  Diet  +  ( 1   /  Repeat),  data =  Tab_cellnumber)     
  ## 
##  studentized Breusch-Pagan test
## 
## data:  log(ISC.AL) ~ Diet + (1/Repeat)
## BP = 0.95685, df = 1, p-value = 0.328  
      mod.gen1  =   fitme ( log (ISC.AL)  ~    1   +  ( 1   |  Repeat),  data =  Tab_cellnumber) 
   test  =   anova (mod.gen, mod.gen1)  
   Chi2_LRT_growth  =   2  * (mod.gen $ APHLs[[ "p_v" ]] - mod.gen1 $ APHLs[[ "p_v" ]]) 
    
   tab_stat  =   data.frame ( Comparison =   as.character ( paste ( "HS vs HY ISC" )), 
      Cell_type =   as.character ( paste ( "ISC.AL" )), 
                                   Rep =   nlevels (Tab_cellnumber $ Repeat), 
                                   chi2_LR =   round ( as.numeric (test $ basicLRT $ chi2_LR),  digits =   2 ), 
                                   intercept =   format (mod.gen $ fixef[ 1 ], digits=  3 ), 
                                   estimate =   format (mod.gen $ fixef[ 2 ], digits=  3 ), 
                                   df =   as.numeric (test $ basicLRT $ df), 
                                   Pvalue =   as.numeric ( format ( pchisq (Chi2_LRT_growth, df=  1 , lower.tail =  F), digits=  2 ))) 
   tab_stat_ISC = tab_stat 
    
    #### EB  
   mod.gen  =   fitme ( log (EB.AL)  ~   Diet  +  ( 1   |  Repeat), data =  Tab_cellnumber) 
    shapiro.test ( residuals (mod.gen))     
  ## 
##  Shapiro-Wilk normality test
## 
## data:  residuals(mod.gen)
## W = 0.98307, p-value = 0.2856  
       bptest ( log (EB.AL)  ~  Diet  +  ( 1   /  Repeat), data =  Tab_cellnumber)     
  ## 
##  studentized Breusch-Pagan test
## 
## data:  log(EB.AL) ~ Diet + (1/Repeat)
## BP = 0.067845, df = 1, p-value = 0.7945  
      mod.gen1  =   fitme ( log (EB.AL)  ~   1   +  ( 1   |  Repeat),  data =  Tab_cellnumber)  
   test  =   anova (mod.gen, mod.gen1)  
   Chi2_LRT_growth  =   2  * (mod.gen $ APHLs[[ "p_v" ]] - mod.gen1 $ APHLs[[ "p_v" ]]) 
    
   tab_stat  =   data.frame ( Comparison =   as.character ( paste ( "HS vs HY EB" )), 
                          Cell_type =   as.character ( paste ( "EB.AL" )), 
                                   Rep =   nlevels (Tab_cellnumber $ Repeat), 
                                   chi2_LR =   round ( as.numeric (test $ basicLRT $ chi2_LR),  digits =   2 ), 
                                   intercept =   format (mod.gen $ fixef[ 1 ], digits=  3 ), 
                                   estimate =   format (mod.gen $ fixef[ 2 ], digits=  3 ), 
                                   df =   as.numeric (test $ basicLRT $ df), 
                                   Pvalue =   as.numeric ( format ( pchisq (Chi2_LRT_growth, df=  1 , lower.tail =  F), digits=  2 ))) 
   tab_stat_EB = tab_stat 
    
    #### EC  
   mod.gen  =   fitme ( log (EC.AL)  ~   Diet  +  ( 1   |  Repeat),  data =  Tab_cellnumber) 
    shapiro.test ( residuals (mod.gen))     
  ## 
##  Shapiro-Wilk normality test
## 
## data:  residuals(mod.gen)
## W = 0.98161, p-value = 0.2211  
       bptest ( log (EC.AL)  ~  Diet  +  ( 1   /  Repeat),  data =  Tab_cellnumber)     
  ## 
##  studentized Breusch-Pagan test
## 
## data:  log(EC.AL) ~ Diet + (1/Repeat)
## BP = 1.7873, df = 1, p-value = 0.1813  
      mod.gen1  =   fitme ( log (EC.AL)  ~   1   +  ( 1   |  Repeat),  data =  Tab_cellnumber)  
   test  =   anova (mod.gen, mod.gen1)  
   test    
  ##      chi2_LR df      p_value
## p_v 37.74043  1 8.081226e-10  
      Chi2_LRT_growth  =   2  * (mod.gen $ APHLs[[ "p_v" ]] - mod.gen1 $ APHLs[[ "p_v" ]]) 
    
   tab_stat  =   data.frame ( Comparison =   as.character ( paste ( "HS vs HY EC" )), 
      Cell_type =   as.character ( paste ( "EC.AL" )), 
                                   Rep =   nlevels (Tab_cellnumber $ Repeat), 
                                   chi2_LR =   round ( as.numeric (test $ basicLRT $ chi2_LR),  digits =   2 ), 
                                   intercept =   format (mod.gen $ fixef[ 1 ], digits=  3 ), 
                                   estimate =   format (mod.gen $ fixef[ 2 ], digits=  3 ), 
                                   df =   as.numeric (test $ basicLRT $ df), 
                                   Pvalue =   as.numeric ( format ( pchisq (Chi2_LRT_growth, df=  1 , lower.tail =  F), digits=  2 ))) 
   tab_stat_EC = tab_stat 
    
    #### EE  
   mod.gen  =   fitme ( log (EE.AL)  ~   Diet  +  ( 1   |  Repeat),  data =  Tab_cellnumber) 
    shapiro.test ( residuals (mod.gen))     
  ## 
##  Shapiro-Wilk normality test
## 
## data:  residuals(mod.gen)
## W = 0.97566, p-value = 0.08254  
       bptest ( log (EE.AL)  ~  Diet  +  ( 1   /  Repeat),  data =  Tab_cellnumber)     
  ## 
##  studentized Breusch-Pagan test
## 
## data:  log(EE.AL) ~ Diet + (1/Repeat)
## BP = 3.8518, df = 1, p-value = 0.04969  
      mod.gen1  =   fitme ( log (EE.AL)  ~   1   +  ( 1   |  Repeat),  data =  Tab_cellnumber)  
   test  =   anova (mod.gen, mod.gen1)  
   Chi2_LRT_growth  =   2  * (mod.gen $ APHLs[[ "p_v" ]] - mod.gen1 $ APHLs[[ "p_v" ]]) 
    
   tab_stat  =   data.frame ( Comparison =   as.character ( paste ( "HS vs HY EE" )), 
                          Cell_type =   as.character ( paste ( "EE.AL" )), 
                                   Rep =   nlevels (Tab_cellnumber $ Repeat), 
                                   chi2_LR =   round ( as.numeric (test $ basicLRT $ chi2_LR),  digits =   2 ), 
                                   intercept =   format (mod.gen $ fixef[ 1 ], digits=  3 ), 
                                   estimate =   format (mod.gen $ fixef[ 2 ], digits=  3 ), 
                                   df =   as.numeric (test $ basicLRT $ df), 
                                   Pvalue =   as.numeric ( format ( pchisq (Chi2_LRT_growth, df=  1 , lower.tail =  F), digits=  2 ))) 
   tab_stat_EE = tab_stat 
    
   tab_stat =  rbind (tab_stat_ISC,tab_stat_EB,tab_stat_EC,tab_stat_EE) 
   tab_stat $ sig  =   ifelse (tab_stat $ Pvalue  <   0.05   &  tab_stat $ Pvalue  >   0.01 ,  "*" , 
                 ifelse (tab_stat $ Pvalue  <   0.01   &  tab_stat $ Pvalue  >   0.001 ,  "**" , 
                  ifelse (tab_stat $ Pvalue  <   0.001 ,  "***" ,  "" ))) 
    
   tab_stat %>%  
      kable ( col.names =   c ( "Comparison" ,  "Cell type" ,  "Replicates" ,  "Chi2" , "Intercept" , "Estimate" , "df"  , "p-value" , "Signif." ), row.names =   FALSE )  %>%     add_header_above ( c ( "log(Cell number) ~  Diet + (1 | Repeat)"   =   9 )) %>%  
      kable_styling ( bootstrap_options =   c ( "striped" ,  "hover" ,  "condensed" ),  full_width =  F)    
 
 
 
 
 
log(Cell number) ~ Diet + (1 | Repeat)
 
 
 
 
 
Comparison
 
 
Cell type
 
 
Replicates
 
 
Chi2
 
 
Intercept
 
 
Estimate
 
 
df
 
 
p-value
 
 
Signif.
 
 
 
 
 
 
HS vs HY ISC
 
 
ISC.AL
 
 
6
 
 
16.77
 
 
6.25
 
 
0.425
 
 
1
 
 
4.2e-05
 
 
***
 
 
 
 
HS vs HY EB
 
 
EB.AL
 
 
6
 
 
7.94
 
 
5.34
 
 
0.39
 
 
1
 
 
4.8e-03
 
 
**
 
 
 
 
HS vs HY EC
 
 
EC.AL
 
 
6
 
 
37.74
 
 
7.62
 
 
0.374
 
 
1
 
 
0.0e+00
 
 
***
 
 
 
 
HS vs HY EE
 
 
EE.AL
 
 
6
 
 
14.78
 
 
5.16
 
 
0.505
 
 
1
 
 
1.2e-04
 
 
***
 
 
 
 
       ### Plot  
   Limits  =   c ( "HS" ,  "HY" ) 
    
   Tab_cellnumber_gather =  
     Tab_cellnumber  %>%  
      select (Diet,Line,Day,Repeat,GutNumber,Region,EB.AL,ISC.AL,EE.AL,EC.AL) %>%  
      gather (key, value,  -  c (Diet,Line,Day,Repeat,GutNumber,Region))  %>%  
     dplyr ::  rename ( Cell_type =  key, 
             Cell_number =  value)  %>%  
      mutate_if (is.character,as.factor) 
    
   Sample_size =  
      subset (Tab_cellnumber_gather, !  is.na (Day)) %>%  
      group_by (Diet,Cell_type) %>%  
      summarise ( Sample_size=  n (), 
                max=  max (Cell_number, na.rm= T)) 
    
   tmp =  subset (Sample_size,Diet ==  "HY" ) 
   tab_stat  =   
      left_join (tab_stat,tmp) 
    
   Treatment.status  =   c ( "ISC" ,  "EB" , "EC" , "EE" ) 
    names (Treatment.status)  =   c ( "ISC.AL" ,  "EB.AL" , "EC.AL" , "EE.AL" ) 
    
    
   Plot_Fig3C =  
      ggplot (Tab_cellnumber_gather,  aes ( x =  Diet,  y =  Cell_number /  100  *  2 )) +   
      geom_violin ( aes ( fill =  Diet),  draw_quantiles =   c ( 0.25 ,  0.5 ,  0.75 ),  colour =   "black" ,  size =   0.2 , adjust =   0.8 )  +  
      geom_dotplot (  colour =   "black" ,  fill =   "white" ,  binaxis =   "y" ,  stackdir =   "center" )  +   
      facet_wrap (. ~ Cell_type, scale=  "free_y" , labeller=  labeller ( Cell_type= Treatment.status)) +  
      geom_blank ( data= tab_stat,  aes ( y =  max /  100  *  2.5 )) +  
      geom_signif ( data =  tab_stat,  aes ( xmin =   1 ,  xmax =   2 ,  annotations =   formatC ( paste ( "p=" ,Pvalue),  digits =   2 ),  y_position =  max /  100  *  2.2 ,),  textsize =   3 ,  vjust =   -  0.2 ,  manual =   TRUE ) +  
      scale_fill_manual ( limits= Limits, 
                        values= palette_diet_2) +  
      scale_x_discrete ( "" , 
                       limits=  c ( "HS" ,  "HY" ), 
                       labels=  c ( "HS (n = 46)" ,  "HY (n = 55)" )) +  
      scale_y_continuous ( expression ( paste ( "Cell number in posterior midgut (x" , 10  ^  2 , ")" , sep=  "" ))) +  
      stat_summary ( fun =  mean,  geom =   "point" ,  size =   2.5 ,  shape =   18 ,  colour =   "black" ,  aes ( group =  Repeat))  +  
                         stat_summary ( fun =  mean,  geom =   "point" ,  size =   1.5 ,  shape =   18 ,  aes ( group =  Repeat,  colour =  Repeat))  +  
                         scale_color_manual ( values =  palette_mean)  +  
      theme ( 
        panel.grid.major.y =   element_line ( colour =   grey ( 0.45 ),  linetype =   "dashed" ,  size =   0.2 ), 
        panel.background =   element_blank (), 
        axis.title.x =   element_text ( size= Smallfont, colour=  "black" ), 
        axis.title.y =   element_text ( size= Smallfont, colour=  "black" ),  
        axis.line.x =   element_line ( colour=  "black" , size=  0.75 ), 
        axis.line.y =   element_line ( colour=  "black" , size=  0.75 ), 
        axis.ticks.x =   element_line ( size =   0.75 ), 
        axis.ticks.y =   element_line ( size =   0.75 ), 
        axis.text.x =   element_text ( size= Smallfont, colour=  "black" , angle=  30 , hjust=  1 ), 
        axis.text.y =   element_text ( size= Smallfont, colour=  "black" ), 
        plot.margin =   unit (Margin,  "cm" ), 
        legend.direction =   "vertical" ,  
        legend.box =   "horizontal" , 
        legend.position =   "none" , 
        legend.key.height =   unit ( 0.4 ,  "cm" ), 
        legend.key.width=   unit ( 0.6 ,  "cm" ), 
        legend.title =   element_text ( face=  "italic" , size= Smallfont),  
        legend.key =   element_rect ( colour =   'white' ,  fill =   "white" ,  linetype=  'dashed' ), 
        legend.text =   element_text ( size= SuperSmallfont), 
        legend.background =   element_rect ( fill=  NA ), 
        strip.text.x =   element_text ( size =  Smallfont,  colour =   "black" ,  margin =   margin ( t =   1 ,  r =   0 ,  b =   1 ,  l =   0 )), 
        strip.text.y =   element_text ( size =  Smallfont,  colour =   "black" ,  margin =   margin ( t =   1 ,  r =   0 ,  b =   1 ,  l =   0 )), 
        strip.background =   element_rect ( fill=  NA ,  colour=  "black" ), 
        strip.placement=  "outside" ) 
    
   Plot_Fig3C    
   
 
 
  3.1.3  Figure 3D 
 
 HS and HY diets do not affect the relative proportion of cell types in the midgut (error is standard error of the mean). 
 
      tab_prop_cell_type  =   
     d[[ "3C" ]] %>%  
     dplyr ::  select (Diet, Line, Day, Repeat, GutNumber, Region  |   ends_with ( ".AL" )  &   !  starts_with ( "ESG" )) %>%  
      mutate_if (is.character,as.factor) %>%   
      drop_na () %>%  
      mutate ( across ( c (ISC.AL,EB.AL,EE.AL,EC.AL),round, 0 )) %>%  
      mutate ( Total_cell= ISC.AL + EB.AL + EE.AL + EC.AL, 
             proportion_ISC= ISC.AL / Total_cell *  100 , 
             proportion_EB= EB.AL / Total_cell *  100 , 
             proportion_EE= EE.AL / Total_cell *  100 , 
             proportion_EC= EC.AL / Total_cell *  100 ) %>%  
     dplyr ::  select (Diet, Line, Day, Repeat, GutNumber, Region  |   starts_with ( "proportion" )) %>%  
      gather (key, value,  -!  starts_with ( "proportion" ) ) %>%  
     dplyr ::  rename ( Cell_type =  key, 
             Cell_proportion =  value)  %>%  
      mutate_if (is.character,as.factor)  %>%  
      group_by (Diet,Cell_type) %>%  
      summarise ( mean_proportion=  mean (Cell_proportion,  na.rm= T), 
                se_proportion=  se (Cell_proportion))  %>%  
       as.data.frame () 
    
    for  (i  in   1  :  length (tab_prop_cell_type $ se_proportion)){ 
       tab_prop_cell_type $ se_proportionGraphPlus[i]  =  tab_prop_cell_type $ mean_proportion[i] + tab_prop_cell_type $ se_proportion[i] 
       tab_prop_cell_type $ se_proportionGraphMinus[i]  =  tab_prop_cell_type $ mean_proportion[i] - tab_prop_cell_type $ se_proportion[i] 
   } 
    
    for  (i  in   1  :  length (tab_prop_cell_type $ se_proportion)){ 
      if (tab_prop_cell_type $ Cell_type[i] ==  "proportion_EB" ){ 
       tab_prop_cell_type $ se_proportionGraphPlus[i]  =  tab_prop_cell_type $ mean_proportion[i] + tab_prop_cell_type $ se_proportion[i] 
        
       tab_prop_cell_type $ se_proportionGraphMinus[i]  =  tab_prop_cell_type $ mean_proportion[i] - tab_prop_cell_type $ se_proportion[i] 
        
     } else { 
        if (tab_prop_cell_type $ Cell_type[i] ==  "proportion_EC" ){ 
          
         tab_prop_cell_type $ se_proportionGraphPlus[i]  =  tab_prop_cell_type $ mean_proportion[i -1 ] + tab_prop_cell_type $ mean_proportion[i] + tab_prop_cell_type $ se_proportion[i] 
          
         tab_prop_cell_type $ se_proportionGraphMinus[i]  =  tab_prop_cell_type $ mean_proportion[i -1 ] + tab_prop_cell_type $ mean_proportion[i] +- tab_prop_cell_type $ se_proportion[i] 
          
       } else { 
          if (tab_prop_cell_type $ Cell_type[i] ==  "proportion_EE" ){ 
            
           tab_prop_cell_type $ se_proportionGraphPlus[i]  = tab_prop_cell_type $ mean_proportion[i -1 ] +  tab_prop_cell_type $ mean_proportion[i -2 ] + tab_prop_cell_type $ mean_proportion[i] + tab_prop_cell_type $ se_proportion[i] 
            
           tab_prop_cell_type $ se_proportionGraphMinus[i]  = tab_prop_cell_type $ mean_proportion[i -1 ] +  tab_prop_cell_type $ mean_proportion[i -2 ] + tab_prop_cell_type $ mean_proportion[i] - tab_prop_cell_type $ se_proportion[i] 
            
         } else { 
           tab_prop_cell_type $ se_proportionGraphPlus[i]  = tab_prop_cell_type $ mean_proportion[i -1 ] + tab_prop_cell_type $ mean_proportion[i -2 ] +  tab_prop_cell_type $ mean_proportion[i -3 ] + tab_prop_cell_type $ mean_proportion[i] + tab_prop_cell_type $ se_proportion[i] 
            
           tab_prop_cell_type $ se_proportionGraphMinus[i]  = tab_prop_cell_type $ mean_proportion[i -1 ] + tab_prop_cell_type $ mean_proportion[i -2 ] +  tab_prop_cell_type $ mean_proportion[i -3 ] + tab_prop_cell_type $ mean_proportion[i] - tab_prop_cell_type $ se_proportion[i] 
         } 
       } 
     } 
   } 
    
   tab_prop_cell_type $ Cell_type  =  factor (tab_prop_cell_type $ Cell_type, levels =   c ( "proportion_ISC" , "proportion_EE" , "proportion_EC" , "proportion_EB" )) 
    
   Plot_Fig3D  =  
    ggplot (tab_prop_cell_type,  aes ( x= Diet,  y= mean_proportion)) +   
      geom_bar ( stat=  "identity" , aes ( fill= Cell_type), color=  "black" , width= . 90 ) +  
      geom_errorbar ( aes ( ymin=  se_proportionGraphMinus,  ymax=  se_proportionGraphPlus), width=  0.25 ) +  
      scale_fill_manual ( name =   "Cell types" ,  
                        values=  c ( "#1fd511" , "#ffffff" ,  "#5869d5" , "#fe0000" ), 
                        labels =   c ( "ISC" ,  "EE" ,  "EC" , "EB" )) +  
       scale_y_continuous ( "Proportion of cells (% \u00B1se)" , 
                          limits=  c ( 0 , 101 ), 
                          breaks=  seq ( 0 , 100 , by=  25 )) +  
      theme ( 
        panel.grid.major.y =   element_line ( colour =   grey ( 0.45 ),  linetype =   "dashed" ,  size =   0.2 ), 
        panel.background =   element_blank (), 
        axis.title.x =   element_blank (), 
        axis.title.y =   element_text ( size= Smallfont, colour=  "black" ,  hjust =   0.1  ),  
        axis.line.x =   element_line ( colour=  "black" , size=  0.75 ), 
        axis.line.y =   element_line ( colour=  "black" , size=  0.75 ), 
        axis.ticks.x =   element_line ( size =   0.75 ), 
        axis.ticks.y =   element_line ( size =   0.75 ), 
        axis.text.x =   element_text ( size= Smallfont, colour=  "black" ), 
        axis.text.y =   element_text ( size= Smallfont, colour=  "black" ), 
        plot.margin =   unit (Margin,  "cm" ), 
        legend.direction =   "vertical" ,  
        legend.box =   "horizontal" , 
        legend.position =   "bottom" , 
        legend.key.height =   unit ( 0.4 ,  "cm" ), 
        legend.key.width=   unit ( 0.6 ,  "cm" ), 
        legend.title =   element_text ( face=  "italic" , size= Smallfont),  
        legend.key =   element_rect ( colour =   'white' ,  fill =   "white" ,  linetype=  'dashed' ), 
        legend.text =   element_text ( size= Smallfont), 
        legend.background =   element_rect ( fill=  NA ), 
        strip.text.x =   element_text ( size = Smallfont,  colour =   "black" , face=  "italic" ), 
        strip.text.y =   element_text ( size = Smallfont,  colour =   "black" , face=  "italic" ), 
        strip.background =   element_rect ( fill=  NA ,  colour=  "black" ), 
        strip.placement=  "outside" ) 
    
    
   Plot_Fig3D    
   
 
 
  3.1.4  Figure 3E - F 
 
  Diet affects enterocyte size . Representative picture of midguts stained with anti-Mesh antibody on HS (D, left) vs HY (E, right) diet.  Complete graphical annotation can be found in manuscript figures  
 
 
 
   
 
 
   
 
 
 
 
  3.1.5  Figure 3G 
 
 Quantification of EC size of individuals on HS or HY diet for 5 days confirms an increase in cell size on HY diet. 
 
      tab_cell_area  =   
     d[[ "3G" ]] %>%  
      mutate_if (is.character,as.factor) %>%  
      mutate_if (is.integer,as.factor) 
    
   Sample_size =  
     tab_cell_area %>%  
      group_by (Diet) %>%  
      summarise ( Sample_size=  n ()) 
    
    ###Stats  
    
   mod.gen  =   fitme ( log (Area)  ~   Diet  +  ( 1  | Repeat), data = tab_cell_area) 
    shapiro.test ( residuals (mod.gen))     
  ## 
##  Shapiro-Wilk normality test
## 
## data:  residuals(mod.gen)
## W = 0.99873, p-value = 0.5532  
       bptest ( log (Area)  ~   Diet  +  ( 1  / Repeat), data = tab_cell_area)    
  ## 
##  studentized Breusch-Pagan test
## 
## data:  log(Area) ~ Diet + (1/Repeat)
## BP = 14.735, df = 1, p-value = 0.0001237  
      mod.gen1  =   fitme ( log (Area)  ~    1   +  ( 1  | Repeat), data = tab_cell_area) 
   test  =   anova (mod.gen, mod.gen1)  
   Chi2_LRT_growth  =   2  * (mod.gen $ APHLs[[ "p_v" ]] - mod.gen1 $ APHLs[[ "p_v" ]]) 
    
   tab_stat  =   data.frame ( Variable =   as.character ( paste ( "HS vs HY" )), 
                                   Rep =   nlevels (tab_cell_area $ Repeat), 
                                   chi2_LR =   round ( as.numeric (test $ basicLRT $ chi2_LR),  digits =   2 ), 
                                   intercept =   format (mod.gen $ fixef[ 1 ], digits=  3 ), 
                                   estimate =   format (mod.gen $ fixef[ 2 ], digits=  3 ), 
                                   df =   as.numeric (test $ basicLRT $ df), 
                                   Pvalue =   as.numeric ( format ( pchisq (Chi2_LRT_growth, df=  1 , lower.tail =  F), digits=  2 ))) 
   tab_stat $ sig  =   ifelse (tab_stat $ Pvalue  <   0.05   &  tab_stat $ Pvalue  >   0.01 ,  "*" , 
                 ifelse (tab_stat $ Pvalue  <   0.01   &  tab_stat $ Pvalue  >   0.001 ,  "**" , 
                  ifelse (tab_stat $ Pvalue  <   0.001 ,  "***" ,  "" ))) 
    
   tab_stat %>%  
      kable ( col.names =   c ( "Comparison" ,  "Replicates" ,  "Chi2" , "Intercept" , "Estimate" , "df"  , "p-value" , "Signif." ), row.names =   FALSE )  %>%     add_header_above ( c ( "log(Cell area) ~  Diet + (1 | Repeat)"   =   8 )) %>%  
      kable_styling ( bootstrap_options =   c ( "striped" ,  "hover" ,  "condensed" ),  full_width =  F)    
 
 
 
 
 
log(Cell area) ~ Diet + (1 | Repeat)
 
 
 
 
 
Comparison
 
 
Replicates
 
 
Chi2
 
 
Intercept
 
 
Estimate
 
 
df
 
 
p-value
 
 
Signif.
 
 
 
 
 
 
HS vs HY
 
 
3
 
 
842.09
 
 
4.8
 
 
0.796
 
 
1
 
 
0
 
 
***
 
 
 
 
       ### Plot  
    
   z  =   max (tab_cell_area $ Area /  1000 ,  na.rm =   TRUE ) 
   Plot_Fig3G =  
      ggplot (tab_cell_area,  aes ( x =  Diet,  y =  Area /  1000 )) +   
      geom_violin ( aes ( fill =  Diet),  draw_quantiles =   c ( 0.25 ,  0.5 ,  0.75 ),  colour =   "black" ,  size =   0.2 )  +  
      geom_dotplot (  colour =   "black" ,  fill =   "white" ,  binaxis =   "y" ,  stackdir =   "center" ,  binwidth =  z /  140 )  +   
      geom_text ( data =  Sample_size,  mapping =   aes ( x =  Diet,  y =   -  0.01 ,  label =   paste ( "(" ,Sample_size, ")" , sep=  "" )), size=  3 ) +  
     # geom_text(data = tab_stat, mapping = aes(x = 1.5, y = 0.62, label = paste("p=",format(Pvalue,digits=3))),size=3)+  
        geom_signif ( annotation =   formatC ( paste ( "p=" ,tab_stat $ Pvalue),  digits =   2 ),  textsize =   3 ,  y_position =   0.86 ,  xmin =   1 ,  xmax =   2 ,  tip_length =   c ( 0.02 ,  0.02 ),  vjust =   -  0.2 ) +  
    
      scale_fill_manual ( limits=  c ( "HS" ,  "HY" ), 
                        values=  palette_diet_2 ) +  
      scale_x_discrete ( "" , 
                       limits=  c ( "HS" ,  "HY" ), 
                       labels=  c ( "HS" ,  "HY" )) +  
      scale_y_continuous ( expression ( paste ( "EC area (10"  ^  3 ,  "mm"  ^  2 , ")" , sep=  "" )), 
                         limits=  c ( -  0.01 , 0.9 ), 
                         breaks=  seq ( 0 , 0.8 , by=  0.1 )) +  
      stat_summary ( fun =  mean,  geom =   "point" ,  size =   3 ,  shape =   18 ,  colour =   "black" ,  aes ( group =  Repeat))  +  
                         stat_summary ( fun =  mean,  geom =   "point" ,  size =   2 ,  shape =   18 ,  aes ( group =  Repeat,  colour =  Repeat))  +  
                         scale_color_manual ( values =  palette_mean)  +  
      theme ( 
        panel.grid.major.y =   element_line ( colour =   grey ( 0.45 ),  linetype =   "dashed" ,  size =   0.2 ), 
        panel.background =   element_blank (), 
        axis.title.x =   element_text ( size= Smallfont, colour=  "black" ), 
        axis.title.y =   element_text ( size= Smallfont, colour=  "black" ),  
        axis.line.x =   element_line ( colour=  "black" , size=  0.75 ), 
        axis.line.y =   element_line ( colour=  "black" , size=  0.75 ), 
        axis.ticks.x =   element_line ( size =   0.75 ), 
        axis.ticks.y =   element_line ( size =   0.75 ), 
        axis.text.x =   element_text ( size= Smallfont, colour=  "black" ), 
        axis.text.y =   element_text ( size= Smallfont, colour=  "black" ), 
        plot.margin =   unit (Margin,  "cm" ), 
        legend.direction =   "vertical" ,  
        legend.box =   "horizontal" , 
        legend.position =   "none" , 
        legend.key.height =   unit ( 0.4 ,  "cm" ), 
        legend.key.width=   unit ( 0.6 ,  "cm" ), 
        legend.title =   element_text ( face=  "italic" , size= Smallfont),  
        legend.key =   element_rect ( colour =   'white' ,  fill =   "white" ,  linetype=  'dashed' ), 
        legend.text =   element_text ( size= SuperSmallfont), 
        legend.background =   element_rect ( fill=  NA )) 
    
   Plot_Fig3G    
   
 ##Export Figure 3 
 
 
 
  3.2  Figure 3 - figure supplement 1 
 
  3.2.1  Figure 3S1A 
 
 ECs are more densely packed on HS diet than on HY diet. 
 
      tab_ECarea  =   
     d[[ "3C" ]] %>%  
      select (Diet, Line, Day, Repeat, GutNumber, Region,EC.A) %>%  
      mutate_if (is.character,as.factor) %>%   
      mutate_if (is.integer,as.factor) %>%   
     dplyr ::  rename ( EC_density= EC.A) %>%   
      mutate ( EC_density_mm= EC_density *  1000 ) %>%   
      drop_na () 
    
   Sample_size =  
     tab_ECarea %>%  
      group_by (Diet) %>%  
      summarise ( Sample_size=  n ()) 
    
    ###Stats  
    
   mod.gen  =   fitme (EC_density_mm  ~   Diet  +  ( 1   |  Repeat),  data =  tab_ECarea) 
    shapiro.test ( residuals (mod.gen))     
  ## 
##  Shapiro-Wilk normality test
## 
## data:  residuals(mod.gen)
## W = 0.98248, p-value = 0.2538  
       bptest (EC_density_mm  ~  Diet  +  ( 1   /  Repeat),  data =  tab_ECarea)     
  ## 
##  studentized Breusch-Pagan test
## 
## data:  EC_density_mm ~ Diet + (1/Repeat)
## BP = 0.027933, df = 1, p-value = 0.8673  
      mod.gen1  =   fitme (EC_density_mm  ~   1   +  ( 1   |  Repeat),  data =  tab_ECarea)  
   test  =   anova (mod.gen, mod.gen1) 
   Chi2_LRT_growth  =   2  * (mod.gen $ APHLs[[ "p_v" ]] - mod.gen1 $ APHLs[[ "p_v" ]]) 
    
    #Now we make a tab with the results  
   tab_stat  =   data.frame ( Variable =   as.character ( paste ( "HS vs HY" )), 
                                   Rep =   nlevels (tab_ECarea $ Repeat), 
                                   chi2_LR =   round ( as.numeric (test $ basicLRT $ chi2_LR),  digits =   2 ), 
                                   intercept =   format (mod.gen $ fixef[ 1 ], digits=  3 ), 
                                   estimate =   format (mod.gen $ fixef[ 2 ], digits=  3 ), 
                                   df =   as.numeric (test $ basicLRT $ df), 
                                   Pvalue =   as.numeric ( format ( pchisq (Chi2_LRT_growth, df=  1 , lower.tail =  F), digits=  2 ))) 
   tab_stat $ sig  =   ifelse (tab_stat $ Pvalue  <   0.05   &  tab_stat $ Pvalue  >   0.01 ,  "*" , 
                 ifelse (tab_stat $ Pvalue  <   0.01   &  tab_stat $ Pvalue  >   0.001 ,  "**" , 
                  ifelse (tab_stat $ Pvalue  <   0.001 ,  "***" ,  "" ))) 
    
   tab_stat %>%  
      kable ( col.names =   c ( "Comparison" ,  "Replicates" ,  "Chi2" , "Intercept" , "Estimate" , "df"  , "p-value" , "Signif." ), row.names =   FALSE )  %>%     add_header_above ( c ( "EC density ~  Diet + (1 | Repeat)"   =   8 )) %>%  
      kable_styling ( bootstrap_options =   c ( "striped" ,  "hover" ,  "condensed" ),  full_width =  F)    
 
 
 
 
 
EC density ~ Diet + (1 | Repeat)
 
 
 
 
 
Comparison
 
 
Replicates
 
 
Chi2
 
 
Intercept
 
 
Estimate
 
 
df
 
 
p-value
 
 
Signif.
 
 
 
 
 
 
HS vs HY
 
 
6
 
 
20.79
 
 
5.98
 
 
-1.18
 
 
1
 
 
5.1e-06
 
 
***
 
 
 
 
      Limits =   c ( "HS" , "HY" ) 
    
   z =  max (tab_ECarea $ EC_density_mm,  na.rm =   TRUE ) 
   Plot_Fig3S1A  =  
    ggplot (tab_ECarea,  aes ( x= Diet,  y= EC_density_mm)) +   
     geom_violin ( aes ( fill =  Diet),  draw_quantiles =   c ( 0.25 ,  0.5 ,  0.75 ),  colour =   "black" ,  size =   0.2 , adjust =   0.8 )  +  
      geom_dotplot (  colour =   "black" ,  fill =   "white" ,  binaxis =   "y" ,  stackdir =   "center" ,  binwidth =  z /  60 )  +   
      geom_text ( data =  Sample_size,  mapping =   aes ( x =  Diet,  y =   1.8 ,  label =   paste ( "(" ,Sample_size, ")" , sep=  "" )), size=  3 ) +  
        geom_signif ( annotation =   formatC ( paste ( "p=" ,tab_stat $ Pvalue),  digits =   2 ),  textsize =   3 ,  y_position =   9 ,  xmin =   1 ,  xmax =   2 ,  tip_length =   c ( 0.02 ,  0.02 ),  vjust =   -  0.2 ) +  
    
      scale_fill_manual ( limits= Limits, 
                        values= palette_diet_2) +  
      scale_x_discrete ( "" , 
                       limits=  c ( "HS" ,  "HY" ), 
                       labels=  c ( "HS" ,  "HY" )) +  
      scale_y_continuous ( expression ( paste ( "EC density (per " ,mm ^  2 , ")" )), 
                         limits=  c ( 1.5 , 9.5 ), 
                         breaks=  seq ( 2 , 8 , by=  1 )) +  
          stat_summary ( fun =  mean,  geom =   "point" ,  size =   3 ,  shape =   18 ,  colour =   "black" ,  aes ( group =  Repeat))  +  
                         stat_summary ( fun =  mean,  geom =   "point" ,  size =   2 ,  shape =   18 ,  aes ( group =  Repeat,  colour =  Repeat))  +  
                         scale_color_manual ( values =  palette_mean)  +  
      theme ( 
        panel.grid.major.y =   element_line ( colour =   grey ( 0.45 ),  linetype =   "dashed" ,  size =   0.2 ), 
        panel.background =   element_blank (), 
        axis.title.x =   element_text ( size= Smallfont, colour=  "black" ), 
        axis.title.y =   element_text ( size= Smallfont, colour=  "black" ),  
        axis.line.x =   element_line ( colour=  "black" , size=  0.75 ), 
        axis.line.y =   element_line ( colour=  "black" , size=  0.75 ), 
        axis.ticks.x =   element_line ( size =   0.75 ), 
        axis.ticks.y =   element_line ( size =   0.75 ), 
        axis.text.x =   element_text ( size= Smallfont, colour=  "black" ), 
        axis.text.y =   element_text ( size= Smallfont, colour=  "black" ), 
        plot.margin =   unit (Margin,  "cm" ), 
        legend.direction =   "vertical" ,  
        legend.box =   "horizontal" , 
        legend.position =   "none" , 
        legend.key.height =   unit ( 0.4 ,  "cm" ), 
        legend.key.width=   unit ( 0.6 ,  "cm" ), 
        legend.title =   element_text ( face=  "italic" , size= Smallfont),  
        legend.key =   element_rect ( colour =   'white' ,  fill =   "white" ,  linetype=  'dashed' ), 
        legend.text =   element_text ( size= SuperSmallfont), 
        legend.background =   element_rect ( fill=  NA )) 
    
   Plot_Fig3S1A    
   
 
 
  3.2.2  Figure 3S1B 
 
 Scheme illustrating area measurements. Top view in this scheme is as in pictures shown in figure 3 D, E. 3D side view show side view with Mesh showing measured surface. 
 
   
 
 
  3.2.3  Figure 3S1C 
 
  Diet affects enterocyte size.  Quantification of EC height of MyoTS>GFP on HS or HY diet demonstrates an increase in cell height on HY diet. 
 
      tab_ECheight  =   
     d[[ "3 - S1C" ]] %>%  
     select (Diet, Repeat, GutNumber, Region, Height) %>%  
      mutate_if (is.character,as.factor) %>%   
      mutate_if (is.integer,as.factor) %>%   
     dplyr ::  rename ( EC_height= Height) %>%   
      drop_na () 
   tab_ECheight $ EC_height  <-   as.numeric (tab_ECheight $ EC_height) 
    
   Sample_size =  
     tab_ECheight %>%  
      group_by (Diet) %>%  
      summarise ( Sample_size=  n ()) 
    
    ###Stats  
    
   mod.gen  =   fitme ((EC_height)  ~   Diet  +  ( 1   |  Repeat),  data =  tab_ECheight) 
    shapiro.test ( residuals (mod.gen))     
  ## 
##  Shapiro-Wilk normality test
## 
## data:  residuals(mod.gen)
## W = 0.98665, p-value = 1.551e-06  
       bptest (EC_height  ~  Diet  +  ( 1   /  Repeat),  data =  tab_ECheight)     
  ## 
##  studentized Breusch-Pagan test
## 
## data:  EC_height ~ Diet + (1/Repeat)
## BP = 40.813, df = 1, p-value = 1.676e-10  
      mod.gen1  =   fitme (EC_height  ~   1   +  ( 1   |  Repeat),  data =  tab_ECheight)  
   test  =   anova (mod.gen, mod.gen1)  
   Chi2_LRT_growth  =   2  * (mod.gen $ APHLs[[ "p_v" ]] - mod.gen1 $ APHLs[[ "p_v" ]]) 
    
    #Now we make a tab with the results  
   tab_stat  =   data.frame ( Variable =   as.character ( paste ( "HS vs HY" )), 
                                   Rep =   nlevels (tab_ECheight $ Repeat), 
                                   chi2_LR =   round ( as.numeric (test $ basicLRT $ chi2_LR),  digits =   2 ), 
                                   intercept =   format (mod.gen $ fixef[ 1 ], digits=  3 ), 
                                   estimate =   format (mod.gen $ fixef[ 2 ], digits=  3 ), 
                                   df =   as.numeric (test $ basicLRT $ df), 
                                   Pvalue =   as.numeric ( format ( pchisq (Chi2_LRT_growth, df=  1 , lower.tail =  F), digits=  2 ))) 
   tab_stat $ sig  =   ifelse (tab_stat $ Pvalue  <   0.05   &  tab_stat $ Pvalue  >   0.01 ,  "*" , 
                 ifelse (tab_stat $ Pvalue  <   0.01   &  tab_stat $ Pvalue  >   0.001 ,  "**" , 
                  ifelse (tab_stat $ Pvalue  <   0.001 ,  "***" ,  "" ))) 
    
   tab_stat %>%  
      kable ( col.names =   c ( "Comparison" ,  "Replicates" ,  "Chi2" , "Intercept" , "Estimate" , "df"  , "p-value" , "Signif." ), row.names =   FALSE )  %>%     add_header_above ( c ( "EC height ~  Diet + (1 | Repeat)"   =   8 )) %>%  
      kable_styling ( bootstrap_options =   c ( "striped" ,  "hover" ,  "condensed" ),  full_width =  F)    
 
 
 
 
 
EC height ~ Diet + (1 | Repeat)
 
 
 
 
 
Comparison
 
 
Replicates
 
 
Chi2
 
 
Intercept
 
 
Estimate
 
 
df
 
 
p-value
 
 
Signif.
 
 
 
 
 
 
HS vs HY
 
 
2
 
 
494.51
 
 
7.09
 
 
5.03
 
 
1
 
 
0
 
 
***
 
 
 
 
      Limits =   c ( "HS" , "HY" ) 
    
   z =  max (tab_ECheight $ EC_height,  na.rm =   TRUE ) 
    
   Plot_Fig3S1C  =  
      ggplot (tab_ECheight,  aes ( x= Diet,  y= EC_height)) +   
      geom_violin ( aes ( fill =  Diet),  draw_quantiles =   c ( 0.25 ,  0.5 ,  0.75 ),  colour =   "black" ,  size =   0.2 , adjust =   0.8 )  +  
      geom_dotplot (  colour =   "black" ,  fill =   "white" ,  binaxis =   "y" ,  stackdir =   "center" ,  binwidth =  z /  150 )  +   
      geom_text ( data =  Sample_size,  mapping =   aes ( x =  Diet,  y =   -  1 ,  label =   paste ( "(" ,Sample_size, ")" , sep=  "" )), size=  3 ) +  
        geom_signif ( annotation =   formatC ( paste ( "p=" ,tab_stat $ Pvalue),  digits =   2 ),  textsize =   3 ,  y_position =   23 ,  xmin =   1 ,  xmax =   2 ,  tip_length =   c ( 0.02 ,  0.02 ),  vjust =   -  0.2 ) +  
    
      scale_fill_manual ( limits= Limits, 
                        values= palette_diet_2) +  
      scale_x_discrete ( "" , 
                       limits=  c ( "HS" ,  "HY" ), 
                       labels=  c ( "HS" ,  "HY" )) +  
      scale_y_continuous ( expression ( paste ( "EC height (" , mu,  "m)" )), 
                         limits=  c ( -  2 , 25 ), 
                         breaks=  seq ( 2 , 25 , by=  5 )) +  
      stat_summary ( fun =  mean,  geom =   "point" ,  size =   3 ,  shape =   18 ,  colour =   "black" ,  aes ( group =  Repeat))  +  
      stat_summary ( fun =  mean,  geom =   "point" ,  size =   2 ,  shape =   18 ,  aes ( group =  Repeat,  colour =  Repeat))  +  
      scale_color_manual ( values =  palette_mean)  +  
      theme ( 
        panel.grid.major.y =   element_line ( colour =   grey ( 0.45 ),  linetype =   "dashed" ,  size =   0.2 ), 
        panel.background =   element_blank (), 
        axis.title.x =   element_text ( size= Smallfont, colour=  "black" ), 
        axis.title.y =   element_text ( size= Smallfont, colour=  "black" ),  
        axis.line.x =   element_line ( colour=  "black" , size=  0.75 ), 
        axis.line.y =   element_line ( colour=  "black" , size=  0.75 ), 
        axis.ticks.x =   element_line ( size =   0.75 ), 
        axis.ticks.y =   element_line ( size =   0.75 ), 
        axis.text.x =   element_text ( size= Smallfont, colour=  "black" ), 
        axis.text.y =   element_text ( size= Smallfont, colour=  "black" ), 
        plot.margin =   unit (Margin,  "cm" ), 
        legend.direction =   "vertical" ,  
        legend.box =   "horizontal" , 
        legend.position =   "none" , 
        legend.key.height =   unit ( 0.4 ,  "cm" ), 
        legend.key.width=   unit ( 0.6 ,  "cm" ), 
        legend.title =   element_text ( face=  "italic" , size= Smallfont),  
        legend.key =   element_rect ( colour =   'white' ,  fill =   "white" ,  linetype=  'dashed' ), 
        legend.text =   element_text ( size= SuperSmallfont), 
        legend.background =   element_rect ( fill=  NA )) 
    
   Plot_Fig3S1C    
   
 
 
  3.2.4  Figure 3S1D 
 
 Representative density plot from FACS for HS diet  Complete annotation found in manuscript’s figures  
 
   
 
 
  3.2.5  Figure 3S1E 
 
 Representative density plot from FACS for HY diet  Complete annotation found in manuscript’s figures  
 
   
 
 
  3.2.6  Figure 3S1F 
 
 Representative frequency plot from FACS for HS diet.  Complete annotation found in manuscript’s figures  
 
   
 
 
  3.2.7  Figure 3S1G 
 
 Representative frequency plot from FACS for HY diet Complete annotation found in manuscript’s figures  
 
   
 
 
  3.2.8  Figure 3S1H 
 
  Ploidy of midguts on either HS or HY diets is largely unchanged.  Stacked bar plot from 7 repeats, each of 25 midguts 
 
      tab_ploidy_rev  =   
     d[[ "3 - S1H" ]] %>%  
      mutate_if (is.character,as.factor) %>%  
      mutate_if (is.integer,as.factor) %>%  
      group_by (Diet, Ploidy) %>%  
      summarise ( mean_Percentage=  mean (Percentage, na.rm= T), 
                sd_Percentage=  sd (Percentage)) %>%  
      mutate ( group=  paste (Diet, Ploidy, sep=  "_" )) %>%  
      mutate ( Ploidy=  fct_relevel (Ploidy, "2" , "4" ,  "8" ,  "16" ,  "32" ,  "64" ,  "64+" )) %>%  
      as.data.frame () 
    
    
   Sample_size =  
       tab_ploidy_rev %>%  
      mutate_if (is.character,as.factor) %>%  
      mutate_if (is.integer,as.factor) %>%  
      group_by (Diet) %>%  
      summarise ( Sample_size=  n ()) 
    
    
    
    # Creation of dataset with right position for error bar  
    ##HS  
   tmp  =   subset (tab_ploidy_rev , Diet %in%  c ( "HS" )) 
    
    
   tmp1  <-   subset (tmp,  select =   -  c (group , sd_Percentage, Diet)) 
    
    
   tmpw1  <-  spread (tmp1, Ploidy, mean_Percentage) 
    
    
    
   tmpw1 $  `  2.p  `   =  tmpw1 $  `  2  `  
   tmpw1 $  `  4.p  `   =  tmpw1 $  `  2  `   +  tmpw1 $  `  4  `  
   tmpw1 $  `  8.p  `   =  tmpw1 $  `  4.p  `   +  tmpw1 $  `  8  `  
   tmpw1 $  `  16.p  `   =  tmpw1 $  `  8.p  `   +  tmpw1 $  `  16  `  
   tmpw1 $  `  32.p  `   =  tmpw1 $  `  16.p  `   +  tmpw1 $  `  32  `  
   tmpw1 $  `  64.p  `   =  tmpw1 $  `  32.p  `   +  tmpw1 $  `  64  `  
   tmpw1 $  `  64+.p  `   =  tmpw1 $  `  64.p  `   +  tmpw1 $  `  64+  `  
    
    
   tmp2  <-   subset (tmp,  select =   -  c (group , mean_Percentage, Diet)) 
   tmpw2  <-  spread (tmp2, Ploidy, sd_Percentage) 
    
   tmpw1 $  `  2.se  `   =  tmpw2 $  `  2  `  
   tmpw1 $  `  4.se  `   =  tmpw2 $  `  4  `  
   tmpw1 $  `  8.se  `   =  tmpw2 $  `  8  `  
   tmpw1 $  `  16.se  `   =  tmpw2 $  `  16  `  
   tmpw1 $  `  32.se  `   =  tmpw2 $  `  32  `  
   tmpw1 $  `  64.se  `   =  tmpw2 $  `  64  `  
   tmpw1 $  `  64+.se  `   =  tmpw2 $  `  64+  `  
    
    
   tmpw1 $  `  2.se+  `   =  tmpw1 $  `  2  `   +  tmpw1 $  `  2.se  `  
   tmpw1 $  `  4.se+  `   =  tmpw1 $  `  4.p  `   +  tmpw1 $  `  4.se  `  
   tmpw1 $  `  8.se+  `   =  tmpw1 $  `  8.p  `   +  tmpw1 $  `  8.se  `  
   tmpw1 $  `  16.se+  `   =  tmpw1 $  `  16.p  `   +  tmpw1 $  `  16.se  `  
   tmpw1 $  `  32.se+  `   =  tmpw1 $  `  32.p  `   +  tmpw1 $  `  32.se  `  
   tmpw1 $  `  64.se+  `   =  tmpw1 $  `  64.p  `   +  tmpw1 $  `  64.se  `  
   tmpw1 $  `  64+.se+  `   =  tmpw1 $  `  64+.p  `   +  tmpw1 $  `  64+.se  `  
    
    
   tmpw1 $  `  2.se-  `   =  tmpw1 $  `  2  `   -  tmpw1 $  `  2.se  `  
   tmpw1 $  `  4.se-  `   =  tmpw1 $  `  4.p  `   -  tmpw1 $  `  4.se  `  
   tmpw1 $  `  8.se-  `   =  tmpw1 $  `  8.p  `   -  tmpw1 $  `  8.se  `  
   tmpw1 $  `  16.se-  `   =  tmpw1 $  `  16.p  `   -  tmpw1 $  `  16.se  `  
   tmpw1 $  `  32.se-  `   =  tmpw1 $  `  32.p  `   -  tmpw1 $  `  32.se  `  
   tmpw1 $  `  64.se-  `   =  tmpw1 $  `  64.p  `   -  tmpw1 $  `  64.se  `  
   tmpw1 $  `  64+.se-  `   =  tmpw1 $  `  64+.p  `   -  tmpw1 $  `  64+.se  `  
    
    
   tmpl  <-   reshape ( data= tmpw1,  
        varying =   list ( Ploidy =   c ( 1  :  7 ),  Position =   c ( 8  :  14 ),  se =   c ( 15  :  21 ),  seplus =   c ( 22  :  28 ),  seminus =   c ( 29  :  35 )), 
        direction =   'long' , 
        v.names =   c ( "Percentage" ,  "Position" ,  "se" ,  "seplus" ,  "seminus" ), 
        sep =   "." ) 
    
   tmpl $ Ploidy  =   c ( "2" , "4" ,  "8" ,  "16" ,  "32" ,  "64" ,  "64+" ) 
    
   tmpl $ Diet  =   "HS"  
    
    
   tmpl  <-   subset (tmpl,  select =   -  c (time , id)) 
    
   tmpl_HS  <-  tmpl 
    
    ##HY  
   tmp  =   subset (tab_ploidy_rev , Diet %in%  c ( "HY" )) 
    
    
   tmp1  <-   subset (tmp,  select =   -  c (group , sd_Percentage, Diet)) 
    
    
   tmpw1  <-  spread (tmp1, Ploidy, mean_Percentage) 
    
    
    
    
    
   tmpw1 $  `  2.p  `   =  tmpw1 $  `  2  `  
   tmpw1 $  `  4.p  `   =  tmpw1 $  `  2  `   +  tmpw1 $  `  4  `  
   tmpw1 $  `  8.p  `   =  tmpw1 $  `  4.p  `   +  tmpw1 $  `  8  `  
   tmpw1 $  `  16.p  `   =  tmpw1 $  `  8.p  `   +  tmpw1 $  `  16  `  
   tmpw1 $  `  32.p  `   =  tmpw1 $  `  16.p  `   +  tmpw1 $  `  32  `  
   tmpw1 $  `  64.p  `   =  tmpw1 $  `  32.p  `   +  tmpw1 $  `  64  `  
   tmpw1 $  `  64+.p  `   =  tmpw1 $  `  64.p  `   +  tmpw1 $  `  64+  `  
    
    
   tmp2  <-   subset (tmp,  select =   -  c (group , mean_Percentage, Diet)) 
   tmpw2  <-  spread (tmp2, Ploidy, sd_Percentage) 
    
   tmpw1 $  `  2.se  `   =  tmpw2 $  `  2  `  
   tmpw1 $  `  4.se  `   =  tmpw2 $  `  4  `  
   tmpw1 $  `  8.se  `   =  tmpw2 $  `  8  `  
   tmpw1 $  `  16.se  `   =  tmpw2 $  `  16  `  
   tmpw1 $  `  32.se  `   =  tmpw2 $  `  32  `  
   tmpw1 $  `  64.se  `   =  tmpw2 $  `  64  `  
   tmpw1 $  `  64+.se  `   =  tmpw2 $  `  64+  `  
    
    
   tmpw1 $  `  2.se+  `   =  tmpw1 $  `  2  `   +  tmpw1 $  `  2.se  `  
   tmpw1 $  `  4.se+  `   =  tmpw1 $  `  4.p  `   +  tmpw1 $  `  4.se  `  
   tmpw1 $  `  8.se+  `   =  tmpw1 $  `  8.p  `   +  tmpw1 $  `  8.se  `  
   tmpw1 $  `  16.se+  `   =  tmpw1 $  `  16.p  `   +  tmpw1 $  `  16.se  `  
   tmpw1 $  `  32.se+  `   =  tmpw1 $  `  32.p  `   +  tmpw1 $  `  32.se  `  
   tmpw1 $  `  64.se+  `   =  tmpw1 $  `  64.p  `   +  tmpw1 $  `  64.se  `  
   tmpw1 $  `  64+.se+  `   =  tmpw1 $  `  64+.p  `   +  tmpw1 $  `  64+.se  `  
    
    
   tmpw1 $  `  2.se-  `   =  tmpw1 $  `  2  `   -  tmpw1 $  `  2.se  `  
   tmpw1 $  `  4.se-  `   =  tmpw1 $  `  4.p  `   -  tmpw1 $  `  4.se  `  
   tmpw1 $  `  8.se-  `   =  tmpw1 $  `  8.p  `   -  tmpw1 $  `  8.se  `  
   tmpw1 $  `  16.se-  `   =  tmpw1 $  `  16.p  `   -  tmpw1 $  `  16.se  `  
   tmpw1 $  `  32.se-  `   =  tmpw1 $  `  32.p  `   -  tmpw1 $  `  32.se  `  
   tmpw1 $  `  64.se-  `   =  tmpw1 $  `  64.p  `   -  tmpw1 $  `  64.se  `  
   tmpw1 $  `  64+.se-  `   =  tmpw1 $  `  64+.p  `   -  tmpw1 $  `  64+.se  `  
    
    
   tmpl  <-   reshape ( data= tmpw1,  
        varying =   list ( Ploidy =   c ( 1  :  7 ),  Position =   c ( 8  :  14 ),  se =   c ( 15  :  21 ),  seplus =   c ( 22  :  28 ),  seminus =   c ( 29  :  35 )), 
        direction =   'long' , 
        v.names =   c ( "Percentage" ,  "Position" ,  "se" ,  "seplus" ,  "seminus" ), 
        sep =   "." ) 
    
   tmpl $ Ploidy  =   c ( "2" , "4" ,  "8" ,  "16" ,  "32" ,  "64" ,  "64+" ) 
    
   tmpl $ Diet  =   "HY"  
    
    
   tmpl  <-   subset (tmpl,  select =   -  c (time , id)) 
    
   tmpl_HY  <-  tmpl 
    
    #Bind, rename and reorcer  
   tab_ploidy  <-   rbind (tmpl_HS, tmpl_HY) 
   tab_ploidy $ Ploidy  <-   as.factor (tab_ploidy $ Ploidy) 
    
   tab_ploidy $ Ploidy  <-  factor (tab_ploidy $ Ploidy,  levels =  c ( "64+" , "64" ,  "32" ,  "16" ,  "8" ,  "4" ,  "2" )) 
    
    #Plot  
    
                                      
   Plot_Fig3S1H  =  
      ggplot (tab_ploidy,  aes ( x= Diet,  y= Percentage)) +   
      geom_bar ( stat=  "identity" , aes ( fill= Ploidy), color=  "black" , width= . 90 ) +  
      geom_errorbar ( aes ( ymin=  seminus,  ymax=  seplus), width=  0.25 ,  color =   "black" ) +  
      geom_text ( data =  Sample_size,  mapping =   aes ( x =  Diet,  y =   -  5 ,  label =   paste ( "(" ,Sample_size, ")" , sep=  "" )), size=  3 ) +  
      scale_fill_manual ( name =   "Ploidy" ,  
                        values=  c ( "#0052A2" , "#1A63AB" ,  "#3375B5" ,  "#6697C7" ,  "#99BADA" ,  "#CCDCEC" ,  "#E6EEF6" ), 
                        labels =   c ( "64+n" ,  "64n" ,  "32n" ,  "16n" ,  "8n" ,  "4n" ,  "2n" )) +  
      scale_y_continuous ( "Percentage of ploidy (mean \u00B1sd)" , 
                         limits=  c ( -  5 , 90 ), 
                         breaks=  seq ( 0 , 80 , by=  20 )) +  
      theme ( 
        panel.grid.major.y =   element_line ( colour =   grey ( 0.45 ),  linetype =   "dashed" ,  size =   0.2 ), 
        panel.background =   element_blank (), 
        axis.title.x =   element_blank (), 
        axis.title.y =   element_text ( size= Smallfont, colour=  "black" ),  
        axis.line.x =   element_line ( colour=  "black" , size=  0.75 ), 
        axis.line.y =   element_line ( colour=  "black" , size=  0.75 ), 
        axis.ticks.x =   element_line ( size =   0.75 ), 
        axis.ticks.y =   element_line ( size =   0.75 ), 
        axis.text.x =   element_text ( size= Smallfont, colour=  "black" ), 
        axis.text.y =   element_text ( size= Smallfont, colour=  "black" ), 
        plot.margin =   unit (Margin,  "cm" ), 
        legend.direction =   "horizontal" ,  
        legend.box =   "horizontal" , 
        legend.position =   "bottom" , 
        #legend.key.height = unit(0.6, "cm"),  
        #legend.key.width= unit(0.4, "cm"),  
        legend.title =   element_blank (),  
        legend.key =   element_rect ( colour =   'white' ,  fill =   "white" ,  linetype=  'dashed' ), 
        legend.text =   element_text ( size= Smallfont), 
        legend.background =   element_rect ( fill=  NA ), 
        strip.text =   element_blank ()) +  
        guides ( fill=  guide_legend ( nrow=  4 , byrow=  TRUE )) 
        
        #strip.background = element_rect(fill=NA, colour="black"),  
        #strip.placement="outside")  
    
   Plot_Fig3S1H    
   
 ##Export Figure 3S1 
 
 
 
 
  4  Figure 4. Shifts in diet composition lead to plastic midgut resizing and changes in absolute and relative cell loss and gain 
 
  4.1  Figure 4 - main 
 
  4.1.1  Figure 4A 
 
 Midguts can respond plastically to changes in isocaloric diets. Midgut length increases from eclosion on HY for 7 days, then decreases when switched to HS for additional 7 days but can re-increase size upon a further 7 days HY feeding. Letters above violin plots represent grouping by statistical differences (Post hoc Tukey on GLMM). 
 
      Length_plasticity_time  =   
     d[[ "4A" ]] %>%  
      mutate_at ( vars ( starts_with ( "Total" )), ~ . /  1000 ) %>%  
      mutate_if (is.character,as.factor) %>%  
      mutate_if (is.integer,as.factor) %>%  
     dplyr ::  rename ( Total_Length_mm= Total.L, 
             Day_of_treatment= Day) 
    
   Sample_size =  
     Length_plasticity_time %>%  
      group_by (Diet) %>%  
      summarise ( Sample_size=  n ()) 
    
    ###Stats  
    
   mod.gen  =   fitme ( log (Total_Length_mm)  ~   Diet  +  ( 1   |  Repeat),  data =  Length_plasticity_time) 
    shapiro.test ( residuals (mod.gen))     
  ## 
##  Shapiro-Wilk normality test
## 
## data:  residuals(mod.gen)
## W = 0.99167, p-value = 0.7868  
       bptest ( log (Total_Length_mm)  ~  Diet  +  ( 1   /  Repeat),  data =  Length_plasticity_time)     
  ## 
##  studentized Breusch-Pagan test
## 
## data:  log(Total_Length_mm) ~ Diet + (1/Repeat)
## BP = 12.435, df = 3, p-value = 0.006032  
      mod.gen1  =   fitme ( log (Total_Length_mm)  ~   1   +  ( 1   |  Repeat),  data =  Length_plasticity_time)  
   test  =   anova (mod.gen, mod.gen1)  
   Chi2_LRT_growth  =   2  * (mod.gen $ APHLs[[ "p_v" ]] - mod.gen1 $ APHLs[[ "p_v" ]]) 
    
   tab_stat  =   data.frame ( Variable =   as.character ( paste ( "Anova diets" )), 
                                   Rep =   nlevels (Length_plasticity_time $ Repeat), 
                                   chi2_LR =   round ( as.numeric (test $ basicLRT $ chi2_LR),  digits =   2 ), 
                                   intercept =   format (mod.gen $ fixef[ 1 ], digits=  3 ), 
                                   estimate =   format (mod.gen $ fixef[ 2 ], digits=  3 ), 
                                   df =   as.numeric (test $ basicLRT $ df), 
                                   Pvalue =   as.numeric ( format ( pchisq (Chi2_LRT_growth, df=  1 , lower.tail =  F), digits=  2 ))) 
   tab_stat $ sig  =   ifelse (tab_stat $ Pvalue  <   0.05   &  tab_stat $ Pvalue  >   0.01 ,  "*" , 
                 ifelse (tab_stat $ Pvalue  <   0.01   &  tab_stat $ Pvalue  >   0.001 ,  "**" , 
                  ifelse (tab_stat $ Pvalue  <   0.001 ,  "***" ,  "" ))) 
    
   tab_stat %>%  
      kable ( col.names =   c ( "Comparison" ,  "Replicates" ,  "Chi2" , "Intercept" , "Estimate" , "df"  , "p-value" , "Signif." ), row.names =   FALSE )  %>%     add_header_above ( c ( "log(Total_Length_mm) ~  Diet + (1 | Repeat)"   =   8 )) %>%  
      kable_styling ( bootstrap_options =   c ( "striped" ,  "hover" ,  "condensed" ),  full_width =  F)    
 
 
 
 
 
log(Total_Length_mm) ~ Diet + (1 | Repeat)
 
 
 
 
 
Comparison
 
 
Replicates
 
 
Chi2
 
 
Intercept
 
 
Estimate
 
 
df
 
 
p-value
 
 
Signif.
 
 
 
 
 
 
Anova diets
 
 
3
 
 
106.66
 
 
1.22
 
 
0.553
 
 
3
 
 
0
 
 
***
 
 
 
 
      mod.gen  =   lmer ( log (Total_Length_mm)  ~   Diet  +  ( 1   |  Repeat),  data =  Length_plasticity_time) 
   multcomp  =   glht (mod.gen,  linfct=  mcp ( Diet=  "Tukey" )) 
    
   tmp  =   cld (multcomp) 
    
   letter_position  =   aggregate ( data= Length_plasticity_time,Total_Length_mm  ~   Diet, max) 
    
   tab_letter  =    as.data.frame (tmp $ mcletters $ Letters) 
   tab_letter $ Diet =  rownames (tab_letter) 
    colnames (tab_letter)[ 1 ]  =   "Letter"  
   tab_letter  =   left_join (tab_letter,letter_position) 
    
    ### Plot  
   Limits  =   c ( "Eclosion" , "HY" , "HYtoHS" ,  "HYtoHStoHY" ) 
   z  =   max (Length_plasticity_time $ Total_Length_mm,  na.rm =   TRUE ) 
    
   Plot_Fig4A =  
      ggplot (Length_plasticity_time,  aes ( x =  Diet,  y =  Total_Length_mm)) +   
      geom_violin ( aes ( fill =  Diet),  draw_quantiles =   c ( 0.25 ,  0.5 ,  0.75 ),  colour =   "black" ,  size =   0.2 , adjust =   0.8 )  +  
      geom_dotplot (  colour =   "black" ,  fill =   "white" ,  binaxis =   "y" ,  stackdir =   "center" ,  binwidth =  z /  60 )  +   
      geom_text ( data =  Sample_size,  mapping =   aes ( x =  Diet,  y =   1.5 ,  label =   paste ( "(" ,Sample_size, ")" , sep=  "" )), size=  3 ) +  
    geom_text ( data =  tab_letter,  mapping =   aes ( x =  Diet,  y =  Total_Length_mm +0.4 ,  label =  Letter), size=  3 ) +  
      geom_text ( data =  tab_stat,  mapping =   aes ( x =   1.2 ,  y =   7.5 ,  label =   paste ( "p=" , format (Pvalue, digits=  2 ))), size=  3 ) +  
      scale_fill_manual ( limits= Limits, 
                        values= cbbPalette_4) +  
      scale_x_discrete ( "" , 
                       limits= Limits, 
                       labels=  c ( "Eclosion" , "HY" , "HY to HS" ,  "HY to HS to HY" )) +  
      scale_y_continuous ( "Midgut length (mm)" , 
                         limits=  c ( 1.3 , 8.2 ), 
                         breaks=  seq ( 2 , 8 , by=  1 ), 
                         minor_breaks =   seq ( 3 ,  7 , by=   1 )) +  
      stat_summary ( fun =  mean,  geom =   "point" ,  size =   3 ,  shape =   18 ,  colour =   "black" ,  aes ( group =  Repeat))  +  
                         stat_summary ( fun =  mean,  geom =   "point" ,  size =   2 ,  shape =   18 ,  aes ( group =  Repeat,  colour =  Repeat))  +  
                         scale_color_manual ( values =  palette_mean)  +  
      theme ( panel.grid.major.y =   element_line ( colour =   grey ( 0.45 ),  linetype =   "dashed" ,  size =   0.2 ), 
            panel.background =   element_blank (), 
            axis.title.x =   element_text ( size= Smallfont, colour=  "black" ), 
            axis.title.y =   element_text ( size= Smallfont, colour=  "black" ),  
            axis.line.x =   element_line ( colour=  "black" , size=  0.75 ), 
            axis.line.y =   element_line ( colour=  "black" , size=  0.75 ), 
            axis.ticks.x =   element_line ( size =   0.75 ), 
            axis.ticks.y =   element_line ( size =   0.75 ), 
            axis.text.x =   element_text ( size= Smallfont, colour=  "black" , angle=  30 , hjust=  1 ), 
            axis.text.y =   element_text ( size= Smallfont, colour=  "black" ), 
            plot.margin =   unit (Margin,  "cm" ), 
            legend.direction =   "vertical" ,  
            legend.box =   "horizontal" , 
            legend.position =   "none" , 
            legend.key.height =   unit ( 0.4 ,  "cm" ), 
            legend.key.width=   unit ( 0.6 ,  "cm" ), 
            legend.title =   element_text ( face=  "italic" , size= Smallfont),  
            legend.key =   element_rect ( colour =   'white' ,  fill =   "white" ,  linetype=  'dashed' ), 
            legend.text =   element_text ( size= SuperSmallfont), 
            legend.background =   element_rect ( fill=  NA ), 
            strip.text.x =   element_text ( size = Smallfont,  colour =   "black" , face=  "italic" ), 
            strip.text.y =   element_text ( size = Smallfont,  colour =   "black" , face=  "italic" ), 
            strip.background =   element_rect ( fill=  NA ,  colour=  "black" ), 
            strip.placement=  "outside" ) 
    
   Plot_Fig4A    
   
 
 
  4.1.2  Figure 4B 
 
 Mitotically active cells visualized by phospho-Histone H3 (pH3) immunostaining are more numerous on HY diet than on HS diet. pH3+ cells gradually increase over time on HY, but not HS diet. Letters above violin plots represent grouping by statistical differences (Post hoc Tukey on GLMM). 
 
      Length_Diet_time  =   
     d[[ "4B, 4S1C" ]] %>%  
      mutate_at ( vars ( ends_with ( ".L" )), ~ . /  1000 ) %>%  
      mutate_at ( vars ( !  starts_with ( "Total" )),as.factor) %>%  
      mutate ( group=  paste (Diet, Day, sep=  "_" )) %>%  
    dplyr ::  rename ( Total_Length_mm= Total.L, 
             PH3_positive_cell= Total.PH3, 
             Day_of_treatment= Day) 
    
   Sample_size =  
     Length_Diet_time %>%  
      group_by (Diet,Day_of_treatment) %>%  
      summarise ( Sample_size=  n ()) 
    
    ###Stats  
    
   mod.gen  =   fitme (PH3_positive_cell  ~   group  +  ( 1   |  Repeat), data =   subset (Length_Diet_time, !  is.na (PH3_positive_cell))) 
    shapiro.test ( residuals (mod.gen))     
  ## 
##  Shapiro-Wilk normality test
## 
## data:  residuals(mod.gen)
## W = 0.97954, p-value = 0.01115  
       bptest (PH3_positive_cell  ~   group  +  ( 1   /  Repeat), data =   subset (Length_Diet_time, !  is.na (PH3_positive_cell)))    
  ## 
##  studentized Breusch-Pagan test
## 
## data:  PH3_positive_cell ~ group + (1/Repeat)
## BP = 41.055, df = 7, p-value = 7.901e-07  
      mod.gen1  =   fitme (PH3_positive_cell  ~    1   +  ( 1   |  Repeat), data =   subset (Length_Diet_time, !  is.na (PH3_positive_cell))) 
   test  =   anova (mod.gen, mod.gen1)  
   Chi2_LRT_growth  =   2  * (mod.gen $ APHLs[[ "p_v" ]] - mod.gen1 $ APHLs[[ "p_v" ]]) 
    
   tab_stat  =   data.frame ( Variable =   as.character ( paste ( "Any difference" )), 
                                   Rep =   nlevels (Length_Diet_time $ Repeat), 
                                   chi2_LR =   round ( as.numeric (test $ basicLRT $ chi2_LR),  digits =   2 ), 
                                   intercept =   format (mod.gen $ fixef[ 1 ], digits=  3 ), 
                                   estimate =   format (mod.gen $ fixef[ 2 ], digits=  3 ), 
                                   df =   as.numeric (test $ basicLRT $ df), 
                                   Pvalue =   as.numeric ( format ( pchisq (Chi2_LRT_growth, df=  1 , lower.tail =  F), digits=  2 ))) 
   tab_stat $ sig  =   ifelse (tab_stat $ Pvalue  <   0.05   &  tab_stat $ Pvalue  >   0.01 ,  "*" , 
                 ifelse (tab_stat $ Pvalue  <   0.01   &  tab_stat $ Pvalue  >   0.001 ,  "**" , 
                  ifelse (tab_stat $ Pvalue  <   0.001 ,  "***" ,  "" ))) 
    
   tab_stat %>%  
      kable ( col.names =   c ( "Comparison" ,  "Replicates" ,  "Chi2" , "Intercept" , "Estimate" , "df"  , "p-value" , "Signif." ), row.names =   FALSE )  %>%     add_header_above ( c ( "PH3_positive_cell ~  group + (1 | Repeat)"   =   8 )) %>%  
      kable_styling ( bootstrap_options =   c ( "striped" ,  "hover" ,  "condensed" ),  full_width =  F)    
 
 
 
 
 
PH3_positive_cell ~ group + (1 | Repeat)
 
 
 
 
 
Comparison
 
 
Replicates
 
 
Chi2
 
 
Intercept
 
 
Estimate
 
 
df
 
 
p-value
 
 
Signif.
 
 
 
 
 
 
Any difference
 
 
3
 
 
248.1
 
 
11.4
 
 
3.98
 
 
7
 
 
0
 
 
***
 
 
 
 
      tab_stat $ Diet =  "HS"  
    
   mod.gen  =   lmer (PH3_positive_cell  ~   group  +  ( 1   |  Repeat), data =  subset (Length_Diet_time, !  is.na (PH3_positive_cell))) 
   multcomp  =   glht (mod.gen,  linfct=  mcp ( group=  "Tukey" )) 
    
   tmp  =   cld (multcomp) 
    
   letter_position  =   aggregate ( data=  subset (Length_Diet_time, !  is.na (PH3_positive_cell)),PH3_positive_cell  ~   group, max) 
    
   tab_letter  =    as.data.frame (tmp $ mcletters $ Letters) 
   tab_letter $ group =  rownames (tab_letter) 
    colnames (tab_letter)[ 1 ]  =   "Letter"  
   tab_letter  =   left_join (tab_letter,letter_position) 
   tab_letter  =   separate (tab_letter,group,  c ( "Diet" ,  "Day_of_treatment" ),  sep =   "_" ,  remove=  FALSE ) 
    
    ### Plot  
   Limits  =   c ( "7" , "14" , "21" ,  "28" ) 
   z  =   max (Length_Diet_time $ PH3_positive_cell,  na.rm =   TRUE ) 
    
   Plot_Fig4B =  
      ggplot (Length_Diet_time,  aes ( x =  Day_of_treatment,  y =  PH3_positive_cell)) +   
      geom_violin ( aes ( fill =  Diet),  draw_quantiles =   c ( 0.25 ,  0.5 ,  0.75 ),  colour =   "black" ,  size =   0.2 , adjust =   0.8 )  +  
      geom_dotplot (  colour =   "black" ,  fill =   "white" ,  binaxis =   "y" ,  stackdir =   "center" ,  binwidth =  z /  40 )  +   
      facet_grid (.  ~  Diet) +  
      geom_text ( data =  Sample_size,  mapping =   aes ( x =  Day_of_treatment,  y =   -  8 ,  label =   paste ( "(" ,Sample_size, ")" , sep=  "" )), size=  3 ) +  
      geom_text ( data =  tab_letter,  mapping =   aes ( x =  Day_of_treatment,  y =  PH3_positive_cell +  10 ,  label =  Letter), size=  3 ) +  
      geom_text ( data =  tab_stat,  mapping =   aes ( x =   2 ,  y =   130 ,  label =   paste ( "p=" , format (Pvalue, digits=  2 ))), size=  3 ) +  
       scale_fill_manual ( limits=  c ( "HS" , "HY" ), 
                         values= palette_diet_2) +  
      scale_x_discrete ( "" , 
                       limits= Limits, 
                       labels=  c ( "7 days" , "14 days" ,  "21 days" ,  "28 days" )) +  
      scale_y_continuous ( expression ( paste ( "pH3"   ^   "+" ,  " cells" )), 
                         limits=  c ( -  10 , 140 ), 
                         breaks=  seq ( 0 , 140 , by=  20 )) +  
         stat_summary ( fun =  mean,  geom =   "point" ,  size =   3 ,  shape =   18 ,  colour =   "black" ,  aes ( group =  Repeat))  +  
                         stat_summary ( fun =  mean,  geom =   "point" ,  size =   2 ,  shape =   18 ,  aes ( group =  Repeat,  colour =  Repeat))  +  
                         scale_color_manual ( values =  palette_mean)  +  
      theme ( panel.grid.major.y =   element_line ( colour =   grey ( 0.45 ),  linetype =   "dashed" ,  size =   0.2 ), 
            panel.background =   element_blank (), 
            axis.title.x =   element_text ( size= Smallfont, colour=  "black" ), 
            axis.title.y =   element_text ( size= Smallfont, colour=  "black" ),  
            axis.line.x =   element_line ( colour=  "black" , size=  0.75 ), 
            axis.line.y =   element_line ( colour=  "black" , size=  0.75 ), 
            axis.ticks.x =   element_line ( size =   0.75 ), 
            axis.ticks.y =   element_line ( size =   0.75 ), 
            axis.text.x =   element_text ( size= Smallfont, colour=  "black" , angle=  30 , hjust=  1 ), 
            axis.text.y =   element_text ( size= Smallfont, colour=  "black" ), 
            plot.margin =   unit (Margin,  "cm" ), 
            legend.direction =   "vertical" ,  
            legend.box =   "horizontal" , 
            legend.position =   "none" , 
            legend.key.height =   unit ( 0.4 ,  "cm" ), 
            legend.key.width=   unit ( 0.6 ,  "cm" ), 
            legend.title =   element_text ( face=  "italic" , size= Smallfont),  
            legend.key =   element_rect ( colour =   'white' ,  fill =   "white" ,  linetype=  'dashed' ), 
            legend.text =   element_text ( size= SuperSmallfont), 
            legend.background =   element_rect ( fill=  NA ), 
            strip.text.x =   element_text ( size =  Smallfont,  colour =   "black" ,  margin =   margin ( t =   2 ,  r =   0 ,  b =   2 ,  l =   0 )), 
            strip.text.y =   element_text ( size =  Smallfont,  colour =   "black" ,  margin =   margin ( t =   2 ,  r =   0 ,  b =   2 ,  l =   0 )), 
            strip.background =   element_rect ( fill=  NA ,  colour=  "black" ), 
            strip.placement=  "outside" ) 
    
   Plot_Fig4B    
   
 
 
  4.1.3  Figure 4C 
 
 Shifting between diets impacts pH3+ cell number in growth (HS to HY) experiments. Statistical comparisons are vs pre-shift measurement. 
 
      Length_Growth_PH3  =   
     d[[ "4C, 4S1D" ]] %>%  
      mutate_at ( vars ( ends_with ( ".L" )), ~ . /  1000 ) %>%  
      mutate_at ( vars ( !  starts_with ( "Total" )),as.factor) %>%  
     dplyr ::  rename ( Total_Length_mm= Total.L, 
             PH3_positive_cell= Total.PH3, 
             Day_of_treatment= Day) %>%  
      as.data.frame () %>%  
      mutate ( Dday=  fct_relevel (Dday, "Shift Day 7" , "Shift Day 14" , "Shift Day 21" )) 
    
   Sample_size =  
     Length_Growth_PH3 %>%  
      group_by (Diet,Dday) %>%  
      summarise ( Sample_size=  n ()) 
    
    ###Stats  
    ###Day 7  
   mod.gen  =   fitme ( log (PH3_positive_cell)  ~   Diet  +  ( 1   |  Repeat), data =   subset (Length_Growth_PH3,Dday ==  "Shift Day 7" )) 
    shapiro.test ( residuals (mod.gen))     
  ## 
##  Shapiro-Wilk normality test
## 
## data:  residuals(mod.gen)
## W = 0.97011, p-value = 0.2797  
       bptest ( log (PH3_positive_cell)  ~   Diet  +  ( 1   /  Repeat), data =   subset (Length_Growth_PH3,Dday ==  "Shift Day 7" ))    
  ## 
##  studentized Breusch-Pagan test
## 
## data:  log(PH3_positive_cell) ~ Diet + (1/Repeat)
## BP = 1.8138, df = 1, p-value = 0.1781  
      mod.gen1  =   fitme ( log (PH3_positive_cell)  ~    1   +  ( 1   |  Repeat), data =   subset (Length_Growth_PH3,Dday ==  "Shift Day 7" )) 
   test  =   anova (mod.gen, mod.gen1) 
   Chi2_LRT_growth  =   2  * (mod.gen $ APHLs[[ "p_v" ]] - mod.gen1 $ APHLs[[ "p_v" ]]) 
    
   tab_stat  =   data.frame ( Comparison =   as.character ( paste ( "HS Day 7 vs HS to HY Day 14" )), 
                          Variable =   as.character ( paste ( "Shift Day 7" )), 
                                   Rep =   nlevels (Length_Growth_PH3 $ Repeat), 
                                   chi2_LR =   round ( as.numeric (test $ basicLRT $ chi2_LR),  digits =   2 ), 
                                   intercept =   format (mod.gen $ fixef[ 1 ], digits=  3 ), 
                                   estimate =   format (mod.gen $ fixef[ 2 ], digits=  3 ), 
                                   df =   as.numeric (test $ basicLRT $ df), 
                                   Pvalue =   as.numeric ( format ( pchisq (Chi2_LRT_growth, df=  1 , lower.tail =  F), digits=  2 ))) 
   tab_stat_7 = tab_stat 
    
    #Day 14  
   mod.gen  =   fitme ( log (PH3_positive_cell)  ~   Diet  +  ( 1   |  Repeat), data =   subset (Length_Growth_PH3,Dday ==  "Shift Day 14" )) 
    shapiro.test ( residuals (mod.gen))     
  ## 
##  Shapiro-Wilk normality test
## 
## data:  residuals(mod.gen)
## W = 0.95426, p-value = 0.1063  
       bptest ( log (PH3_positive_cell)  ~   Diet  +  ( 1   /  Repeat), data =   subset (Length_Growth_PH3,Dday ==  "Shift Day 14" ))    
  ## 
##  studentized Breusch-Pagan test
## 
## data:  log(PH3_positive_cell) ~ Diet + (1/Repeat)
## BP = 0.20868, df = 1, p-value = 0.6478  
      mod.gen1  =   fitme ( log (PH3_positive_cell)  ~    1   +  ( 1   |  Repeat), data =   subset (Length_Growth_PH3,Dday ==  "Shift Day 14" )) 
   test  =   anova (mod.gen, mod.gen1)  
   Chi2_LRT_growth  =   2  * (mod.gen $ APHLs[[ "p_v" ]] - mod.gen1 $ APHLs[[ "p_v" ]]) 
    
   tab_stat  =   data.frame ( Comparison =   as.character ( paste ( "HS Day 14 vs HS to HY Day 21" )), 
                          Variable =   as.character ( paste ( "Shift Day 14" )), 
                                   Rep =   nlevels (Length_Growth_PH3 $ Repeat), 
                                   chi2_LR =   round ( as.numeric (test $ basicLRT $ chi2_LR),  digits =   2 ), 
                                   intercept =   format (mod.gen $ fixef[ 1 ], digits=  3 ), 
                                   estimate =   format (mod.gen $ fixef[ 2 ], digits=  3 ), 
                                   df =   as.numeric (test $ basicLRT $ df), 
                                   Pvalue =   as.numeric ( format ( pchisq (Chi2_LRT_growth, df=  1 , lower.tail =  F), digits=  2 ))) 
   tab_stat_14 = tab_stat 
    
    #Day 21  
   mod.gen  =   fitme ( log (PH3_positive_cell)  ~   Diet  +  ( 1   |  Repeat), data =   subset (Length_Growth_PH3,Dday ==  "Shift Day 21" )) 
    shapiro.test ( residuals (mod.gen))     
  ## 
##  Shapiro-Wilk normality test
## 
## data:  residuals(mod.gen)
## W = 0.93625, p-value = 0.005926  
       bptest ( log (PH3_positive_cell)  ~   Diet  +  ( 1   /  Repeat), data =   subset (Length_Growth_PH3,Dday ==  "Shift Day 21" ))    
  ## 
##  studentized Breusch-Pagan test
## 
## data:  log(PH3_positive_cell) ~ Diet + (1/Repeat)
## BP = 1.3963, df = 1, p-value = 0.2373  
      mod.gen1  =   fitme ( log (PH3_positive_cell)  ~    1   +  ( 1   |  Repeat), data =   subset (Length_Growth_PH3,Dday ==  "Shift Day 21" )) 
   test  =   anova (mod.gen, mod.gen1)  
   Chi2_LRT_growth  =   2  * (mod.gen $ APHLs[[ "p_v" ]] - mod.gen1 $ APHLs[[ "p_v" ]]) 
    
   tab_stat  =   data.frame ( Comparison =   as.character ( paste ( "HS Day 21 vs HS to HY Day 28" )), 
                          Variable =   as.character ( paste ( "Shift Day 21" )), 
                                   Rep =   nlevels (Length_Growth_PH3 $ Repeat), 
                                   chi2_LR =   round ( as.numeric (test $ basicLRT $ chi2_LR),  digits =   2 ), 
                                   intercept =   format (mod.gen $ fixef[ 1 ], digits=  3 ), 
                                   estimate =   format (mod.gen $ fixef[ 2 ], digits=  3 ), 
                                   df =   as.numeric (test $ basicLRT $ df), 
                                   Pvalue =   as.numeric ( format ( pchisq (Chi2_LRT_growth, df=  1 , lower.tail =  F), digits=  2 ))) 
   tab_stat_21  =  tab_stat 
    
   tab_stat =  rbind (tab_stat_7,tab_stat_14,tab_stat_21) 
   tab_stat $ sig  =   ifelse (tab_stat $ Pvalue  <   0.05   &  tab_stat $ Pvalue  >   0.01 ,  "*" , 
                 ifelse (tab_stat $ Pvalue  <   0.01   &  tab_stat $ Pvalue  >   0.001 ,  "**" , 
                  ifelse (tab_stat $ Pvalue  <   0.001 ,  "***" ,  "" ))) 
    
   tab_stat %>%  
      kable ( col.names =   c ( "Comparison" ,  "Variable" ,  "Replicates" ,  "Chi2" , "Intercept" , "Estimate" , "df"  , "p-value" , "Signif." ), row.names =   FALSE )  %>%     add_header_above ( c ( "log(PH3_positive_cell) ~  Diet + (1 | Repeat)"   =   9 )) %>%  
      kable_styling ( bootstrap_options =   c ( "striped" ,  "hover" ,  "condensed" ),  full_width =  F)    
 
 
 
 
 
log(PH3_positive_cell) ~ Diet + (1 | Repeat)
 
 
 
 
 
Comparison
 
 
Variable
 
 
Replicates
 
 
Chi2
 
 
Intercept
 
 
Estimate
 
 
df
 
 
p-value
 
 
Signif.
 
 
 
 
 
 
HS Day 7 vs HS to HY Day 14
 
 
Shift Day 7
 
 
3
 
 
28.80
 
 
2.07
 
 
1.38
 
 
1
 
 
1e-07
 
 
***
 
 
 
 
HS Day 14 vs HS to HY Day 21
 
 
Shift Day 14
 
 
3
 
 
24.15
 
 
2.26
 
 
1.12
 
 
1
 
 
9e-07
 
 
***
 
 
 
 
HS Day 21 vs HS to HY Day 28
 
 
Shift Day 21
 
 
3
 
 
45.01
 
 
2.57
 
 
1.29
 
 
1
 
 
0e+00
 
 
***
 
 
 
 
      tab_stat =  
     tab_stat  %>%  
     dplyr ::  rename ( Dday= Variable) %>%  
      as.data.frame () %>%  
      mutate ( Dday=  fct_relevel (Dday, "Shift Day 7" , "Shift Day 14" , "Shift Day 21" )) 
    
    ### Plot  
   Limits  =   c ( "HS" , "HStoHY" ) 
   z =  max (Length_Growth_PH3 $ PH3_positive_cell,  na.rm =   TRUE ) 
    
   Plot_Fig4C =  
      ggplot (Length_Growth_PH3,  aes ( x =  Diet,  y =  PH3_positive_cell)) +   
      geom_violin ( aes ( fill =  Diet),  draw_quantiles =   c ( 0.25 ,  0.5 ,  0.75 ),  colour =   "black" ,  size =   0.2 , adjust =   0.8 )  +  
      geom_dotplot (  colour =   "black" ,  fill =   "white" ,  binaxis =   "y" ,  stackdir =   "center" ,  binwidth =  z /  40 )  +   
      facet_grid (.  ~  Dday) +  
      geom_text ( data =  Sample_size,  mapping =   aes ( x =  Diet,  y =   -  5 ,  label =   paste ( "(" ,Sample_size, ")" , sep=  "" )), size=  3 ) +  
        geom_signif ( data =  tab_stat,  aes ( xmin =   1 ,  xmax =   2 ,  annotations =   formatC ( paste ( "p=" ,Pvalue),  digits =   2 ),  y_position =   132 ,),  textsize =   3 ,  vjust =   -  0.2 ,  manual =   TRUE ) +  
    
      scale_fill_manual ( limits=  c ( "HS" , "HStoHY" ), 
                        values= cbbHS_HStoHY) +  
      scale_x_discrete ( "" , 
                       limits= Limits, 
                       labels=  c ( "HS" , "HS to HY" )) +  
      scale_y_continuous ( expression ( paste ( "pH3"   ^   "+" ,  " cells" )), 
                         limits=  c ( -  8 , 136 ), 
                         breaks=  seq ( 0 , 120 , by=  20 )) +  
    stat_summary ( fun =  mean,  geom =   "point" ,  size =   3 ,  shape =   18 ,  colour =   "black" ,  aes ( group =  Repeat))  +  
                         stat_summary ( fun =  mean,  geom =   "point" ,  size =   2 ,  shape =   18 ,  aes ( group =  Repeat,  colour =  Repeat))  +  
                         scale_color_manual ( values =  palette_mean)  +  
      theme ( panel.grid.major.y =   element_line ( colour =   grey ( 0.45 ),  linetype =   "dashed" ,  size =   0.2 ), 
            panel.background =   element_blank (), 
            axis.title.x =   element_text ( size= Smallfont, colour=  "black" ), 
            axis.title.y =   element_text ( size= Smallfont, colour=  "black" ),  
            axis.line.x =   element_line ( colour=  "black" , size=  0.75 ), 
            axis.line.y =   element_line ( colour=  "black" , size=  0.75 ), 
            axis.ticks.x =   element_line ( size =   0.75 ), 
            axis.ticks.y =   element_line ( size =   0.75 ), 
            axis.text.x =   element_text ( size= Smallfont, colour=  "black" , angle=  30 , hjust=  1 ), 
            axis.text.y =   element_text ( size= Smallfont, colour=  "black" ), 
            plot.margin =   unit (Margin,  "cm" ), 
            legend.direction =   "vertical" ,  
            legend.box =   "horizontal" , 
            legend.position =   "none" , 
            legend.key.height =   unit ( 0.4 ,  "cm" ), 
            legend.key.width=   unit ( 0.6 ,  "cm" ), 
            legend.title =   element_text ( face=  "italic" , size= Smallfont),  
            legend.key =   element_rect ( colour =   'white' ,  fill =   "white" ,  linetype=  'dashed' ), 
            legend.text =   element_text ( size= SuperSmallfont), 
            legend.background =   element_rect ( fill=  NA ), 
                strip.text.x =   element_text ( size =  Smallfont,  colour =   "black" ,  margin =   margin ( t =   2 ,  r =   0 ,  b =   2 ,  l =   0 )), 
            strip.text.y =   element_text ( size =  Smallfont,  colour =   "black" ,  margin =   margin ( t =   2 ,  r =   0 ,  b =   2 ,  l =   0 )), 
            strip.background =   element_rect ( fill=  NA ,  colour=  "black" ), 
            strip.placement=  "outside" ) 
    
   Plot_Fig4C    
   
 
 
  4.1.4  Figure 4D 
 
 Shifting between diets impacts pH3+ cell number in shrinkage (HY to HS) experiments. Statistical comparisons are vs pre-shift measurement. 
 
      Length_shrinkage_PH3  =   
     d[[ "4C', 4S1D'" ]] %>%  
      mutate_at ( vars ( ends_with ( ".L" )), ~ . /  1000 ) %>%  
      mutate_at ( vars ( !  starts_with ( "Total" )),as.factor) %>%  
     dplyr ::  rename ( Total_Length_mm= Total.L, 
             PH3_positive_cell= Total.PH3, 
             Day_of_treatment= Day) %>%  
      as.data.frame () %>%  
      mutate ( Dday=  fct_relevel (Dday, "Shift Day 7" , "Shift Day 14" , "Shift Day 21" )) 
    
   Sample_size =  
     Length_shrinkage_PH3 %>%  
      group_by (Diet,Dday) %>%  
      summarise ( Sample_size=  n ()) 
    
    ###Stats  
    ###Day 7  
   mod.gen  =   fitme ( log (PH3_positive_cell)  ~   Diet  +  ( 1   |  Repeat), data =   subset (Length_shrinkage_PH3,Dday ==  "Shift Day 7" )) 
    shapiro.test ( residuals (mod.gen))     
  ## 
##  Shapiro-Wilk normality test
## 
## data:  residuals(mod.gen)
## W = 0.96318, p-value = 0.1917  
       bptest ( log (PH3_positive_cell)  ~   Diet  +  ( 1   /  Repeat), data =   subset (Length_shrinkage_PH3,Dday ==  "Shift Day 7" ))    
  ## 
##  studentized Breusch-Pagan test
## 
## data:  log(PH3_positive_cell) ~ Diet + (1/Repeat)
## BP = 1.9935, df = 1, p-value = 0.158  
      mod.gen1  =   fitme ( log (PH3_positive_cell)  ~    1   +  ( 1   |  Repeat), data =   subset (Length_shrinkage_PH3,Dday ==  "Shift Day 7" )) 
   test  =   anova (mod.gen, mod.gen1)  
   Chi2_LRT_shrinkage  =   2  * (mod.gen $ APHLs[[ "p_v" ]] - mod.gen1 $ APHLs[[ "p_v" ]]) 
    
   tab_stat  =   data.frame ( Comparison =   as.character ( paste ( "HS Day 7 vs HS to HY Day 14" )), 
                          Variable =   as.character ( paste ( "Shift Day 7" )), 
                                   Rep =   nlevels (Length_shrinkage_PH3 $ Repeat), 
                                   chi2_LR =   round ( as.numeric (test $ basicLRT $ chi2_LR),  digits =   2 ), 
                                   intercept =   format (mod.gen $ fixef[ 1 ], digits=  3 ), 
                                   estimate =   format (mod.gen $ fixef[ 2 ], digits=  3 ), 
                                   df =   as.numeric (test $ basicLRT $ df), 
                                   Pvalue =   as.numeric ( format ( pchisq (Chi2_LRT_shrinkage, df=  1 , lower.tail =  F), digits=  1 , scientific= F))) 
   tab_stat_7 = tab_stat 
    
    #Day 14  
   mod.gen  =   fitme ( log (PH3_positive_cell)  ~   Diet  +  ( 1   |  Repeat), data =   subset (Length_shrinkage_PH3,Dday ==  "Shift Day 14" )) 
    shapiro.test ( residuals (mod.gen))     
  ## 
##  Shapiro-Wilk normality test
## 
## data:  residuals(mod.gen)
## W = 0.88271, p-value = 0.0008579  
       bptest ( log (PH3_positive_cell)  ~   Diet  +  ( 1   /  Repeat), data =   subset (Length_shrinkage_PH3,Dday ==  "Shift Day 14" ))    
  ## 
##  studentized Breusch-Pagan test
## 
## data:  log(PH3_positive_cell) ~ Diet + (1/Repeat)
## BP = 0.31605, df = 1, p-value = 0.574  
      mod.gen1  =   fitme ( log (PH3_positive_cell)  ~    1   +  ( 1   |  Repeat), data =   subset (Length_shrinkage_PH3,Dday ==  "Shift Day 14" )) 
   test  =   anova (mod.gen, mod.gen1)  
   Chi2_LRT_shrinkage  =   2  * (mod.gen $ APHLs[[ "p_v" ]] - mod.gen1 $ APHLs[[ "p_v" ]]) 
    
   tab_stat  =   data.frame ( Comparison =   as.character ( paste ( "HS Day 14 vs HS to HY Day 21" )), 
                          Variable =   as.character ( paste ( "Shift Day 14" )), 
                                   Rep =   nlevels (Length_shrinkage_PH3 $ Repeat), 
                                   chi2_LR =   round ( as.numeric (test $ basicLRT $ chi2_LR),  digits =   2 ), 
                                   intercept =   format (mod.gen $ fixef[ 1 ], digits=  3 ), 
                                   estimate =   format (mod.gen $ fixef[ 2 ], digits=  3 ), 
                                   df =   as.numeric (test $ basicLRT $ df), 
                                   Pvalue =   as.numeric ( format ( pchisq (Chi2_LRT_shrinkage, df=  1 , lower.tail =  F), digits=  1 ))) 
   tab_stat_14 = tab_stat 
    
    #Day 21  
   mod.gen  =   fitme ( log (PH3_positive_cell)  ~   Diet  +  ( 1   |  Repeat), data =   subset (Length_shrinkage_PH3,Dday ==  "Shift Day 21" )) 
    shapiro.test ( residuals (mod.gen))     
  ## 
##  Shapiro-Wilk normality test
## 
## data:  residuals(mod.gen)
## W = 0.97516, p-value = 0.3966  
       bptest ( log (PH3_positive_cell)  ~   Diet  +  ( 1   /  Repeat), data =   subset (Length_shrinkage_PH3,Dday ==  "Shift Day 21" ))    
  ## 
##  studentized Breusch-Pagan test
## 
## data:  log(PH3_positive_cell) ~ Diet + (1/Repeat)
## BP = 0.66778, df = 1, p-value = 0.4138  
      mod.gen1  =   fitme ( log (PH3_positive_cell)  ~    1   +  ( 1   |  Repeat), data =   subset (Length_shrinkage_PH3,Dday ==  "Shift Day 21" )) 
   test  =   anova (mod.gen, mod.gen1)  
   Chi2_LRT_shrinkage  =   2  * (mod.gen $ APHLs[[ "p_v" ]] - mod.gen1 $ APHLs[[ "p_v" ]]) 
    
   tab_stat  =   data.frame ( Comparison =   as.character ( paste ( "HS Day 21 vs HS to HY Day 28" )), 
                          Variable =   as.character ( paste ( "Shift Day 21" )), 
                                   Rep =   nlevels (Length_shrinkage_PH3 $ Repeat), 
                                   chi2_LR =   round ( as.numeric (test $ basicLRT $ chi2_LR),  digits =   2 ), 
                                   intercept =   format (mod.gen $ fixef[ 1 ], digits=  3 ), 
                                   estimate =   format (mod.gen $ fixef[ 2 ], digits=  3 ), 
                                   df =   as.numeric (test $ basicLRT $ df), 
                                   Pvalue =   as.numeric ( format ( pchisq (Chi2_LRT_shrinkage, df=  1 , lower.tail =  F), digits=  2 ))) 
   tab_stat_21  =  tab_stat 
    
   tab_stat =  rbind (tab_stat_7,tab_stat_14,tab_stat_21) 
   tab_stat $ sig  =   ifelse (tab_stat $ Pvalue  <   0.05   &  tab_stat $ Pvalue  >   0.01 ,  "*" , 
                 ifelse (tab_stat $ Pvalue  <   0.01   &  tab_stat $ Pvalue  >   0.001 ,  "**" , 
                  ifelse (tab_stat $ Pvalue  <   0.001 ,  "***" ,  "" ))) 
   tab_stat %>%  
      kable ( col.names =   c ( "Comparison" ,  "Variable" ,  "Replicates" ,  "Chi2" , "Intercept" , "Estimate" , "df"  , "p-value" , "Signif." ), row.names =   FALSE )  %>%     add_header_above ( c ( "log(PH3_positive_cell) ~  Diet + (1 | Repeat)"   =   9 )) %>%  
      kable_styling ( bootstrap_options =   c ( "striped" ,  "hover" ,  "condensed" ),  full_width =  F)    
 
 
 
 
 
log(PH3_positive_cell) ~ Diet + (1 | Repeat)
 
 
 
 
 
Comparison
 
 
Variable
 
 
Replicates
 
 
Chi2
 
 
Intercept
 
 
Estimate
 
 
df
 
 
p-value
 
 
Signif.
 
 
 
 
 
 
HS Day 7 vs HS to HY Day 14
 
 
Shift Day 7
 
 
3
 
 
1.93
 
 
3.56
 
 
-0.25
 
 
1
 
 
2.0e-01
 
 
 
 
 
 
HS Day 14 vs HS to HY Day 21
 
 
Shift Day 14
 
 
3
 
 
13.65
 
 
3.98
 
 
-0.74
 
 
1
 
 
2.0e-04
 
 
***
 
 
 
 
HS Day 21 vs HS to HY Day 28
 
 
Shift Day 21
 
 
3
 
 
20.21
 
 
4.18
 
 
-0.59
 
 
1
 
 
6.9e-06
 
 
***
 
 
 
 
      tab_stat =  
     tab_stat  %>%  
     dplyr ::  rename ( Dday= Variable) %>%  
      as.data.frame () %>%  
      mutate ( Dday=  fct_relevel (Dday, "Shift Day 7" , "Shift Day 14" , "Shift Day 21" )) 
    
    ### Plot  
   Limits  =   c ( "HY" , "HYtoHS" ) 
   z  =   max (Length_shrinkage_PH3 $ PH3_positive_cell,  na.rm =   TRUE ) 
    
   Plot_Fig4D =  
      ggplot (Length_shrinkage_PH3,  aes ( x =  Diet,  y =  PH3_positive_cell)) +   
      geom_violin ( aes ( fill =  Diet),  draw_quantiles =   c ( 0.25 ,  0.5 ,  0.75 ),  colour =   "black" ,  size =   0.2 , adjust =   0.8 )  +  
      geom_dotplot (  colour =   "black" ,  fill =   "white" ,  binaxis =   "y" ,  stackdir =   "center" ,  binwidth =  z /  40 )  +   
      facet_grid (.  ~  Dday) +  
      geom_text ( data =  Sample_size,  mapping =   aes ( x =  Diet,  y =   -  5 ,  label =   paste ( "(" ,Sample_size, ")" , sep=  "" )), size=  3 ) +  
          geom_signif ( data =  tab_stat,  aes ( xmin =   1 ,  xmax =   2 ,  annotations =   formatC ( paste ( "p=" ,Pvalue),  digits =   2 ),  y_position =   132 ,),  textsize =   3 ,  vjust =   -  0.2 ,  manual =   TRUE ) +  
    
      scale_fill_manual ( limits=  c ( "HY" , "HYtoHS" ), 
                        values= cbbHY_HYtoHS) +  
      scale_x_discrete ( "" , 
                       limits= Limits, 
                       labels=  c ( "HY" , "HY to HS" )) +  
      scale_y_continuous ( expression ( paste ( "pH3"   ^   "+" ,  " cells" )), 
                         limits=  c ( -  8 , 136 ), 
                         breaks=  seq ( 0 , 120 , by=  20 )) +  
      stat_summary ( fun =  mean,  geom =   "point" ,  size =   3 ,  shape =   18 ,  colour =   "black" ,  aes ( group =  Repeat))  +  
                         stat_summary ( fun =  mean,  geom =   "point" ,  size =   2 ,  shape =   18 ,  aes ( group =  Repeat,  colour =  Repeat))  +  
                         scale_color_manual ( values =  palette_mean)  +  
      theme ( panel.grid.major.y =   element_line ( colour =   grey ( 0.45 ),  linetype =   "dashed" ,  size =   0.2 ), 
            panel.background =   element_blank (), 
            axis.title.x =   element_text ( size= Smallfont, colour=  "black" ), 
            axis.title.y =   element_text ( size= Smallfont, colour=  "black" ),  
            axis.line.x =   element_line ( colour=  "black" , size=  0.75 ), 
            axis.line.y =   element_line ( colour=  "black" , size=  0.75 ), 
            axis.ticks.x =   element_line ( size =   0.75 ), 
            axis.ticks.y =   element_line ( size =   0.75 ), 
            axis.text.x =   element_text ( size= Smallfont, colour=  "black" , angle=  30 , hjust=  1 ), 
            axis.text.y =   element_text ( size= Smallfont, colour=  "black" ), 
            plot.margin =   unit (Margin,  "cm" ), 
            legend.direction =   "vertical" ,  
            legend.box =   "horizontal" , 
            legend.position =   "none" , 
            legend.key.height =   unit ( 0.4 ,  "cm" ), 
            legend.key.width=   unit ( 0.6 ,  "cm" ), 
            legend.title =   element_text ( face=  "italic" , size= Smallfont),  
            legend.key =   element_rect ( colour =   'white' ,  fill =   "white" ,  linetype=  'dashed' ), 
            legend.text =   element_text ( size= SuperSmallfont), 
            legend.background =   element_rect ( fill=  NA ), 
            strip.text.x =   element_text ( size =  Smallfont,  colour =   "black" ,  margin =   margin ( t =   2 ,  r =   0 ,  b =   2 ,  l =   0 )), 
            strip.text.y =   element_text ( size =  Smallfont,  colour =   "black" ,  margin =   margin ( t =   2 ,  r =   0 ,  b =   2 ,  l =   0 )), 
            strip.background =   element_rect ( fill=  NA ,  colour=  "black" ), 
            strip.placement=  "outside" ) 
    
   Plot_Fig4D    
   
 
 
  4.1.5  Figure 4E-F 
 
 Clonal assay with EsgF/O system illustrates increased number of marked cells on HY (F) vs HS (E) diets 5 days post-eclosion in region 4 of the midgut. GFP, in green, marks all cells made since the EsgF/O system was activated.  Complete graphical annotation can be found in manuscript figures  
 
 
 
   
 
 
   
 
 
 
 
  4.1.6  Figure 4G-H-I-J 
 
  Cell loss assay enables analysis of the impact of diet composition on replacement ratio and rate.  Description of experimental design is found in materials and methods and illustrated in figure 4–figure supplement 1H. In brief, this assay allows us to mark ECs and EBs at the start of the experiment and to count their numbers 14 days after shifting dietary conditions recapitulating growth and shrinkage of the midgut, thus estimating cell gain and cell loss in these conditions. Representative pictures for the cell loss assay in growing conditions (G, H, top row) and shrinkage conditions (I, J, bottom row).  Complete graphical annotation can be found in manuscript figures  
 
 
 
   
 
 
   
 
 
 
 
   
 
 
   
 
 
 
 
  4.1.7  Figure 4K 
 
 In red 5966GS> His-RFP, marking EB and EC. Number of ECs in the posterior midgut, both marked (Red, old ECs) and unmarked (Blue, new ECs) by RFP, error bars are SE from 3 repeats. 
 
      tab_prop_cell_RFP  =   
     d[[ "4K" ]] %>%  
      mutate_if (is.character,as.factor) %>%  
      mutate_if (is.integer,as.factor) %>%  
      mutate ( Post.Dapi.Number =  (Post.Dapi.Number) *  2 ) %>%  
      mutate ( Post.RFP.Number =  (Post.RFP.Number) *  2 ) %>%  
      mutate ( Post.NonRFP.Number =  (Post.Dapi.Number  -  Post.RFP.Number)) %>%  
      group_by (Day, Diet, Diet1, Experiment) %>%  
      summarise ( mean_RFP_positive=  mean (Post.RFP.Number, na.rm= T), 
                se_RFP_positive=  se (Post.RFP.Number), 
                mean_RFP_negative=  mean (Post.NonRFP.Number, na.rm= T), 
                se_RFP_negative=  se (Post.NonRFP.Number)) %>%  
      mutate ( group=  paste (Diet1,Experiment, sep=  "_" )) %>%  
      as.data.frame () 
    
    attach (tab_prop_cell_RFP) 
    for  (i  in   1  :  length (Experiment)){ 
       tab_prop_cell_RFP $ se_proportionGraphPlus_pos[i]  =  mean_RFP_positive[i] + se_RFP_positive[i] 
       tab_prop_cell_RFP $ se_proportionGraphMinus_pos[i]  =  mean_RFP_positive[i] - se_RFP_positive[i] 
       tab_prop_cell_RFP $ se_proportionGraphPlus_neg[i]  =  mean_RFP_positive[i] + mean_RFP_negative[i] + se_RFP_negative[i] 
       tab_prop_cell_RFP $ se_proportionGraphMinus_neg[i]  =  mean_RFP_positive[i] + mean_RFP_negative[i] - se_RFP_negative[i] 
   } 
    
   tmp1  =  tab_prop_cell_RFP[, c ( "Day" ,  "Diet" ,  "Diet1" ,  "Experiment" , "mean_RFP_positive" ,  "se_RFP_positive" , "se_proportionGraphPlus_pos" ,  "se_proportionGraphMinus_pos" )] 
   tmp1 $ RFP =  "Positive"  
   tmp1 = dplyr ::  rename (tmp1, mean_RFP =  mean_RFP_positive, 
                se_RFP =  se_RFP_positive, 
                se_proportionGraphPlus = se_proportionGraphPlus_pos, 
                se_proportionGraphMinus=  se_proportionGraphMinus_pos) 
    
   tmp2  =  tab_prop_cell_RFP[, c ( "Day" ,  "Diet" ,  "Diet1" ,  "Experiment" , "mean_RFP_negative" ,  "se_RFP_negative" ,  "se_proportionGraphMinus_neg"  , "se_proportionGraphPlus_neg" )] 
   tmp2 $ RFP =  "Negative"  
   tmp2 = dplyr ::  rename (tmp2, mean_RFP =  mean_RFP_negative,  
                se_RFP =  se_RFP_negative, 
                se_proportionGraphPlus = se_proportionGraphPlus_neg, 
                se_proportionGraphMinus=  se_proportionGraphMinus_neg) 
     
   tab_prop_cell_RFP  =   rbind (tmp1,tmp2) 
   tab_prop_cell_RFP  =   
     tab_prop_cell_RFP %>%  
      mutate_if (is.numeric,round, 0 ) %>%  
      mutate_if (is.character,as.factor) 
    
    #tab_prop_cell_RFP$Experiment = as.factor(ifelse(tab_prop_cell_RFP$Day=="0" & tab_prop_cell_RFP$Diet1=="HS","HS",  
                                          #ifelse(tab_prop_cell_RFP$Day=="0" & tab_prop_cell_RFP$Diet1=="HY","HY",   
                                                # as.character(tab_prop_cell_RFP$Experiment))))  
    
    #tab_prop_cell_RFP=  
      #tab_prop_cell_RFP%>%  
      #as.data.frame()%>%  
      #mutate(Diet1=fct_relevel(Diet1,"HS", "HStoHS" , "HStoHY", "HY", "HYtoHS", "HYtoHY"),  
             #Experiment=fct_relevel(Experiment, "HS", "Growth", "HY", "Shrinkage"))  
    
    levels (tab_prop_cell_RFP $ Diet1)  <-   c ( "HS" ,  "HS to HS"  ,  "HS to HY" ,  "HY" ,  "HY to HS" ,  "HY to HY" ) 
    
   Sample_size =  
       d[[ "4K" ]] %>%  
      mutate_if (is.character,as.factor) %>%  
      mutate_if (is.integer,as.factor) %>%  
      group_by (Diet1) %>%  
      summarise ( Sample_size=  n ()) 
    
   Sample_size $ Experiment  =   c ( "G" ,  "G" ,  "G" ,  "S" ,  "S" ,  "S" ) 
    
    levels (Sample_size $ Diet1)  <-   c ( "HS" ,  "HS to HS"  ,  "HS to HY" ,  "HY" ,  "HY to HS" ,  "HY to HY" ) 
    
                                      
   Plot_Fig4K  =  
      ggplot (tab_prop_cell_RFP,  aes ( x= Diet1,  y= mean_RFP)) +   
      geom_bar ( stat=  "identity" , aes ( fill= RFP), color=  "black" , width= . 90 ) +  
      geom_errorbar ( aes ( ymin=  se_proportionGraphMinus,  ymax=  se_proportionGraphPlus), width=  0.25 ) +  
       geom_text ( data =  Sample_size,  mapping =   aes ( x =  Diet1,  y =   200 ,  label =   paste ( "(" ,Sample_size, ")" , sep=  "" )), size=  3 ) +  
      facet_wrap (. ~ Experiment, scales=  "free_x" ) +  
      scale_fill_manual ( name =   "RFP labelling" ,  
                        values=  c ( "#3a5ecc" , "#cc0000" ), 
                        labels =   c ( "Negative" ,  "Positive" )) +  
      scale_y_continuous ( "Number of cells (mean \u00B1se)" , 
                         limits=  c ( 0 , 5800 ), 
                         breaks=  seq ( 0 , 5000 , by=  500 )) +  
      theme ( 
        panel.grid.major.y =   element_line ( colour =   grey ( 0.45 ),  linetype =   "dashed" ,  size =   0.2 ), 
        panel.background =   element_blank (), 
        axis.title.x =   element_blank (), 
        axis.title.y =   element_text ( size= Smallfont, colour=  "black" ),  
        axis.line.x =   element_line ( colour=  "black" , size=  0.75 ), 
        axis.line.y =   element_line ( colour=  "black" , size=  0.75 ), 
        axis.ticks.x =   element_line ( size =   0.75 ), 
        axis.ticks.y =   element_line ( size =   0.75 ), 
        axis.text.x =   element_text ( size= Smallfont, colour=  "black" , angle=  30 , hjust=  1 ), 
        axis.text.y =   element_text ( size= Smallfont, colour=  "black" ), 
        plot.margin =   unit (Margin,  "cm" ), 
        legend.direction =   "vertical" ,  
        legend.box =   "horizontal" , 
        legend.position =   c ( 0.2 , 0.87 ), 
        legend.key.height =   unit ( 0.4 ,  "cm" ), 
        legend.key.width=   unit ( 0.6 ,  "cm" ), 
        legend.title =   element_text ( face=  "italic" , size= Smallfont),  
        legend.key =   element_rect ( colour =   'white' ,  fill =   "white" ,  linetype=  'dashed' ), 
        legend.text =   element_text ( size= Smallfont), 
        legend.background =   element_rect ( fill=  NA ), 
        strip.text =   element_blank ()) 
        #strip.background = element_rect(fill=NA, colour="black"),  
        #strip.placement="outside")  
    
   Plot_Fig4K    
   
 
 
  4.1.8  Figure 4L 
 
 Data shown as rate relative to experiment start (cell /initial EC/ day). Number on bar in red is ratio of EC gained/EC lost (see materials and methods for formula). 
 
      tab_relative_cell_rate  =   
     d[[ "4L, 4S2D" ]] %>%  
      select ( -  starts_with ( "X" )) %>%  
      drop_na () %>%  
      mutate_if (is.character,as.factor) %>%  
      group_by (Diet1, Experiment, Experiment2, GL) %>%  
      summarise ( mean_RelativeRate=  mean (RelativeRate, na.rm= T), 
                se_RelativeRate=  se (RelativeRate)) %>%  
                mutate ( group=  paste (Diet1,Experiment, sep=  "_" )) %>%  
      as.data.frame () 
    
    
    
    
    
    
   tab_relative_cell_rate_ratio  =   
     tab_relative_cell_rate %>%  
      group_by (Diet1,Experiment2) %>%  
      summarize ( Ratio =   round (mean_RelativeRate[GL  ==   "Gain" ]  /  ( - mean_RelativeRate[GL  ==   "Loss" ]), 2 )) %>%  
      mutate ( GL=  "Loss" ) 
    
    levels (tab_relative_cell_rate $ Diet1)  <-   c ( "HS to HS"  ,  "HS to HY" ,  "HY to HS" ,  "HY to HY" ) 
    levels (tab_relative_cell_rate_ratio $ Diet1)  <-   c ( "HS to HS"  ,  "HS to HY" ,  "HY to HS" ,  "HY to HY" ) 
    
    
    
    
    
   Plot_Fig4L  =  
      ggplot (tab_relative_cell_rate,  aes ( x =  Diet1,  y =  mean_RelativeRate,  fill =  GL)) +   
      geom_bar ( stat=  "identity" , aes ( fill= GL), color=  "black" , width= . 90 ) +  
      geom_hline ( yintercept =   0 ) +  
      geom_text ( data= tab_relative_cell_rate_ratio, mapping=  aes ( x= Diet1, y=  -  0.01 , label= Ratio),  color =   "red" ) +  
      facet_grid (. ~ Experiment2, scales=  "free_x" ) +  
    
      scale_fill_manual ( name =   "Enterocyte" ,  
                        values=  c ( "palegreen" ,  "moccasin" ), 
                        labels =   c ( "Gain" ,  "Loss" )) +  
      scale_y_continuous ( "Cell rate (cell/ initialEC/ day)" , 
                         limits=  c ( -  0.11 , 0.11 ), 
                         breaks=  seq ( -  0.2 , 0.2 , by=  0.05 )) +  
      theme ( 
        panel.grid.major.y =   element_line ( colour =   grey ( 0.45 ),  linetype =   "dashed" ,  size =   0.2 ), 
        panel.background =   element_blank (), 
        axis.title.x =   element_blank (), 
        axis.title.y =   element_text ( size= Smallfont, colour=  "black" ),  
        axis.line.x =   element_line ( colour=  "black" , size=  0.75 ), 
        axis.line.y =   element_line ( colour=  "black" , size=  0.75 ), 
        axis.ticks.x =   element_line ( size =   0.75 ), 
        axis.ticks.y =   element_line ( size =   0.75 ), 
        axis.text.x =   element_text ( size= Smallfont, colour=  "black" , angle=  30 , hjust=  1 ), 
        axis.text.y =   element_text ( size= Smallfont, colour=  "black" ), 
        plot.margin =   unit (Margin,  "cm" ), 
        legend.direction =   "vertical" ,  
        legend.box =   "horizontal" , 
        legend.position =   c ( 0.18 , 0.83 ), 
        legend.key.height =   unit ( 0.4 ,  "cm" ), 
        legend.key.width=   unit ( 0.6 ,  "cm" ), 
        legend.title =   element_text ( face=  "italic" , size= Smallfont),  
        legend.key =   element_rect ( colour =   'white' ,  fill =   "white" ,  linetype=  'dashed' ), 
        legend.text =   element_text ( size= Smallfont), 
        legend.background =   element_rect ( fill=  NA ), 
        strip.text.x =   element_text ( size =  Smallfont,  colour =   "black" ,  margin =   margin ( t =   2 ,  r =   0 ,  b =   2 ,  l =   0 )), 
        strip.text.y =   element_text ( size =  Smallfont,  colour =   "black" ,  margin =   margin ( t =   2 ,  r =   0 ,  b =   2 ,  l =   0 )), 
        strip.background =   element_rect ( fill=  NA ,  colour=  "black" ), 
        strip.placement=  "outside" ) 
    
    
   Plot_Fig4L    
   
 ##Export Figure 4 
 
 
 
  4.2  Figure 4 - supplementary 1 
 
  4.2.1  Figure 4S1A 
 
 Midgut length increases progressively on HY, but not on HS. Statistics compare HS vs HY for each day, *** = p<0.01. 
 
      Length_dayseclosion  =   
     d[[ "4 - S1A" ]] %>%  
      select ( -  starts_with ( "X" )) %>%  
      mutate_at ( vars ( starts_with ( "Total" )), ~ . /  1000 ) %>%  
      mutate_if (is.character,as.factor) %>%  
      mutate_if (is.integer,as.factor) %>%  
     dplyr ::  rename ( Total_Length_mm= Total.Length) %>%  
      mutate ( group=  paste (TreatCol,Day, sep=  "_" )) 
    
   Sample_size =  
     Length_dayseclosion %>%  
      group_by (Day,TreatCol) %>%  
      summarise ( Sample_size=  n ()) %>%  
     dplyr ::  rename ( Diet= TreatCol) 
    
    #Stats4S1A  
    ###Stats  
    
    #Day 1  
   tmp  =   subset (Length_dayseclosion, Day  ==   "1" )  %>%  
        mutate ( Day =   as.factor (Day)) 
    
   mod.gen  =   fitme ( log (Total_Length_mm)  ~  Diet  +  ( 1   |  Repeat),  data =  tmp) 
    shapiro.test ( residuals (mod.gen))    
  ## 
##  Shapiro-Wilk normality test
## 
## data:  residuals(mod.gen)
## W = 0.97708, p-value = 0.614  
       bptest ( log (Total_Length_mm)  ~  Diet  +  ( 1   /  Repeat),  data =  tmp)    
  ## 
##  studentized Breusch-Pagan test
## 
## data:  log(Total_Length_mm) ~ Diet + (1/Repeat)
## BP = 0.1261, df = 1, p-value = 0.7225  
      mod.gen1  =   fitme ( log (Total_Length_mm)  ~   1   +  ( 1   |  Repeat),  data =  tmp) 
   test  =   anova (mod.gen, mod.gen1) 
   Chi2_LRT_growth  =   2   *  (mod.gen $ APHLs[[ "p_v" ]]  -  mod.gen1 $ APHLs[[ "p_v" ]]) 
    
   tab_stat  =   data.frame ( Variable =   as.character ( "1" ), 
                          Rep =   nlevels (tmp $ Repeat), 
                          chi2_LR =   round ( as.numeric (test $ basicLRT $ chi2_LR),  digits =   2 ), 
                          intercept =   format (mod.gen $ fixef[ 1 ], digits=  3 ), 
                          estimate =   format (mod.gen $ fixef[ 2 ], digits=  3 ), 
                          df =   as.numeric (test $ basicLRT $ df), 
                          Pvalue =   as.numeric ( format ( pchisq (Chi2_LRT_growth, df=  1 , lower.tail =  F), digits=  2 ))) 
   tab_stat_day_1  =  tab_stat 
    
    #Day 2  
   tmp  =   subset (Length_dayseclosion, Day  ==   "2" )  %>%  
        mutate ( Day =   as.factor (Day)) 
    
   mod.gen  =   fitme ( log (Total_Length_mm)  ~  Diet  +  ( 1   |  Repeat),  data =  tmp) 
    shapiro.test ( residuals (mod.gen))    
  ## 
##  Shapiro-Wilk normality test
## 
## data:  residuals(mod.gen)
## W = 0.99417, p-value = 0.9992  
       bptest ( log (Total_Length_mm)  ~  Diet  +  ( 1   /  Repeat),  data =  tmp)    
  ## 
##  studentized Breusch-Pagan test
## 
## data:  log(Total_Length_mm) ~ Diet + (1/Repeat)
## BP = 1.0007, df = 1, p-value = 0.3171  
      mod.gen1  =   fitme ( log (Total_Length_mm)  ~   1   +  ( 1   |  Repeat),  data =  tmp) 
   test  =   anova (mod.gen, mod.gen1) 
   Chi2_LRT_growth  =   2   *  (mod.gen $ APHLs[[ "p_v" ]]  -  mod.gen1 $ APHLs[[ "p_v" ]]) 
    
   tab_stat  =   data.frame ( Variable =   as.character ( "2" ), 
                          Rep =   nlevels (tmp $ Repeat), 
                          chi2_LR =   round ( as.numeric (test $ basicLRT $ chi2_LR),  digits =   2 ), 
                          intercept =   format (mod.gen $ fixef[ 1 ], digits=  3 ), 
                          estimate =   format (mod.gen $ fixef[ 2 ], digits=  3 ), 
                          df =   as.numeric (test $ basicLRT $ df), 
                          Pvalue =   as.numeric ( format ( pchisq (Chi2_LRT_growth, df=  1 , lower.tail =  F), digits=  2 ))) 
   tab_stat_day_2  =  tab_stat 
    
    #Day 3  
   tmp  =   subset (Length_dayseclosion, Day  ==   "3" )  %>%  
        mutate ( Day =   as.factor (Day)) 
    
   mod.gen  =   fitme ( log (Total_Length_mm)  ~  Diet  +  ( 1   |  Repeat),  data =  tmp) 
    shapiro.test ( residuals (mod.gen))    
  ## 
##  Shapiro-Wilk normality test
## 
## data:  residuals(mod.gen)
## W = 0.98604, p-value = 0.8874  
       bptest ( log (Total_Length_mm)  ~  Diet  +  ( 1   /  Repeat),  data =  tmp)    
  ## 
##  studentized Breusch-Pagan test
## 
## data:  log(Total_Length_mm) ~ Diet + (1/Repeat)
## BP = 4.4343, df = 1, p-value = 0.03522  
      mod.gen1  =   fitme ( log (Total_Length_mm)  ~   1   +  ( 1   |  Repeat),  data =  tmp) 
   test  =   anova (mod.gen, mod.gen1) 
    
   Chi2_LRT_growth  =   2   *  (mod.gen $ APHLs[[ "p_v" ]]  -  mod.gen1 $ APHLs[[ "p_v" ]]) 
    
   tab_stat  =   data.frame ( Variable =   as.character ( "3" ), 
                          Rep =   nlevels (tmp $ Repeat), 
                          chi2_LR =   round ( as.numeric (test $ basicLRT $ chi2_LR),  digits =   2 ), 
                          intercept =   format (mod.gen $ fixef[ 1 ], digits=  3 ), 
                          estimate =   format (mod.gen $ fixef[ 2 ], digits=  3 ), 
                          df =   as.numeric (test $ basicLRT $ df), 
                          Pvalue =   as.numeric ( format ( pchisq (Chi2_LRT_growth, df=  1 , lower.tail =  F), digits=  2 ))) 
   tab_stat_day_3  =  tab_stat 
    
    #Day 4  
   tmp  =   subset (Length_dayseclosion, Day  ==   "4" )  %>%  
      mutate ( Day =   as.factor (Day)) 
    
   mod.gen  =   fitme ( log (Total_Length_mm)  ~  Diet  +  ( 1   |  Repeat),  data =  tmp) 
    shapiro.test ( residuals (mod.gen))    
  ## 
##  Shapiro-Wilk normality test
## 
## data:  residuals(mod.gen)
## W = 0.98212, p-value = 0.7675  
       bptest ( log (Total_Length_mm)  ~  Diet  +  ( 1   /  Repeat),  data =  tmp)    
  ## 
##  studentized Breusch-Pagan test
## 
## data:  log(Total_Length_mm) ~ Diet + (1/Repeat)
## BP = 2.4272, df = 1, p-value = 0.1192  
      mod.gen1  =   fitme ( log (Total_Length_mm)  ~   1   +  ( 1   |  Repeat),  data =  tmp) 
   test  =   anova (mod.gen, mod.gen1) 
   Chi2_LRT_growth  =   2   *  (mod.gen $ APHLs[[ "p_v" ]]  -  mod.gen1 $ APHLs[[ "p_v" ]]) 
    
   tab_stat  =   data.frame ( Variable =   as.character ( paste ( "4" )), 
                          Rep =   nlevels (tmp $ Repeat), 
                          chi2_LR =   round ( as.numeric (test $ basicLRT $ chi2_LR),  digits =   2 ), 
                          intercept =   format (mod.gen $ fixef[ 1 ], digits=  3 ), 
                          estimate =   format (mod.gen $ fixef[ 2 ], digits=  3 ), 
                          df =   as.numeric (test $ basicLRT $ df), 
                          Pvalue =   as.numeric ( format ( pchisq (Chi2_LRT_growth, df=  1 , lower.tail =  F), digits=  2 ))) 
    
   tab_stat_day_4  =  tab_stat 
    
    #Day 5  
   tmp  =   subset (Length_dayseclosion, Day  ==   "5" )  %>%  
        mutate ( Day =   as.factor (Day)) 
    
   mod.gen  =   fitme ( log (Total_Length_mm)  ~  Diet  +  ( 1   |  Repeat),  data =  tmp) 
    shapiro.test ( residuals (mod.gen))    
  ## 
##  Shapiro-Wilk normality test
## 
## data:  residuals(mod.gen)
## W = 0.98337, p-value = 0.8115  
       bptest ( log (Total_Length_mm)  ~  Diet  +  ( 1   /  Repeat),  data =  tmp)    
  ## 
##  studentized Breusch-Pagan test
## 
## data:  log(Total_Length_mm) ~ Diet + (1/Repeat)
## BP = 0.17809, df = 1, p-value = 0.673  
      mod.gen1  =   fitme ( log (Total_Length_mm)  ~   1   +  ( 1   |  Repeat),  data =  tmp) 
   test  =   anova (mod.gen, mod.gen1) 
   Chi2_LRT_growth  =   2   *  (mod.gen $ APHLs[[ "p_v" ]]  -  mod.gen1 $ APHLs[[ "p_v" ]]) 
    
   tab_stat  =   data.frame ( Variable =   as.character ( "5" ), 
                          Rep =   nlevels (tmp $ Repeat), 
                          chi2_LR =   round ( as.numeric (test $ basicLRT $ chi2_LR),  digits =   2 ), 
                          intercept =   format (mod.gen $ fixef[ 1 ], digits=  3 ), 
                          estimate =   format (mod.gen $ fixef[ 2 ], digits=  3 ), 
                          df =   as.numeric (test $ basicLRT $ df), 
                          Pvalue =   as.numeric ( format ( pchisq (Chi2_LRT_growth, df=  1 , lower.tail =  F), digits=  2 ))) 
    
   tab_stat_day_5  =  tab_stat 
    
   tab_stat  =   rbind (tab_stat_day_1, tab_stat_day_2, tab_stat_day_3, tab_stat_day_4, tab_stat_day_5) 
    
   tab_stat $ padj  =   p.adjust (tab_stat $ Pvalue,  method =   "BH" ) 
    
   tab_stat $ sig  =   ifelse (tab_stat $ padj  <   0.05   &  tab_stat $ padj  >   0.01 ,  "*" , 
                          ifelse (tab_stat $ padj  <   0.01   &  tab_stat $ padj  >   0.001 ,  "**" , 
                                 ifelse (tab_stat $ padj  <   0.001 ,  "***" ,  "" ))) 
   tab_stat %>%  
      kable ( col.names =   c ( "Comparisons diet within days" ,  "Replicates" ,  "Chi2" , "Intercept" , "Estimate" , "df"  , "p-value" , "p-value adjusted" , "Signif." ), row.names =   FALSE )  %>%  
      add_header_above ( c ( "log(Total_Length_mm) ~  Diet + (1 | Repeat)"   =   9 )) %>%  
      kable_styling ( bootstrap_options =   c ( "striped" ,  "hover" ,  "condensed" ),  full_width =  F)    
 
 
 
 
 
log(Total_Length_mm) ~ Diet + (1 | Repeat)
 
 
 
 
 
Comparisons diet within days
 
 
Replicates
 
 
Chi2
 
 
Intercept
 
 
Estimate
 
 
df
 
 
p-value
 
 
p-value adjusted
 
 
Signif.
 
 
 
 
 
 
1
 
 
3
 
 
12.43
 
 
1.58
 
 
0.095
 
 
1
 
 
4.2e-04
 
 
4.20e-04
 
 
***
 
 
 
 
2
 
 
3
 
 
23.56
 
 
1.59
 
 
0.167
 
 
1
 
 
1.2e-06
 
 
2.00e-06
 
 
***
 
 
 
 
3
 
 
3
 
 
13.67
 
 
1.64
 
 
0.122
 
 
1
 
 
2.2e-04
 
 
2.75e-04
 
 
***
 
 
 
 
4
 
 
3
 
 
45.37
 
 
1.52
 
 
0.271
 
 
1
 
 
0.0e+00
 
 
0.00e+00
 
 
***
 
 
 
 
5
 
 
3
 
 
30.29
 
 
1.57
 
 
0.244
 
 
1
 
 
0.0e+00
 
 
1.00e-07
 
 
***
 
 
 
 
      tab_stat  =  
       tab_stat  %>%  
       dplyr ::  rename ( Day =  Variable)  %>%  
        mutate ( Day =   as.factor (Day)) 
   tab_stat $ Diet  =   "HY"  
    
   letter_position  =   aggregate ( data =  Length_dayseclosion, Total_Length_mm  ~  Day, max) 
    
   tab_stat1  =   left_join (tab_stat, letter_position) 
    
    #Stats vs eclosion for HS  
    
   Length_dayseclosionHS  =   subset (Length_dayseclosion, Diet  ==   "0"   |  Diet  ==   "HS" ) 
    #Day 1  
   tmp  =   subset (Length_dayseclosionHS, Day  ==   "0"   |  Day  ==   "1" )  %>%  
        mutate ( Day =   as.factor (Day)) 
    
   mod.gen  =   fitme ( log (Total_Length_mm)  ~  Day  +  ( 1   |  Repeat),  data =  tmp) 
    shapiro.test ( residuals (mod.gen))    
  ## 
##  Shapiro-Wilk normality test
## 
## data:  residuals(mod.gen)
## W = 0.99072, p-value = 0.9926  
       bptest ( log (Total_Length_mm)  ~  Day  +  ( 1   /  Repeat),  data =  tmp)    
  ## 
##  studentized Breusch-Pagan test
## 
## data:  log(Total_Length_mm) ~ Day + (1/Repeat)
## BP = 1.1212, df = 1, p-value = 0.2897  
      mod.gen1  =   fitme ( log (Total_Length_mm)  ~   1   +  ( 1   |  Repeat),  data =  tmp) 
   test  =   anova (mod.gen, mod.gen1) 
   Chi2_LRT_growth  =   2   *  (mod.gen $ APHLs[[ "p_v" ]]  -  mod.gen1 $ APHLs[[ "p_v" ]]) 
    
   tab_stat  =   data.frame ( Variable =   as.character ( paste ( "1" )), 
                          Rep =   nlevels (tmp $ Repeat), 
                          chi2_LR =   round ( as.numeric (test $ basicLRT $ chi2_LR),  digits =   2 ), 
                          intercept =   format (mod.gen $ fixef[ 1 ], digits=  3 ), 
                          estimate =   format (mod.gen $ fixef[ 2 ], digits=  3 ), 
                          df =   as.numeric (test $ basicLRT $ df), 
                          Pvalue =   as.numeric ( format ( pchisq (Chi2_LRT_growth, df=  1 , lower.tail =  F), digits=  2 ))) 
    
   tab_stat_day_0vs1  =  tab_stat 
    
    #Day 2  
   tmp  =   subset (Length_dayseclosionHS, Day  ==   "0"   |  Day  ==   "2" )  %>%  
        mutate ( Day =   as.factor (Day)) 
    
   mod.gen  =   fitme ( log (Total_Length_mm)  ~  Day  +  ( 1   |  Repeat),  data =  tmp) 
    shapiro.test ( residuals (mod.gen))    
  ## 
##  Shapiro-Wilk normality test
## 
## data:  residuals(mod.gen)
## W = 0.96616, p-value = 0.4006  
       bptest ( log (Total_Length_mm)  ~  Day  +  ( 1   /  Repeat),  data =  tmp)    
  ## 
##  studentized Breusch-Pagan test
## 
## data:  log(Total_Length_mm) ~ Day + (1/Repeat)
## BP = 0.15716, df = 1, p-value = 0.6918  
      mod.gen1  =   fitme ( log (Total_Length_mm)  ~   1   +  ( 1   |  Repeat),  data =  tmp) 
   test  =   anova (mod.gen, mod.gen1) 
   Chi2_LRT_growth  =   2   *  (mod.gen $ APHLs[[ "p_v" ]]  -  mod.gen1 $ APHLs[[ "p_v" ]]) 
    
   tab_stat  =   data.frame ( Variable =   as.character ( paste ( "2" )), 
                          Rep =   nlevels (tmp $ Repeat), 
                          chi2_LR =   round ( as.numeric (test $ basicLRT $ chi2_LR),  digits =   2 ), 
                          intercept =   format (mod.gen $ fixef[ 1 ], digits=  3 ), 
                          estimate =   format (mod.gen $ fixef[ 2 ], digits=  3 ), 
                          df =   as.numeric (test $ basicLRT $ df), 
                          Pvalue =   as.numeric ( format ( pchisq (Chi2_LRT_growth, df=  1 , lower.tail =  F), digits=  2 ))) 
    
   tab_stat_day_0vs2  =  tab_stat 
    
    #Day 3  
   tmp  =   subset (Length_dayseclosionHS, Day  ==   "0"   |  Day  ==   "3" )  %>%  
        mutate ( Day =   as.factor (Day)) 
    
   mod.gen  =   fitme ( log (Total_Length_mm)  ~  Day  +  ( 1   |  Repeat),  data =  tmp) 
    shapiro.test ( residuals (mod.gen))    
  ## 
##  Shapiro-Wilk normality test
## 
## data:  residuals(mod.gen)
## W = 0.98717, p-value = 0.9438  
       bptest ( log (Total_Length_mm)  ~  Day  +  ( 1   /  Repeat),  data =  tmp)    
  ## 
##  studentized Breusch-Pagan test
## 
## data:  log(Total_Length_mm) ~ Day + (1/Repeat)
## BP = 0.11255, df = 1, p-value = 0.7373  
      mod.gen1  =   fitme ( log (Total_Length_mm)  ~   1   +  ( 1   |  Repeat),  data =  tmp) 
   test  =   anova (mod.gen, mod.gen1) 
   Chi2_LRT_growth  =   2   *  (mod.gen $ APHLs[[ "p_v" ]]  -  mod.gen1 $ APHLs[[ "p_v" ]]) 
    
   tab_stat  =   data.frame ( Variable =   as.character ( paste ( "3" )), 
                          Rep =   nlevels (tmp $ Repeat), 
                          chi2_LR =   round ( as.numeric (test $ basicLRT $ chi2_LR),  digits =   2 ), 
                          intercept =   format (mod.gen $ fixef[ 1 ], digits=  3 ), 
                          estimate =   format (mod.gen $ fixef[ 2 ], digits=  3 ), 
                          df =   as.numeric (test $ basicLRT $ df), 
                          Pvalue =   as.numeric ( format ( pchisq (Chi2_LRT_growth, df=  1 , lower.tail =  F), digits=  2 ))) 
    
   tab_stat_day_0vs3  =  tab_stat 
    
    #Day 4  
   tmp  =   subset (Length_dayseclosionHS, Day  ==   "0"   |  Day  ==   "4" )  %>%  
        mutate ( Day =   as.factor (Day)) 
    
   mod.gen  =   fitme ( log (Total_Length_mm)  ~  Day  +  ( 1   |  Repeat),  data =  tmp) 
    shapiro.test ( residuals (mod.gen))    
  ## 
##  Shapiro-Wilk normality test
## 
## data:  residuals(mod.gen)
## W = 0.98443, p-value = 0.8899  
       bptest ( log (Total_Length_mm)  ~  Day  +  ( 1   /  Repeat),  data =  tmp)    
  ## 
##  studentized Breusch-Pagan test
## 
## data:  log(Total_Length_mm) ~ Day + (1/Repeat)
## BP = 2.049, df = 1, p-value = 0.1523  
      mod.gen1  =   fitme ( log (Total_Length_mm)  ~   1   +  ( 1   |  Repeat),  data =  tmp) 
   test  =   anova (mod.gen, mod.gen1) 
   Chi2_LRT_growth  =   2   *  (mod.gen $ APHLs[[ "p_v" ]]  -  mod.gen1 $ APHLs[[ "p_v" ]]) 
    
   tab_stat  =   data.frame ( Variable =   as.character ( paste ( "4" )), 
                          Rep =   nlevels (tmp $ Repeat), 
                          chi2_LR =   round ( as.numeric (test $ basicLRT $ chi2_LR),  digits =   2 ), 
                          intercept =   format (mod.gen $ fixef[ 1 ], digits=  3 ), 
                          estimate =   format (mod.gen $ fixef[ 2 ], digits=  3 ), 
                          df =   as.numeric (test $ basicLRT $ df), 
                          Pvalue =   as.numeric ( format ( pchisq (Chi2_LRT_growth, df=  1 , lower.tail =  F), digits=  2 ))) 
    
   tab_stat_day_0vs4  =  tab_stat 
    
    #Day 5  
   tmp  =   subset (Length_dayseclosionHS, Day  ==   "0"   |  Day  ==   "5" )  %>%  
        mutate ( Day =   as.factor (Day)) 
    
   mod.gen  =   fitme ( log (Total_Length_mm)  ~  Day  +  ( 1   |  Repeat),  data =  tmp) 
    shapiro.test ( residuals (mod.gen))    
  ## 
##  Shapiro-Wilk normality test
## 
## data:  residuals(mod.gen)
## W = 0.97527, p-value = 0.5856  
       bptest ( log (Total_Length_mm)  ~  Day  +  ( 1   /  Repeat),  data =  tmp)    
  ## 
##  studentized Breusch-Pagan test
## 
## data:  log(Total_Length_mm) ~ Day + (1/Repeat)
## BP = 0.010695, df = 1, p-value = 0.9176  
      mod.gen1  =   fitme ( log (Total_Length_mm)  ~   1   +  ( 1   |  Repeat),  data =  tmp) 
   test  =   anova (mod.gen, mod.gen1) 
   Chi2_LRT_growth  =   2   *  (mod.gen $ APHLs[[ "p_v" ]]  -  mod.gen1 $ APHLs[[ "p_v" ]]) 
    
   tab_stat  =   data.frame ( Variable =   as.character ( paste ( "5" )), 
                          Rep =   nlevels (tmp $ Repeat), 
                          chi2_LR =   round ( as.numeric (test $ basicLRT $ chi2_LR),  digits =   2 ), 
                          intercept =   format (mod.gen $ fixef[ 1 ], digits=  3 ), 
                          estimate =   format (mod.gen $ fixef[ 2 ], digits=  3 ), 
                          df =   as.numeric (test $ basicLRT $ df), 
                          Pvalue =   as.numeric ( format ( pchisq (Chi2_LRT_growth, df=  1 , lower.tail =  F), digits=  2 ))) 
    
   tab_stat_day_0vs5  =  tab_stat 
    
   tab_statHSeclosion  =   rbind (tab_stat_day_0vs1, tab_stat_day_0vs2, tab_stat_day_0vs3, tab_stat_day_0vs4, tab_stat_day_0vs5) 
    
   tab_statHSeclosion $ padj  =   p.adjust (tab_statHSeclosion $ Pvalue,  method =   "BH" ) 
    
   tab_statHSeclosion $ sig  =   ifelse (tab_statHSeclosion $ padj  >   0.05 ,  "ns" , 
      ifelse (tab_statHSeclosion $ padj  <   0.05   &  tab_statHSeclosion $ padj  >   0.01 ,  "*" , 
                          ifelse (tab_statHSeclosion $ padj  <   0.01   &  tab_statHSeclosion $ padj  >   0.001 ,  "**" , 
                                 ifelse (tab_statHSeclosion $ padj  <   0.001 ,  "***" ,  "" )))) 
   tab_statHSeclosion %>%  
      kable ( col.names =   c ( "Comparison to eclosion on HS" ,  "Replicates" ,  "Chi2" , "Intercept" , "Estimate" , "df"  , "p-value" , "p-value adjusted" , "Signif." ), row.names =   FALSE )  %>%  
      add_header_above ( c ( "log(Total_Length_mm) ~  Day + (1 | Repeat)"   =   9 )) %>%  
      kable_styling ( bootstrap_options =   c ( "striped" ,  "hover" ,  "condensed" ),  full_width =  F)    
 
 
 
 
 
log(Total_Length_mm) ~ Day + (1 | Repeat)
 
 
 
 
 
Comparison to eclosion on HS
 
 
Replicates
 
 
Chi2
 
 
Intercept
 
 
Estimate
 
 
df
 
 
p-value
 
 
p-value adjusted
 
 
Signif.
 
 
 
 
 
 
1
 
 
3
 
 
1.28
 
 
1.54
 
 
0.0399
 
 
1
 
 
0.260
 
 
0.4333333
 
 
ns
 
 
 
 
2
 
 
3
 
 
1.34
 
 
1.55
 
 
0.0447
 
 
1
 
 
0.250
 
 
0.4333333
 
 
ns
 
 
 
 
3
 
 
3
 
 
4.80
 
 
1.55
 
 
0.0868
 
 
1
 
 
0.028
 
 
0.1400000
 
 
ns
 
 
 
 
4
 
 
3
 
 
0.60
 
 
1.54
 
 
-0.0244
 
 
1
 
 
0.440
 
 
0.4400000
 
 
ns
 
 
 
 
5
 
 
3
 
 
0.74
 
 
1.54
 
 
0.0319
 
 
1
 
 
0.390
 
 
0.4400000
 
 
ns
 
 
 
 
      tab_statHSeclosion  =  
       tab_statHSeclosion  %>%  
       dplyr ::  rename ( Day =  Variable)  %>%  
        mutate ( Day =   as.factor (Day)) 
   tab_statHSeclosion $ Diet  =   "HS"  
    
   letter_position  =   aggregate ( data =  Length_dayseclosion, Total_Length_mm  ~  Day, max) 
    
   tab_statHSeclosion  =   left_join (tab_statHSeclosion, letter_position) 
    
    #Stats vs eclosion for HY  
    
   Length_dayseclosionHS  =   subset (Length_dayseclosion, Diet  ==   "0"   |  Diet  ==   "HY" ) 
    #Day 1  
   tmp  =   subset (Length_dayseclosionHS, Day  ==   "0"   |  Day  ==   "1" )  %>%  
        mutate ( Day =   as.factor (Day)) 
    
   mod.gen  =   fitme ( log (Total_Length_mm)  ~  Day  +  ( 1   |  Repeat),  data =  tmp) 
    shapiro.test ( residuals (mod.gen))    
  ## 
##  Shapiro-Wilk normality test
## 
## data:  residuals(mod.gen)
## W = 0.96951, p-value = 0.4124  
       bptest ( log (Total_Length_mm)  ~  Day  +  ( 1   /  Repeat),  data =  tmp)    
  ## 
##  studentized Breusch-Pagan test
## 
## data:  log(Total_Length_mm) ~ Day + (1/Repeat)
## BP = 1.8328, df = 1, p-value = 0.1758  
      mod.gen1  =   fitme ( log (Total_Length_mm)  ~   1   +  ( 1   |  Repeat),  data =  tmp) 
   test  =   anova (mod.gen, mod.gen1) 
   Chi2_LRT_growth  =   2   *  (mod.gen $ APHLs[[ "p_v" ]]  -  mod.gen1 $ APHLs[[ "p_v" ]]) 
    
   tab_stat  =   data.frame ( Variable =   as.character ( paste ( "1" )), 
                          Rep =   nlevels (tmp $ Repeat), 
                          chi2_LR =   round ( as.numeric (test $ basicLRT $ chi2_LR),  digits =   2 ), 
                          intercept =   format (mod.gen $ fixef[ 1 ], digits=  3 ), 
                          estimate =   format (mod.gen $ fixef[ 2 ], digits=  3 ), 
                          df =   as.numeric (test $ basicLRT $ df), 
                          Pvalue =   as.numeric ( format ( pchisq (Chi2_LRT_growth, df=  1 , lower.tail =  F), digits=  2 ))) 
    
   tab_stat_day_0vs1  =  tab_stat 
    
    #Day 2  
   tmp  =   subset (Length_dayseclosionHS, Day  ==   "0"   |  Day  ==   "2" )  %>%  
        mutate ( Day =   as.factor (Day)) 
    
   mod.gen  =   fitme ( log (Total_Length_mm)  ~  Day  +  ( 1   |  Repeat),  data =  tmp) 
    shapiro.test ( residuals (mod.gen))    
  ## 
##  Shapiro-Wilk normality test
## 
## data:  residuals(mod.gen)
## W = 0.98822, p-value = 0.9615  
       bptest ( log (Total_Length_mm)  ~  Day  +  ( 1   /  Repeat),  data =  tmp)    
  ## 
##  studentized Breusch-Pagan test
## 
## data:  log(Total_Length_mm) ~ Day + (1/Repeat)
## BP = 1.6976, df = 1, p-value = 0.1926  
      mod.gen1  =   fitme ( log (Total_Length_mm)  ~   1   +  ( 1   |  Repeat),  data =  tmp) 
   test  =   anova (mod.gen, mod.gen1) 
   Chi2_LRT_growth  =   2   *  (mod.gen $ APHLs[[ "p_v" ]]  -  mod.gen1 $ APHLs[[ "p_v" ]]) 
    
   tab_stat  =   data.frame ( Variable =   as.character ( paste ( "2" )), 
                          Rep =   nlevels (tmp $ Repeat), 
                          chi2_LR =   round ( as.numeric (test $ basicLRT $ chi2_LR),  digits =   2 ), 
                          intercept =   format (mod.gen $ fixef[ 1 ], digits=  3 ), 
                          estimate =   format (mod.gen $ fixef[ 2 ], digits=  3 ), 
                          df =   as.numeric (test $ basicLRT $ df), 
                          Pvalue =   as.numeric ( format ( pchisq (Chi2_LRT_growth, df=  1 , lower.tail =  F), digits=  2 ))) 
    
   tab_stat_day_0vs2  =  tab_stat 
    
    #Day 3  
   tmp  =   subset (Length_dayseclosionHS, Day  ==   "0"   |  Day  ==   "3" )  %>%  
        mutate ( Day =   as.factor (Day)) 
    
   mod.gen  =   fitme ( log (Total_Length_mm)  ~  Day  +  ( 1   |  Repeat),  data =  tmp) 
    shapiro.test ( residuals (mod.gen))    
  ## 
##  Shapiro-Wilk normality test
## 
## data:  residuals(mod.gen)
## W = 0.98545, p-value = 0.9144  
       bptest ( log (Total_Length_mm)  ~  Day  +  ( 1   /  Repeat),  data =  tmp)    
  ## 
##  studentized Breusch-Pagan test
## 
## data:  log(Total_Length_mm) ~ Day + (1/Repeat)
## BP = 3.1177, df = 1, p-value = 0.07745  
      mod.gen1  =   fitme ( log (Total_Length_mm)  ~   1   +  ( 1   |  Repeat),  data =  tmp) 
   test  =   anova (mod.gen, mod.gen1) 
   Chi2_LRT_growth  =   2   *  (mod.gen $ APHLs[[ "p_v" ]]  -  mod.gen1 $ APHLs[[ "p_v" ]]) 
    
   tab_stat  =   data.frame ( Variable =   as.character ( paste ( "3" )), 
                          Rep =   nlevels (tmp $ Repeat), 
                          chi2_LR =   round ( as.numeric (test $ basicLRT $ chi2_LR),  digits =   2 ), 
                          intercept =   format (mod.gen $ fixef[ 1 ], digits=  3 ), 
                          estimate =   format (mod.gen $ fixef[ 2 ], digits=  3 ), 
                          df =   as.numeric (test $ basicLRT $ df), 
                          Pvalue =   as.numeric ( format ( pchisq (Chi2_LRT_growth, df=  1 , lower.tail =  F), digits=  2 ))) 
    
   tab_stat_day_0vs3  =  tab_stat 
    
    #Day 4  
   tmp  =   subset (Length_dayseclosionHS, Day  ==   "0"   |  Day  ==   "4" )  %>%  
        mutate ( Day =   as.factor (Day)) 
    
   mod.gen  =   fitme ( log (Total_Length_mm)  ~  Day  +  ( 1   |  Repeat),  data =  tmp) 
    shapiro.test ( residuals (mod.gen))    
  ## 
##  Shapiro-Wilk normality test
## 
## data:  residuals(mod.gen)
## W = 0.97795, p-value = 0.6915  
       bptest ( log (Total_Length_mm)  ~  Day  +  ( 1   /  Repeat),  data =  tmp)    
  ## 
##  studentized Breusch-Pagan test
## 
## data:  log(Total_Length_mm) ~ Day + (1/Repeat)
## BP = 0.026163, df = 1, p-value = 0.8715  
      mod.gen1  =   fitme ( log (Total_Length_mm)  ~   1   +  ( 1   |  Repeat),  data =  tmp) 
   test  =   anova (mod.gen, mod.gen1) 
   Chi2_LRT_growth  =   2   *  (mod.gen $ APHLs[[ "p_v" ]]  -  mod.gen1 $ APHLs[[ "p_v" ]]) 
    
   tab_stat  =   data.frame ( Variable =   as.character ( paste ( "4" )), 
                          Rep =   nlevels (tmp $ Repeat), 
                          chi2_LR =   round ( as.numeric (test $ basicLRT $ chi2_LR),  digits =   2 ), 
                          intercept =   format (mod.gen $ fixef[ 1 ], digits=  3 ), 
                          estimate =   format (mod.gen $ fixef[ 2 ], digits=  3 ), 
                          df =   as.numeric (test $ basicLRT $ df), 
                          Pvalue =   as.numeric ( format ( pchisq (Chi2_LRT_growth, df=  1 , lower.tail =  F), digits=  2 ))) 
   tab_stat_day_0vs4  =  tab_stat 
    
    
    #Day 5  
   tmp  =   subset (Length_dayseclosionHS, Day  ==   "0"   |  Day  ==   "5" )  %>%  
        mutate ( Day =   as.factor (Day)) 
    
   mod.gen  =   fitme ( log (Total_Length_mm)  ~  Day  +  ( 1   |  Repeat),  data =  tmp) 
    shapiro.test ( residuals (mod.gen))    
  ## 
##  Shapiro-Wilk normality test
## 
## data:  residuals(mod.gen)
## W = 0.98099, p-value = 0.8035  
       bptest ( log (Total_Length_mm)  ~  Day  +  ( 1   /  Repeat),  data =  tmp)    
  ## 
##  studentized Breusch-Pagan test
## 
## data:  log(Total_Length_mm) ~ Day + (1/Repeat)
## BP = 0.21338, df = 1, p-value = 0.6441  
      mod.gen1  =   fitme ( log (Total_Length_mm)  ~   1   +  ( 1   |  Repeat),  data =  tmp) 
   test  =   anova (mod.gen, mod.gen1) 
   Chi2_LRT_growth  =   2   *  (mod.gen $ APHLs[[ "p_v" ]]  -  mod.gen1 $ APHLs[[ "p_v" ]]) 
    
   tab_stat  =   data.frame ( Variable =   as.character ( paste ( "5" )), 
                          Rep =   nlevels (tmp $ Repeat), 
                          chi2_LR =   round ( as.numeric (test $ basicLRT $ chi2_LR),  digits =   2 ), 
                          intercept =   format (mod.gen $ fixef[ 1 ], digits=  3 ), 
                          estimate =   format (mod.gen $ fixef[ 2 ], digits=  3 ), 
                          df =   as.numeric (test $ basicLRT $ df), 
                          Pvalue =   as.numeric ( format ( pchisq (Chi2_LRT_growth, df=  1 , lower.tail =  F), digits=  2 ))) 
   tab_stat_day_0vs5  =  tab_stat 
    
   tab_statHYeclosion  =   rbind (tab_stat_day_0vs1, tab_stat_day_0vs2, tab_stat_day_0vs3, tab_stat_day_0vs4, tab_stat_day_0vs5) 
    
   tab_statHYeclosion $ padj  =   as.numeric ( p.adjust (tab_statHYeclosion $ Pvalue,  method =   "BH" )) 
    
   tab_statHYeclosion $ sig  =   ifelse (tab_statHYeclosion $ padj  >   0.05 ,  "ns" , 
      ifelse (tab_statHYeclosion $ padj  <   0.05   &  tab_statHYeclosion $ padj  >   0.01 ,  "*" , 
                          ifelse (tab_statHYeclosion $ padj  <   0.01   &  tab_statHYeclosion $ padj  >   0.001 ,  "**" , 
                                 ifelse (tab_statHYeclosion $ padj  <   0.001 ,  "***" ,  "" )))) 
    
   tab_statHYeclosion %>%  
      kable ( col.names =   c ( "Comparison to eclosion on HY" ,  "Replicates" ,  "Chi2" , "Intercept" , "Estimate" , "df"  , "p-value" , "p-value adjusted" , "Signif." ), row.names =   FALSE )  %>%  
      add_header_above ( c ( "log(Total_Length_mm) ~  Day + (1 | Repeat)"   =   9 )) %>%  
      kable_styling ( bootstrap_options =   c ( "striped" ,  "hover" ,  "condensed" ),  full_width =  F)    
 
 
 
 
 
log(Total_Length_mm) ~ Day + (1 | Repeat)
 
 
 
 
 
Comparison to eclosion on HY
 
 
Replicates
 
 
Chi2
 
 
Intercept
 
 
Estimate
 
 
df
 
 
p-value
 
 
p-value adjusted
 
 
Signif.
 
 
 
 
 
 
1
 
 
3
 
 
15.38
 
 
1.53
 
 
0.141
 
 
1
 
 
8.8e-05
 
 
8.8e-05
 
 
***
 
 
 
 
2
 
 
3
 
 
28.64
 
 
1.55
 
 
0.209
 
 
1
 
 
1.0e-07
 
 
2.0e-07
 
 
***
 
 
 
 
3
 
 
3
 
 
29.67
 
 
1.55
 
 
0.209
 
 
1
 
 
1.0e-07
 
 
2.0e-07
 
 
***
 
 
 
 
4
 
 
3
 
 
27.96
 
 
1.55
 
 
0.24
 
 
1
 
 
1.0e-07
 
 
2.0e-07
 
 
***
 
 
 
 
5
 
 
3
 
 
28.06
 
 
1.55
 
 
0.263
 
 
1
 
 
1.0e-07
 
 
2.0e-07
 
 
***
 
 
 
 
      tab_statHYeclosion  =  
       tab_statHYeclosion  %>%  
       dplyr ::  rename ( Day =  Variable)  %>%  
        mutate ( Day =   as.factor (Day)) 
   tab_statHYeclosion $ Diet  =   "HY"  
    
   letter_position  =   aggregate ( data =  Length_dayseclosion, Total_Length_mm  ~  Day, max) 
    
   tab_statHYeclosion  =   left_join (tab_statHYeclosion, letter_position) 
    
    ### Plot  
   z  =   max (Length_dayseclosion $ Total_Length_mm,  na.rm =   TRUE ) 
    
   Plot_Fig4S1A =  
      ggplot (Length_dayseclosion,  aes ( x =  Day,  y =  Total_Length_mm)) +   
      geom_violin ( aes ( fill =  Diet),  draw_quantiles =   c ( 0.25 ,  0.5 ,  0.75 ),  colour =   "black" ,  size =   0.2 , adjust =   0.8 )  +  
      geom_dotplot (  colour =   "black" ,  fill =   "white" ,  binaxis =   "y" ,  stackdir =   "center" ,  binwidth =  z /  60 )  +   
      facet_grid (. ~ Diet, scales=  "free_x" , space=  "free" ) +  
      geom_text ( data =  Sample_size,  mapping =   aes ( x =  Day,  y =   2.3 ,  label =   paste ( "(" ,Sample_size, ")" , sep=  "" )), size=  3 ) +  
      geom_text ( data =  tab_stat1,  mapping =   aes ( x = Day,  y =   7.5 ,  label =  sig), size=  3 ) +  
      geom_text ( data =  tab_statHSeclosion,  mapping =   aes ( x = Day,  y =   3.5 ,  label =  sig), size=  3 ) +  
      geom_text ( data =  tab_statHYeclosion,  mapping =   aes ( x = Day,  y =   3.5 ,  label =  sig), size=  3 ) +  
      
      
      scale_fill_manual ( limits=  c ( "0" , "HS" , "HY" ), 
                        values=   c ( "#cfe7cf" , "#FFB4B4" ,  "#C3E6FC" )) +  
      scale_x_discrete ( "Days post eclosion" ) +  
      scale_y_continuous ( "Midgut length (mm)" , 
                         limits=  c ( 2 , 8.2 ), 
                         breaks=  seq ( 2 , 8 , by=  1 )) +  
      stat_summary ( fun =  mean,  geom =   "point" ,  size =   3 ,  shape =   18 ,  colour =   "black" ,  aes ( group =  Repeat))  +  
      stat_summary ( fun =  mean,  geom =   "point" ,  size =   2 ,  shape =   18 ,  aes ( group =  Repeat,  colour =  Repeat))  +  
      scale_color_manual ( values =  palette_mean)  +  
      theme ( panel.grid.major.y =   element_line ( colour =   grey ( 0.45 ),  linetype =   "dashed" ,  size =   0.2 ), 
            panel.background =   element_blank (), 
            axis.title.x =   element_text ( size= Smallfont, colour=  "black" ), 
            axis.title.y =   element_text ( size= Smallfont, colour=  "black" ),  
            axis.line.x =   element_line ( colour=  "black" , size=  0.75 ), 
            axis.line.y =   element_line ( colour=  "black" , size=  0.75 ), 
            axis.ticks.x =   element_line ( size =   0.75 ), 
            axis.ticks.y =   element_line ( size =   0.75 ), 
            axis.text.x =   element_text ( size= Smallfont, colour=  "black" ), 
            axis.text.y =   element_text ( size= Smallfont, colour=  "black" ), 
            plot.margin =   unit (Margin,  "cm" ), 
            legend.direction =   "vertical" ,  
            legend.box =   "horizontal" , 
            legend.position =   "none" , 
            legend.key.height =   unit ( 0.4 ,  "cm" ), 
            legend.key.width=   unit ( 0.6 ,  "cm" ), 
            legend.title =   element_text ( face=  "italic" , size= Smallfont),  
            legend.key =   element_rect ( colour =   'white' ,  fill =   "white" ,  linetype=  'dashed' ), 
            legend.text =   element_text ( size= SuperSmallfont), 
            legend.background =   element_rect ( fill=  NA ), 
            strip.text.x =   element_text ( size =  Smallfont,  colour =   "black" ,  margin =   margin ( t =   2 ,  r =   0 ,  b =   2 ,  l =   0 )), 
            strip.text.y =   element_text ( size =  Smallfont,  colour =   "black" ,  margin =   margin ( t =   2 ,  r =   0 ,  b =   2 ,  l =   0 )), 
            strip.background =   element_rect ( fill=  NA ,  colour=  "black" ), 
            strip.placement=  "outside" ) 
    
   Plot_Fig4S1A    
   
 
 
  4.2.2  Figure 4S1B 
 
 Scheme for Figure 4 B, C, D and Figure 4 supplement 1C, D, E. At eclosion, flies were allocated to either HS or HY diet. 7-, 14- and 21-days post eclosion flies were either kept on the same diet or shifted on the opposite diet for 7 days (HS to HY or HY to HS). Flies were dissected every 7 days, up until day 28. 
 
      img4S1B  =   readImage ( "D:/Dropbox/z_ Ale Shared work/z_Nutrition Paper Markdown/Ale/Revision/4 - S1B.jpg" )  
   gob_imageFig4S1B  =   rasterGrob (img4S1B) 
    grid.draw (gob_imageFig4S1B)    
   
 
 
  4.2.3  Figure 4S1C 
 
 HS diet does not postpone post-eclosion development, but rather induces continual midgut shrinkage over 28 days of feeding. Letters above violin plots represent grouping by statistical differences (Post hoc Tukey on GLMM). 
 
      Length_longshift  =   
     d[[ "4B, 4S1C" ]] %>%  
      select ( - Total.PH3) %>%  
      mutate ( Total_Length_mm =  Total.L /  1000 ) %>%  
      mutate_if (is.character,as.factor) %>%  
      mutate_if (is.integer,as.factor) %>%  
     dplyr ::  rename ( Day_of_treatment= Day) 
    
   Sample_size =  
     Length_longshift %>%  
      group_by (Day_of_treatment,Diet) %>%  
      summarise ( Sample_size=  n ()) 
    
    ###Stats  
    
    #HS  
   tmp =   subset (Length_longshift, Diet ==  "HS" ) 
   mod.gen  =   fitme ( log (Total_Length_mm)  ~   Day_of_treatment  +  ( 1   |  Repeat),  data =  tmp) 
    shapiro.test ( residuals (mod.gen))    
  ## 
##  Shapiro-Wilk normality test
## 
## data:  residuals(mod.gen)
## W = 0.98712, p-value = 0.5309  
       bptest ( log (Total_Length_mm)  ~  Day_of_treatment  +  ( 1   /  Repeat),  data =  tmp)    
  ## 
##  studentized Breusch-Pagan test
## 
## data:  log(Total_Length_mm) ~ Day_of_treatment + (1/Repeat)
## BP = 4.1872, df = 3, p-value = 0.2419  
      mod.gen1  =   fitme ( log (Total_Length_mm)  ~   1   +  ( 1   |  Repeat),  data =  tmp) 
   test  =   anova (mod.gen, mod.gen1) 
   Chi2_LRT_growth  =   2  * (mod.gen $ APHLs[[ "p_v" ]] - mod.gen1 $ APHLs[[ "p_v" ]]) 
    
   tab_stat  =   data.frame ( Diet =   as.character ( paste ( "HS" )), 
                          Rep =   nlevels (tmp $ Repeat), 
                          chi2_LR =   round ( as.numeric (test $ basicLRT $ chi2_LR),  digits =   2 ), 
                          intercept =   format (mod.gen $ fixef[ 1 ], digits=  3 ), 
                          estimate =   format (mod.gen $ fixef[ 2 ], digits=  3 ), 
                          df =   as.numeric (test $ basicLRT $ df), 
                          Pvalue =   as.numeric ( format ( pchisq (Chi2_LRT_growth, df=  1 , lower.tail =  F), digits=  2 ))) 
   tab_stat_HS =  tab_stat 
    
   mod.gen  =   lmer ( log (Total_Length_mm)  ~   Day_of_treatment  +  ( 1   |  Repeat),  data =  tmp) 
   multcomp  =   glht (mod.gen,  linfct=  mcp ( Day_of_treatment=  "Tukey" )) 
    
   Comp_HS  =   cld (multcomp) 
   letter_position_HS  =   aggregate ( data= tmp,Total_Length_mm  ~   Day_of_treatment, max) 
   letter_position_HS $ Diet =  "HS"  
    
    #HY  
   tmp =   subset (Length_longshift, Diet ==  "HY" ) 
   mod.gen  =   fitme ( log (Total_Length_mm)  ~   Day_of_treatment  +  ( 1   |  Repeat),  data =  tmp) 
    shapiro.test ( residuals (mod.gen))    
  ## 
##  Shapiro-Wilk normality test
## 
## data:  residuals(mod.gen)
## W = 0.98536, p-value = 0.3654  
       bptest ( log (Total_Length_mm)  ~  Day_of_treatment  +  ( 1   /  Repeat),  data =  tmp)    
  ## 
##  studentized Breusch-Pagan test
## 
## data:  log(Total_Length_mm) ~ Day_of_treatment + (1/Repeat)
## BP = 3.2143, df = 3, p-value = 0.3598  
      mod.gen1  =   fitme ( log (Total_Length_mm)  ~   1   +  ( 1   |  Repeat),  data =  tmp) 
   test  =   anova (mod.gen, mod.gen1) 
   Chi2_LRT_growth  =   2  * (mod.gen $ APHLs[[ "p_v" ]] - mod.gen1 $ APHLs[[ "p_v" ]]) 
    
   tab_stat  =   data.frame ( Diet =   as.character ( paste ( "HY" )), 
                          Rep =   nlevels (tmp $ Repeat), 
                          chi2_LR =   round ( as.numeric (test $ basicLRT $ chi2_LR),  digits =   2 ), 
                          intercept =   format (mod.gen $ fixef[ 1 ], digits=  3 ), 
                          estimate =   format (mod.gen $ fixef[ 2 ], digits=  3 ), 
                          df =   as.numeric (test $ basicLRT $ df), 
                          Pvalue =   as.numeric ( format ( pchisq (Chi2_LRT_growth, df=  1 , lower.tail =  F), digits=  2 ))) 
   tab_stat_HY  = tab_stat 
    
   mod.gen  =   lmer ( log (Total_Length_mm)  ~   Day_of_treatment  +  ( 1   |  Repeat),  data =  tmp) 
   multcomp  =   glht (mod.gen,  linfct=  mcp ( Day_of_treatment=  "Tukey" )) 
    
   Comp_HY  =   cld (multcomp) 
   letter_position_HY  =   aggregate ( data= tmp,Total_Length_mm  ~   Day_of_treatment, max) 
   letter_position_HY $ Diet =  "HY"  
    
   tab_stat  =   rbind (tab_stat_HS,tab_stat_HY) 
    
   tab_stat $ sig  =   ifelse (tab_stat $ Pvalue  <   0.05   &  tab_stat $ Pvalue  >   0.01 ,  "*" , 
                 ifelse (tab_stat $ Pvalue  <   0.01   &  tab_stat $ Pvalue  >   0.001 ,  "**" , 
                  ifelse (tab_stat $ Pvalue  <   0.001 ,  "***" ,  "" ))) 
   tab_stat %>%  
      kable ( col.names =   c ( "Any difference" ,  "Replicates" ,  "Chi2" , "Intercept" , "Estimate" , "df"  , "p-value" , "Signif." ), row.names =   FALSE )  %>%  
      add_header_above ( c ( "log(Total_Length_mm) ~  Day + (1 | Repeat)"   =   8 )) %>%  
      kable_styling ( bootstrap_options =   c ( "striped" ,  "hover" ,  "condensed" ),  full_width =  F)    
 
 
 
 
 
log(Total_Length_mm) ~ Day + (1 | Repeat)
 
 
 
 
 
Any difference
 
 
Replicates
 
 
Chi2
 
 
Intercept
 
 
Estimate
 
 
df
 
 
p-value
 
 
Signif.
 
 
 
 
 
 
HS
 
 
3
 
 
31.86
 
 
1.43
 
 
-0.185
 
 
3
 
 
0.00000
 
 
***
 
 
 
 
HY
 
 
3
 
 
12.49
 
 
1.81
 
 
0.0575
 
 
3
 
 
0.00041
 
 
***
 
 
 
 
      letter_position  =   rbind (letter_position_HS,letter_position_HY) 
    
   tab_letter_HS  =    as.data.frame (Comp_HS $ mcletters $ Letters) 
   tab_letter_HS $ Diet  =   "HS"  
   tab_letter_HS $ Day_of_treatment =  rownames (tab_letter_HS) 
    colnames (tab_letter_HS)[ 1 ]  =   "Letter"  
    
   tab_letter_HY  =    as.data.frame (Comp_HY $ mcletters $ Letters) 
   tab_letter_HY $ Day_of_treatment =  rownames (tab_letter_HY) 
   tab_letter_HY $ Diet  =   "HY"  
    colnames (tab_letter_HY)[ 1 ]  =   "Letter"  
    
    
   tab_letter  =   rbind (tab_letter_HS,tab_letter_HY) 
   tab_letter  =   left_join (tab_letter,letter_position) 
    
    ### Plot  
   z =  max (Length_longshift $ Total_Length_mm,  na.rm =   TRUE ) 
    
   Plot_Fig4S1C =  
      ggplot (Length_longshift,  aes ( x =  Day_of_treatment,  y =  Total_Length_mm)) +   
      geom_violin ( aes ( fill =  Diet),  draw_quantiles =   c ( 0.25 ,  0.5 ,  0.75 ),  colour =   "black" ,  size =   0.2 , adjust =   0.8 )  +  
      geom_dotplot (  colour =   "black" ,  fill =   "white" ,  binaxis =   "y" ,  stackdir =   "center" ,  binwidth =   0.15 )  +   
      facet_grid (. ~ Diet, scales=  "free_x" , space=  "free" ) +  
      geom_text ( data =  Sample_size,  mapping =   aes ( x =  Day_of_treatment,  y =   1.5 ,  label =   paste ( "(" ,Sample_size, ")" , sep=  "" )), size=  3 ) +  
      geom_text ( data =  tab_letter,  mapping =   aes ( x =  Day_of_treatment,  y =  Total_Length_mm +0.4 ,  label =  Letter), size=  3 ) +  
      geom_text ( data =  tab_stat,  mapping =   aes ( x =   1.5 ,  y =   9.5 ,  label =   paste ( "p=" , format (Pvalue, digits=  2 ))), size=  3 ) +  
      scale_fill_manual ( limits=  c ( "0" , "HS" , "HY" ), 
                        values=   c ( "#cfe7cf" , "#FFB4B4" ,  "#C3E6FC" )) +  
      scale_x_discrete ( "Days post eclosion" ) +  
      scale_y_continuous ( "Midgut length (mm)" , 
                         limits=  c ( 1.5 , 9.5 ), 
                         breaks=  seq ( 3 , 9 , by=  1 )) +  
         stat_summary ( fun =  mean,  geom =   "point" ,  size =   3 ,  shape =   18 ,  colour =   "black" ,  aes ( group =  Repeat))  +  
                         stat_summary ( fun =  mean,  geom =   "point" ,  size =   2 ,  shape =   18 ,  aes ( group =  Repeat,  colour =  Repeat))  +  
                         scale_color_manual ( values =  palette_mean)  +  
      theme ( panel.grid.major.y =   element_line ( colour =   grey ( 0.45 ),  linetype =   "dashed" ,  size =   0.2 ), 
            panel.background =   element_blank (), 
            axis.title.x =   element_text ( size= Smallfont, colour=  "black" ), 
            axis.title.y =   element_text ( size= Smallfont, colour=  "black" ),  
            axis.line.x =   element_line ( colour=  "black" , size=  0.75 ), 
            axis.line.y =   element_line ( colour=  "black" , size=  0.75 ), 
            axis.ticks.x =   element_line ( size =   0.75 ), 
            axis.ticks.y =   element_line ( size =   0.75 ), 
            axis.text.x =   element_text ( size= Smallfont, colour=  "black" ), 
            axis.text.y =   element_text ( size= Smallfont, colour=  "black" ), 
            plot.margin =   unit (Margin,  "cm" ), 
            legend.direction =   "vertical" ,  
            legend.box =   "horizontal" , 
            legend.position =   "none" , 
            legend.key.height =   unit ( 0.4 ,  "cm" ), 
            legend.key.width=   unit ( 0.6 ,  "cm" ), 
            legend.title =   element_text ( face=  "italic" , size= Smallfont),  
            legend.key =   element_rect ( colour =   'white' ,  fill =   "white" ,  linetype=  'dashed' ), 
            legend.text =   element_text ( size= SuperSmallfont), 
            legend.background =   element_rect ( fill=  NA ), 
            strip.text.x =   element_text ( size =  Smallfont,  colour =   "black" ,  margin =   margin ( t =   2 ,  r =   0 ,  b =   2 ,  l =   0 )), 
            strip.text.y =   element_text ( size =  Smallfont,  colour =   "black" ,  margin =   margin ( t =   2 ,  r =   0 ,  b =   2 ,  l =   0 )), 
            strip.background =   element_rect ( fill=  NA ,  colour=  "black" ), 
            strip.placement=  "outside" ) 
    
   Plot_Fig4S1C    
   
 
 
  4.2.4  Figure 4S1D 
 
 Midgut size is a plastic, diet-dependent trait. Midguts of flies shifted between HS or HY can reversibly grow throughout 21 days. Statistical comparison is vs pre-shift measurement. 
 
      Length_shift_growth  =   
     d[[ "4C, 4S1D" ]] %>%  
      select ( - Total.PH3) %>%  
      mutate ( Total_Length_mm =  Total.L /  1000 ) %>%  
      mutate_if (is.character,as.factor) %>%  
      mutate_if (is.integer,as.factor) %>%  
     dplyr ::  rename ( Day_of_treatment= Day) %>%  
      as.data.frame () %>%  
      mutate ( Dday=  fct_relevel (Dday, "Shift Day 7" , "Shift Day 14" , "Shift Day 21" )) 
      
   Sample_size =  
     Length_shift_growth %>%  
      group_by (Dday,Diet) %>%  
      summarise ( Sample_size=  n ()) 
    
    ###Stats  
    # Day 7  
   tmp =   subset (Length_shift_growth,Dday ==  "Shift Day 7" ) 
   mod.gen  =   fitme ( log (Total_Length_mm)  ~   Diet  +  ( 1   |  Repeat),  data =  tmp) 
    shapiro.test ( residuals (mod.gen))    
  ## 
##  Shapiro-Wilk normality test
## 
## data:  residuals(mod.gen)
## W = 0.98038, p-value = 0.6352  
       bptest ( log (Total_Length_mm)  ~  Diet  +  ( 1   /  Repeat),  data =  tmp)    
  ## 
##  studentized Breusch-Pagan test
## 
## data:  log(Total_Length_mm) ~ Diet + (1/Repeat)
## BP = 0.59118, df = 1, p-value = 0.442  
      mod.gen1  =   fitme ( log (Total_Length_mm)  ~   1   +  ( 1   |  Repeat),  data =  tmp) 
   test  =   anova (mod.gen, mod.gen1) 
   Chi2_LRT_growth  =   2  * (mod.gen $ APHLs[[ "p_v" ]] - mod.gen1 $ APHLs[[ "p_v" ]]) 
    
   tab_stat  =   data.frame ( Comparison =   as.character ( paste (( "HS Day 7 vs HS to HY Day 14" ))), 
                          Dday =   as.character ( paste ( "Shift Day 7" )), 
                          Rep =   nlevels (tmp $ Repeat), 
                          chi2_LR =   round ( as.numeric (test $ basicLRT $ chi2_LR),  digits =   2 ), 
                          intercept =   format (mod.gen $ fixef[ 1 ], digits=  3 ), 
                          estimate =   format (mod.gen $ fixef[ 2 ], digits=  3 ), 
                          df =   as.numeric (test $ basicLRT $ df), 
                          Pvalue =   as.numeric ( format ( pchisq (Chi2_LRT_growth, df=  1 , lower.tail =  F), digits=  2 ))) 
   tab_stat_7  =  tab_stat 
    
    # Day 14  
   tmp =   subset (Length_shift_growth,Dday ==  "Shift Day 14" ) 
   mod.gen  =   fitme ( log (Total_Length_mm)  ~   Diet  +  ( 1   |  Repeat),  data =  tmp) 
    shapiro.test ( residuals (mod.gen))    
  ## 
##  Shapiro-Wilk normality test
## 
## data:  residuals(mod.gen)
## W = 0.95853, p-value = 0.1396  
       bptest ( log (Total_Length_mm)  ~  Diet  +  ( 1   /  Repeat),  data =  tmp)    
  ## 
##  studentized Breusch-Pagan test
## 
## data:  log(Total_Length_mm) ~ Diet + (1/Repeat)
## BP = 5.1346, df = 1, p-value = 0.02345  
      mod.gen1  =   fitme ( log (Total_Length_mm)  ~   1   +  ( 1   |  Repeat),  data =  tmp) 
   test  =   anova (mod.gen, mod.gen1) 
   Chi2_LRT_growth  =   2  * (mod.gen $ APHLs[[ "p_v" ]] - mod.gen1 $ APHLs[[ "p_v" ]]) 
    
   tab_stat  =   data.frame ( Comparison =   as.character ( paste (( "HS Day 14 vs HS to HY Day 21" ))), 
                          Dday =   as.character ( paste ( "Shift Day 14" )), 
                          Rep =   nlevels (tmp $ Repeat), 
                          chi2_LR =   round ( as.numeric (test $ basicLRT $ chi2_LR),  digits =   2 ), 
                          intercept =   format (mod.gen $ fixef[ 1 ], digits=  3 ), 
                          estimate =   format (mod.gen $ fixef[ 2 ], digits=  3 ), 
                          df =   as.numeric (test $ basicLRT $ df), 
                          Pvalue =   as.numeric ( format ( pchisq (Chi2_LRT_growth, df=  1 , lower.tail =  F), digits=  2 ))) 
   tab_stat_14  =  tab_stat 
    
    # Day 21  
   tmp =   subset (Length_shift_growth,Dday ==  "Shift Day 21" ) 
   mod.gen  =   fitme ( log (Total_Length_mm)  ~   Diet  +  ( 1   |  Repeat),  data =  tmp) 
    shapiro.test ( residuals (mod.gen))    
  ## 
##  Shapiro-Wilk normality test
## 
## data:  residuals(mod.gen)
## W = 0.97628, p-value = 0.346  
       bptest ( log (Total_Length_mm)  ~  Diet  +  ( 1   /  Repeat),  data =  tmp)    
  ## 
##  studentized Breusch-Pagan test
## 
## data:  log(Total_Length_mm) ~ Diet + (1/Repeat)
## BP = 0.19389, df = 1, p-value = 0.6597  
      mod.gen1  =   fitme ( log (Total_Length_mm)  ~   1   +  ( 1   |  Repeat),  data =  tmp) 
   test  =   anova (mod.gen, mod.gen1) 
   Chi2_LRT_growth  =   2  * (mod.gen $ APHLs[[ "p_v" ]] - mod.gen1 $ APHLs[[ "p_v" ]]) 
    
   tab_stat  =   data.frame ( Comparison =   as.character ( paste (( "HS Day 21 vs HS to HY Day 28" ))), 
                          Dday =   as.character ( paste ( "Shift Day 21" )), 
                          Rep =   nlevels (tmp $ Repeat), 
                          chi2_LR =   round ( as.numeric (test $ basicLRT $ chi2_LR),  digits =   2 ), 
                          intercept =   format (mod.gen $ fixef[ 1 ], digits=  3 ), 
                          estimate =   format (mod.gen $ fixef[ 2 ], digits=  3 ), 
                          df =   as.numeric (test $ basicLRT $ df), 
                          Pvalue =   as.numeric ( format ( pchisq (Chi2_LRT_growth, df=  1 , lower.tail =  F), digits=  2 ))) 
   tab_stat_21  =  tab_stat 
    
   tab_stat =  rbind (tab_stat_7,tab_stat_14,tab_stat_21) 
    
   tab_stat $ sig  =   ifelse (tab_stat $ Pvalue  <   0.05   &  tab_stat $ Pvalue  >   0.01 ,  "*" , 
                 ifelse (tab_stat $ Pvalue  <   0.01   &  tab_stat $ Pvalue  >   0.001 ,  "**" , 
                  ifelse (tab_stat $ Pvalue  <   0.001 ,  "***" ,  "" ))) 
    
   tab_stat %>%  
      kable ( col.names =   c ( "Comparison" ,  "Shift day" ,  "Replicates" ,  "Chi2" , "Intercept" , "Estimate" , "df"  , "p-value" , "Signif." ), row.names =   FALSE )  %>%     add_header_above ( c ( "log(Total_Length_mm) ~  Diet + (1 | Repeat)"   =   9 )) %>%  
      kable_styling ( bootstrap_options =   c ( "striped" ,  "hover" ,  "condensed" ),  full_width =  F)    
 
 
 
 
 
log(Total_Length_mm) ~ Diet + (1 | Repeat)
 
 
 
 
 
Comparison
 
 
Shift day
 
 
Replicates
 
 
Chi2
 
 
Intercept
 
 
Estimate
 
 
df
 
 
p-value
 
 
Signif.
 
 
 
 
 
 
HS Day 7 vs HS to HY Day 14
 
 
Shift Day 7
 
 
3
 
 
47.53
 
 
1.43
 
 
0.284
 
 
1
 
 
0
 
 
***
 
 
 
 
HS Day 14 vs HS to HY Day 21
 
 
Shift Day 14
 
 
3
 
 
51.25
 
 
1.24
 
 
0.412
 
 
1
 
 
0
 
 
***
 
 
 
 
HS Day 21 vs HS to HY Day 28
 
 
Shift Day 21
 
 
3
 
 
54.31
 
 
1.19
 
 
0.497
 
 
1
 
 
0
 
 
***
 
 
 
 
      tab_stat =  
     tab_stat %>%  
      mutate_if (is.character,as.factor) %>%  
      as.data.frame () %>%  
      mutate ( Dday=  fct_relevel (Dday, "Shift Day 7" , "Shift Day 14" , "Shift Day 21" )) 
    
    ### Plot  
    
   Treatment.status  =   c ( "Shift  \n  Day 7" , "Shift  \n  Day 14" , "Shift  \n  Day 21" ) 
    names (Treatment.status)  =   c ( "Shift Day 7" ,  "Shift Day 14" , "Shift Day 21" ) 
   z =  max (Length_shift_growth $ Total_Length_mm,  na.rm =   TRUE ) 
    
   Plot_Fig4S1D =  
      ggplot (Length_shift_growth,  aes ( x =  Diet,  y =  Total_Length_mm)) +   
      geom_violin ( aes ( fill =  Diet),  draw_quantiles =   c ( 0.25 ,  0.5 ,  0.75 ),  colour =   "black" ,  size =   0.2 , adjust =   0.8 )  +  
      geom_dotplot (  colour =   "black" ,  fill =   "white" ,  binaxis =   "y" ,  stackdir =   "center" ,  binwidth =  z /  60 )  +   
      facet_grid (.  ~  Dday, labeller=  labeller ( Dday= Treatment.status) ) +  
      geom_text ( data =  Sample_size,  mapping =   aes ( x =  Diet,  y =   1.6 ,  label =   paste ( "(" ,Sample_size, ")" , sep=  "" )), size=  3 ) +  
          geom_signif ( data =  tab_stat,  aes ( xmin =   1 ,  xmax =   2 ,  annotations =   formatC ( paste ( "p=" ,Pvalue),  digits =   2 ),  y_position =   9 ),  textsize =   2.5 ,  vjust =   -  0.2 ,  manual =   TRUE ) +  
    
      scale_fill_manual ( limits=  c ( "HS" , "HStoHY" ), 
                        values= cbbHS_HStoHY) +  
      scale_x_discrete ( "" , 
                       limits=  c ( "HS" , "HStoHY" ), 
                       labels=  c ( "HS" , "HS to HY" )) +  
      scale_y_continuous ( "Midgut length (mm)" , 
                         limits=  c ( 1.5 , 9.5 ), 
                         breaks=  seq ( 2 , 8 , by=  1 )) +  
      stat_summary ( fun =  mean,  geom =   "point" ,  size =   2.5 ,  shape =   18 , aes ( group= Repeat,  colour =  Repeat))  +  
      stat_summary ( fun =  mean,  geom =   "point" ,  size =   3 ,  shape =   18 ,  colour =   "black" ,  aes ( group =  Repeat))  +  
      stat_summary ( fun =  mean,  geom =   "point" ,  size =   2 ,  shape =   18 ,  aes ( group =  Repeat,  colour =  Repeat))  +  
      scale_color_manual ( values =  palette_mean)  +  
      theme ( panel.grid.major.y =   element_line ( colour =   grey ( 0.45 ),  linetype =   "dashed" ,  size =   0.2 ), 
            panel.background =   element_blank (), 
            axis.title.x =   element_text ( size= Smallfont, colour=  "black" ), 
            axis.title.y =   element_text ( size= Smallfont, colour=  "black" ),  
            axis.line.x =   element_line ( colour=  "black" , size=  0.75 ), 
            axis.line.y =   element_line ( colour=  "black" , size=  0.75 ), 
            axis.ticks.x =   element_line ( size =   0.75 ), 
            axis.ticks.y =   element_line ( size =   0.75 ), 
            axis.text.x =   element_text ( size= Smallfont, colour=  "black" , angle=  30 , hjust=  1 ), 
            axis.text.y =   element_text ( size= Smallfont, colour=  "black" ), 
            plot.margin =   unit (Margin,  "cm" ), 
            legend.direction =   "vertical" ,  
            legend.box =   "horizontal" , 
            legend.position =   "none" , 
            legend.key.height =   unit ( 0.4 ,  "cm" ), 
            legend.key.width=   unit ( 0.6 ,  "cm" ), 
            legend.title =   element_text ( face=  "italic" , size= Smallfont),  
            legend.key =   element_rect ( colour =   'white' ,  fill =   "white" ,  linetype=  'dashed' ), 
            legend.text =   element_text ( size= SuperSmallfont), 
            legend.background =   element_rect ( fill=  NA ), 
            strip.text.x =   element_text ( size =  Smallfont,  colour =   "black" ,  margin =   margin ( t =   2 ,  r =   0 ,  b =   2 ,  l =   0 )), 
            strip.text.y =   element_text ( size =  Smallfont,  colour =   "black" ,  margin =   margin ( t =   2 ,  r =   0 ,  b =   2 ,  l =   0 )), 
            strip.background =   element_rect ( fill=  NA ,  colour=  "black" ), 
            strip.placement=  "outside" ) 
    
   Plot_Fig4S1D    
   
 
 
  4.2.5  Figure 4S1E 
 
 Midgut size is a plastic, diet-dependent trait. Midguts of flies shifted between HS or HY can reversibly shrink throughout 21 days. Statistical comparison is vs pre-shift measurement. 
 
      Length_shift_Shrink  =   
     d[[ "4C', 4S1D'" ]] %>%  
      select ( - Total.PH3) %>%  
      mutate ( Total_Length_mm =  Total.L /  1000 ) %>%  
      mutate_if (is.character,as.factor) %>%  
      mutate_if (is.integer,as.factor) %>%  
     dplyr ::  rename ( Day_of_treatment= Day) %>%  
      as.data.frame () %>%  
      mutate ( Dday=  fct_relevel (Dday, "Shift Day 7" , "Shift Day 14" , "Shift Day 21" )) 
      
   Sample_size =  
     Length_shift_Shrink %>%  
      group_by (Dday,Diet) %>%  
      summarise ( Sample_size=  n ()) 
    
    ###Stats  
    # Day 7  
   tmp =   subset (Length_shift_Shrink,Dday ==  "Shift Day 7" ) 
   mod.gen  =   fitme ( log (Total_Length_mm)  ~   Diet  +  ( 1   |  Repeat),  data =  tmp) 
    shapiro.test ( residuals (mod.gen))    
  ## 
##  Shapiro-Wilk normality test
## 
## data:  residuals(mod.gen)
## W = 0.96958, p-value = 0.2925  
       bptest ( log (Total_Length_mm)  ~  Diet  +  ( 1   /  Repeat),  data =  tmp)    
  ## 
##  studentized Breusch-Pagan test
## 
## data:  log(Total_Length_mm) ~ Diet + (1/Repeat)
## BP = 0.0069323, df = 1, p-value = 0.9336  
      mod.gen1  =   fitme ( log (Total_Length_mm)  ~   1   +  ( 1   |  Repeat),  data =  tmp) 
   test  =   anova (mod.gen, mod.gen1) 
   Chi2_LRT_growth  =   2  * (mod.gen $ APHLs[[ "p_v" ]] - mod.gen1 $ APHLs[[ "p_v" ]]) 
    
   tab_stat  =   data.frame ( Comparison =   as.character ( paste (( "HS Day 7 vs HS to HY Day 14" ))), 
      Dday =   as.character ( paste ( "Shift Day 7" )), 
                          Rep =   nlevels (tmp $ Repeat), 
                          chi2_LR =   round ( as.numeric (test $ basicLRT $ chi2_LR),  digits =   2 ), 
                          intercept =   format (mod.gen $ fixef[ 1 ], digits=  3 ), 
                          estimate =   format (mod.gen $ fixef[ 2 ], digits=  3 ), 
                          df =   as.numeric (test $ basicLRT $ df), 
                          Pvalue =   as.numeric ( format ( pchisq (Chi2_LRT_growth, df=  1 , lower.tail =  F), digits=  2 ))) 
   tab_stat_7  =  tab_stat 
    
    # Day 14  
   tmp =   subset (Length_shift_Shrink,Dday ==  "Shift Day 14" ) 
   mod.gen  =   fitme ( log (Total_Length_mm)  ~   Diet  +  ( 1   |  Repeat),  data =  tmp) 
    shapiro.test ( residuals (mod.gen))    
  ## 
##  Shapiro-Wilk normality test
## 
## data:  residuals(mod.gen)
## W = 0.9797, p-value = 0.6218  
       bptest ( log (Total_Length_mm)  ~  Diet  +  ( 1   /  Repeat),  data =  tmp)    
  ## 
##  studentized Breusch-Pagan test
## 
## data:  log(Total_Length_mm) ~ Diet + (1/Repeat)
## BP = 2.953, df = 1, p-value = 0.08572  
      mod.gen1  =   fitme ( log (Total_Length_mm)  ~   1   +  ( 1   |  Repeat),  data =  tmp) 
   test  =   anova (mod.gen, mod.gen1) 
   Chi2_LRT_growth  =   2  * (mod.gen $ APHLs[[ "p_v" ]] - mod.gen1 $ APHLs[[ "p_v" ]]) 
    
   tab_stat  =   data.frame ( Comparison =   as.character ( paste (( "HS Day 14 vs HS to HY Day 21" ))), 
                          Dday =   as.character ( paste ( "Shift Day 14" )), 
                          Rep =   nlevels (tmp $ Repeat), 
                          chi2_LR =   round ( as.numeric (test $ basicLRT $ chi2_LR),  digits =   2 ), 
                          intercept =   format (mod.gen $ fixef[ 1 ], digits=  3 ), 
                          estimate =   format (mod.gen $ fixef[ 2 ], digits=  3 ), 
                          df =   as.numeric (test $ basicLRT $ df), 
                          Pvalue =   as.numeric ( format ( pchisq (Chi2_LRT_growth, df=  1 , lower.tail =  F), digits=  2 ))) 
   tab_stat_14  =  tab_stat 
    
    # Day 21  
   tmp =   subset (Length_shift_Shrink,Dday ==  "Shift Day 21" ) 
   mod.gen  =   fitme ( log (Total_Length_mm)  ~   Diet  +  ( 1   |  Repeat),  data =  tmp) 
    shapiro.test ( residuals (mod.gen))    
  ## 
##  Shapiro-Wilk normality test
## 
## data:  residuals(mod.gen)
## W = 0.9841, p-value = 0.7537  
       bptest ( log (Total_Length_mm)  ~  Diet  +  ( 1   /  Repeat),  data =  tmp)    
  ## 
##  studentized Breusch-Pagan test
## 
## data:  log(Total_Length_mm) ~ Diet + (1/Repeat)
## BP = 0.11454, df = 1, p-value = 0.735  
      mod.gen1  =   fitme ( log (Total_Length_mm)  ~   1   +  ( 1   |  Repeat),  data =  tmp) 
   test  =   anova (mod.gen, mod.gen1) 
   Chi2_LRT_growth  =   2  * (mod.gen $ APHLs[[ "p_v" ]] - mod.gen1 $ APHLs[[ "p_v" ]]) 
    
   tab_stat  =   data.frame ( Comparison =   as.character ( paste (( "HS Day 21 vs HS to HY Day 28" ))), 
                          Dday =   as.character ( paste ( "Shift Day 21" )), 
                          Rep =   nlevels (tmp $ Repeat), 
                          chi2_LR =   round ( as.numeric (test $ basicLRT $ chi2_LR),  digits =   2 ), 
                          intercept =   format (mod.gen $ fixef[ 1 ], digits=  3 ), 
                          estimate =   format (mod.gen $ fixef[ 2 ], digits=  3 ), 
                          df =   as.numeric (test $ basicLRT $ df), 
                          Pvalue =   as.numeric ( format ( pchisq (Chi2_LRT_growth, df=  1 , lower.tail =  F), digits=  2 ))) 
   tab_stat_21  =  tab_stat 
    
   tab_stat =  rbind (tab_stat_7,tab_stat_14,tab_stat_21) 
    
   tab_stat $ sig  =   ifelse (tab_stat $ Pvalue  <   0.05   &  tab_stat $ Pvalue  >   0.01 ,  "*" , 
                 ifelse (tab_stat $ Pvalue  <   0.01   &  tab_stat $ Pvalue  >   0.001 ,  "**" , 
                  ifelse (tab_stat $ Pvalue  <   0.001 ,  "***" ,  "" ))) 
    
   tab_stat %>%  
      kable ( col.names =   c ( "Comparison" , "Shift day" ,  "Replicates" ,  "Chi2" , "Intercept" , "Estimate" , "df"  , "p-value" , "Signif." ), row.names =   FALSE )  %>%     add_header_above ( c ( "log(Total length) ~ Day + Diet + (1 | Repeat)"   =   9 )) %>%  
      kable_styling ( bootstrap_options =   c ( "striped" ,  "hover" ,  "condensed" ),  full_width =  F)    
 
 
 
 
 
log(Total length) ~ Day + Diet + (1 | Repeat)
 
 
 
 
 
Comparison
 
 
Shift day
 
 
Replicates
 
 
Chi2
 
 
Intercept
 
 
Estimate
 
 
df
 
 
p-value
 
 
Signif.
 
 
 
 
 
 
HS Day 7 vs HS to HY Day 14
 
 
Shift Day 7
 
 
3
 
 
43.00
 
 
1.8
 
 
-0.349
 
 
1
 
 
0.0e+00
 
 
***
 
 
 
 
HS Day 14 vs HS to HY Day 21
 
 
Shift Day 14
 
 
3
 
 
38.48
 
 
1.86
 
 
-0.366
 
 
1
 
 
0.0e+00
 
 
***
 
 
 
 
HS Day 21 vs HS to HY Day 28
 
 
Shift Day 21
 
 
3
 
 
21.89
 
 
1.8
 
 
-0.251
 
 
1
 
 
2.9e-06
 
 
***
 
 
 
 
      tab_stat =  
     tab_stat %>%  
      mutate_if (is.character,as.factor) %>%  
      as.data.frame () %>%  
      mutate ( Dday=  fct_relevel (Dday, "Shift Day 7" , "Shift Day 14" , "Shift Day 21" )) 
    
    ### Plot  
    
   Treatment.status  =   c ( "Shift  \n  Day 7" , "Shift  \n  Day 14" , "Shift  \n  Day 21" ) 
    names (Treatment.status)  =   c ( "Shift Day 7" ,  "Shift Day 14" , "Shift Day 21" ) 
    
   z =  max (Length_shift_Shrink $ Total_Length_mm,  na.rm =   TRUE ) 
    
   Plot_Fig4S1E =  
      ggplot (Length_shift_Shrink,  aes ( x =  Diet,  y =  Total_Length_mm)) +   
      geom_violin ( aes ( fill =  Diet),  draw_quantiles =   c ( 0.25 ,  0.5 ,  0.75 ),  colour =   "black" ,  size =   0.2 , adjust =   0.8 )  +  
      geom_dotplot (  colour =   "black" ,  fill =   "white" ,  binaxis =   "y" ,  stackdir =   "center" ,  binwidth =   0.15 )  +   
      facet_grid (.  ~  Dday, labeller=  labeller ( Dday= Treatment.status) ) +  
      geom_text ( data =  Sample_size,  mapping =   aes ( x =  Diet,  y =   1.6 ,  label =   paste ( "(" ,Sample_size, ")" , sep=  "" )), size=  3 ) +  
          geom_signif ( data =  tab_stat,  aes ( xmin =   1 ,  xmax =   2 ,  annotations =   formatC ( paste ( "p=" ,Pvalue),  digits =   2 ),  y_position =   9 ),  textsize =   2.5 ,  vjust =   -  0.2 ,  manual =   TRUE ) +  
    
      scale_fill_manual ( limits=  c ( "HY" , "HYtoHS" ), 
                        values= cbbHY_HYtoHS) +  
      scale_x_discrete ( "" , 
                       limits=  c ( "HY" , "HYtoHS" ), 
                       labels=  c ( "HY" , "HY to HS" )) +  
      scale_y_continuous ( "Midgut length (mm)" , 
                         limits=  c ( 1.5 , 9.5 ), 
                         breaks=  seq ( 2 , 8 , by=  1 )) +  
      stat_summary ( fun =  mean,  geom =   "point" ,  size =   2.5 ,  shape =   18 , aes ( group= Repeat,  colour =  Repeat))  +  
      stat_summary ( fun =  mean,  geom =   "point" ,  size =   3 ,  shape =   18 ,  colour =   "black" ,  aes ( group =  Repeat))  +  
      stat_summary ( fun =  mean,  geom =   "point" ,  size =   2 ,  shape =   18 ,  aes ( group =  Repeat,  colour =  Repeat))  +  
      scale_color_manual ( values =  palette_mean)  +  
      theme ( panel.grid.major.y =   element_line ( colour =   grey ( 0.45 ),  linetype =   "dashed" ,  size =   0.2 ), 
            panel.background =   element_blank (), 
            axis.title.x =   element_text ( size= Smallfont, colour=  "black" ), 
            axis.title.y =   element_text ( size= Smallfont, colour=  "black" ),  
            axis.line.x =   element_line ( colour=  "black" , size=  0.75 ), 
            axis.line.y =   element_line ( colour=  "black" , size=  0.75 ), 
            axis.ticks.x =   element_line ( size =   0.75 ), 
            axis.ticks.y =   element_line ( size =   0.75 ), 
            axis.text.x =   element_text ( size= Smallfont, colour=  "black" , angle=  30 , hjust=  1 ), 
            axis.text.y =   element_text ( size= Smallfont, colour=  "black" ), 
            plot.margin =   unit (Margin,  "cm" ), 
            legend.direction =   "vertical" ,  
            legend.box =   "horizontal" , 
            legend.position =   "none" , 
            legend.key.height =   unit ( 0.4 ,  "cm" ), 
            legend.key.width=   unit ( 0.6 ,  "cm" ), 
            legend.title =   element_text ( face=  "italic" , size= Smallfont),  
            legend.key =   element_rect ( colour =   'white' ,  fill =   "white" ,  linetype=  'dashed' ), 
            legend.text =   element_text ( size= SuperSmallfont), 
            legend.background =   element_rect ( fill=  NA ), 
            strip.text.x =   element_text ( size =  Smallfont,  colour =   "black" ,  margin =   margin ( t =   2 ,  r =   0 ,  b =   2 ,  l =   0 )), 
            strip.text.y =   element_text ( size =  Smallfont,  colour =   "black" ,  margin =   margin ( t =   2 ,  r =   0 ,  b =   2 ,  l =   0 )), 
            strip.background =   element_rect ( fill=  NA ,  colour=  "black" ), 
            strip.placement=  "outside" ) 
    
   Plot_Fig4S1E    
   
 
 
  4.2.6  Figure 4S1F 
 
 ISC proliferation is promoted by yeast and antagonized by sugar. Cell proliferation (pH3 stain) is impeded by dietary sucrose, and increased by yeast, similar to total midgut length (Figure 2A). Plots showing counts of pH3+ cells as a function of ingested yeast and sucrose. 
 
      tab_nutri_geo_PH3  =   
     d[[ "4 - S1F" ]] 
    
    jpeg ( filename =    "Plot_Fig4-S1F.jpeg" , 
         res =   600 , 
         width =   5 ,  height =   4 ,  units =   'in'  ) 
    par ( cex=  1 ,  mar =   c ( 4.5 ,  4.5 ,  1 ,  3 )) 
    with (tab_nutri_geo_PH3,  geomPlotta ( x =  Sucrose.in.Diet,  y =  Yeast.in.Diet,  z =  Ph3.Total,  alf =   1 ,  xlim =   c ( -  10 ,  300 ),  ylim =   c ( -  10 ,  300 ),  xlab =   "Sucrose in diet (g/L)" ,  ylab =   "Yeast in diet (g/L)" ,  frame.plot=   FALSE ,  cex.lab=  1.2 ,  cex.axis =  1 ,  las=  1 ,  labcex=  1 ,  asp=  1 ))    
      img4S1F  =   readImage ( "D:/Dropbox/z_ Ale Shared work/z_Nutrition Paper Markdown/Ale/Revision/Plot_Fig4-S1F.jpeg" )  
   gob_imageFig4S1F  =   rasterGrob (img4S1F) 
    grid.draw (gob_imageFig4S1F)    
   
 
 
  4.2.7  Figure 4S1G 
 
 Plots showing pH3+ cell counts increase with dietary yeast, with an optimum around HY diet. Increasing sucrose reduces counts of pH3+ cells. 
 
      tab_nutri_geo_PH3  =   
     d[[ "4 - S1F" ]] 
    
   tab_nutri_geo_PH32  <-  tab_nutri_geo_PH3  %>%  
        group_by (concatenate)  %>%  
        summarize ( Calories.ingested =   mean (Calories.ingested), 
                  Ph3.Total =   mean (Ph3.Total), 
                  Yeast.ingested =   mean (Yeast.ingested), 
                  Sucrose.ingested =   mean (Sucrose.ingested)) 
    
   graph  <-   ggplot (tab_nutri_geo_PH32,  aes ( x= Sucrose.ingested,  y= Yeast.ingested)) 
   Plot_Fig4S1G =  
    graph  +   geom_point ( aes ( size= Calories.ingested,  fill= Ph3.Total),  stroke=  1 ,  shape=  21 ,   color=  "black" )  +  
      scale_size ( range =   c ( 1 , 5 ))  +  
      scale_fill_viridis_c ()  +  
      theme ( plot.title=   element_text ( hjust =   0.5 )) +  
    scale_x_continuous ( "Sucrose ingested (g/L x Absorbance)" , 
                         limits=  c ( -  5 , 160 ), 
                         breaks=  seq ( 0 , 160 , by=  25 )) +  
      scale_y_continuous ( "Yeast ingested (g/L x Absorbance)" , 
                         limits=  c ( -  5 , 50 ), 
                         breaks=  seq ( 0 , 50 , by=  10 )) +  
        scale_size_continuous ( range =   c ( 1 , 5 ))  +  
        theme ( panel.background =   element_blank (), 
            panel.grid.major =   element_line ( colour =   "black" , linetype=  3 ), 
            axis.title.x =   element_text ( size= Smallfont, colour=  "black" ),  
            axis.title.y =   element_text ( size= Smallfont, colour=  "black" ),  
            axis.line.x =   element_line ( colour=  "black" , size=  0.75 ), 
            axis.line.y =   element_line ( colour=  "black" , size=  0.75 ), 
            axis.ticks.x =   element_line ( size =   0.75 ), 
            axis.ticks.y =   element_line ( size =   0.75 ), 
            axis.text.x =   element_text ( size= Smallfont, colour=  "black" ),  
            axis.text.y =   element_text ( size= Smallfont, colour=  "black" ), 
            plot.margin =   unit ( c ( 0 , 0 , 0 , 0.5 ),  "cm" ), 
            legend.direction =   "horizontal" ,  
            legend.box =   "vertical" , 
            legend.position =   c ( 0.75 , 0.79 ), 
           legend.key.height =   unit ( 0.3 ,  "cm" ), 
            legend.key.width=   unit ( 0.3 ,  "cm" ), 
            legend.title =   element_text ( face=  "italic" , size= Smallfont),  
            legend.key =   element_rect ( colour =   'white' ,  fill =   "white" ,  linetype=  'dashed' ), 
            legend.text =   element_text ( size= SuperSmallfont), 
            legend.box.background =   element_rect ( fill=  "white" ,  colour =  "black" ), 
            legend.spacing.y =   unit ( 0 ,  "cm" )) +  
      labs ( fill =   expression ( paste ( "pH3"   ^   "+" ,  " cells" )),  size =   "Calories  
    ingested" ,  vjust=  "center"  ) 
    
   Plot_Fig4S1G    
   
 ##Export Figure 4S1 
 
 
 
  4.3  Figure 4 - supplementary 2 
 
  4.3.1  Figure 4S2A 
 
 Illustration of the cell loss assay (Figure 4G - L). A pulse of RU486 for 3 days marks all ECs and EBs through 5966GS>His-2BRFP. Flies were dissected at 2 and 16-days after cessation of hormone pulse. 
 
      img4S2A  =   readImage ( "D:/Dropbox/z_ Ale Shared work/z_Nutrition Paper Markdown/Ale/Revision/4 - S2A.jpg" )  
   gob_imageFig4S2A  =   rasterGrob (img4S2A) 
    grid.draw (gob_imageFig4S2A)    
   
 
 
  4.3.2  Figure 4S2B 
 
 His2B-RFP is highly stable on both HS and HY diets on tissues not undergoing turnover in a manner similar to the midgut. We assayed the stability of the His2B-RFP by driving it through an ActGS driver, in the crop and hindgut in the same timeline as the experiment presented in Figure 4K. In both organs, we found a high degree of cells marked by His2B-RFP, and on both diets, at both the initial timepoint and after 14 days from the start of the chase. Day of dissection at the bottom of the chart are relative to start of pulse chase. 
 
      tab_Hisstab_rev  =   
     d[[ "4S2B" ]] %>%  
      mutate_at ( vars ( !  starts_with ( "RFP" )),as.factor) 
    
    
    
    ###Stats  
    
    #Crop  
   tmp  =   subset (tab_Hisstab_rev , Tissue %in%  c ( "Crop" )) 
    
   mod.gen  =   fitme ((RFP.Dapi)  ~   Day  *  Diet  +  ( 1   |  Repeat), data =  tmp) 
    shapiro.test ( residuals (mod.gen))    
  ## 
##  Shapiro-Wilk normality test
## 
## data:  residuals(mod.gen)
## W = 0.97787, p-value = 0.3043  
       bptest ((RFP.Dapi)  ~   Day  *  Diet  +  ( 1   /  Repeat), data =  tmp)    
  ## 
##  studentized Breusch-Pagan test
## 
## data:  (RFP.Dapi) ~ Day * Diet + (1/Repeat)
## BP = 11.797, df = 3, p-value = 0.008111  
      mod.gen1  =   fitme ((RFP.Dapi)  ~   Day  +  Diet  +  ( 1   |  Repeat), data =  tmp) 
   test  =   anova (mod.gen, mod.gen1) 
   test    
  ##       chi2_LR df   p_value
## p_v 0.3811947  1 0.5369646  
      Chi2_LRT_growth  =   2  * (mod.gen $ APHLs[[ "p_v" ]] - mod.gen1 $ APHLs[[ "p_v" ]]) 
    
    
   tab_stat  =   data.frame ( Tissue =   as.character ( paste ( "Crop" )), 
                                   Rep =   nlevels (tmp $ Repeat), 
                                   chi2_LR =   round ( as.numeric (test $ basicLRT $ chi2_LR),  digits =   2 ), 
                                   intercept =   format (mod.gen $ fixef[ 1 ], digits=  3 ), 
                                   estimate =   format (mod.gen $ fixef[ 2 ], digits=  3 ), 
                                   df =   as.numeric (test $ basicLRT $ df), 
                                   Pvalue =   as.numeric ( format ( pchisq (Chi2_LRT_growth, df=  1 , lower.tail =  F), digits=  1 , scientific= F))) 
    
   tab_stat_Crop = tab_stat 
    
    #Hindgut  
   tmp  =   subset (tab_Hisstab_rev , Tissue %in%  c ( "Hindgut" )) 
    
   mod.gen  =   fitme ((RFP.Dapi)  ~   Day  *  Diet  +  ( 1   |  Repeat), data =  tmp) 
    shapiro.test ( residuals (mod.gen))    
  ## 
##  Shapiro-Wilk normality test
## 
## data:  residuals(mod.gen)
## W = 0.63759, p-value = 2.106e-12  
       bptest ((RFP.Dapi)  ~   Day  *  Diet  +  ( 1   /  Repeat), data =  tmp)    
  ## 
##  studentized Breusch-Pagan test
## 
## data:  (RFP.Dapi) ~ Day * Diet + (1/Repeat)
## BP = 1.526, df = 3, p-value = 0.6763  
      mod.gen1  =   fitme ((RFP.Dapi)  ~   Day  +  Diet  +  ( 1   |  Repeat), data =  tmp) 
   test  =   anova (mod.gen, mod.gen1) 
   test    
  ##         chi2_LR df   p_value
## p_v 0.008096241  1 0.9283038  
      Chi2_LRT_growth  =   2  * (mod.gen $ APHLs[[ "p_v" ]] - mod.gen1 $ APHLs[[ "p_v" ]]) 
    
    
   tab_stat  =   data.frame ( Tissue =   as.character ( paste ( "Hindgut" )), 
                                   Rep =   nlevels (tmp $ Repeat), 
                                   chi2_LR =   round ( as.numeric (test $ basicLRT $ chi2_LR),  digits =   2 ), 
                                   intercept =   format (mod.gen $ fixef[ 1 ], digits=  3 ), 
                                   estimate =   format (mod.gen $ fixef[ 2 ], digits=  3 ), 
                                   df =   as.numeric (test $ basicLRT $ df), 
                                   Pvalue =   as.numeric ( format ( pchisq (Chi2_LRT_growth, df=  1 , lower.tail =  F), digits=  1 , scientific= F))) 
    
   tab_stat_Hindgut = tab_stat 
    
    
    #Table  
   tab_stat =  rbind (tab_stat_Crop,tab_stat_Hindgut) 
   tab_stat $ padj  =   as.numeric ( format ( p.adjust (tab_stat $ Pvalue,  method =   "BH" ), digits=  2 , scientific = F)) 
   tab_stat $ sig  =   ifelse (tab_stat $ padj  <   0.05   &  tab_stat $ padj  >   0.01 ,  "*" , 
                 ifelse (tab_stat $ padj  <   0.01   &  tab_stat $ padj  >   0.001 ,  "**" , 
                  ifelse (tab_stat $ padj  <   0.001 ,  "***" ,  "" ))) 
    
   tab_stat %>%  
      kable ( col.names =   c ( "Variable" ,  "Replicates" ,  "Chi2" , "Intercept" , "Estimate" , "df"  , "p-value" , "p-value adjusted" , "Signif." ), row.names =   FALSE )  %>%  
      add_header_above ( c ( "(RFP/Dapi) ~  Diet + Genotype + Diet : Genotype + (1 | Repeat)"   =   9 )) %>%  
      kable_styling ( bootstrap_options =   c ( "striped" ,  "hover" ,  "condensed" ),  full_width =  F)    
 
 
 
 
 
(RFP/Dapi) ~ Diet + Genotype + Diet : Genotype + (1 | Repeat)
 
 
 
 
 
Variable
 
 
Replicates
 
 
Chi2
 
 
Intercept
 
 
Estimate
 
 
df
 
 
p-value
 
 
p-value adjusted
 
 
Signif.
 
 
 
 
 
 
Crop
 
 
3
 
 
0.38
 
 
84.4
 
 
4.21
 
 
1
 
 
0.5
 
 
0.9
 
 
 
 
 
 
Hindgut
 
 
3
 
 
0.01
 
 
99.1
 
 
-0.00928
 
 
1
 
 
0.9
 
 
0.9
 
 
 
 
 
 
      tab_stat_rev_Hisstab = tab_stat 
   tab_stat_rev_Hisstab $ Diet  =   "HY"  
    
    
   Sample_size =  
     tab_Hisstab_rev %>%  
      group_by (Diet, Day, Tissue) %>%  
      summarise ( Sample_size=  n ()) 
    
    
    ### Plot  
   Limits  =   c ( "0" , "14" ) 
    
   Plot_Fig4S2B =  
      ggplot (tab_Hisstab_rev,  aes ( x =  Day,  y =  RFP.Dapi)) +   
      geom_violin ( aes ( fill =  Diet),  draw_quantiles =   c ( 0.25 ,  0.5 ,  0.75 ),  colour =   "black" ,  size =   0.2 , adjust =   0.8 )  +  
      geom_dotplot (  colour =   "black" ,  fill =   "white" ,  binaxis =   "y" ,  stackdir =   "center" ,  binwidth =   4 )  +   
      facet_grid (Tissue  ~  Diet, labeller= label_parsed) +  
      geom_text ( data =  Sample_size,  mapping =   aes ( x =  Day,  y =   10 ,  label =   paste ( "(" ,Sample_size, ")" , sep=  "" )), size=  3 ) +  
      scale_fill_manual ( limits=  c ( "HS" , "HY" ), 
                        values=  c ( "#FFB4B4" , "#C3E6FC" )) +  
      scale_x_discrete ( "Day of dissection post RU486 pulse" , 
                       limits= Limits, 
                       labels=  c ( "0" , "14" )) +  
      scale_y_continuous ( expression ( paste ( "RFP pos. cells / Total cells %" )), 
                         limits=  c ( 0 , 110 ), 
                         breaks=  seq ( 0 , 100 , by=  20 )) +  
      stat_summary ( fun =  mean,  geom =   "point" ,  size =   3 ,  shape =   18 ,  colour =   "black" ,  aes ( group =  Repeat))  +  
      stat_summary ( fun =  mean,  geom =   "point" ,  size =   2 ,  shape =   18 ,  aes ( group =  Repeat,  colour =  Repeat))  +  
      stat_summary ( fun =  mean,  colour =   "black" ,  geom =   "line" ,  aes ( group =  Repeat))  +  
      scale_color_manual ( values =  palette_mean)  +  
      theme ( panel.grid.major.y =   element_line ( colour =   grey ( 0.45 ),  linetype =   "dashed" ,  size =   0.2 ), 
            panel.background =   element_blank (), 
            axis.title.x =   element_text ( size= Smallfont, colour=  "black" ), 
            axis.title.y =   element_text ( size= Smallfont, colour=  "black" ,  margin =   margin ( t =   0 ,  r =   0 ,  b =   0 ,  l =   0 ) ),  
            axis.line.x =   element_line ( colour=  "black" , size=  0.75 ), 
            axis.line.y =   element_line ( colour=  "black" , size=  0.75 ), 
            axis.ticks.x =   element_line ( size =   0.75 ), 
            axis.ticks.y =   element_line ( size =   0.75 ), 
            axis.text.x =   element_text ( size= Smallfont, colour=  "black" ), 
            axis.text.y =   element_text ( size= Smallfont, colour=  "black" ), 
            plot.margin =   unit (Margin,  "cm" ), 
            legend.direction =   "vertical" ,  
            legend.box =   "horizontal" , 
            legend.position =   "none" , 
            legend.key.height =   unit ( 0.4 ,  "cm" ), 
            legend.key.width=   unit ( 0.6 ,  "cm" ), 
            legend.title =   element_text ( face=  "italic" , size= Smallfont),  
            legend.key =   element_rect ( colour =   'white' ,  fill =   "white" ,  linetype=  'dashed' ), 
            legend.text =   element_text ( size= SuperSmallfont), 
            legend.background =   element_rect ( fill=  NA ), 
            strip.text =   element_text ( size = Smallfont -2 ,  colour =   "black" , face=  "italic" ,  margin =   margin ( t =   2 ,  r =   1 ,  b =   2 ,  l =   1 )), 
            strip.background =   element_rect ( fill=  NA ,  colour=  "black" ), 
            strip.placement=  "outside" ) 
    
   Plot_Fig4S2B    
   
 
 
  4.3.3  Figure 4S2C 
 
 Cell loss assay performed at 5 days post start of chase shows limited cell loss in HY to HY condition. Number of ECs in the posterior midgut, both marked (Red, old ECs) and unmarked (Blue, new ECs) by RFP, error bars are SE from 3 repeats. 
 
      tab_prop_cell_RFP_kin  =   
     d[[ "4S2C" ]] %>%  
      mutate_if (is.character,as.factor) %>%  
      mutate_if (is.integer,as.factor) %>%  
      mutate ( Post.Dapi.Number =  (Post.Dapi.Number) *  2 ) %>%  
      mutate ( Post.RFP.Number =  (Post.RFP.Number) *  2 ) %>%  
      mutate ( Post.NonRFP.Number =  (Post.Dapi.Number  -  Post.RFP.Number)) %>%  
      group_by (Day, Diet, CG, Experiment) %>%  
      summarise ( mean_RFP_positive=  mean (Post.RFP.Number, na.rm= T), 
                se_RFP_positive=  se (Post.RFP.Number), 
                mean_RFP_negative=  mean (Post.NonRFP.Number, na.rm= T), 
                se_RFP_negative=  se (Post.NonRFP.Number)) %>%  
      mutate ( group=  paste (Diet,Experiment, sep=  "_" )) %>%  
      as.data.frame () 
    
    
    attach (tab_prop_cell_RFP_kin) 
    for  (i  in   1  :  length (Experiment)){ 
       tab_prop_cell_RFP_kin $ se_proportionGraphPlus_pos[i]  =  mean_RFP_positive[i] + se_RFP_positive[i] 
       tab_prop_cell_RFP_kin $ se_proportionGraphMinus_pos[i]  =  mean_RFP_positive[i] - se_RFP_positive[i] 
       tab_prop_cell_RFP_kin $ se_proportionGraphPlus_neg[i]  =  mean_RFP_positive[i] + mean_RFP_negative[i] + se_RFP_negative[i] 
       tab_prop_cell_RFP_kin $ se_proportionGraphMinus_neg[i]  =  mean_RFP_positive[i] + mean_RFP_negative[i] - se_RFP_negative[i] 
   } 
    
   tmp1  =  tab_prop_cell_RFP_kin[, c ( "Day" ,  "Diet" ,  "CG" ,  "Experiment" , "mean_RFP_positive" ,  "se_RFP_positive" , "se_proportionGraphPlus_pos" ,  "se_proportionGraphMinus_pos" )] 
   tmp1 $ RFP =  "Positive"  
   tmp1 = dplyr ::  rename (tmp1, mean_RFP =  mean_RFP_positive, 
                se_RFP =  se_RFP_positive, 
                se_proportionGraphPlus = se_proportionGraphPlus_pos, 
                se_proportionGraphMinus=  se_proportionGraphMinus_pos) 
    
   tmp2  =  tab_prop_cell_RFP_kin[, c ( "Day" ,  "Diet" ,  "CG" ,  "Experiment" , "mean_RFP_negative" ,  "se_RFP_negative" ,  "se_proportionGraphMinus_neg"  , "se_proportionGraphPlus_neg" )] 
   tmp2 $ RFP =  "Negative"  
   tmp2 = dplyr ::  rename (tmp2, mean_RFP =  mean_RFP_negative,  
                se_RFP =  se_RFP_negative, 
                se_proportionGraphPlus = se_proportionGraphPlus_neg, 
                se_proportionGraphMinus=  se_proportionGraphMinus_neg) 
     
   tab_prop_cell_RFP_kin  =   rbind (tmp1,tmp2) 
   tab_prop_cell_RFP_kin  =   
     tab_prop_cell_RFP_kin %>%  
      mutate_if (is.numeric,round, 0 ) %>%  
      mutate_if (is.character,as.factor) 
    
    
    
    levels (tab_prop_cell_RFP_kin $ Diet)  <-   c ( "HS" ,  "HS to HS"  ,  "HS to HY" ,  "HY" ,  "HY to HS" ,  "HY to HY" ) 
    
   Sample_size =  
       d[[ "4S2C" ]] %>%  
      mutate_if (is.character,as.factor) %>%  
      mutate_if (is.integer,as.factor) %>%  
      group_by (Diet) %>%  
      summarise ( Sample_size=  n ()) 
    
   Sample_size $ Experiment  =   c ( "G" ,  "G" ,  "G" ,  "S" ,  "S" ,  "S" ) 
    
    levels (Sample_size $ Diet)  <-   c ( "HS" ,  "HS to HS"  ,  "HS to HY" ,  "HY" ,  "HY to HS" ,  "HY to HY" ) 
    
                                      
   Plot_Fig4S2C  =  
      ggplot (tab_prop_cell_RFP_kin,  aes ( x= Diet,  y= mean_RFP)) +   
      geom_bar ( stat=  "identity" , aes ( fill= RFP), color=  "black" , width= . 90 ) +  
      geom_errorbar ( aes ( ymin=  se_proportionGraphMinus,  ymax=  se_proportionGraphPlus), width=  0.25 ) +  
      geom_text ( data =  Sample_size,  mapping =   aes ( x =  Diet,  y =   200 ,  label =   paste ( "(" ,Sample_size, ")" , sep=  "" )), size=  3 ) +  
      #facet_wrap(.~Experiment,scales="free_x")+  
      scale_fill_manual ( name =   "RFP labelling" ,  
                        values=  c ( "#3a5ecc" , "#cc0000" ), 
                        labels =   c ( "Negative" ,  "Positive" )) +  
      scale_y_continuous ( "Number of cells (mean \u00B1se)" , 
                         limits=  c ( 0 , 5800 ), 
                         breaks=  seq ( 0 , 5000 , by=  500 )) +  
      theme ( 
        panel.grid.major.y =   element_line ( colour =   grey ( 0.45 ),  linetype =   "dashed" ,  size =   0.2 ), 
        panel.background =   element_blank (), 
        axis.title.x =   element_blank (), 
        axis.title.y =   element_text ( size= Smallfont, colour=  "black" ),  
        axis.line.x =   element_line ( colour=  "black" , size=  0.75 ), 
        axis.line.y =   element_line ( colour=  "black" , size=  0.75 ), 
        axis.ticks.x =   element_line ( size =   0.75 ), 
        axis.ticks.y =   element_line ( size =   0.75 ), 
        axis.text.x =   element_text ( size= Smallfont, colour=  "black" , angle=  30 , hjust=  1 ), 
        axis.text.y =   element_text ( size= Smallfont, colour=  "black" ), 
        plot.margin =   unit (Margin,  "cm" ), 
        legend.direction =   "vertical" ,  
        legend.box =   "horizontal" , 
        legend.position =   c ( 0.2 , 0.87 ), 
        legend.key.height =   unit ( 0.4 ,  "cm" ), 
        legend.key.width=   unit ( 0.6 ,  "cm" ), 
        legend.title =   element_text ( face=  "italic" , size= Smallfont),  
        legend.key =   element_rect ( colour =   'white' ,  fill =   "white" ,  linetype=  'dashed' ), 
        legend.text =   element_text ( size= Smallfont), 
        legend.background =   element_rect ( fill=  NA ), 
        strip.text =   element_blank ()) 
        #strip.background = element_rect(fill=NA, colour="black"),  
        #strip.placement="outside")  
    
   Plot_Fig4S2C    
   
 
 
  4.3.4  Figure 4S2D 
 
 Diet composition modulates cell replacement rate (cell/day). Bar chart was made using the same data as in Figure 4 K, L. 
 
      tab_relative_cell_rate_supp  =   
     d[[ "4L, 4S2D" ]] %>%  
      select ( -  starts_with ( "X" )) %>%  
      drop_na () %>%  
    mutate_if (is.character,as.factor) %>%  
      group_by (Diet1, Experiment, Experiment2, GL) %>%  
      summarise ( mean_Daily=  mean (Daily, na.rm= T)) %>%  
                mutate ( group=  paste (Diet1,Experiment, sep=  "_" )) %>%  
      as.data.frame () 
    
    
    
   tab_relative_cell_rate_ratio  =   
     tab_relative_cell_rate_supp %>%  
      group_by (Diet1,Experiment2) %>%  
      summarize ( Ratio =   round (mean_Daily[GL  ==   "Gain" ]  /  ( - mean_Daily[GL  ==   "Loss" ]), 2 )) %>%  
      mutate ( GL=  "Loss" ) 
    
    levels (tab_relative_cell_rate_supp $ Diet1)  <-   c ( "HS to HS"  ,  "HS to HY" ,  "HY to HS" ,  "HY to HY" ) 
    levels (tab_relative_cell_rate_ratio $ Diet1)  <-   c ( "HS to HS"  ,  "HS to HY" ,  "HY to HS" ,  "HY to HY" ) 
    
    
    
    
    
   Plot_Fig4S2D  =  
      ggplot (tab_relative_cell_rate_supp,  aes ( x =  Diet1,  y =  mean_Daily,  fill =  GL)) +   
      geom_bar ( stat=  "identity" , aes ( fill= GL), color=  "black" , width= . 90 ) +  
      geom_hline ( yintercept =   0 ) +  
      geom_text ( data= tab_relative_cell_rate_ratio, mapping=  aes ( x= Diet1, y=  -  20 , label= Ratio),  color =   "red" ) +  
      facet_grid (. ~ Experiment2, scales=  "free_x" ) +  
    
      scale_fill_manual ( name =   "Enterocyte" ,  
                        values=  c ( "palegreen" ,  "moccasin" ), 
                        labels =   c ( "Gain" ,  "Loss" )) +  
      scale_y_continuous ( "Cell rate (cell/ day)" , 
                         limits=  c ( -  220 , 220 ), 
                         breaks=  seq ( -  200 , 200 , by=  50 )) +  
      theme ( 
        panel.grid.major.y =   element_line ( colour =   grey ( 0.45 ),  linetype =   "dashed" ,  size =   0.2 ), 
        panel.background =   element_blank (), 
        axis.title.x =   element_blank (), 
        axis.title.y =   element_text ( size= Smallfont, colour=  "black" ),  
        axis.line.x =   element_line ( colour=  "black" , size=  0.75 ), 
        axis.line.y =   element_line ( colour=  "black" , size=  0.75 ), 
        axis.ticks.x =   element_line ( size =   0.75 ), 
        axis.ticks.y =   element_line ( size =   0.75 ), 
        axis.text.x =   element_text ( size= Smallfont, colour=  "black" , angle=  30 , hjust=  1 ), 
        axis.text.y =   element_text ( size= Smallfont, colour=  "black" ), 
        plot.margin =   unit (Margin,  "cm" ), 
        legend.direction =   "vertical" ,  
        legend.box =   "horizontal" , 
        legend.position =   c ( 0.18 , 0.88 ), 
        legend.key.height =   unit ( 0.4 ,  "cm" ), 
        legend.key.width=   unit ( 0.6 ,  "cm" ), 
        legend.title =   element_text ( face=  "italic" , size= Smallfont),  
        legend.key =   element_rect ( colour =   'white' ,  fill =   "white" ,  linetype=  'dashed' ), 
        legend.text =   element_text ( size= Smallfont), 
        legend.background =   element_rect ( fill=  NA ), 
        strip.text.x =   element_text ( size =  Smallfont,  colour =   "black" ,  margin =   margin ( t =   2 ,  r =   0 ,  b =   2 ,  l =   0 )), 
        strip.text.y =   element_text ( size =  Smallfont,  colour =   "black" ,  margin =   margin ( t =   2 ,  r =   0 ,  b =   2 ,  l =   0 )), 
        strip.background =   element_rect ( fill=  NA ,  colour=  "black" ), 
        strip.placement=  "outside" ) 
    
    
   Plot_Fig4S2D    
   
 ##Export Figure 4S2 
 
 
 
 
  5  Figure 5. Sugar uncouples ISC proliferation from niche signal expression by inducing translational stress 
 
  5.1  Figure 5 - main 
 
  5.1.1  Figure 5A 
 
 Diet influences midgut transcriptomes after an initial programmed developmental transition. The plot shows a PCA of the whole transcriptome, with means per diet per day ± standard error (3 repeats). Numbers on the plot represent the day of dissection from eclosion. Lines connect the datapoints sequentially (Day 0 to day 1, day 1 to day 2, and so on), and show the divergent transcriptomic trajectory followed by midguts on the two different diets from eclosion. 
 
      dataInd  =  
     d[[ "gutGrowthDataIndex" ]] %>%  
      mutate_if (is.character,as.factor) %>%  
      mutate_if (is.integer,as.factor) 
        #add string to direct to enumerated reads  
   dataInd $ countFile  =   gsub ( ".fastq" ,  "_sn.sam.count" , dataInd $ library) 
   dataInd $ samp  =   paste ( "day" ,dataInd $ day, "_diet" ,dataInd $ diet, "_rep" ,dataInd $ rep, sep=  "" ) 
   dataInd $ expCond  =   factor ( paste (dataInd $ day, dataInd $ diet,  sep=  "" )) 
   dataInd $ dietCol  =   with ( dataInd,  ifelse (diet ==  "x" ,  "#ff9595" ,  ifelse (diet ==  "y" ,  "#abefff" ,  "#67ff67" ))  ) 
   tab_read_RNAseq  =  d[[ "readTable" ]] 
   geneID  =   rownames (tab_read_RNAseq)  =  tab_read_RNAseq $ FBid 
   tab_read_RNAseq  =  tab_read_RNAseq[, 2  :  ncol (tab_read_RNAseq)] 
   normCounts  =  d[[ "normCounts" ]] 
    rownames (normCounts)  =  geneID 
   normCounts  =  normCounts[, 2  :  ncol (normCounts)] 
        #pca without day 4  
   dataInd2  =   droplevels ( subset (dataInd, day !=  4 )) 
   normCounts2  =  normCounts[,dataInd $ day !=  4 ] 
   pca2  =   prcomp ( t (normCounts2))    
    summary (pca2)    
  ## Importance of components:
##                            PC1     PC2      PC3      PC4      PC5     PC6
## Standard deviation     27.1902 19.9723 14.67551 13.46729 10.71975 9.34310
## Proportion of Variance  0.2674  0.1443  0.07789  0.06559  0.04156 0.03157
## Cumulative Proportion   0.2674  0.4116  0.48951  0.55510  0.59666 0.62823
##                            PC7     PC8     PC9    PC10    PC11   PC12    PC13
## Standard deviation     8.98836 8.74368 8.35311 8.18187 8.02046 7.7286 7.69699
## Proportion of Variance 0.02922 0.02765 0.02523 0.02421 0.02326 0.0216 0.02143
## Cumulative Proportion  0.65745 0.68510 0.71033 0.73454 0.75780 0.7794 0.80083
##                           PC14    PC15    PC16    PC17    PC18  PC19    PC20
## Standard deviation     7.55465 7.32756 7.21305 6.84985 6.72589 6.651 6.33040
## Proportion of Variance 0.02064 0.01942 0.01882 0.01697 0.01636 0.016 0.01449
## Cumulative Proportion  0.82147 0.84089 0.85970 0.87667 0.89303 0.909 0.92352
##                           PC21    PC22   PC23   PC24    PC25    PC26      PC27
## Standard deviation     6.22794 6.14888 6.0655 5.9721 5.81879 5.34384 6.139e-14
## Proportion of Variance 0.01403 0.01367 0.0133 0.0129 0.01224 0.01033 0.000e+00
## Cumulative Proportion  0.93755 0.95122 0.9645 0.9774 0.98967 1.00000 1.000e+00  
           #calculate means and SEs of the PCs  
            #without day 4  
   pcMns  =   aggregate (pca2 $ x  ~  day  *  diet  +  dietCol,  data= dataInd2, mean) 
   pcSEs  =   aggregate (pca2 $ x  ~  day  *  diet  +  dietCol,  data= dataInd2,  function (x){ sd (x) /  sqrt ( length (x))}) 
   pcMns  =   droplevels ( subset (pcMns, day !=  4 )) 
   pcSEs  =   droplevels ( subset (pcSEs, day !=  4 )) 
   pcMns  =  pcMns[ order (pcMns $ day),] 
   pcSEs  =  pcSEs[ order (pcSEs $ day),] 
   pcMns  =  pcMns %>%  
      mutate_if (is.character,as.factor) %>%  
      select (day,diet,dietCol,PC1,PC2) %>%  
      as.data.frame () %>%  
     dplyr ::  rename ( PC1_mean= PC1, 
             PC2_mean= PC2) 
   pcSEs  =  pcSEs %>%  
      mutate_if (is.character,as.factor) %>%  
      select (day,diet,dietCol,PC1,PC2) %>%  
     dplyr ::  rename ( PC1_se= PC1, 
             PC2_se= PC2) 
   tab_PCA  =   left_join (pcMns,pcSEs) 
   tab_PCA $ diet1  =  ifelse (tab_PCA $ diet ==  "x" , "#ff9595" , "#abefff" ) 
   tab_PCA $ diet1[ 1 ] =  "#ff9595"  
   tab_PCA $ diet2 =  ifelse (tab_PCA $ diet ==  "y" , "#abefff" , "#ff9595" ) 
   tab_PCA $ diet2[ 1 ] =  "#abefff"  
   tab_PCA =  mutate_if (tab_PCA,is.character,as.factor) 
   tab_PCA $ diet1 =   factor (tab_PCA $ diet1, levels=  c ( "#67ff67" , "#abefff" , "#ff9595" )) 
   tab_PCA $ diet2 =   factor (tab_PCA $ diet2, levels=  c ( "#67ff67" , "#abefff" , "#ff9595" )) 
    
   Plot_Fig5A =  
     ggplot (tab_PCA, aes ( x= PC1_mean,  y= PC2_mean)) +  
      geom_point ( aes ( color= dietCol), size=  2  , shape=  19 ) +  
      geom_errorbarh ( aes ( xmax =  PC1_mean + PC1_se,  xmin = PC1_mean - PC1_se, color= dietCol ), height=  0.1 ) +  
      geom_errorbar ( aes ( ymax =  PC2_mean + PC2_se,  ymin = PC2_mean - PC2_se, color= dietCol ), width =  0.1 ) +  
      geom_path ( size=  0.8 , aes ( group= diet1, color= diet1)) +  
      geom_path ( size=  0.8 , aes ( group= diet2, color= diet2)) +  
      geom_text ( aes ( label= day), size=  4 , vjust =   0 ,  nudge_y =   -  2.5 ) +  
      scale_color_manual ( values=  c ( "#67ff67" , "#abefff" , "#ff9595" )) +  
      scale_x_continuous ( paste ( "PC1 (" ,  round ( summary (pca2) $ importance[ 2 , 1 ] *  100 ),  "%)" ,  sep=  "" ), 
                       limits=  c ( -  50 , 60 ), 
                       breaks=  seq ( -  50 , 60 , by=  20 )) +  
      scale_y_continuous ( paste ( "PC2 (" ,  round ( summary (pca2) $ importance[ 2 , 2 ] *  100 ),  "%)" ,  sep=  "" ), 
                         limits=  c ( -  30 , 45 ), 
                         breaks=  seq ( -  30 , 40 , by=  10 )) +  
      theme ( panel.grid.major.y =   element_line ( colour =   grey ( 0.45 ),  linetype =   "dashed" ,  size =   0.2 ), 
            panel.background =   element_blank (), 
            axis.title.x =   element_text ( size= Smallfont, colour=  "black" ), 
            axis.title.y =   element_text ( size= Smallfont, colour=  "black" ), 
            axis.line.x =   element_line ( colour=  "black" , size=  0.75 ), 
            axis.line.y =   element_line ( colour=  "black" , size=  0.75 ), 
            axis.ticks.x =   element_line ( size =   0.75 ), 
            axis.ticks.y =   element_line ( size =   0.75 ), 
            axis.text.x =   element_text ( size= Smallfont, colour=  "black" ), 
            axis.text.y =   element_text ( size= Smallfont, colour=  "black" ), 
            plot.margin =   unit (Margin,  "cm" ), 
            legend.direction =   "vertical" , 
            legend.box =   "horizontal" , 
            legend.position =   "none" , 
            legend.key.height =   unit ( 0.4 ,  "cm" ), 
            legend.key.width=   unit ( 0.6 ,  "cm" ), 
            legend.title =   element_text ( face=  "italic" , size= Smallfont), 
            legend.key =   element_rect ( colour =   'white' ,  fill =   "white" ,  linetype=  'dashed' ), 
            legend.text =   element_text ( size= SuperSmallfont), 
            legend.background =   element_rect ( fill=  NA ), 
            strip.text.x =   element_text ( size = Smallfont,  colour =   "black" , face=  "italic" ), 
            strip.text.y =   element_text ( size = Smallfont,  colour =   "black" , face=  "italic" ), 
            strip.background =   element_rect ( fill=  NA ,  colour=  "black" ), 
            strip.placement=  "outside" ) 
   Plot_Fig5A    
   
 
 
  5.1.2  Figure 5B 
 
  Diet modulates expression of functionally distinct gene classes.  Midguts of flies fed HY diet show higher expression of genes with digestive functions, while HS diet involves mainly genes attributed to stress response and growth. X-axis represents the statistical significance of the gene ontology (GO) categories (y-axis) after adjustment for multiple testing. Size of the dot is proportional to number of genes in the given GO category 
 
      tab_GO_results  =   
     d[[ "5B" ]] %>%  
      mutate_if (is.character,as.factor) %>%  
      mutate ( Padj=  -  log (p.value, 10 ), 
             Term =   str_to_sentence (Term)) %>%  
      as.data.frame () 
      
   tab_GO_results $ Term  <-   factor (tab_GO_results $ Term,  levels =  tab_GO_results $ Term[ order (tab_GO_results $ Diet, tab_GO_results $ Padj, tab_GO_results $ Significant)]) 
    #order GO categories based on size of Padj  
    
   tab_GO_results $ Diet  <-   factor (tab_GO_results $ Diet,  levels =   c ( "HY" ,  "HS" )) 
    ### Plot  
    
   Plot_Fig5B =  
      ggplot (tab_GO_results,  aes ( x =  Padj,  y =  Term)) +   
      geom_point ( aes ( size= Significant, fill= Diet), shape=  21 )  +  
      facet_grid (Diet ~ ., scales=  "free_y" , space=  "free" ) +  
      scale_fill_manual ( limits=  c ( "HS" , "HY" ), 
                        values=  c ( "#FFB4B4" , "#C3E6FC" )) +  
      geom_vline ( xintercept =   1.3 , linetype=  3 ) +  
      scale_y_discrete ( "GO categories" ) +  
      scale_x_continuous ( expression ( paste ( "-P-value adjusted (" , log[ 10 ], ")" )), 
                         limits=  c ( 0 , 31 ), 
                         breaks=  c ( 0 , seq ( 10 , 30 , by=  10 ))) +  
      scale_size_continuous ( "Nbr genes" ,  range =  c ( 1 , 5 ) ) +  
      theme ( panel.grid.major.y =   element_line ( colour =   grey ( 0.45 ),  linetype =   "dashed" ,  size =   0.2 ), 
            panel.background =   element_blank (), 
            axis.title.x =   element_text ( size= Smallfont, colour=  "black" ), 
            axis.title.y =   element_text ( size= Smallfont, colour=  "black" ),  
            axis.line.x =   element_line ( colour=  "black" , size=  0.75 ), 
            axis.line.y =   element_line ( colour=  "black" , size=  0.75 ), 
            axis.ticks.x =   element_line ( size =   0.75 ), 
            axis.ticks.y =   element_line ( size =   0.75 ), 
            axis.text.x =   element_text ( size= Smallfont, colour=  "black" ), 
            axis.text.y =   element_text ( size= Smallfont -2 , colour=  "black" ), 
            plot.margin =   unit (Margin,  "cm" ), 
            legend.direction =   "horizontal" ,  
            legend.box =   "horizontal" , 
            legend.position =   "top" , 
            legend.key.height =   unit ( 0.4 ,  "cm" ), 
            legend.key.width=   unit ( 0.6 ,  "cm" ), 
            legend.margin=  margin ( b=  0 ,  unit=  'cm' ), 
            legend.title =   element_text ( face=  "italic" , size= Smallfont),  
            legend.key =   element_rect ( colour =   'white' ,  fill =   "white" ,  linetype=  'dashed' ), 
            legend.text =   element_text ( size= Smallfont -2 ), 
            legend.background =   element_rect ( fill=  NA ), 
            strip.text.x =   element_text ( size = Smallfont,  colour =   "black" , face=  "italic" ), 
            strip.text.y =   element_text ( size =  Smallfont,  colour =   "black" ,  margin =   margin ( t =   3 ,  r =   1 ,  b =   3 ,  l =   1 )), 
            strip.background =   element_rect ( fill=  NA ,  colour=  "black" ), 
            strip.placement=  "outside" ) +  
      guides ( fill= F) 
    
   Plot_Fig5B    
   
 
 
  5.1.3  Figure 5C 
 
 Table of genes significantly differently expressed, between HS and HY, as a ratio of HS/HY, representing midgut response to HS and HY diets; additional information on the statistics is found in material and methods; asterisks denote genes significantly different for p-value but not for adjusted p-value 
 
      img  =   readImage ( "D:/Dropbox/z_ Ale Shared work/z_Nutrition Paper Markdown/Ale/Revision/5C.jpg" )  
   gob_imageFig5C  =   rasterGrob (img) 
    grid.draw (gob_imageFig5C)    
   
 
 
  5.1.4  Figure 5D 
 
 Cell proliferation is possible on HS diet when genetically induced. Progenitor-specific (EsgTS) overexpression of a constitutively active form of Ras (UAS-RasV12) and of UAS-Tor-DER (EGFR Active), both known proliferative inducers, allows for increased proliferation on HS diet. P-values on top of the chart refer to comparison vs control, P-values at the bottom refer to comparison between HS and HY for each sample.  Complete statistical annotation on image can be fund in the manuscript’s figures.  
 
      Tab_Ras_PH3_Rev  =   
     d[[ "5D" ]] %>%  
      mutate_at ( vars ( ends_with ( ".L" )), ~ . /  1000 ) %>%  
      mutate_at ( vars ( !  starts_with ( "Total" )),as.factor) %>%  
     dplyr ::  rename ( PH3_positive_cell= Total.PH3, 
                    Diet= Treatment, 
                    Male_Line= Male.Line) %>%  
      mutate ( Cross=  fct_relevel (Cross, "EsgTsXControl" , "EsgTsX64195" ,  "EsgTsXTorDER"  )) 
    
    
    
    
    ###Stats  
    
    ##Control  
   tmp  =   subset (Tab_Ras_PH3_Rev , Male_Line %in%  c ( "Control" )) 
    
    
   mod.gen  =   fitme ( log (PH3_positive_cell +  1 )  ~   Diet  +  ( 1   |  Repeat), data =  tmp) 
    shapiro.test ( residuals (mod.gen))     
  ## 
##  Shapiro-Wilk normality test
## 
## data:  residuals(mod.gen)
## W = 0.98547, p-value = 0.4099  
       bptest ( log (PH3_positive_cell +  1 )  ~   Diet  +  ( 1   /  Repeat), data =  tmp)    
  ## 
##  studentized Breusch-Pagan test
## 
## data:  log(PH3_positive_cell + 1) ~ Diet + (1/Repeat)
## BP = 0.10949, df = 1, p-value = 0.7407  
      mod.gen1  =   fitme ( log (PH3_positive_cell +  1 )  ~    1   +  ( 1   |  Repeat), data =  tmp) 
   test  =   anova (mod.gen, mod.gen1)  
   Chi2_LRT  =   2  * (mod.gen $ APHLs[[ "p_v" ]] - mod.gen1 $ APHLs[[ "p_v" ]]) 
    
   tab_stat  =   data.frame ( Variable =   as.character ( paste ( "HS vs HY Control" )), 
                                   Rep =   nlevels (Tab_Ras_PH3_Rev $ Repeat), 
                                   chi2_LR =   round ( as.numeric (test $ basicLRT $ chi2_LR),  digits =   2 ), 
                                   intercept =   format (mod.gen $ fixef[ 1 ], digits=  3 ), 
                                   estimate =   format (mod.gen $ fixef[ 2 ], digits=  3 ), 
                                   df =   as.numeric (test $ basicLRT $ df), 
                                   Pvalue =   as.numeric ( format ( pchisq (Chi2_LRT, df=  1 , lower.tail =  F), digits=  2 ))) 
    
   tab_stat_Control = tab_stat 
    
    
    ##Ras85DV12 (64195)  
    
   tmp  =   subset (Tab_Ras_PH3_Rev , Male_Line %in%  c ( "Ras85DV12" )) 
    
    
   mod.gen  =   fitme ( log (PH3_positive_cell +  1 )  ~   Diet  +  ( 1   |  Repeat), data =  tmp) 
    shapiro.test ( residuals (mod.gen))     
  ## 
##  Shapiro-Wilk normality test
## 
## data:  residuals(mod.gen)
## W = 0.98728, p-value = 0.5179  
       bptest ( log (PH3_positive_cell +  1 )  ~   Diet  +  ( 1   /  Repeat), data =  tmp)    
  ## 
##  studentized Breusch-Pagan test
## 
## data:  log(PH3_positive_cell + 1) ~ Diet + (1/Repeat)
## BP = 0.16649, df = 1, p-value = 0.6832  
      mod.gen1  =   fitme ( log (PH3_positive_cell +  1 )  ~    1   +  ( 1   |  Repeat), data =  tmp) 
   test  =   anova (mod.gen, mod.gen1)  
   Chi2_LRT  =   2  * (mod.gen $ APHLs[[ "p_v" ]] - mod.gen1 $ APHLs[[ "p_v" ]]) 
    
   tab_stat  =   data.frame ( Variable =   as.character ( paste ( "HS vs HY Ras85DV12" )), 
                                   Rep =   nlevels (Tab_Ras_PH3_Rev $ Repeat), 
                                   chi2_LR =   round ( as.numeric (test $ basicLRT $ chi2_LR),  digits =   2 ), 
                                   intercept =   format (mod.gen $ fixef[ 1 ], digits=  3 ), 
                                   estimate =   format (mod.gen $ fixef[ 2 ], digits=  3 ), 
                                   df =   as.numeric (test $ basicLRT $ df), 
                                   Pvalue =   as.numeric ( format ( pchisq (Chi2_LRT, df=  1 , lower.tail =  F), digits=  2 ))) 
    
   tab_stat_Ras85DV12 = tab_stat 
    
    
    
    
    
    ##Ras85DV12_2 (TorDER)  
    
   tmp  =   subset (Tab_Ras_PH3_Rev , Male_Line %in%  c ( "TorDER" )) 
    
    
   mod.gen  =   fitme ( log (PH3_positive_cell +  1 )  ~   Diet  +  ( 1   |  Repeat), data =  tmp) 
    shapiro.test ( residuals (mod.gen))     
  ## 
##  Shapiro-Wilk normality test
## 
## data:  residuals(mod.gen)
## W = 0.96675, p-value = 0.1456  
       bptest ( log (PH3_positive_cell +  1 )  ~   Diet  +  ( 1   /  Repeat), data =  tmp)    
  ## 
##  studentized Breusch-Pagan test
## 
## data:  log(PH3_positive_cell + 1) ~ Diet + (1/Repeat)
## BP = 0.77051, df = 1, p-value = 0.3801  
      mod.gen1  =   fitme ( log (PH3_positive_cell +  1 )  ~    1   +  ( 1   |  Repeat), data =  tmp) 
   test  =   anova (mod.gen, mod.gen1)  
   Chi2_LRT  =   2  * (mod.gen $ APHLs[[ "p_v" ]] - mod.gen1 $ APHLs[[ "p_v" ]]) 
    
   tab_stat  =   data.frame ( Variable =   as.character ( paste ( "HS vs HY TorDER" )), 
                                   Rep =   nlevels (Tab_Ras_PH3_Rev $ Repeat), 
                                   chi2_LR =   round ( as.numeric (test $ basicLRT $ chi2_LR),  digits =   2 ), 
                                   intercept =   format (mod.gen $ fixef[ 1 ], digits=  3 ), 
                                   estimate =   format (mod.gen $ fixef[ 2 ], digits=  3 ), 
                                   df =   as.numeric (test $ basicLRT $ df), 
                                   Pvalue =   as.numeric ( format ( pchisq (Chi2_LRT, df=  1 , lower.tail =  F), digits=  2 ))) 
    
   tab_stat_TorDER = tab_stat 
    
    
    
   tab_stat =  rbind (tab_stat_Control, tab_stat_Ras85DV12, tab_stat_TorDER) 
   tab_stat $ padj  =   as.numeric ( format ( p.adjust (tab_stat $ Pvalue,  method =   "BH" ), digits=  2 , scientific = F)) 
   tab_stat $ sig  =   ifelse (tab_stat $ padj  <   0.05   &  tab_stat $ padj  >   0.01 ,  "*" , 
                 ifelse (tab_stat $ padj  <   0.01   &  tab_stat $ padj  >   0.001 ,  "**" , 
                  ifelse (tab_stat $ padj  <   0.001 ,  "***" ,  "" ))) 
    
   tab_stat %>%  
      kable ( col.names =   c ( "Comparison" ,  "Replicates" ,  "Chi2" , "Intercept" , "Estimate" , "df"  , "p-value" , "p-value adjusted" , "Signif." ), row.names =   FALSE )  %>%  
      add_header_above ( c ( "log(PH3_positive_cell+1) ~  Diet + (1 | Repeat)"   =   9 )) %>%  
      kable_styling ( bootstrap_options =   c ( "striped" ,  "hover" ,  "condensed" ),  full_width =  F)    
 
 
 
 
 
log(PH3_positive_cell+1) ~ Diet + (1 | Repeat)
 
 
 
 
 
Comparison
 
 
Replicates
 
 
Chi2
 
 
Intercept
 
 
Estimate
 
 
df
 
 
p-value
 
 
p-value adjusted
 
 
Signif.
 
 
 
 
 
 
HS vs HY Control
 
 
5
 
 
0.00
 
 
1.22
 
 
-0.0117
 
 
1
 
 
0.95
 
 
0.95
 
 
 
 
 
 
HS vs HY Ras85DV12
 
 
5
 
 
1.39
 
 
3.87
 
 
0.0679
 
 
1
 
 
0.24
 
 
0.36
 
 
 
 
 
 
HS vs HY TorDER
 
 
5
 
 
2.56
 
 
3.58
 
 
0.168
 
 
1
 
 
0.11
 
 
0.33
 
 
 
 
 
 
      tab_stat_rev_RasPH3 = tab_stat 
   tab_stat_rev_RasPH3 $ Male_Line =  tab_stat_rev_RasPH3 $ Variable 
    
    
    
    ###Stats HS vs control  
    
   tmpd  =   subset (Tab_Ras_PH3_Rev , Diet %in%  c ( "HS" )) 
    
    
    ##Ras85DV12  
    
   tmp  =   subset (tmpd , Male_Line %in%  c ( "Control" ,  "Ras85DV12" )) 
    
    
    
   mod.gen  =   fitme ((PH3_positive_cell +  1 )  ~   Male_Line  +  ( 1   |  Repeat), data =  tmp) 
    shapiro.test ( residuals (mod.gen))     
  ## 
##  Shapiro-Wilk normality test
## 
## data:  residuals(mod.gen)
## W = 0.96712, p-value = 0.01814  
       bptest ((PH3_positive_cell +  1 )  ~   Male_Line  +  ( 1   /  Repeat), data =  tmp)    
  ## 
##  studentized Breusch-Pagan test
## 
## data:  (PH3_positive_cell + 1) ~ Male_Line + (1/Repeat)
## BP = 10.691, df = 1, p-value = 0.001077  
      mod.gen1  =   fitme ((PH3_positive_cell +  1 )  ~    1   +  ( 1   |  Repeat), data =  tmp) 
   test  =   anova (mod.gen, mod.gen1)  
   Chi2_LRT  =   2  * (mod.gen $ APHLs[[ "p_v" ]] - mod.gen1 $ APHLs[[ "p_v" ]]) 
    
   tab_stat  =   data.frame ( Variable =   as.character ( paste ( "HS Ras85DV12 vs HS Control" )), 
                                   Rep =   nlevels (Tab_Ras_PH3_Rev $ Repeat), 
                                   chi2_LR =   round ( as.numeric (test $ basicLRT $ chi2_LR),  digits =   2 ), 
                                   intercept =   format (mod.gen $ fixef[ 1 ], digits=  3 ), 
                                   estimate =   format (mod.gen $ fixef[ 2 ], digits=  3 ), 
                                   df =   as.numeric (test $ basicLRT $ df), 
                                   Pvalue =   as.numeric ( format ( pchisq (Chi2_LRT, df=  1 , lower.tail =  F), digits=  2 ))) 
    
   tab_stat_HS_Ras85DV12 = tab_stat 
    
    
    
    
    
    ##TorDER  
   tmp  =   subset (tmpd , Male_Line %in%  c ( "Control" ,  "TorDER" )) 
    
    
    
   mod.gen  =   fitme ( log (PH3_positive_cell +  1 )  ~   Male_Line  +  ( 1   |  Repeat), data =  tmp) 
    shapiro.test ( residuals (mod.gen))     
  ## 
##  Shapiro-Wilk normality test
## 
## data:  residuals(mod.gen)
## W = 0.98961, p-value = 0.8224  
       bptest ( log (PH3_positive_cell +  1 )  ~   Male_Line  +  ( 1   /  Repeat), data =  tmp)    
  ## 
##  studentized Breusch-Pagan test
## 
## data:  log(PH3_positive_cell + 1) ~ Male_Line + (1/Repeat)
## BP = 10.017, df = 1, p-value = 0.001551  
      mod.gen1  =   fitme ( log (PH3_positive_cell +  1 )  ~    1   +  ( 1   |  Repeat), data =  tmp) 
   test  =   anova (mod.gen, mod.gen1)  
   Chi2_LRT  =   2  * (mod.gen $ APHLs[[ "p_v" ]] - mod.gen1 $ APHLs[[ "p_v" ]]) 
    
   tab_stat  =   data.frame ( Variable =   as.character ( paste ( "HS TorDER vs HS Control" )), 
                                   Rep =   nlevels (Tab_Ras_PH3_Rev $ Repeat), 
                                   chi2_LR =   round ( as.numeric (test $ basicLRT $ chi2_LR),  digits =   2 ), 
                                   intercept =   format (mod.gen $ fixef[ 1 ], digits=  3 ), 
                                   estimate =   format (mod.gen $ fixef[ 2 ], digits=  3 ), 
                                   df =   as.numeric (test $ basicLRT $ df), 
                                   Pvalue =   as.numeric ( format ( pchisq (Chi2_LRT, df=  1 , lower.tail =  F), digits=  2 ))) 
    
   tab_stat_HS_TorDER = tab_stat 
    #Table  
    
    
   tab_stat =  rbind (tab_stat_HS_Ras85DV12, tab_stat_HS_TorDER) 
   tab_stat $ padj  =   as.numeric ( format ( p.adjust (tab_stat $ Pvalue,  method =   "BH" ), digits=  2 , scientific = F)) 
   tab_stat $ sig  =   ifelse (tab_stat $ padj  <   0.05   &  tab_stat $ padj  >   0.01 ,  "*" , 
                 ifelse (tab_stat $ padj  <   0.01   &  tab_stat $ padj  >   0.001 ,  "**" , 
                  ifelse (tab_stat $ padj  <   0.001 ,  "***" ,  "" ))) 
    
   tab_stat %>%  
      kable ( col.names =   c ( "Comparison" ,  "Replicates" ,  "Chi2" , "Intercept" , "Estimate" , "df"  , "p-value" , "p-value adjusted" , "Signif." ), row.names =   FALSE )  %>%  
      add_header_above ( c ( "log(PH3_positive_cell+1) ~  Genotype + (1 | Repeat)"   =   9 )) %>%  
      kable_styling ( bootstrap_options =   c ( "striped" ,  "hover" ,  "condensed" ),  full_width =  F)    
 
 
 
 
 
log(PH3_positive_cell+1) ~ Genotype + (1 | Repeat)
 
 
 
 
 
Comparison
 
 
Replicates
 
 
Chi2
 
 
Intercept
 
 
Estimate
 
 
df
 
 
p-value
 
 
p-value adjusted
 
 
Signif.
 
 
 
 
 
 
HS Ras85DV12 vs HS Control
 
 
5
 
 
138.73
 
 
6.28
 
 
45.3
 
 
1
 
 
0
 
 
0
 
 
***
 
 
 
 
HS TorDER vs HS Control
 
 
5
 
 
71.82
 
 
1.23
 
 
2.46
 
 
1
 
 
0
 
 
0
 
 
***
 
 
 
 
      tab_stat_rev_RasPH3_HS = tab_stat 
   tab_stat_rev_RasPH3_HS $ Male_Line =  tab_stat_rev_RasPH3_HS $ Variable 
    
    
    
    
    ###Stats HY vs control  
    
   tmpd  =   subset (Tab_Ras_PH3_Rev , Diet %in%  c ( "HY" )) 
    
    
    ##Ras85DV12  
    
   tmp  =   subset (tmpd , Male_Line %in%  c ( "Control" ,  "Ras85DV12" )) 
    
    
    
   mod.gen  =   fitme ((PH3_positive_cell +  1 )  ~   Male_Line  +  ( 1   |  Repeat), data =  tmp) 
    shapiro.test ( residuals (mod.gen))     
  ## 
##  Shapiro-Wilk normality test
## 
## data:  residuals(mod.gen)
## W = 0.96149, p-value = 0.009738  
       bptest ((PH3_positive_cell +  1 )  ~   Male_Line  +  ( 1   /  Repeat), data =  tmp)    
  ## 
##  studentized Breusch-Pagan test
## 
## data:  (PH3_positive_cell + 1) ~ Male_Line + (1/Repeat)
## BP = 13.223, df = 1, p-value = 0.0002765  
      mod.gen1  =   fitme ((PH3_positive_cell +  1 )  ~    1   +  ( 1   |  Repeat), data =  tmp) 
   test  =   anova (mod.gen, mod.gen1)  
   Chi2_LRT  =   2  * (mod.gen $ APHLs[[ "p_v" ]] - mod.gen1 $ APHLs[[ "p_v" ]]) 
    
   tab_stat  =   data.frame ( Variable =   as.character ( paste ( "HY Ras85DV12 vs HY Control" )), 
                                   Rep =   nlevels (Tab_Ras_PH3_Rev $ Repeat), 
                                   chi2_LR =   round ( as.numeric (test $ basicLRT $ chi2_LR),  digits =   2 ), 
                                   intercept =   format (mod.gen $ fixef[ 1 ], digits=  3 ), 
                                   estimate =   format (mod.gen $ fixef[ 2 ], digits=  3 ), 
                                   df =   as.numeric (test $ basicLRT $ df), 
                                   Pvalue =   as.numeric ( format ( pchisq (Chi2_LRT, df=  1 , lower.tail =  F), digits=  2 ))) 
    
   tab_stat_HY_Ras85DV12 = tab_stat 
    
    
    
    
    ##TorDER  
   tmp  =   subset (tmpd , Male_Line %in%  c ( "Control" ,  "TorDER" )) 
    
    
    
   mod.gen  =   fitme ( log (PH3_positive_cell +  1 )  ~   Male_Line  +  ( 1   |  Repeat), data =  tmp) 
    shapiro.test ( residuals (mod.gen))     
  ## 
##  Shapiro-Wilk normality test
## 
## data:  residuals(mod.gen)
## W = 0.9602, p-value = 0.02268  
       bptest ( log (PH3_positive_cell +  1 )  ~   Male_Line  +  ( 1   /  Repeat), data =  tmp)    
  ## 
##  studentized Breusch-Pagan test
## 
## data:  log(PH3_positive_cell + 1) ~ Male_Line + (1/Repeat)
## BP = 13.229, df = 1, p-value = 0.0002757  
      mod.gen1  =   fitme ( log (PH3_positive_cell +  1 )  ~    1   +  ( 1   |  Repeat), data =  tmp) 
   test  =   anova (mod.gen, mod.gen1)  
   Chi2_LRT  =   2  * (mod.gen $ APHLs[[ "p_v" ]] - mod.gen1 $ APHLs[[ "p_v" ]]) 
    
   tab_stat  =   data.frame ( Variable =   as.character ( paste ( "HY TorDER vs HY Control" )), 
                                   Rep =   nlevels (Tab_Ras_PH3_Rev $ Repeat), 
                                   chi2_LR =   round ( as.numeric (test $ basicLRT $ chi2_LR),  digits =   2 ), 
                                   intercept =   format (mod.gen $ fixef[ 1 ], digits=  3 ), 
                                   estimate =   format (mod.gen $ fixef[ 2 ], digits=  3 ), 
                                   df =   as.numeric (test $ basicLRT $ df), 
                                   Pvalue =   as.numeric ( format ( pchisq (Chi2_LRT, df=  1 , lower.tail =  F), digits=  2 ))) 
    
   tab_stat_HY_TorDER = tab_stat 
    #Table  
    
    
   tab_stat =  rbind (tab_stat_HY_Ras85DV12, tab_stat_HY_TorDER) 
   tab_stat $ padj  =   as.numeric ( format ( p.adjust (tab_stat $ Pvalue,  method =   "BH" ), digits=  2 , scientific = F)) 
   tab_stat $ sig  =   ifelse (tab_stat $ padj  <   0.05   &  tab_stat $ padj  >   0.01 ,  "*" , 
                 ifelse (tab_stat $ padj  <   0.01   &  tab_stat $ padj  >   0.001 ,  "**" , 
                  ifelse (tab_stat $ padj  <   0.001 ,  "***" ,  "" ))) 
    
   tab_stat %>%  
      kable ( col.names =   c ( "Comparison" ,  "Replicates" ,  "Chi2" , "Intercept" , "Estimate" , "df"  , "p-value" , "p-value adjusted" , "Signif." ), row.names =   FALSE )  %>%  
      add_header_above ( c ( "log(PH3_positive_cell+1) ~  Genotype + (1 | Repeat)"   =   9 )) %>%  
      kable_styling ( bootstrap_options =   c ( "striped" ,  "hover" ,  "condensed" ),  full_width =  F)    
 
 
 
 
 
log(PH3_positive_cell+1) ~ Genotype + (1 | Repeat)
 
 
 
 
 
Comparison
 
 
Replicates
 
 
Chi2
 
 
Intercept
 
 
Estimate
 
 
df
 
 
p-value
 
 
p-value adjusted
 
 
Signif.
 
 
 
 
 
 
HY Ras85DV12 vs HY Control
 
 
5
 
 
141.81
 
 
5.89
 
 
47.7
 
 
1
 
 
0
 
 
0
 
 
***
 
 
 
 
HY TorDER vs HY Control
 
 
5
 
 
102.66
 
 
1.2
 
 
2.67
 
 
1
 
 
0
 
 
0
 
 
***
 
 
 
 
      tab_stat_rev_RasPH3_HY = tab_stat 
   tab_stat_rev_RasPH3_HY $ Male_Line =  tab_stat_rev_RasPH3_HY $ Variable 
    
    #Samplesize  
    
   Sample_size =  
    Tab_Ras_PH3_Rev %>%  
      group_by (Male_Line,Diet) %>%  
      summarise ( Sample_size=  n ()) 
    
    
    
    ### Plot  
   Tab_Ras_PH3_Rev $ Male_Line  =   factor (Tab_Ras_PH3_Rev $ Male_Line,  labels =   c ( expression ( italic ( paste ( "Control" ))),  expression ( italic ( paste ( "Ra" ,s ^ {V12}, sep=  "" ))),  expression ( italic ( paste ( "Tor-DER" ))))) 
    
    
   Sample_size $ Male_Line  =   factor (Sample_size $ Male_Line,  labels =   c ( expression ( italic ( paste ( "Control" ))),  expression ( italic ( paste ( "Ra" ,s ^ {V12}, sep=  "" ))),  expression ( italic ( paste ( "Tor-DER" ))))) 
    
   tab_stat_rev_RasPH3 $ Male_Line  =   factor (tab_stat_rev_RasPH3 $ Male_Line,  labels =   c ( expression ( italic ( paste ( "Control" ))),  expression ( italic ( paste ( "Ra" ,s ^ {V12}, sep=  "" ))),  expression ( italic ( paste ( "Tor-DER" ))))) 
    
   tab_stat_rev_RasPH3_HS $ Male_Line  =   factor (tab_stat_rev_RasPH3_HS $ Male_Line,  labels =   c ( expression ( italic ( paste ( "Ra" ,s ^ {V12}, sep=  "" ))),  expression ( italic ( paste ( "Tor-DER" ))))) 
    
   tab_stat_rev_RasPH3_HY $ Male_Line  =   factor (tab_stat_rev_RasPH3_HY $ Male_Line,  labels =   c ( expression ( italic ( paste ( "Ra" ,s ^ {V12}, sep=  "" ))),  expression ( italic ( paste ( "Tor-DER" ))))) 
    
    
    #Annotation in the plot  
    
    
   ann_textHS  <-  data.frame ( Male_Line =   "Control" ,  anot =   "Vs Ctrl HS" ) 
   ann_textHS $ Male_Line  =   factor (ann_textHS $ Male_Line,  labels =   c ( expression ( italic ( paste ( "Control" ))))) 
    
   ann_textHY  <-  data.frame ( Male_Line =   "Control" ,  anot =   "Vs Ctrl HY" ) 
   ann_textHY $ Male_Line  =   factor (ann_textHY $ Male_Line,  labels =   c ( expression ( italic ( paste ( "Control" ))))) 
    
   ann_text  <-  data.frame ( Male_Line =   "Ras85DV12" ,  anot =   "HS vs HY" ) 
   ann_text $ Male_Line  =   factor (ann_text $ Male_Line,  labels =   c ( expression ( italic ( paste ( "Ra" ,s ^ {V12}, sep=  "" ))))) 
    
    
    #Plot  
   Limits  =   c ( "HS" , "HY" ) 
    
   Plot_Fig5D =  
      ggplot (Tab_Ras_PH3_Rev,  aes ( x =  Diet,  y =  PH3_positive_cell)) +   
        geom_violin ( aes ( fill =  Diet),  draw_quantiles =   c ( 0.25 ,  0.5 ,  0.75 ),  colour =   "black" ,  size =   0.2 , adjust =   0.8 )  +  
      geom_dotplot (  colour =   "black" ,  fill =   "white" ,  binaxis =   "y" ,  stackdir =   "center" ,  binwidth =   3 )  +   
      facet_grid (.  ~  Male_Line, labeller= label_parsed) +  
      geom_text ( data =  Sample_size,  mapping =   aes ( x =  Diet,  y =   -  10 ,  label =   paste ( "(" ,Sample_size, ")" , sep=  "" )), size=  3 ) +  
      geom_signif ( data =  tab_stat_rev_RasPH3,  aes ( xmin =   1 ,  xmax =   2 ,  annotations =   formatC ( paste ( "p=" ,Pvalue),  digits =   2 ),  y_position =   105 ),  textsize =   2.5 ,  vjust =   -  0.2 ,  manual =   TRUE ) +  
      scale_fill_manual ( limits=  c ( "HS" , "HY" ), 
                        values=  c ( "#FFB4B4" , "#C3E6FC" )) +  
      scale_x_discrete ( "" , 
                       limits= Limits, 
                       labels=  c ( "HS" , "HY" )) +  
      scale_y_continuous ( expression ( paste ( "pH3"   ^   "+" ,  " cells" )), 
                         limits=  c ( -  15 , 175 ), 
                         breaks=  seq ( 0 , 110 , by=  20 )) +  
      stat_summary ( fun =  mean,  geom =   "point" ,  size =   3 ,  shape =   18 ,  colour =   "black" ,  aes ( group =  Repeat))  +  
      stat_summary ( fun =  mean,  geom =   "point" ,  size =   2 ,  shape =   18 ,  aes ( group =  Repeat,  colour =  Repeat))  +  
      scale_color_manual ( values =  palette_mean)  +  
      theme ( panel.grid.major.y =   element_line ( colour =   grey ( 0.45 ),  linetype =   "dashed" ,  size =   0.2 ), 
            panel.background =   element_blank (), 
            axis.title.x =   element_text ( size= Smallfont, colour=  "black" ), 
            axis.title.y =   element_text ( size= Smallfont, colour=  "black" ,  margin =   margin ( t =   0 ,  r =   0 ,  b =   0 ,  l =   0 ) ),  
            axis.line.x =   element_line ( colour=  "black" , size=  0.75 ), 
            axis.line.y =   element_line ( colour=  "black" , size=  0.75 ), 
            axis.ticks.x =   element_line ( size =   0.75 ), 
            axis.ticks.y =   element_line ( size =   0.75 ), 
            axis.text.x =   element_text ( size= Smallfont, colour=  "black" ), 
            axis.text.y =   element_text ( size= Smallfont, colour=  "black" ), 
            plot.margin =   unit (Margin,  "cm" ), 
            legend.direction =   "vertical" ,  
            legend.box =   "horizontal" , 
            legend.position =   "none" , 
            legend.key.height =   unit ( 0.4 ,  "cm" ), 
            legend.key.width=   unit ( 0.6 ,  "cm" ), 
            legend.title =   element_text ( face=  "italic" , size= Smallfont),  
            legend.key =   element_rect ( colour =   'white' ,  fill =   "white" ,  linetype=  'dashed' ), 
            legend.text =   element_text ( size= SuperSmallfont), 
            legend.background =   element_rect ( fill=  NA ), 
            strip.text =   element_text ( size = Smallfont -2 ,  colour =   "black" , face=  "italic" ,  margin =   margin ( t =   2 ,  r =   1 ,  b =   2 ,  l =   1 )), 
            strip.background =   element_rect ( fill=  NA ,  colour=  "black" ), 
            strip.placement=  "outside" ) 
    
   Plot_Fig5D    
   
 
 
  5.1.5  Figure 5E 
 
 Enterocyte specific over expression (MyoTS) of UAS-upd3-OE and UAS-spi-SEC elicit increased proliferation, strongly only on HY diet, and weakly on HS with UAS-upd3-OE. P-values on top of the chart refer to comparisons with the control, p-values at the bottom refer to comparison between HS and HY for each sample. Flies were 9 days old when dissected.  Complete statistical annotation on image can be fund in the manuscript’s figures.  
 
      tab_Myo_PH3_Rev  =   
     d[[ "5E" ]] %>%  
      mutate_at ( vars ( ends_with ( ".L" )), ~ . /  1000 ) %>%  
      mutate_at ( vars ( !  starts_with ( "Total" )),as.factor) %>%  
     dplyr ::  rename ( PH3_positive_cell= Total.PH3, 
                    Diet= Treatment, 
                    Male_Line= Male.Line) 
    
    
    ###Stats HS vs HY  
    
    
    
    ##Control  
   tmp  =   subset (tab_Myo_PH3_Rev, Male_Line %in%  c ( "Control" )) 
    
    
   mod.gen  =   fitme ( log (PH3_positive_cell +  1 )  ~   Diet  +  ( 1   |  Repeat), data =  tmp) 
    
    shapiro.test ( residuals (mod.gen))     
  ## 
##  Shapiro-Wilk normality test
## 
## data:  residuals(mod.gen)
## W = 0.94719, p-value = 0.04711  
       bptest ( log (PH3_positive_cell +  1 )  ~   Diet  +  ( 1   /  Repeat), data =  tmp)    
  ## 
##  studentized Breusch-Pagan test
## 
## data:  log(PH3_positive_cell + 1) ~ Diet + (1/Repeat)
## BP = 2.986, df = 1, p-value = 0.08399  
      mod.gen1  =   fitme ( log (PH3_positive_cell +  1 )  ~    1   +  ( 1   |  Repeat), data =  tmp) 
    
   test  =   anova (mod.gen, mod.gen1)  
    
   Chi2_LRT  =   2  * (mod.gen $ APHLs[[ "p_v" ]] - mod.gen1 $ APHLs[[ "p_v" ]]) 
    
   tab_stat  =   data.frame ( Comparison =   as.character ( paste ( "Control HS vs HY" )), 
                          
                                   Rep =   nlevels (tab_Myo_PH3_Rev $ Repeat), 
                          
                                   chi2_LR =   round ( as.numeric (test $ basicLRT $ chi2_LR),  digits =   2 ), 
                          
                                   intercept =   format (mod.gen $ fixef[ 1 ], digits=  3 ), 
                          
                                   estimate =   format (mod.gen $ fixef[ 2 ], digits=  3 ), 
                          
                                   df =   as.numeric (test $ basicLRT $ df), 
                          
                                   Pvalue =   as.numeric ( format ( pchisq (Chi2_LRT, df=  1 , lower.tail =  F), digits=  2 ))) 
    
   tab_stat_Control = tab_stat 
    
   tab_stat_Control $ Male_Line  =   "Control"  
    
    
    
    
    ##spi-sec  
    
    
   tmp  =   subset (tab_Myo_PH3_Rev , Male_Line %in%  c ( "spi-SEC" )) 
    
    
   mod.gen  =   fitme ( log (PH3_positive_cell +  1 )  ~   Diet  +  ( 1   |  Repeat), data =  tmp) 
    
    shapiro.test ( residuals (mod.gen))     
  ## 
##  Shapiro-Wilk normality test
## 
## data:  residuals(mod.gen)
## W = 0.98552, p-value = 0.8119  
       bptest ( log (PH3_positive_cell +  1 )  ~   Diet  +  ( 1   /  Repeat), data =  tmp)    
  ## 
##  studentized Breusch-Pagan test
## 
## data:  log(PH3_positive_cell + 1) ~ Diet + (1/Repeat)
## BP = 0.079412, df = 1, p-value = 0.7781  
      mod.gen1  =   fitme ( log (PH3_positive_cell +  1 )  ~    1   +  ( 1   |  Repeat), data =  tmp) 
    
   test  =   anova (mod.gen, mod.gen1)  
    
   Chi2_LRT  =   2  * (mod.gen $ APHLs[[ "p_v" ]] - mod.gen1 $ APHLs[[ "p_v" ]]) 
    
   tab_stat  =   data.frame ( Comparison =   as.character ( paste ( "spi-SEC HS vs HY" )), 
                          
                                   Rep =   nlevels (tmp $ Repeat), 
                          
                                   chi2_LR =   round ( as.numeric (test $ basicLRT $ chi2_LR),  digits =   2 ), 
                          
                                   intercept =   format (mod.gen $ fixef[ 1 ], digits=  3 ), 
                          
                                   estimate =   format (mod.gen $ fixef[ 2 ], digits=  3 ), 
                          
                                   df =   as.numeric (test $ basicLRT $ df), 
                          
                                   Pvalue =   as.numeric ( format ( pchisq (Chi2_LRT, df=  1 , lower.tail =  F), digits=  2 ))) 
    
   tab_stat_spi_sec = tab_stat 
    
   tab_stat_spi_sec $ Male_Line  =   "spi-SEC"  
    
    ##upd3-OE  
    
   tmp  =   subset (tab_Myo_PH3_Rev , Male_Line %in%  c ( "upd3-OE" )) 
    
    
   mod.gen  =   fitme ( log (PH3_positive_cell +  1 )  ~   Diet  +  ( 1   |  Repeat), data =  tmp) 
    
    shapiro.test ( residuals (mod.gen))     
  ## 
##  Shapiro-Wilk normality test
## 
## data:  residuals(mod.gen)
## W = 0.98739, p-value = 0.8533  
       bptest ( log (PH3_positive_cell +  1 )  ~   Diet  +  ( 1   /  Repeat), data =  tmp)    
  ## 
##  studentized Breusch-Pagan test
## 
## data:  log(PH3_positive_cell + 1) ~ Diet + (1/Repeat)
## BP = 1.9695, df = 1, p-value = 0.1605  
      mod.gen1  =   fitme ( log (PH3_positive_cell +  1 )  ~    1   +  ( 1   |  Repeat), data =  tmp)
[truncated: 259,253 more chars]
