# Supplementary figures and images for: Multiscale analysis reveals that diet-dependent midgut plasticity emerges from alterations in both stem cell niche coupling and enterocyte size (part 2 of 2)
Source: eLife. 2021 Sep 23;10:e64125. doi: 10.7554/eLife.64125 (PMC8528489; doi:10.7554/eLife.64125)

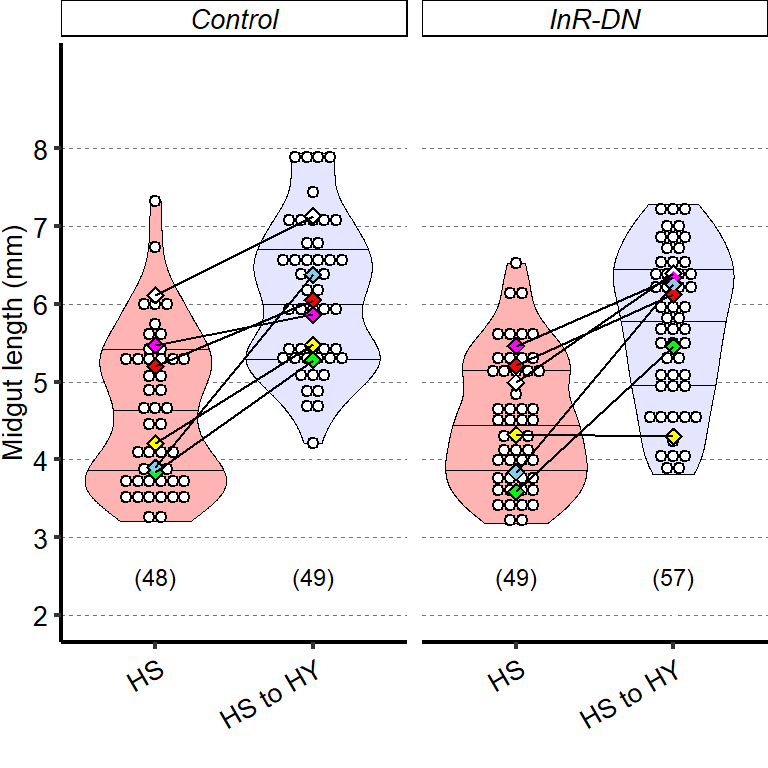

Supplement: Supplementary file 2. [file elife-64125-supp2.zip › Bonfini_script_GutPlasticity_diet_files/figure-html/Figure 6G-1.png]

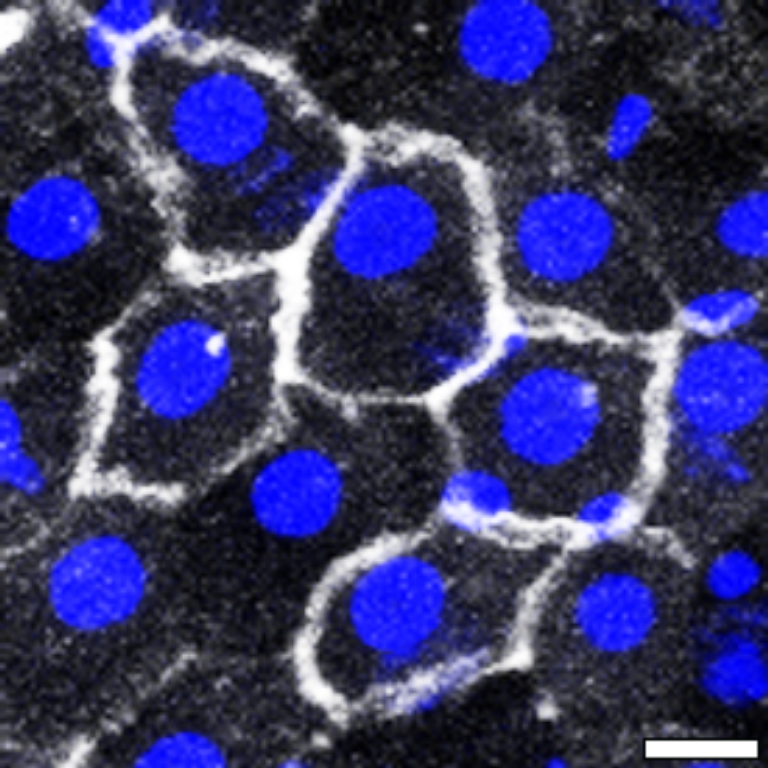

Supplement: Supplementary file 2. [file elife-64125-supp2.zip › Bonfini_script_GutPlasticity_diet_files/figure-html/Figure 6H-1.png]

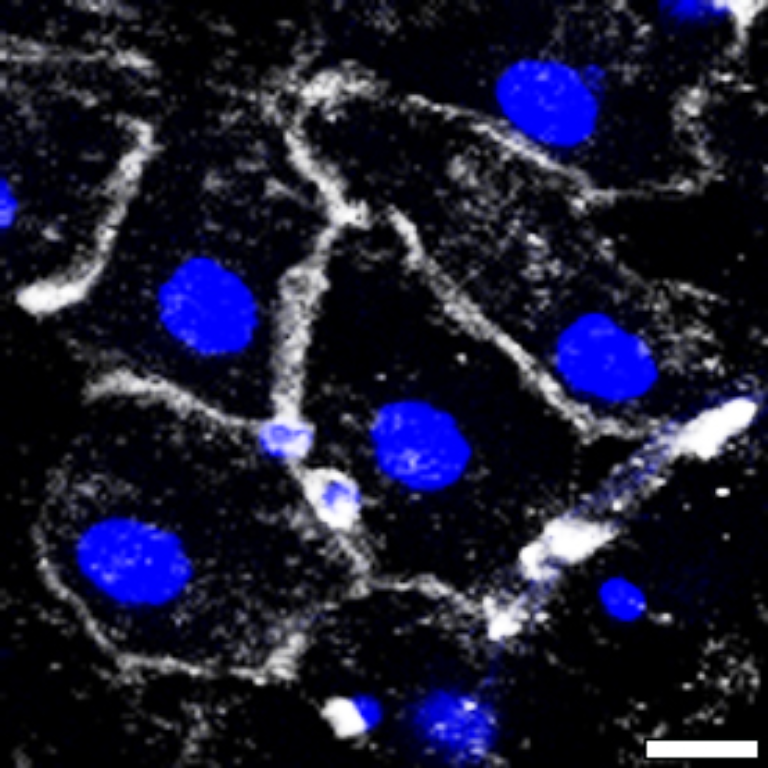

Supplement: Supplementary file 2. [file elife-64125-supp2.zip › Bonfini_script_GutPlasticity_diet_files/figure-html/Figure 6I-1.png]

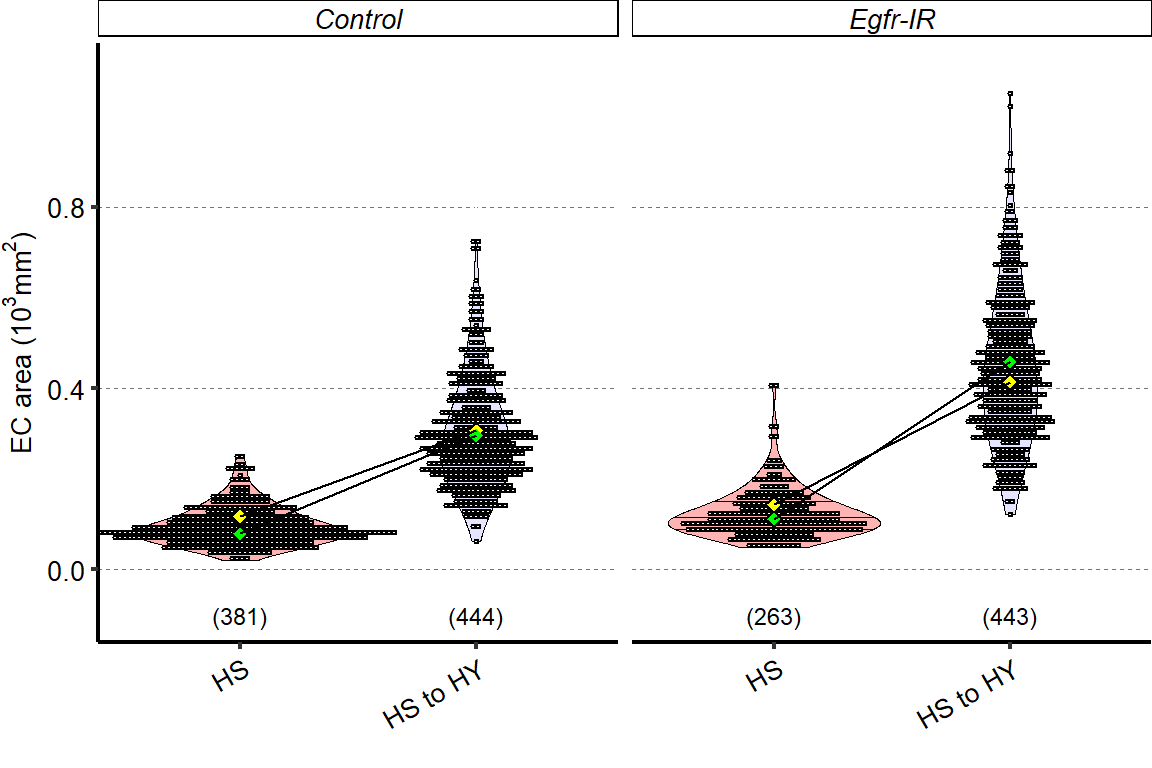

Supplement: Supplementary file 2. [file elife-64125-supp2.zip › Bonfini_script_GutPlasticity_diet_files/figure-html/Figure 6J-1.png]

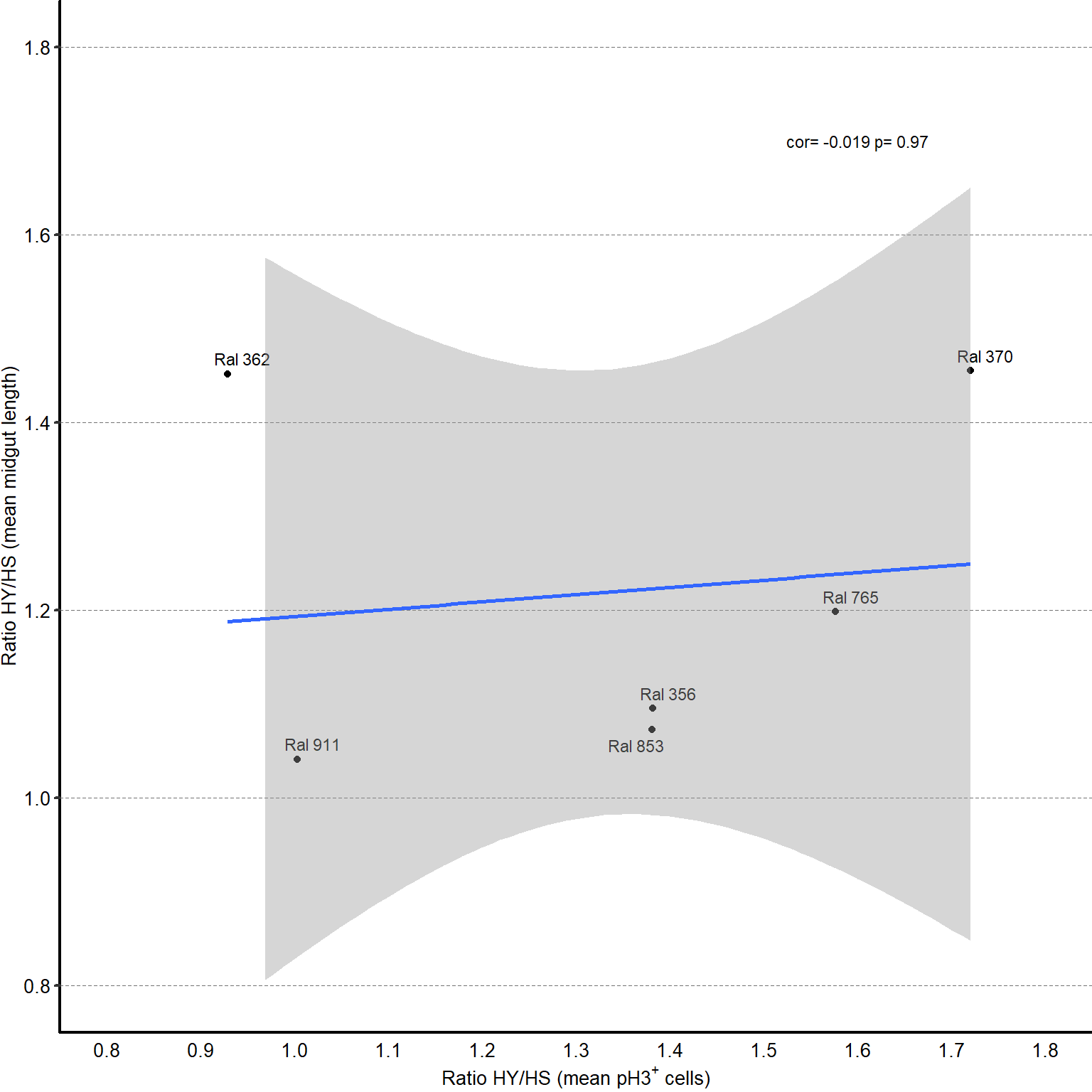

Supplement: Supplementary file 2. [file elife-64125-supp2.zip › Bonfini_script_GutPlasticity_diet_files/figure-html/Figure 6S1A-1.png]

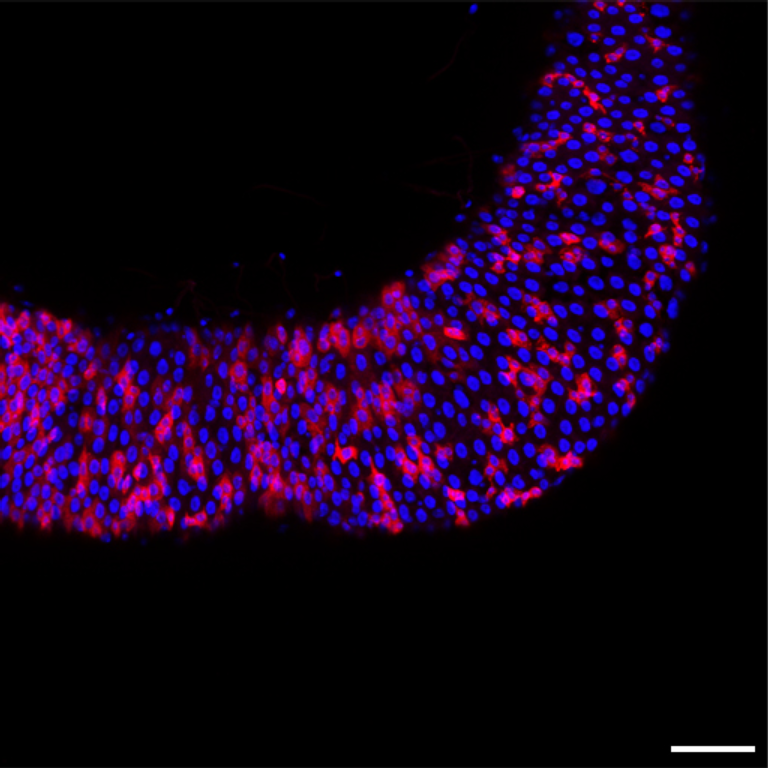

Supplement: Supplementary file 2. [file elife-64125-supp2.zip › Bonfini_script_GutPlasticity_diet_files/figure-html/Figure 6S1B-1.png]

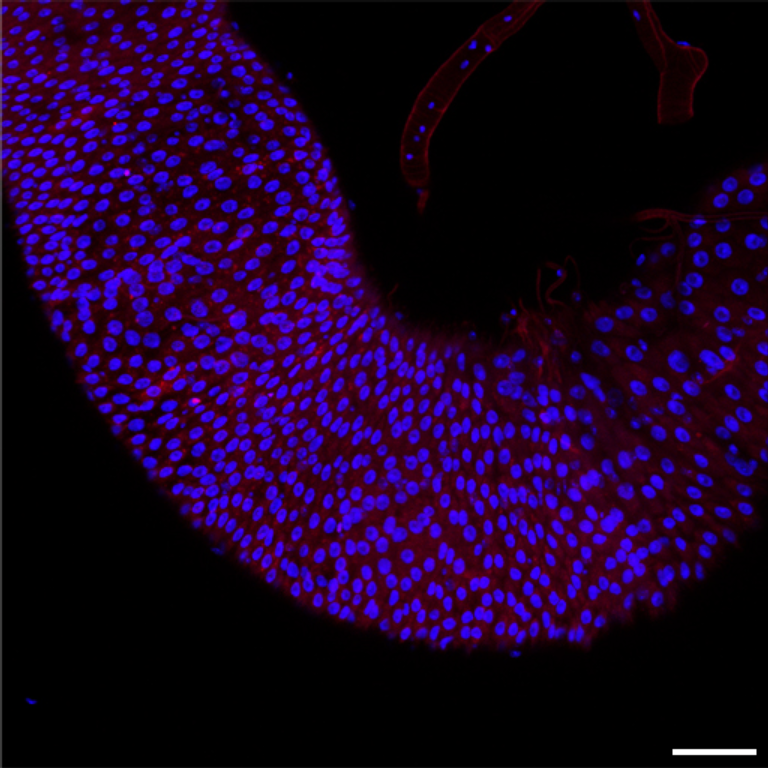

Supplement: Supplementary file 2. [file elife-64125-supp2.zip › Bonfini_script_GutPlasticity_diet_files/figure-html/Figure 6S1C-1.png]

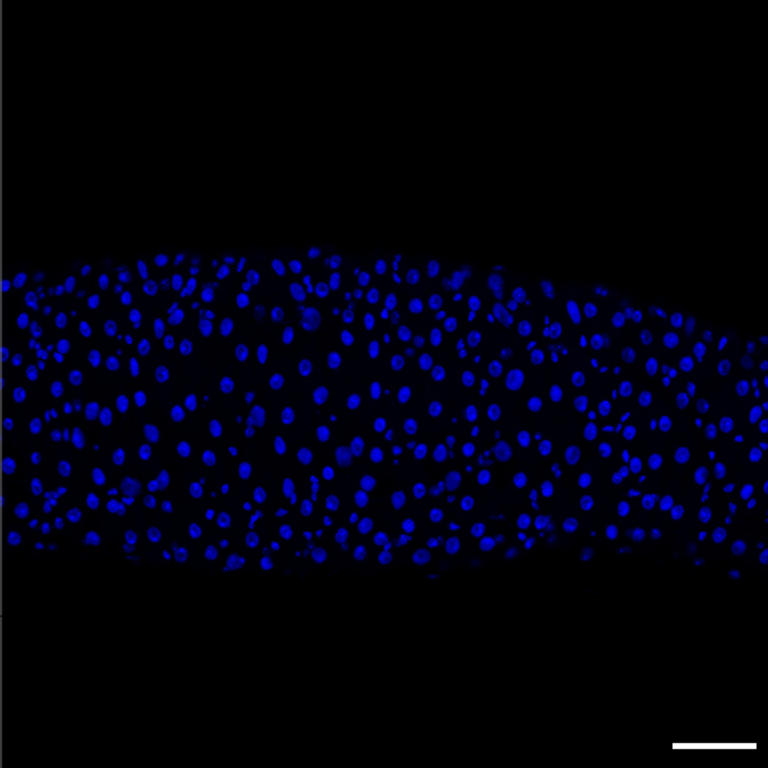

Supplement: Supplementary file 2. [file elife-64125-supp2.zip › Bonfini_script_GutPlasticity_diet_files/figure-html/Figure 6S1D-1.png]

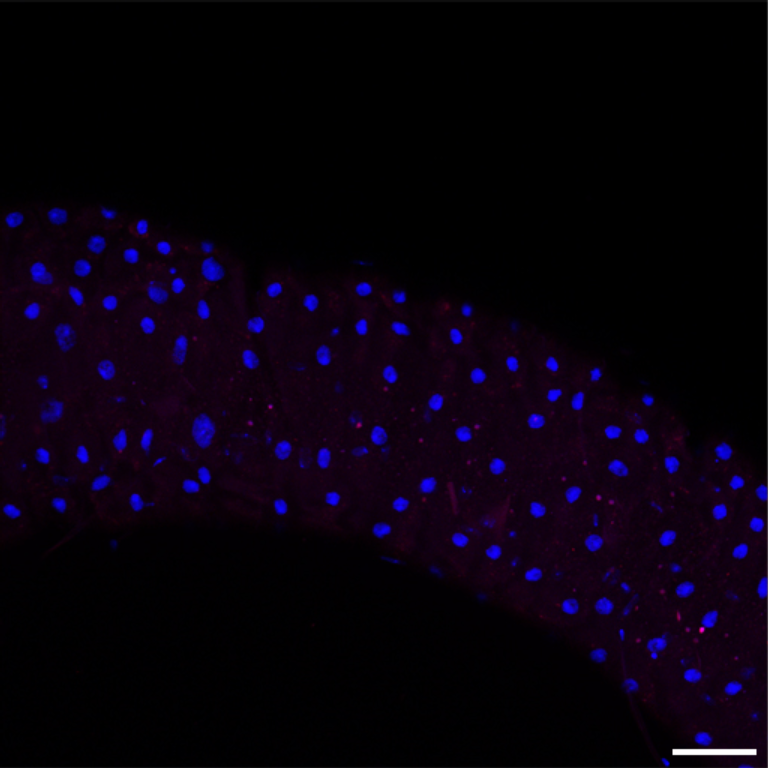

Supplement: Supplementary file 2. [file elife-64125-supp2.zip › Bonfini_script_GutPlasticity_diet_files/figure-html/Figure 6S1E-1.png]

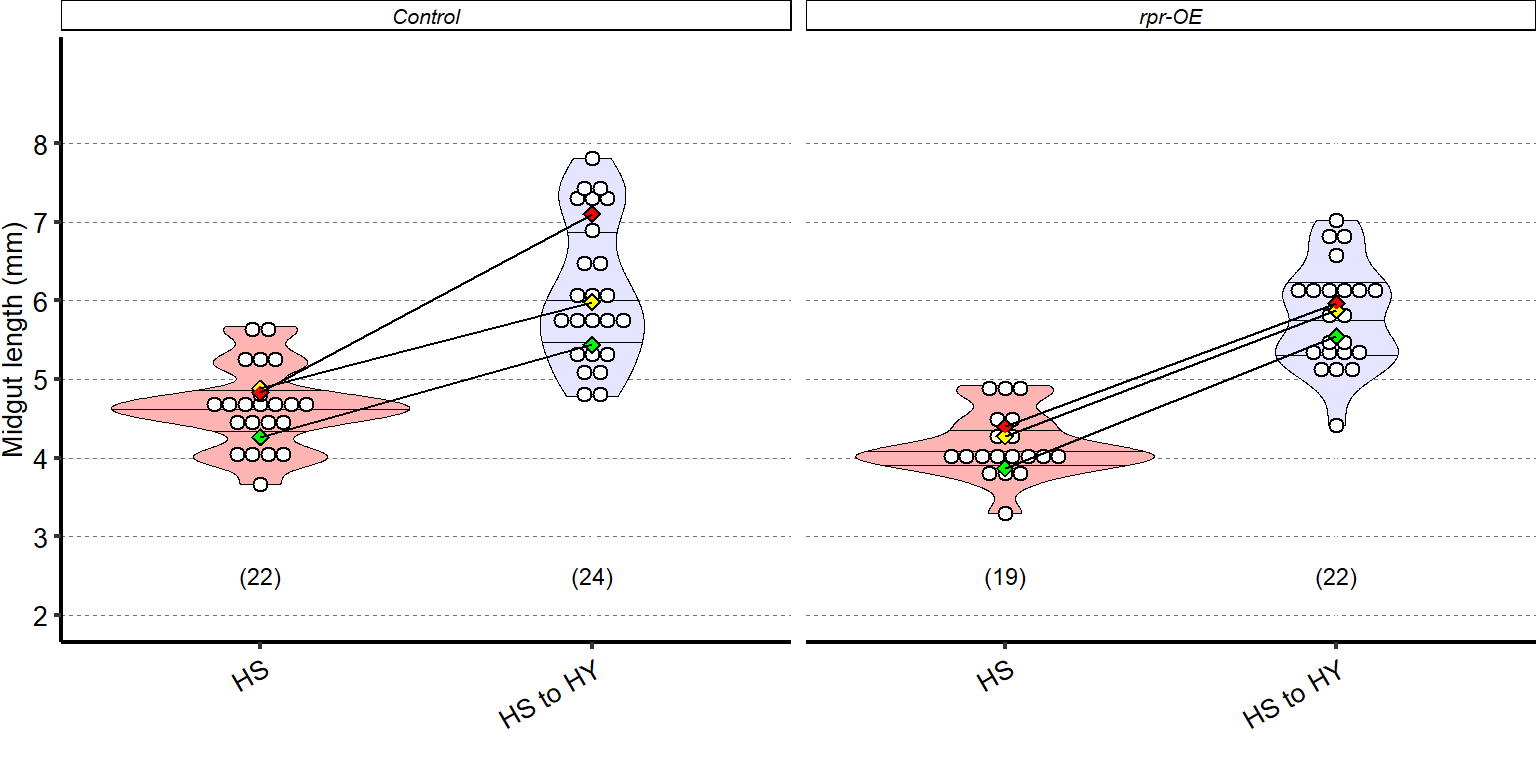

Supplement: Supplementary file 2. [file elife-64125-supp2.zip › Bonfini_script_GutPlasticity_diet_files/figure-html/Figure 6S1F-1.png]

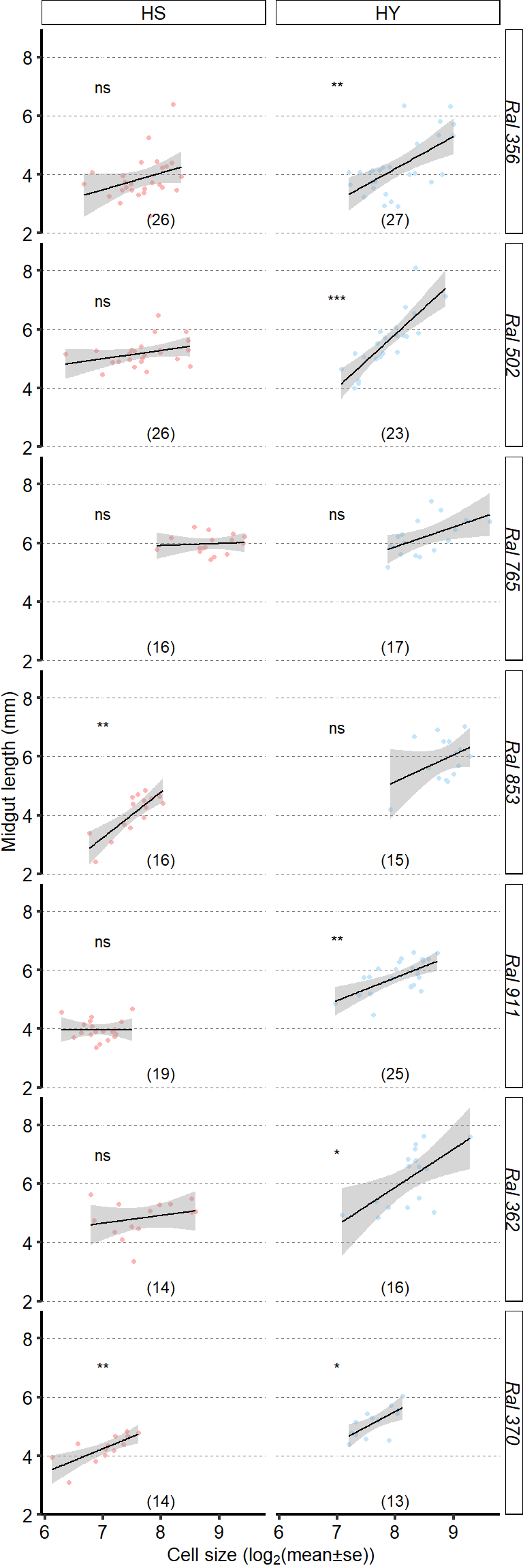

Supplement: Supplementary file 2. [file elife-64125-supp2.zip › Bonfini_script_GutPlasticity_diet_files/figure-html/Figure 7A-1.png]

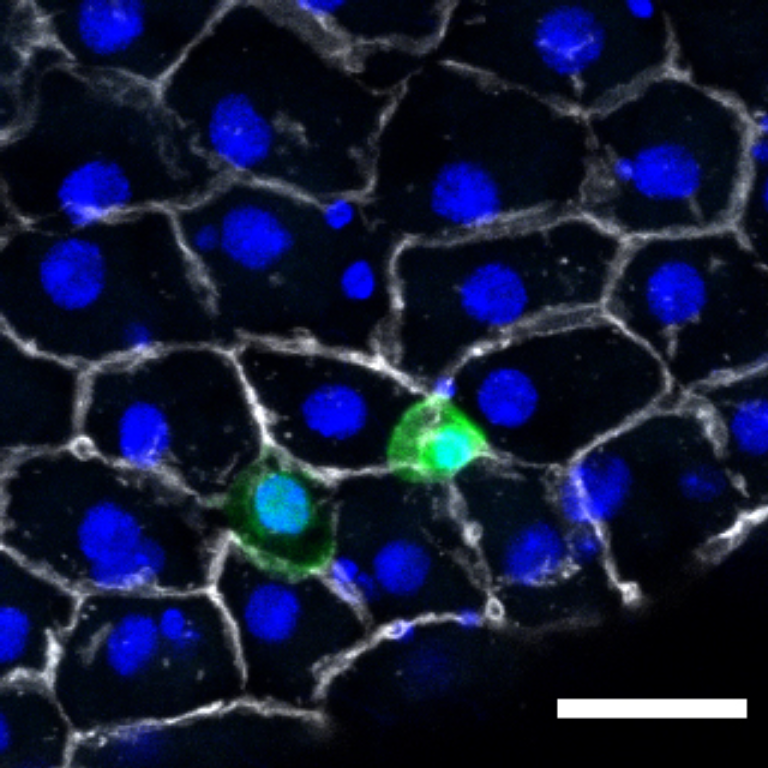

Supplement: Supplementary file 2. [file elife-64125-supp2.zip › Bonfini_script_GutPlasticity_diet_files/figure-html/Figure 7B-1.png]

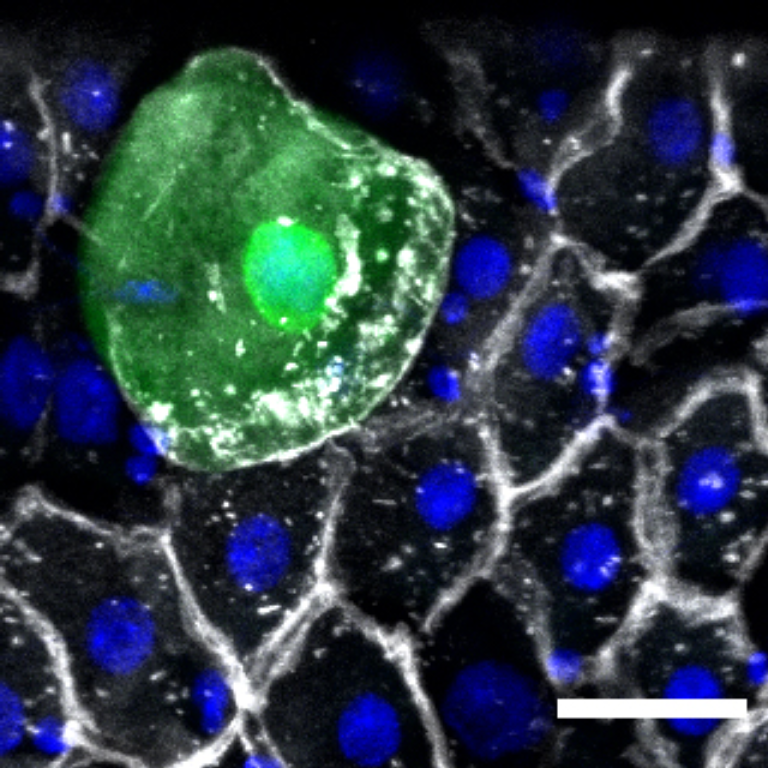

Supplement: Supplementary file 2. [file elife-64125-supp2.zip › Bonfini_script_GutPlasticity_diet_files/figure-html/Figure 7C-1.png]

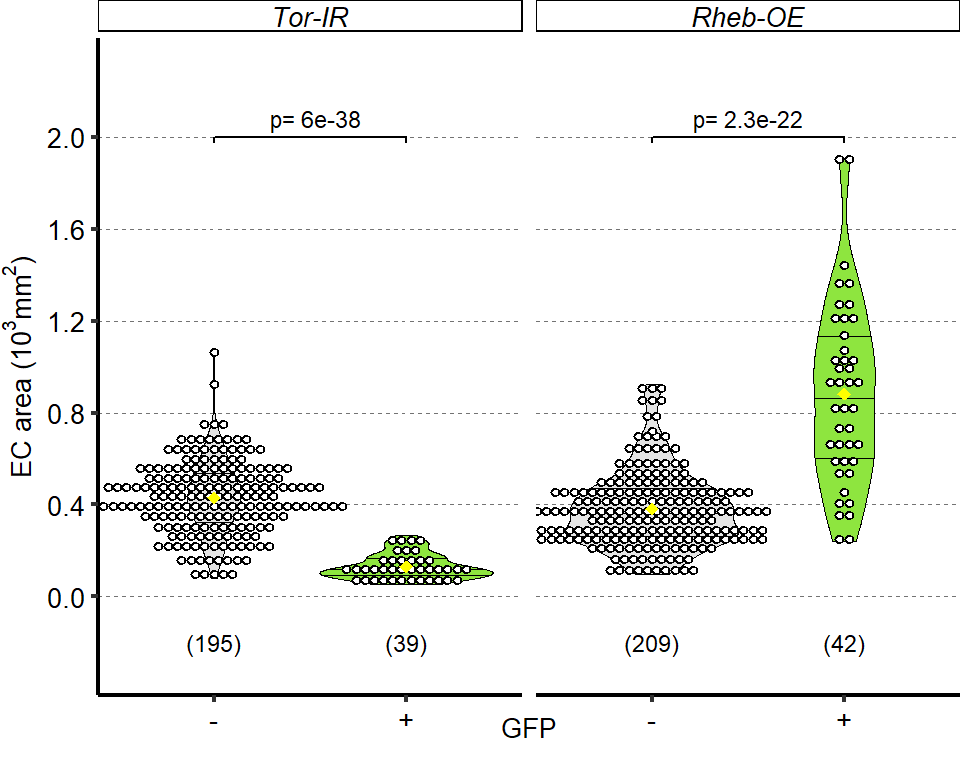

Supplement: Supplementary file 2. [file elife-64125-supp2.zip › Bonfini_script_GutPlasticity_diet_files/figure-html/Figure 7D-1.png]

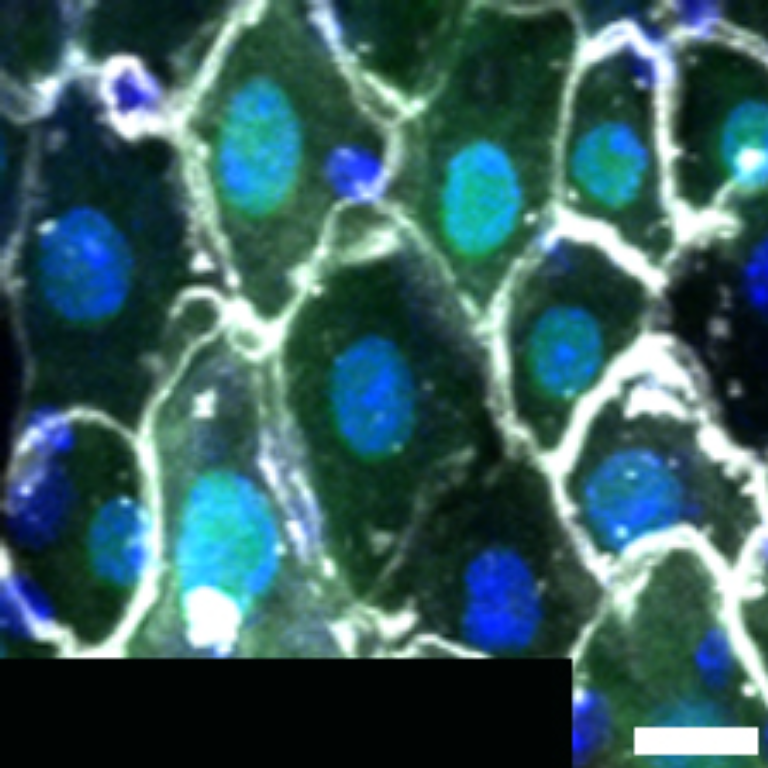

Supplement: Supplementary file 2. [file elife-64125-supp2.zip › Bonfini_script_GutPlasticity_diet_files/figure-html/Figure 7E-1.png]

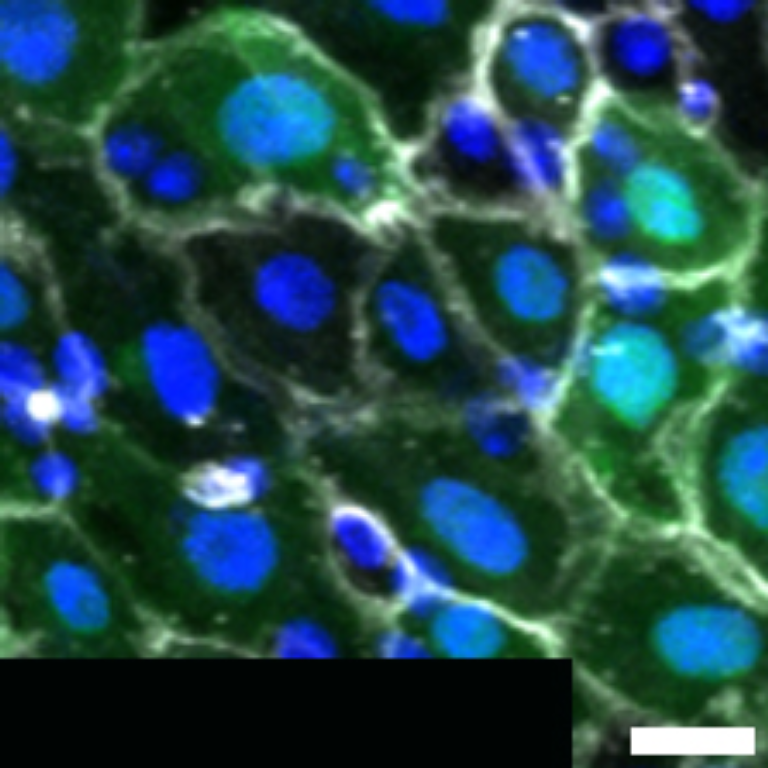

Supplement: Supplementary file 2. [file elife-64125-supp2.zip › Bonfini_script_GutPlasticity_diet_files/figure-html/Figure 7F-1.png]

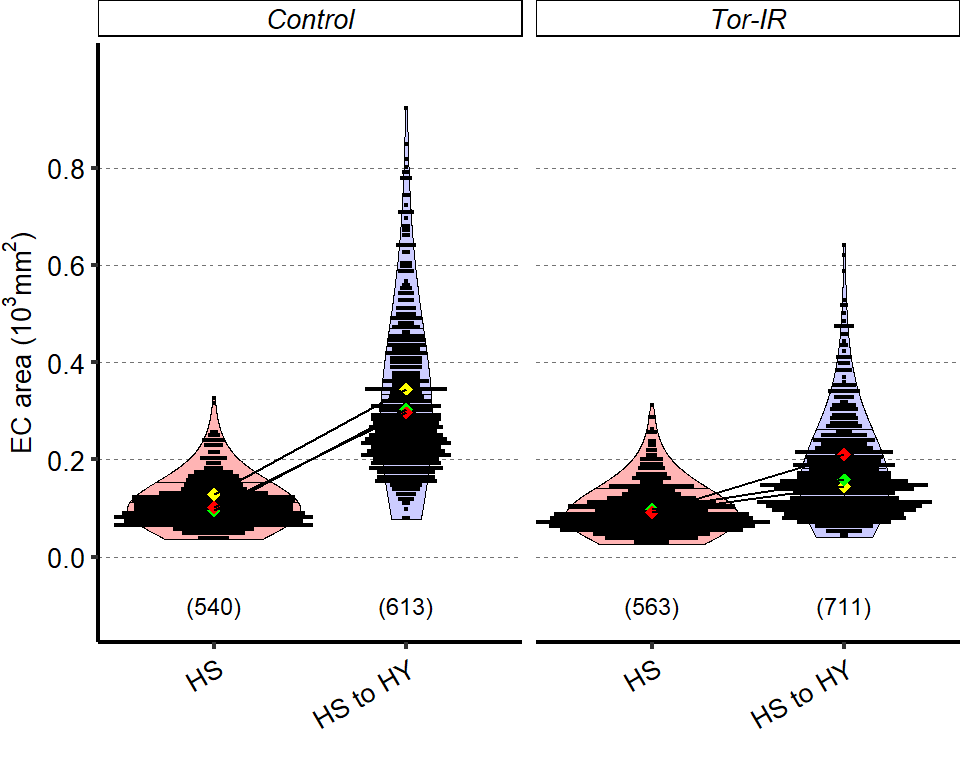

Supplement: Supplementary file 2. [file elife-64125-supp2.zip › Bonfini_script_GutPlasticity_diet_files/figure-html/Figure 7G-1.png]

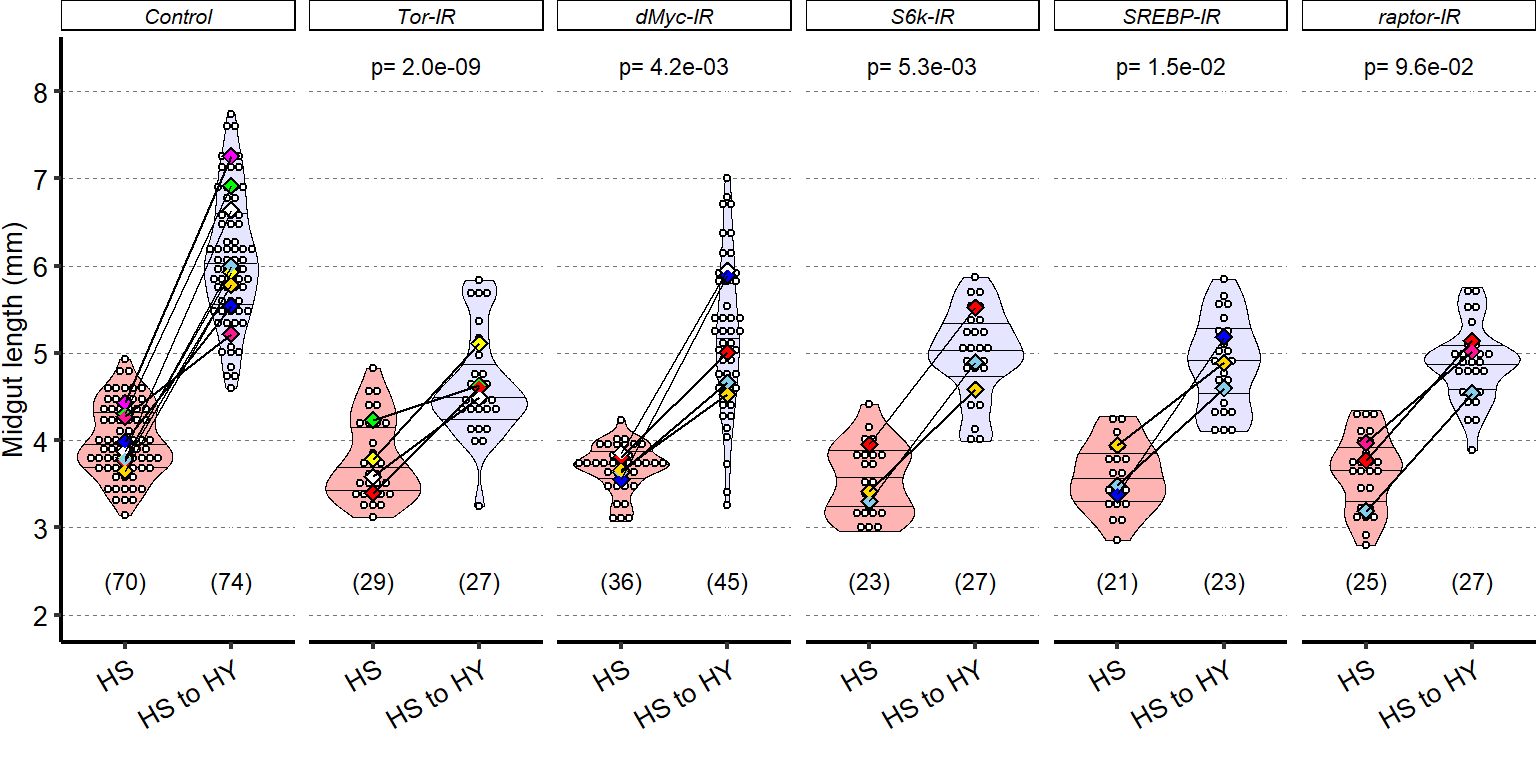

Supplement: Supplementary file 2. [file elife-64125-supp2.zip › Bonfini_script_GutPlasticity_diet_files/figure-html/Figure 7H-1.png]

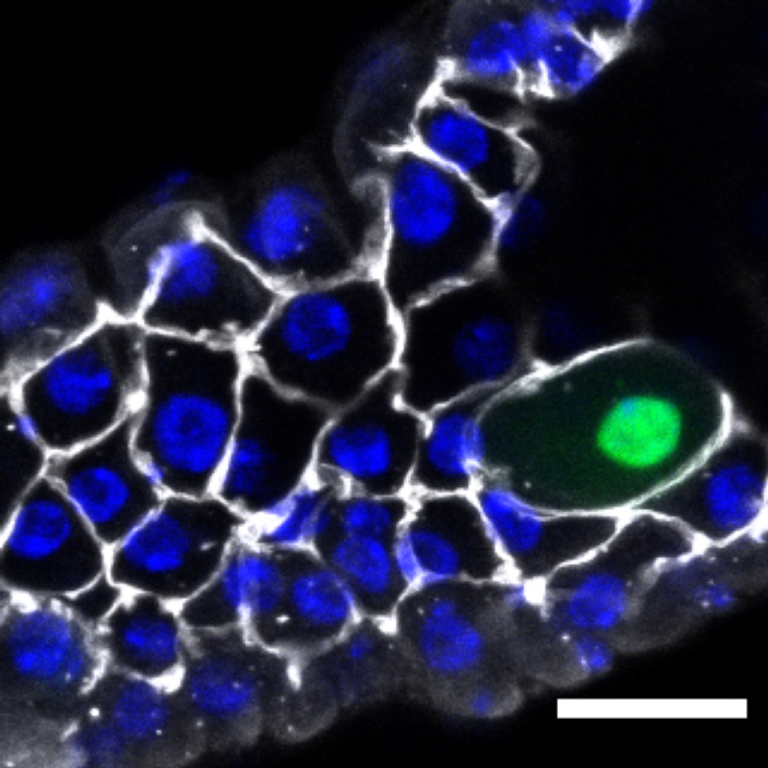

Supplement: Supplementary file 2. [file elife-64125-supp2.zip › Bonfini_script_GutPlasticity_diet_files/figure-html/Figure 7I-1.png]

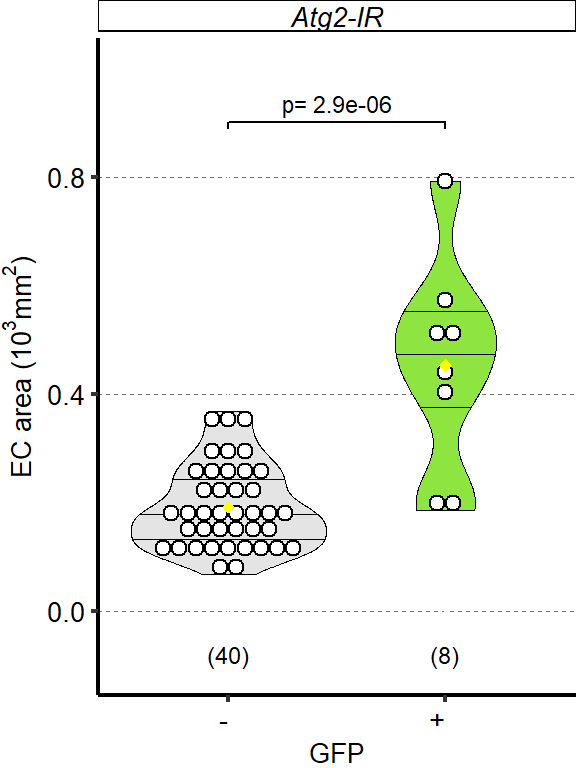

Supplement: Supplementary file 2. [file elife-64125-supp2.zip › Bonfini_script_GutPlasticity_diet_files/figure-html/Figure 7J-1.png]

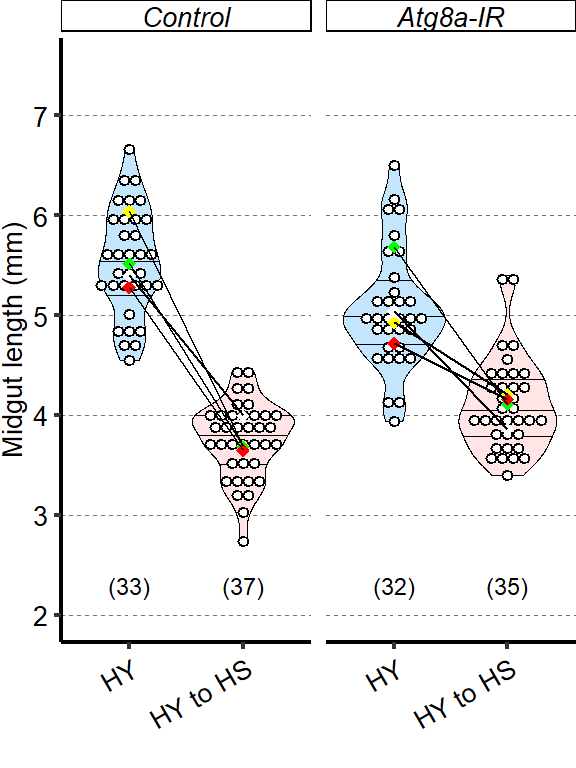

Supplement: Supplementary file 2. [file elife-64125-supp2.zip › Bonfini_script_GutPlasticity_diet_files/figure-html/Figure 7K-1.png]

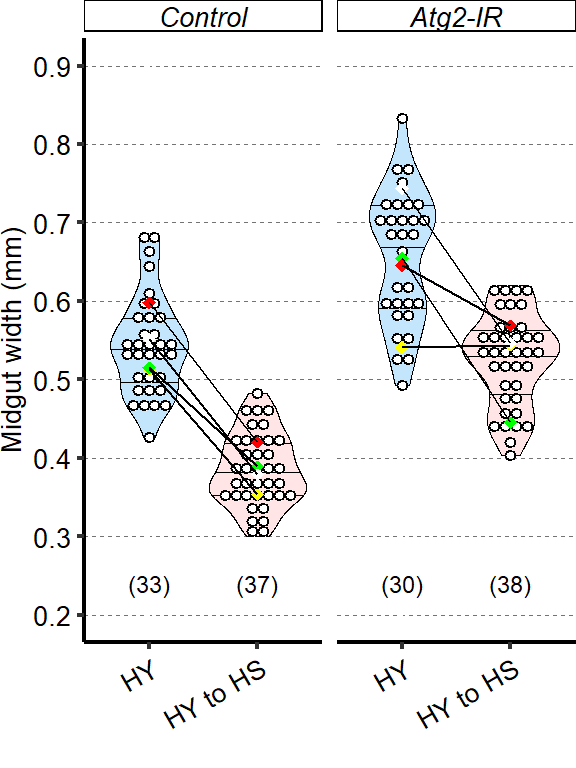

Supplement: Supplementary file 2. [file elife-64125-supp2.zip › Bonfini_script_GutPlasticity_diet_files/figure-html/Figure 7L-1.png]

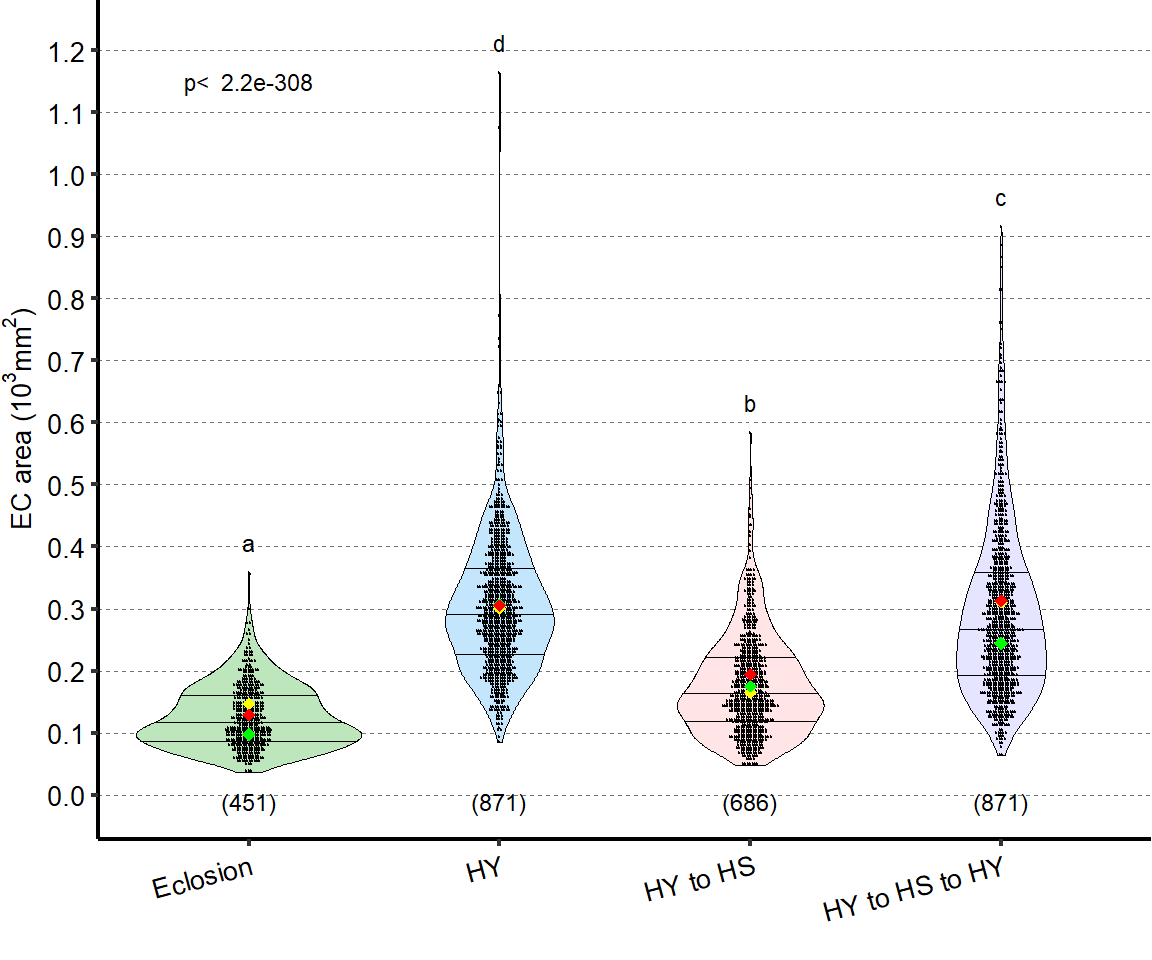

Supplement: Supplementary file 2. [file elife-64125-supp2.zip › Bonfini_script_GutPlasticity_diet_files/figure-html/Figure 7S1A-1.png]

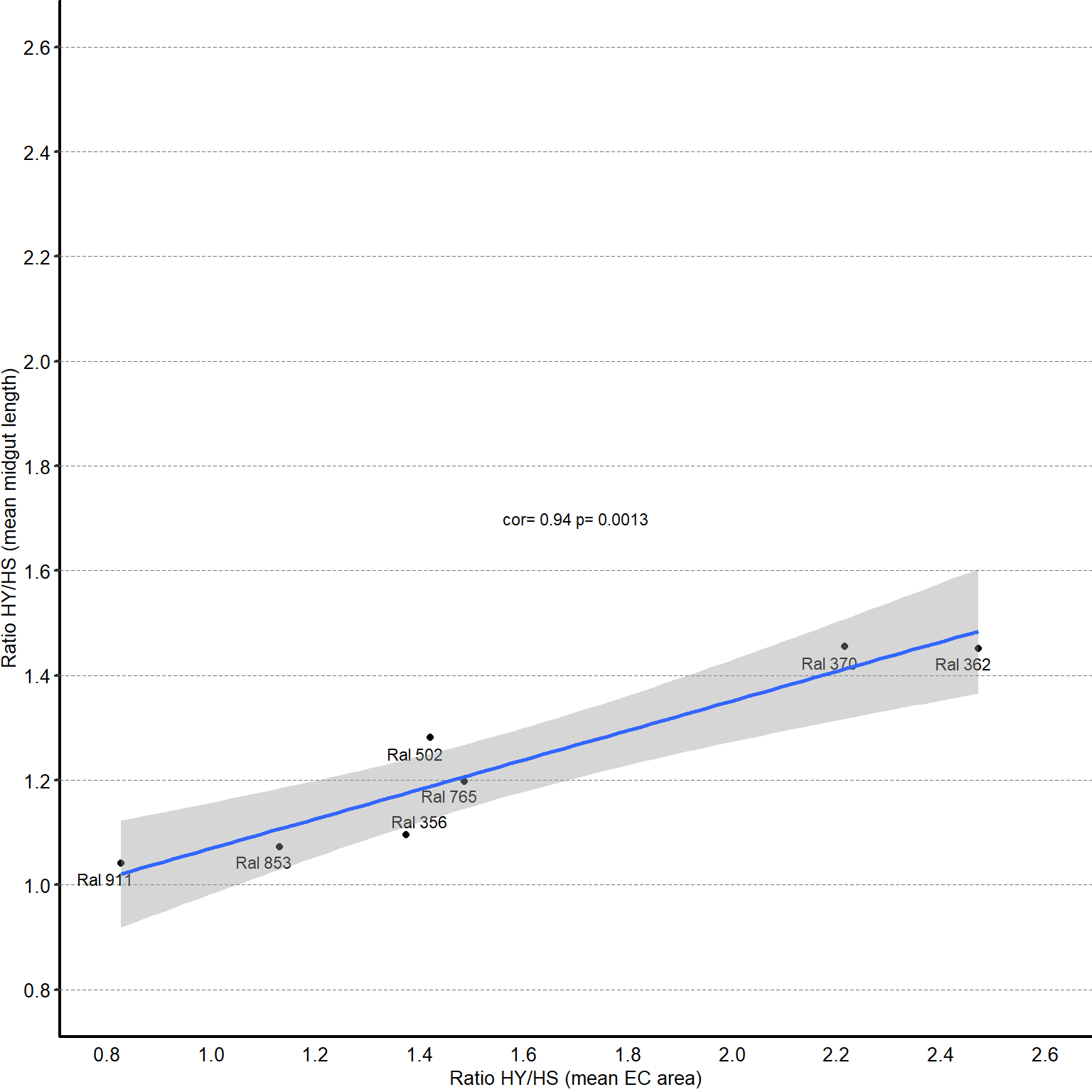

Supplement: Supplementary file 2. [file elife-64125-supp2.zip › Bonfini_script_GutPlasticity_diet_files/figure-html/Figure 7S1B-1.png]

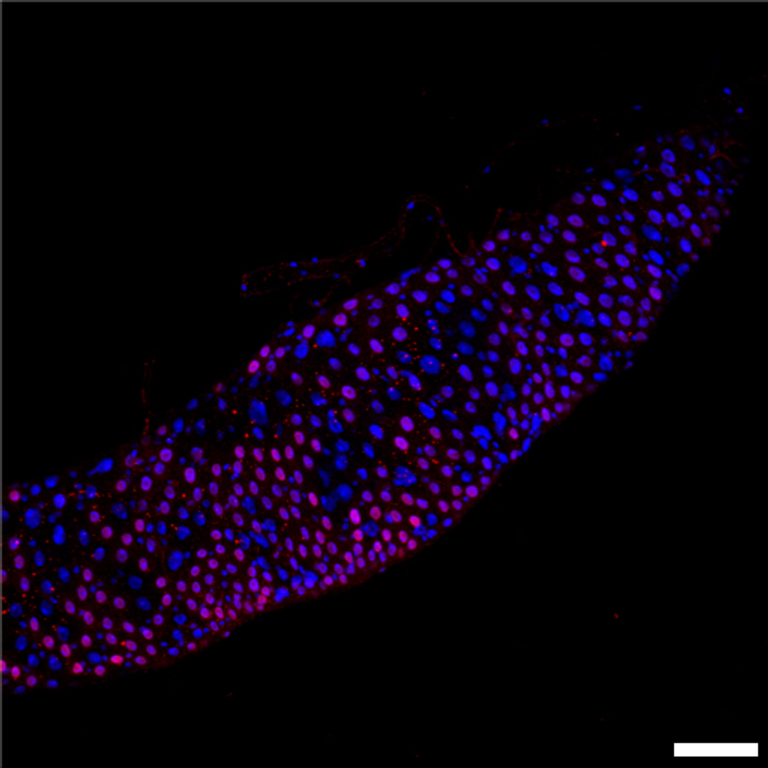

Supplement: Supplementary file 2. [file elife-64125-supp2.zip › Bonfini_script_GutPlasticity_diet_files/figure-html/Figure 7S2A-1.png]

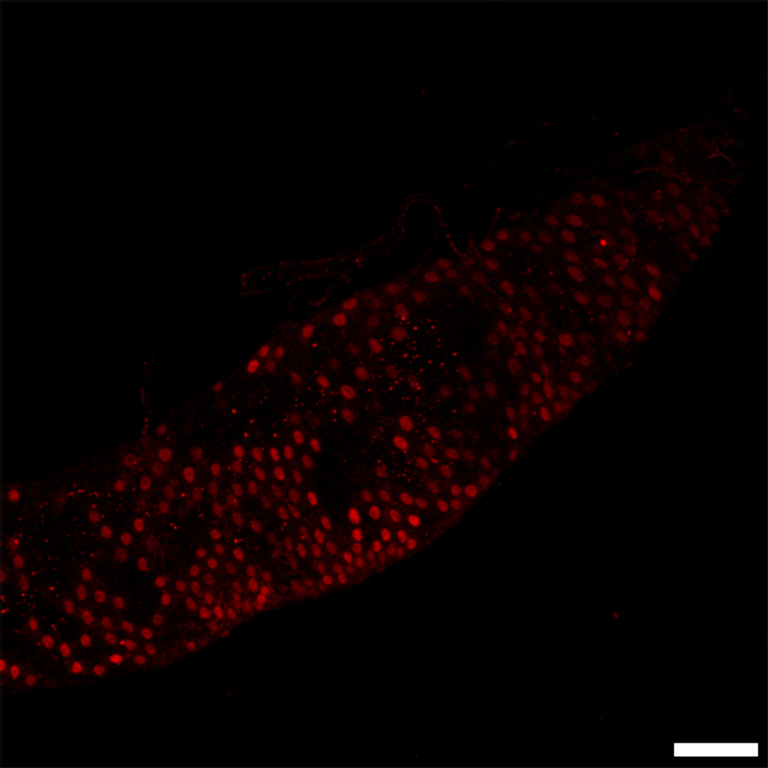

Supplement: Supplementary file 2. [file elife-64125-supp2.zip › Bonfini_script_GutPlasticity_diet_files/figure-html/Figure 7S2A1-1.png]

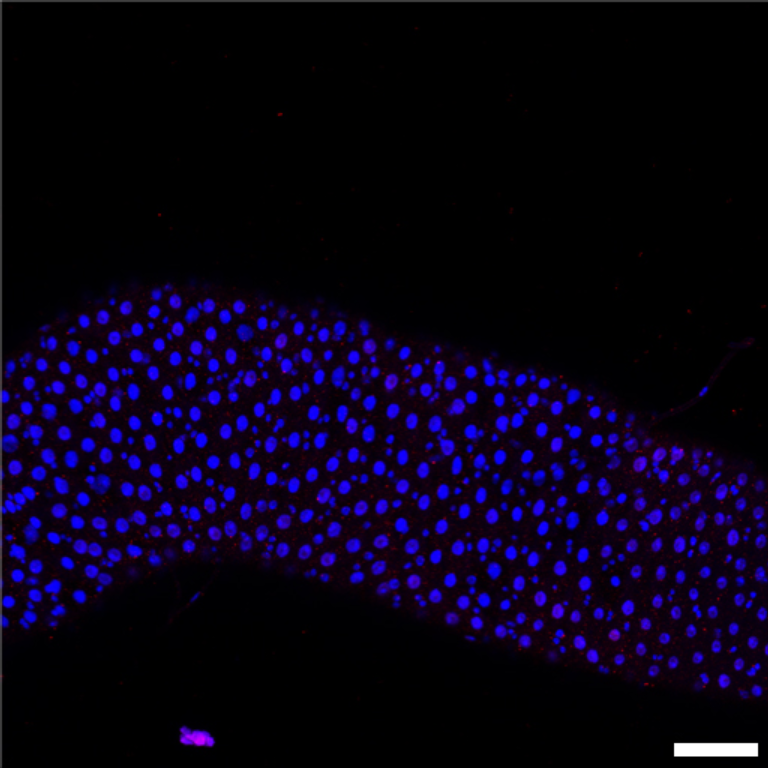

Supplement: Supplementary file 2. [file elife-64125-supp2.zip › Bonfini_script_GutPlasticity_diet_files/figure-html/Figure 7S2B-1.png]

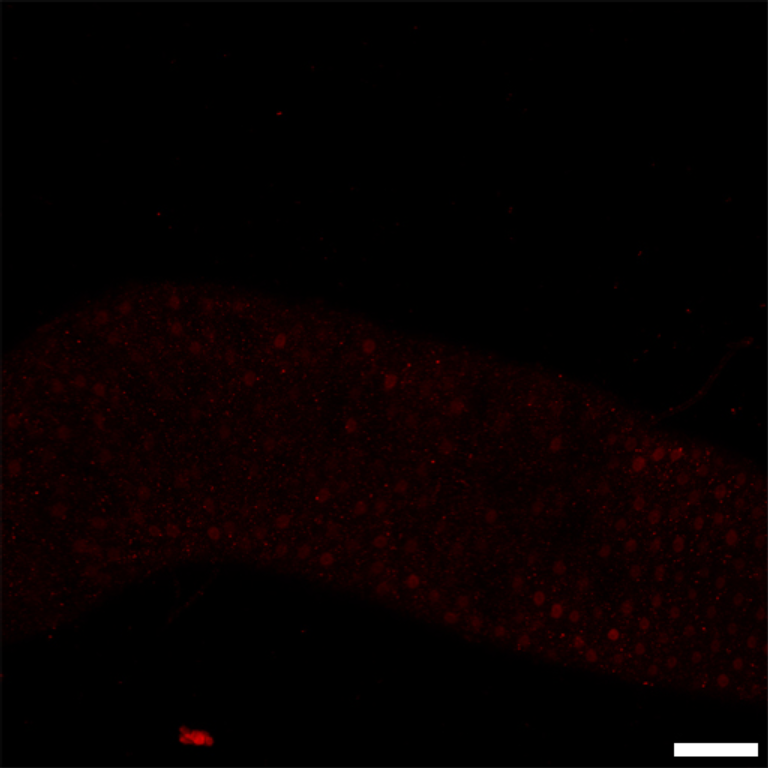

Supplement: Supplementary file 2. [file elife-64125-supp2.zip › Bonfini_script_GutPlasticity_diet_files/figure-html/Figure 7S2B1-1.png]

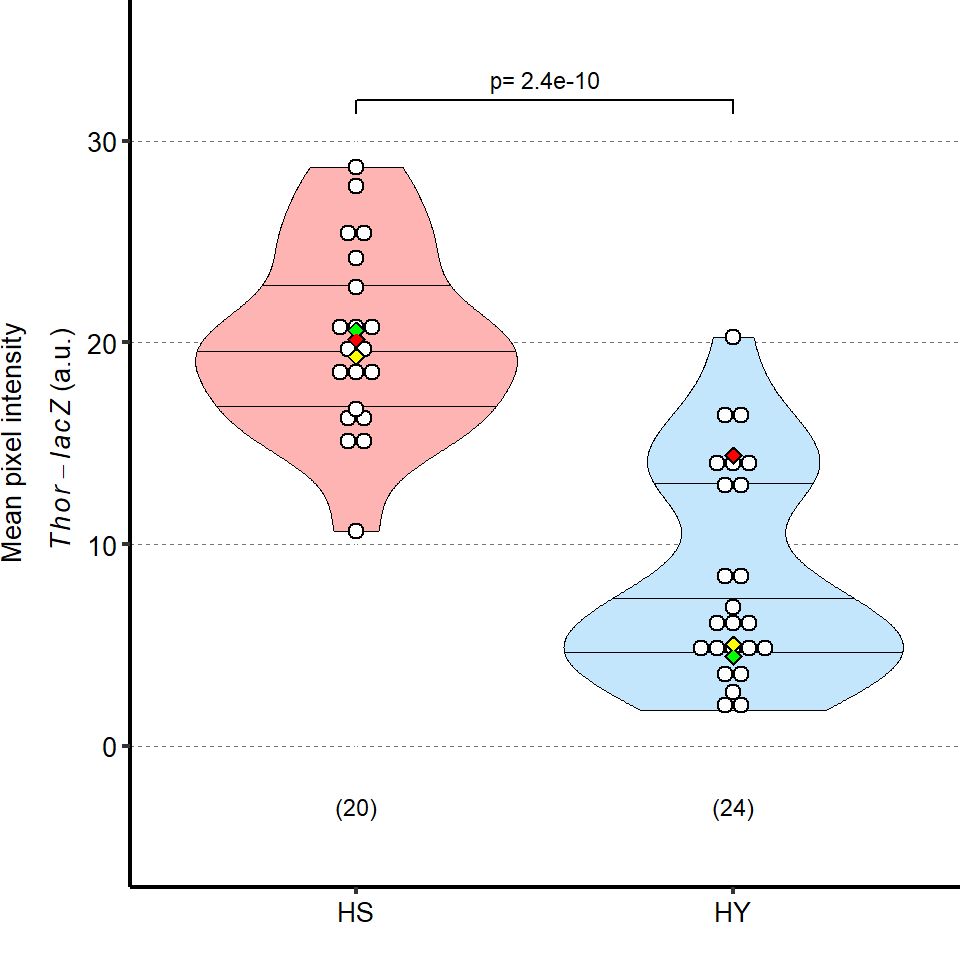

Supplement: Supplementary file 2. [file elife-64125-supp2.zip › Bonfini_script_GutPlasticity_diet_files/figure-html/Figure 7S2C-1.png]

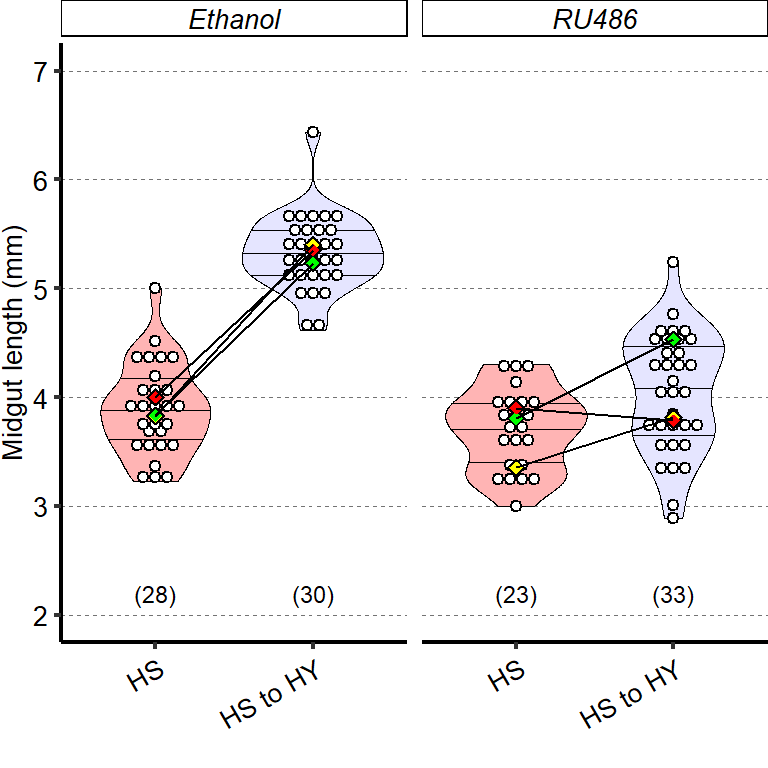

Supplement: Supplementary file 2. [file elife-64125-supp2.zip › Bonfini_script_GutPlasticity_diet_files/figure-html/Figure 7S2D-1.png]

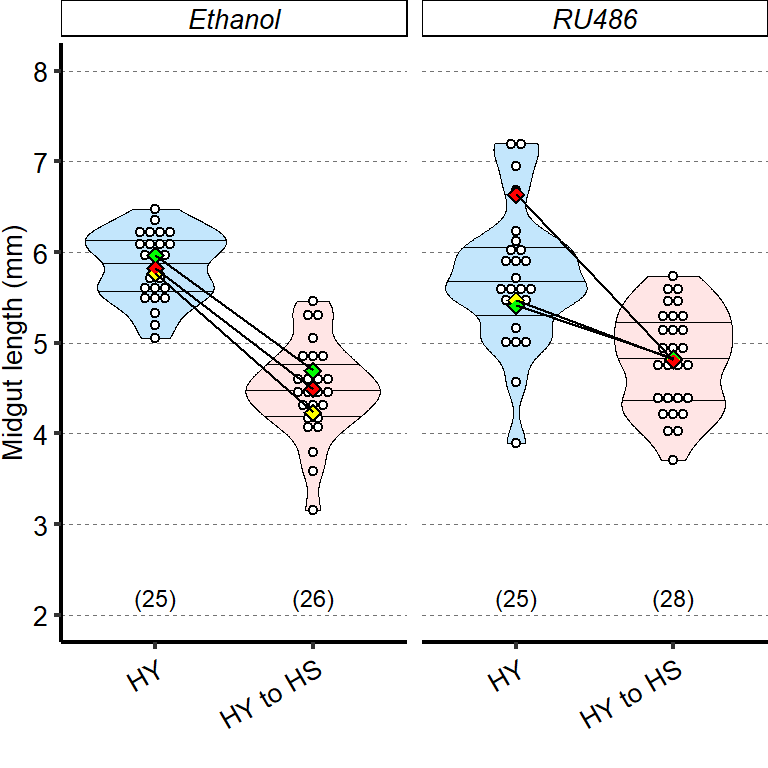

Supplement: Supplementary file 2. [file elife-64125-supp2.zip › Bonfini_script_GutPlasticity_diet_files/figure-html/Figure 7S2E-1.png]

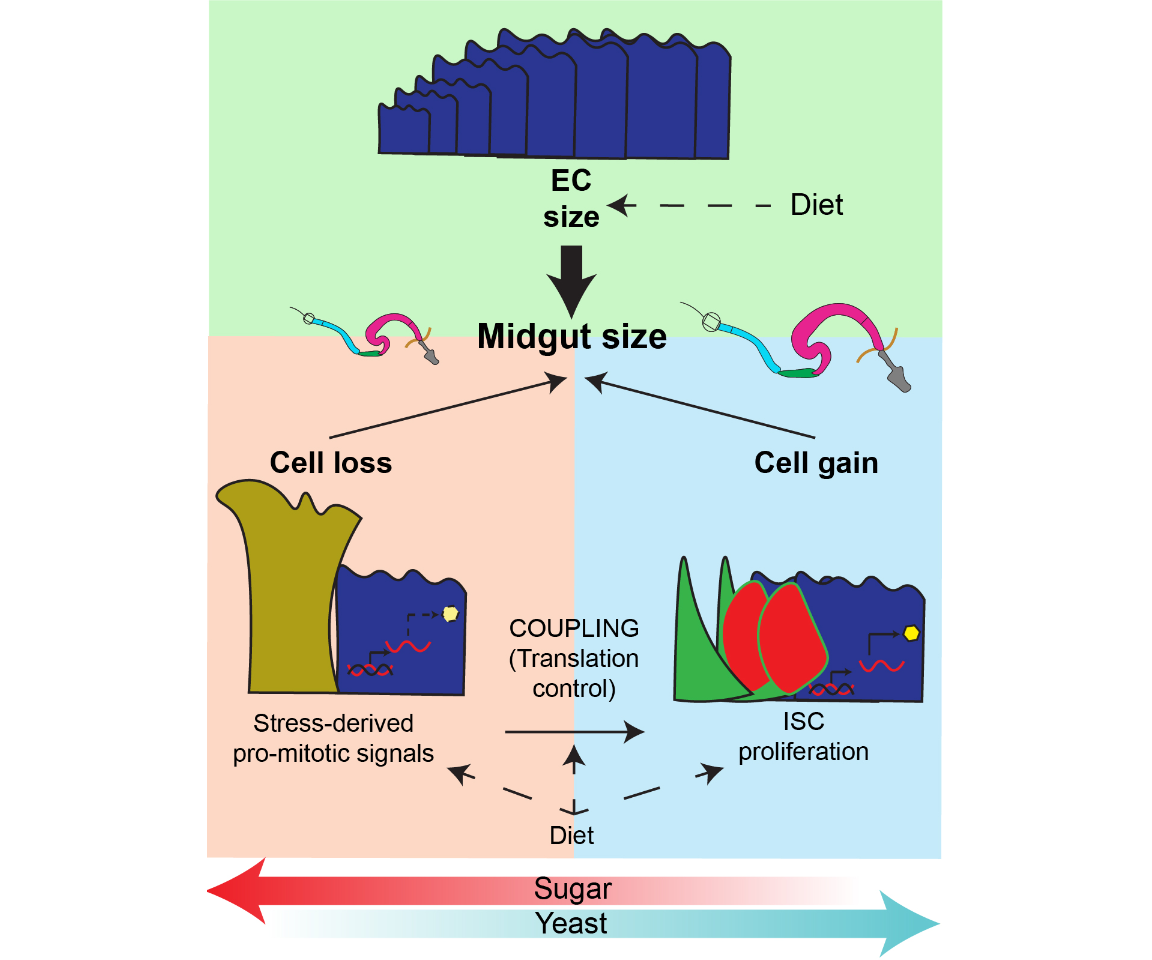

Supplement: Supplementary file 2. [file elife-64125-supp2.zip › Bonfini_script_GutPlasticity_diet_files/figure-html/Figure 7S3A-1.png]

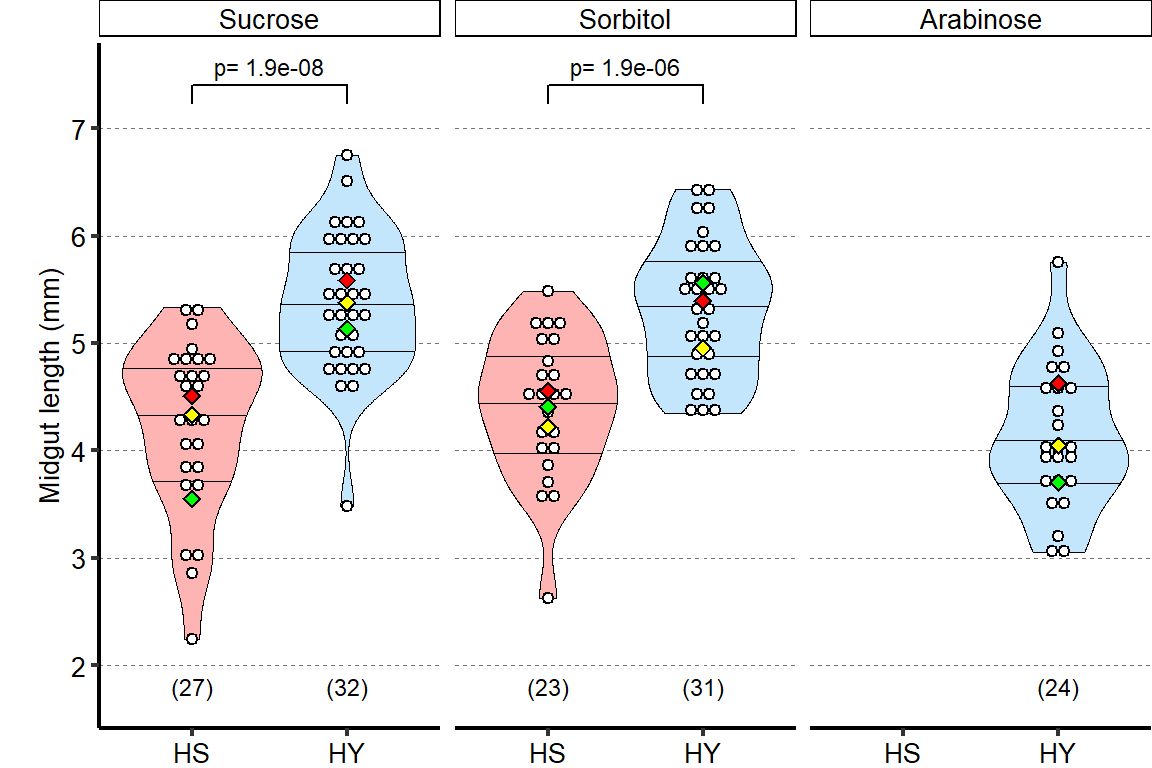

Supplement: Supplementary file 2. [file elife-64125-supp2.zip › Bonfini_script_GutPlasticity_diet_files/figure-html/Figure2S2C -1.png]
