# Supplementary figures and images for: Multiscale analysis reveals that diet-dependent midgut plasticity emerges from alterations in both stem cell niche coupling and enterocyte size (part 1 of 2)
Source: eLife. 2021 Sep 23;10:e64125. doi: 10.7554/eLife.64125 (PMC8528489; doi:10.7554/eLife.64125)

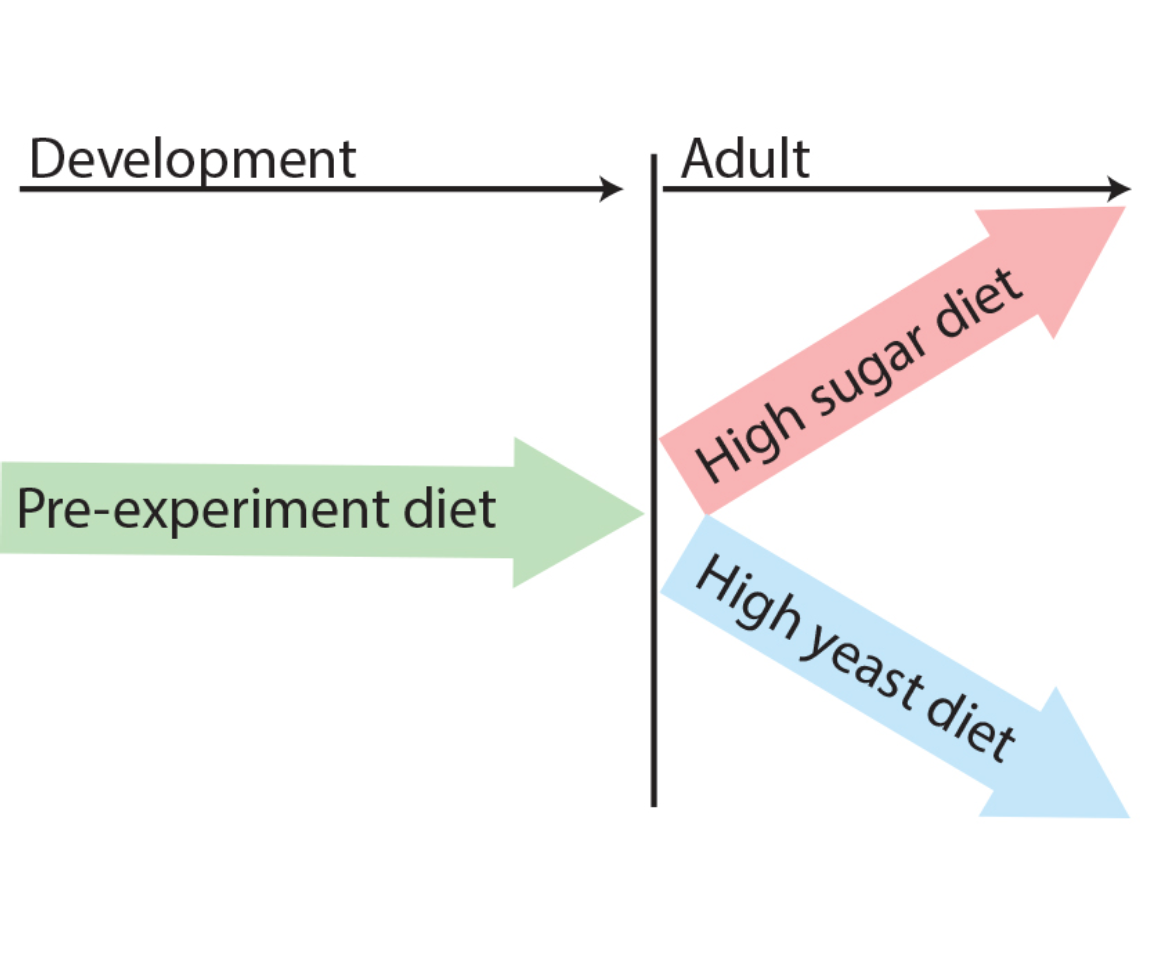

Supplement: Supplementary file 2. [file elife-64125-supp2.zip › Bonfini_script_GutPlasticity_diet_files/figure-html/Figure 1A-1.png]

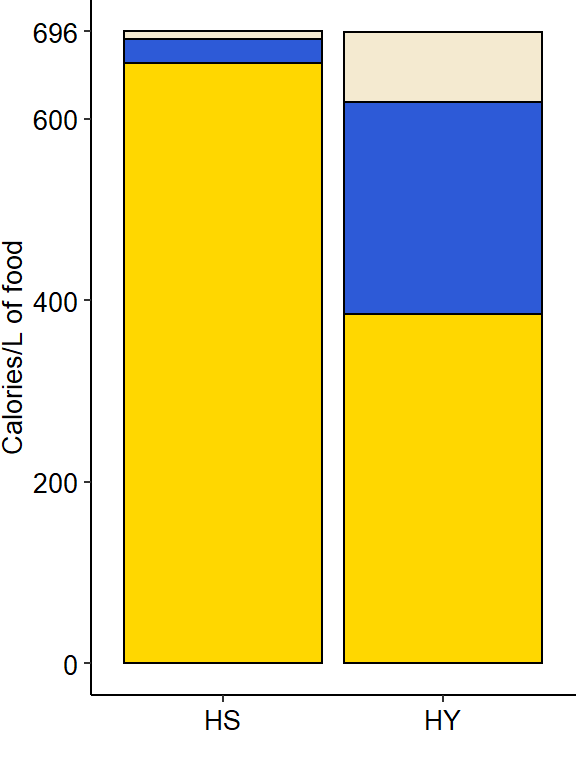

Supplement: Supplementary file 2. [file elife-64125-supp2.zip › Bonfini_script_GutPlasticity_diet_files/figure-html/Figure 1B-1.png]

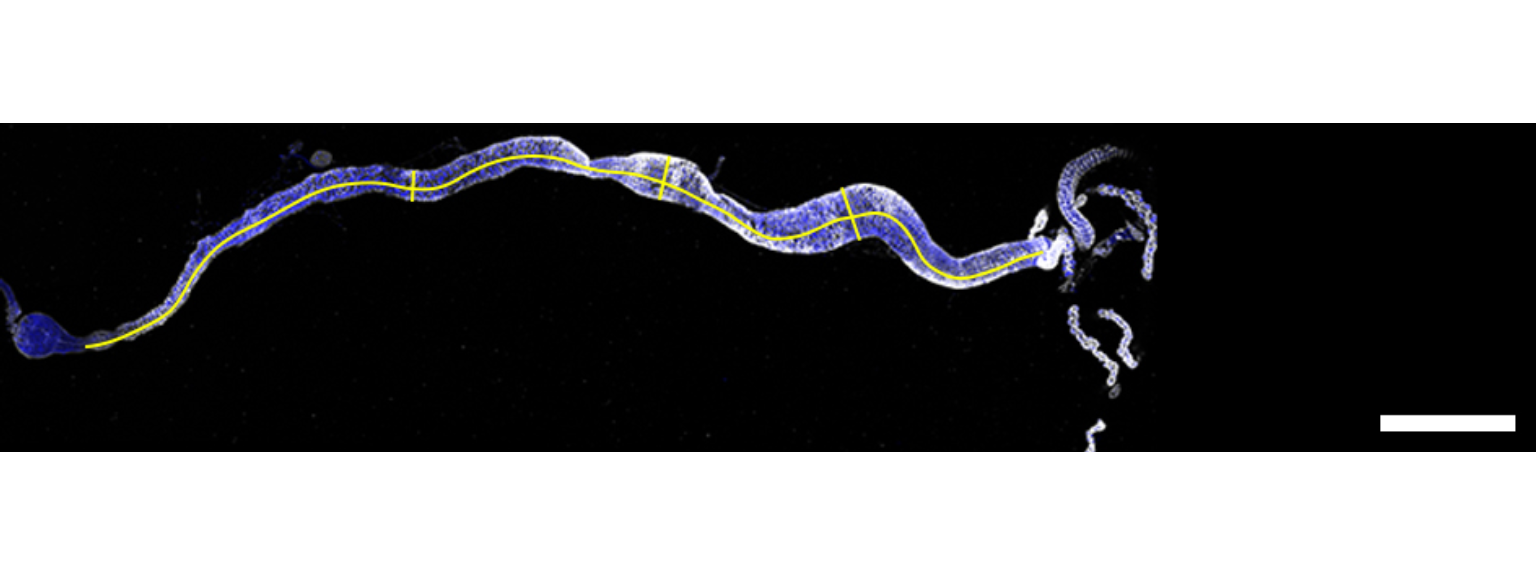

Supplement: Supplementary file 2. [file elife-64125-supp2.zip › Bonfini_script_GutPlasticity_diet_files/figure-html/Figure 1C-1.png]

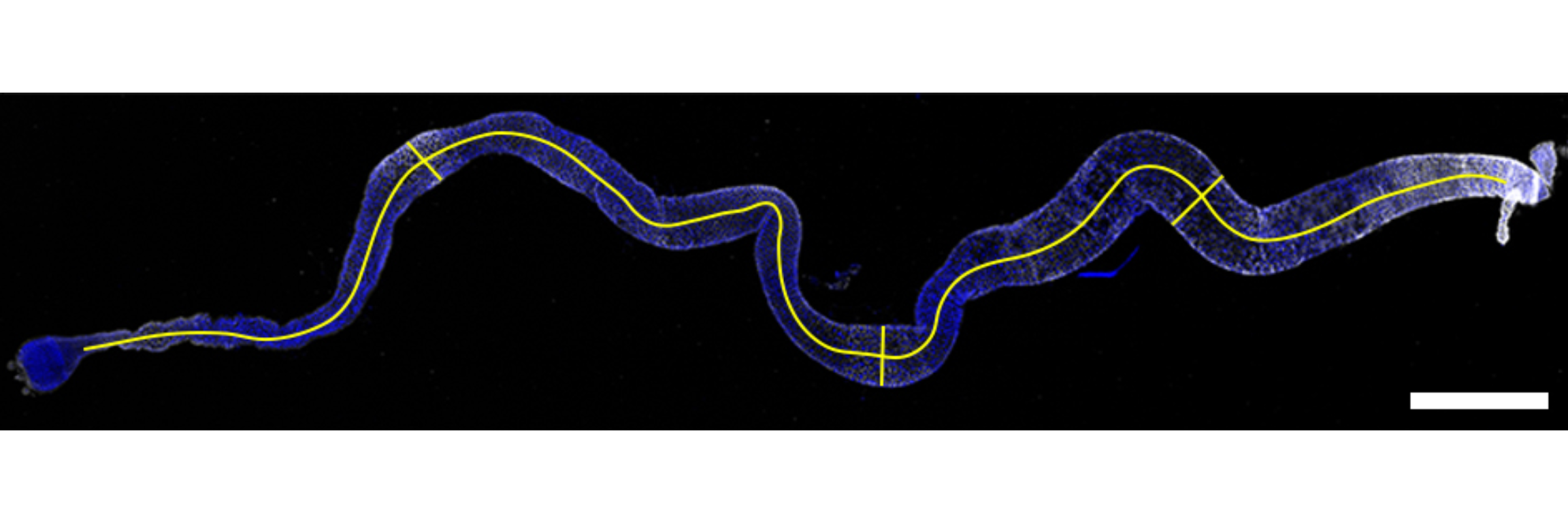

Supplement: Supplementary file 2. [file elife-64125-supp2.zip › Bonfini_script_GutPlasticity_diet_files/figure-html/Figure 1D-1.png]

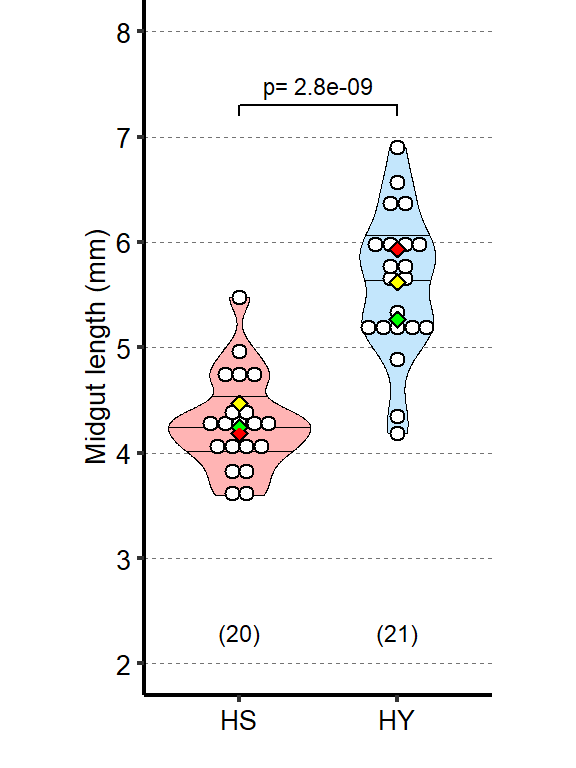

Supplement: Supplementary file 2. [file elife-64125-supp2.zip › Bonfini_script_GutPlasticity_diet_files/figure-html/Figure 1E-1.png]

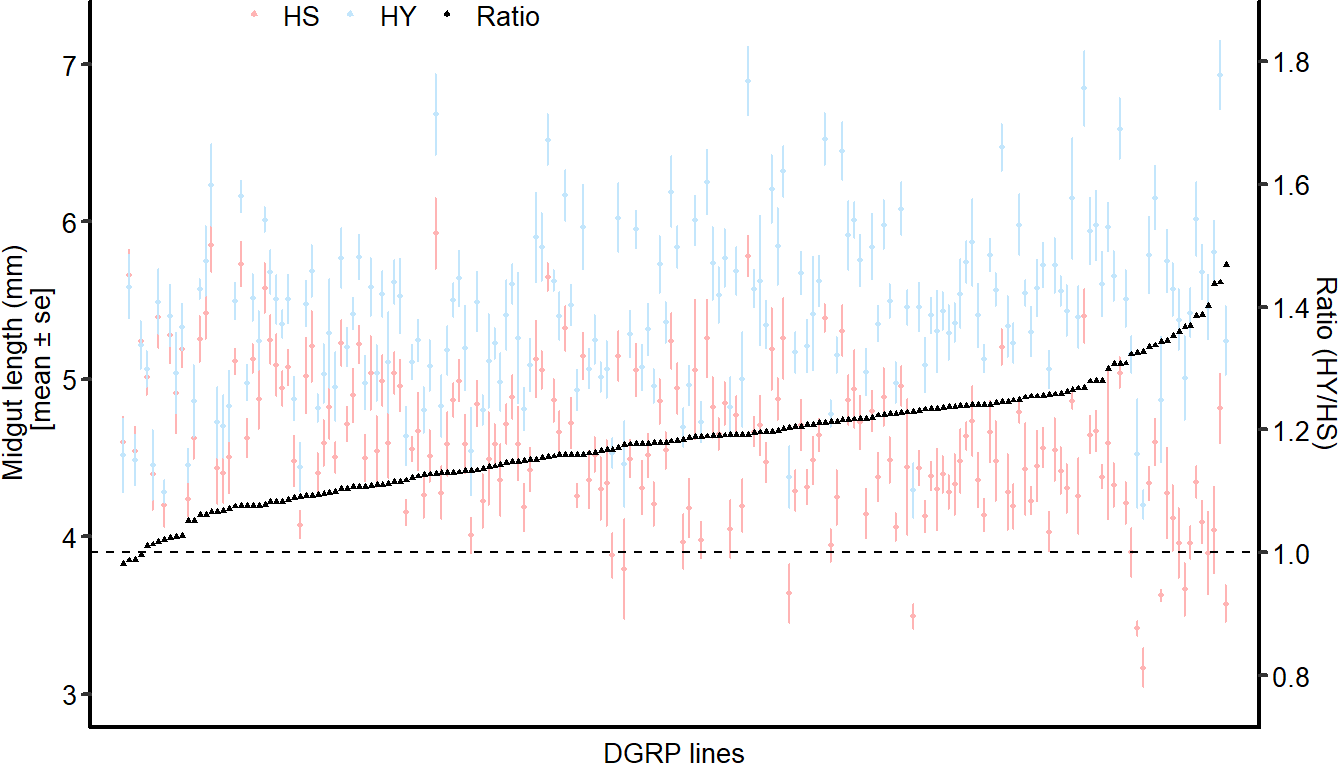

Supplement: Supplementary file 2. [file elife-64125-supp2.zip › Bonfini_script_GutPlasticity_diet_files/figure-html/Figure 1F-1.png]

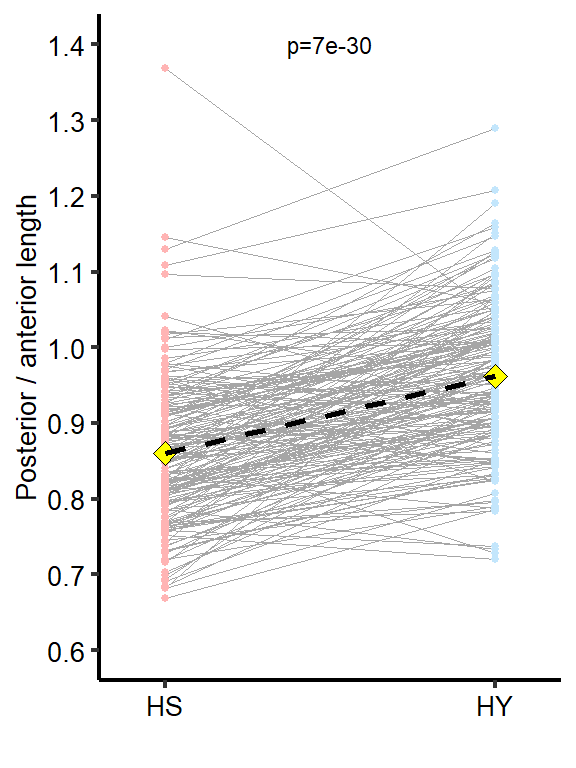

Supplement: Supplementary file 2. [file elife-64125-supp2.zip › Bonfini_script_GutPlasticity_diet_files/figure-html/Figure 1G-1.png]

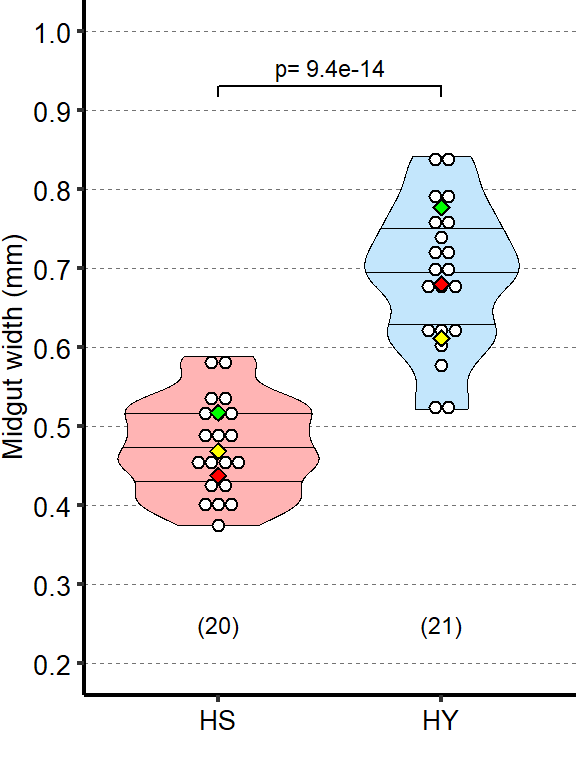

Supplement: Supplementary file 2. [file elife-64125-supp2.zip › Bonfini_script_GutPlasticity_diet_files/figure-html/Figure 1S1A-1.png]

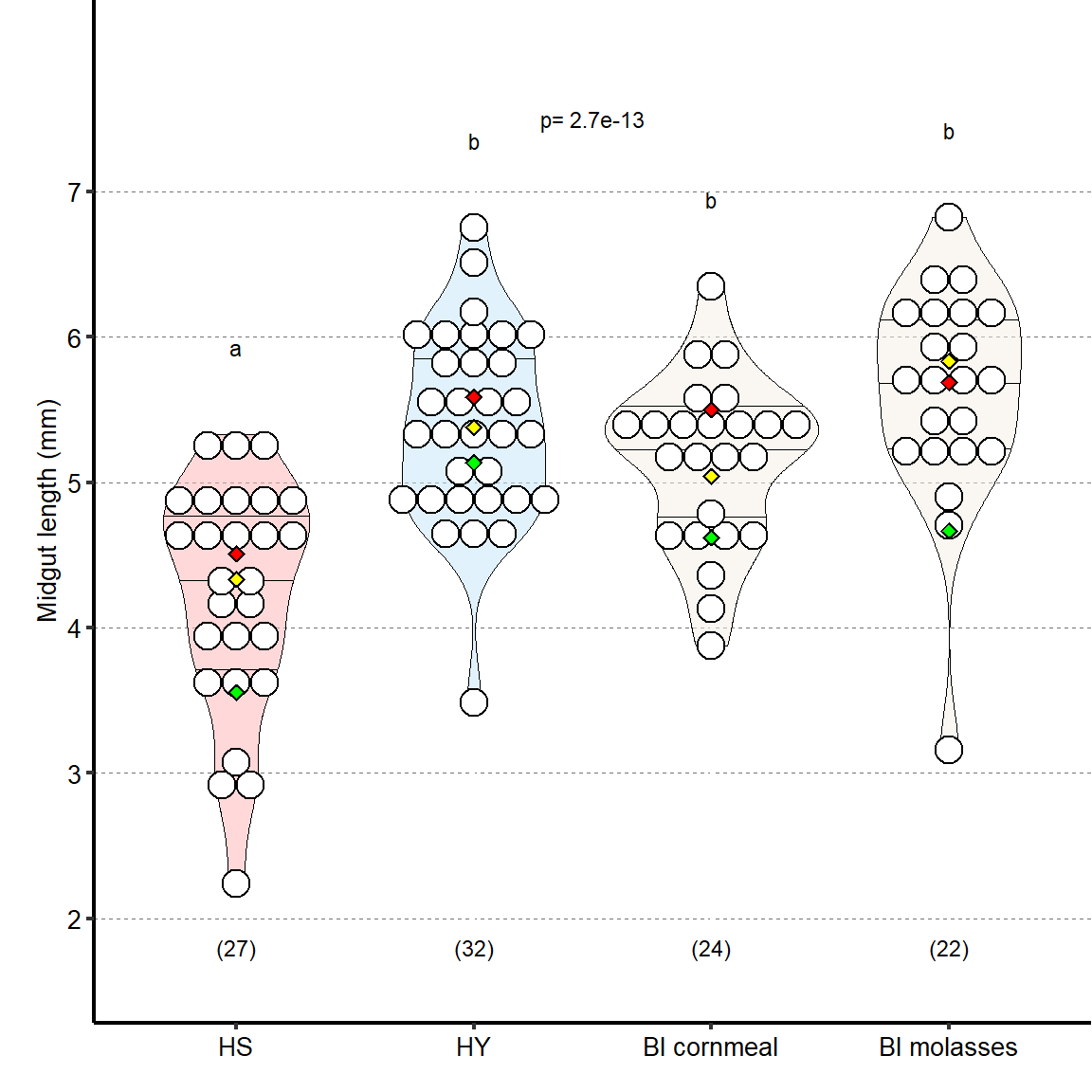

Supplement: Supplementary file 2. [file elife-64125-supp2.zip › Bonfini_script_GutPlasticity_diet_files/figure-html/Figure 1S1B-1.png]

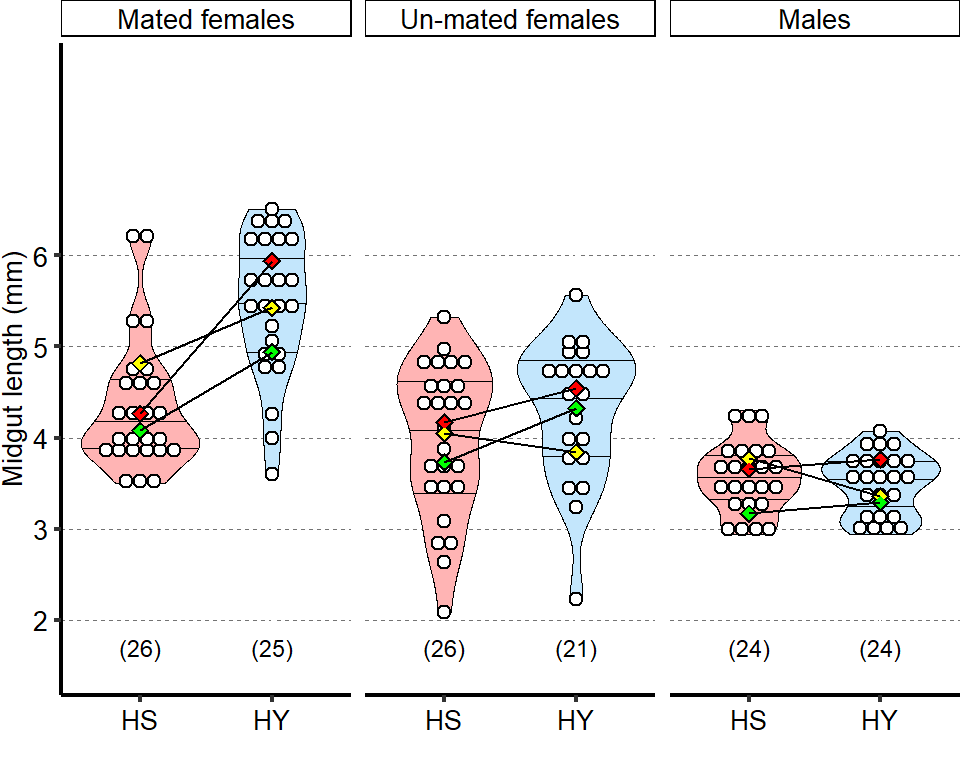

Supplement: Supplementary file 2. [file elife-64125-supp2.zip › Bonfini_script_GutPlasticity_diet_files/figure-html/Figure 1S1C-1.png]

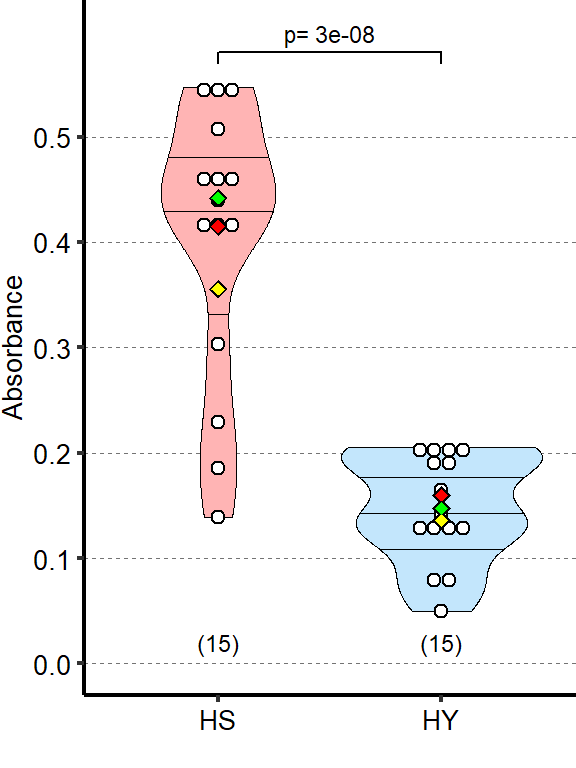

Supplement: Supplementary file 2. [file elife-64125-supp2.zip › Bonfini_script_GutPlasticity_diet_files/figure-html/Figure 1S1D-1.png]

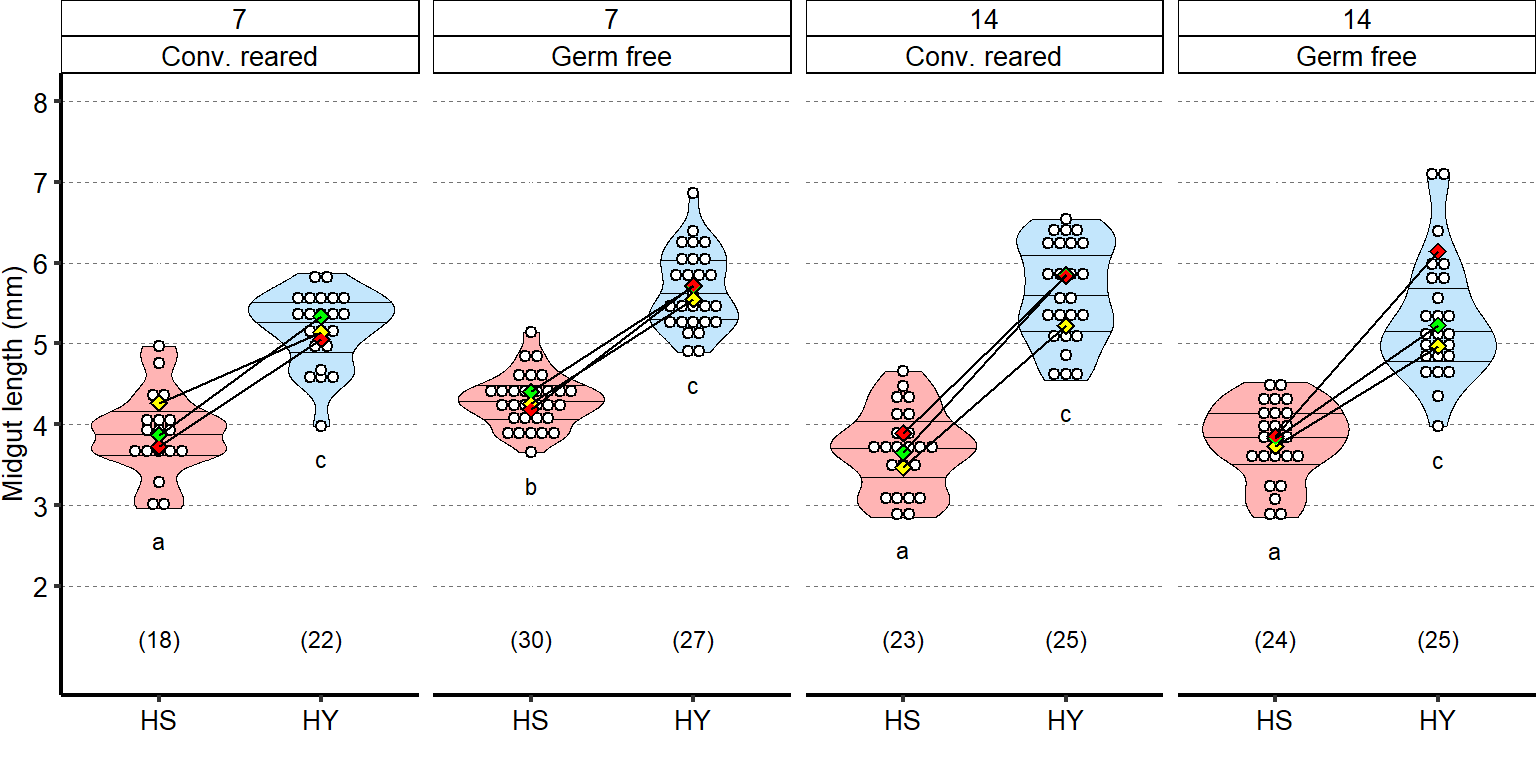

Supplement: Supplementary file 2. [file elife-64125-supp2.zip › Bonfini_script_GutPlasticity_diet_files/figure-html/Figure 1S1E-1.png]

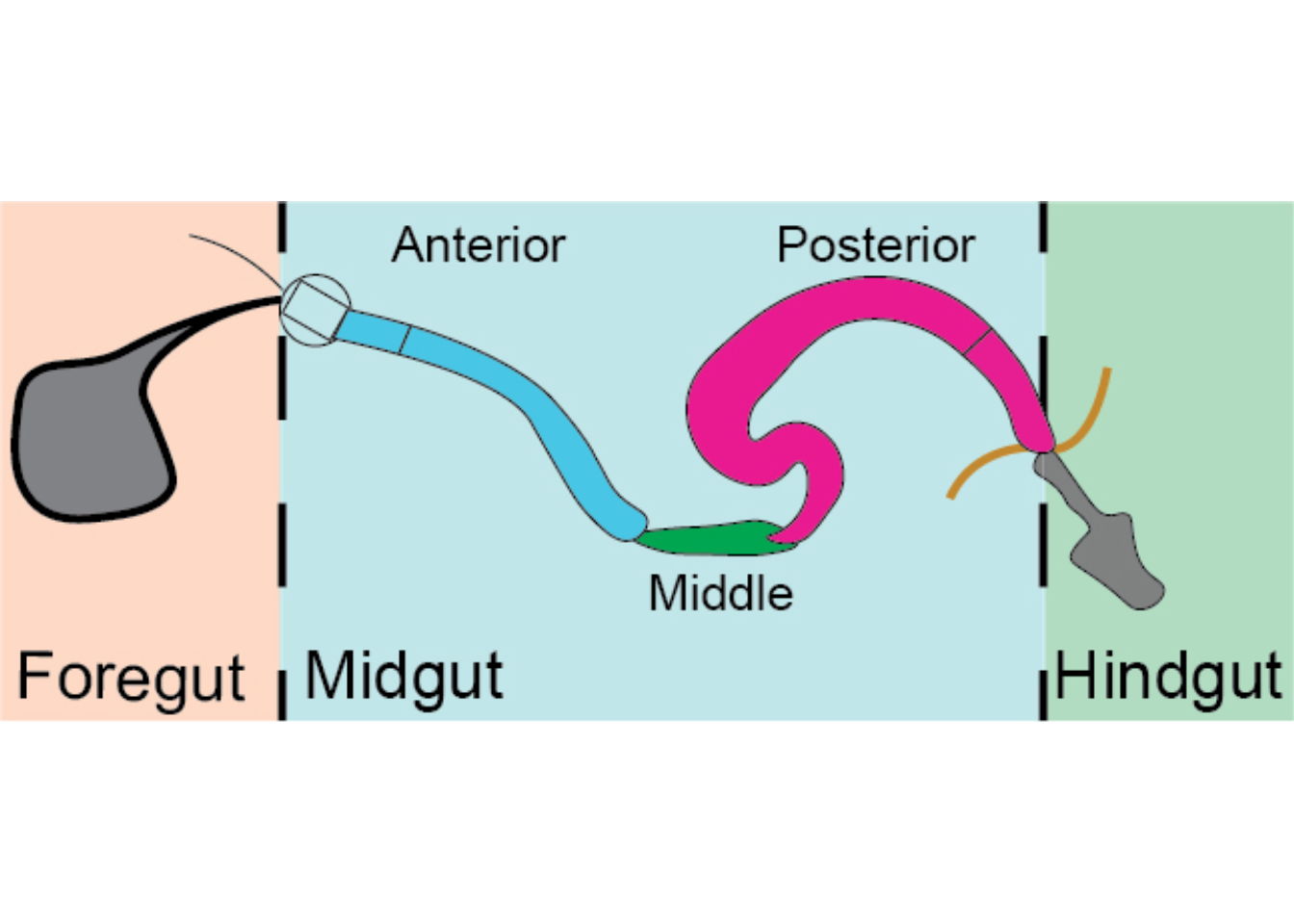

Supplement: Supplementary file 2. [file elife-64125-supp2.zip › Bonfini_script_GutPlasticity_diet_files/figure-html/Figure 1S2A-1.png]

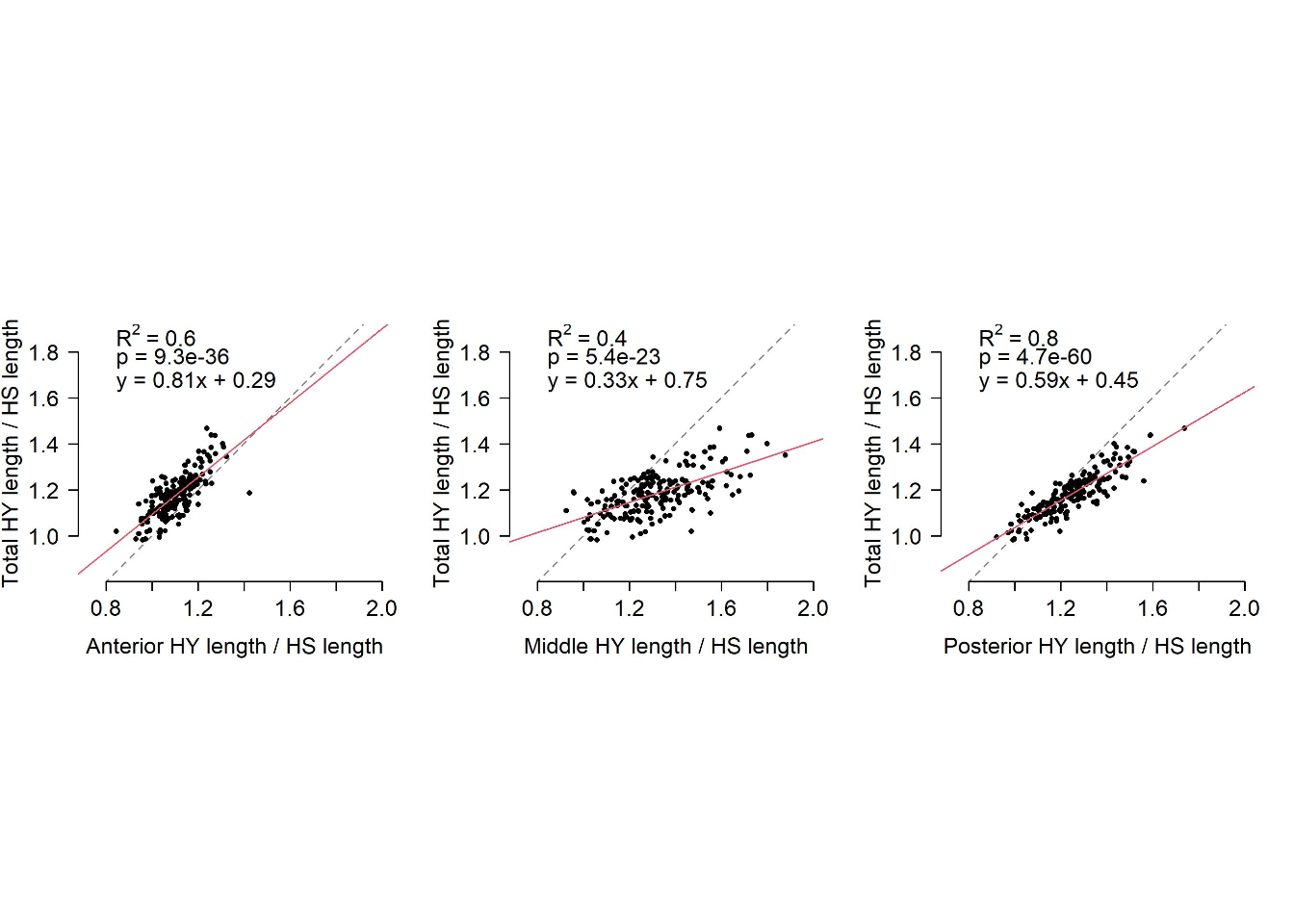

Supplement: Supplementary file 2. [file elife-64125-supp2.zip › Bonfini_script_GutPlasticity_diet_files/figure-html/Figure 1S2B-1.png]

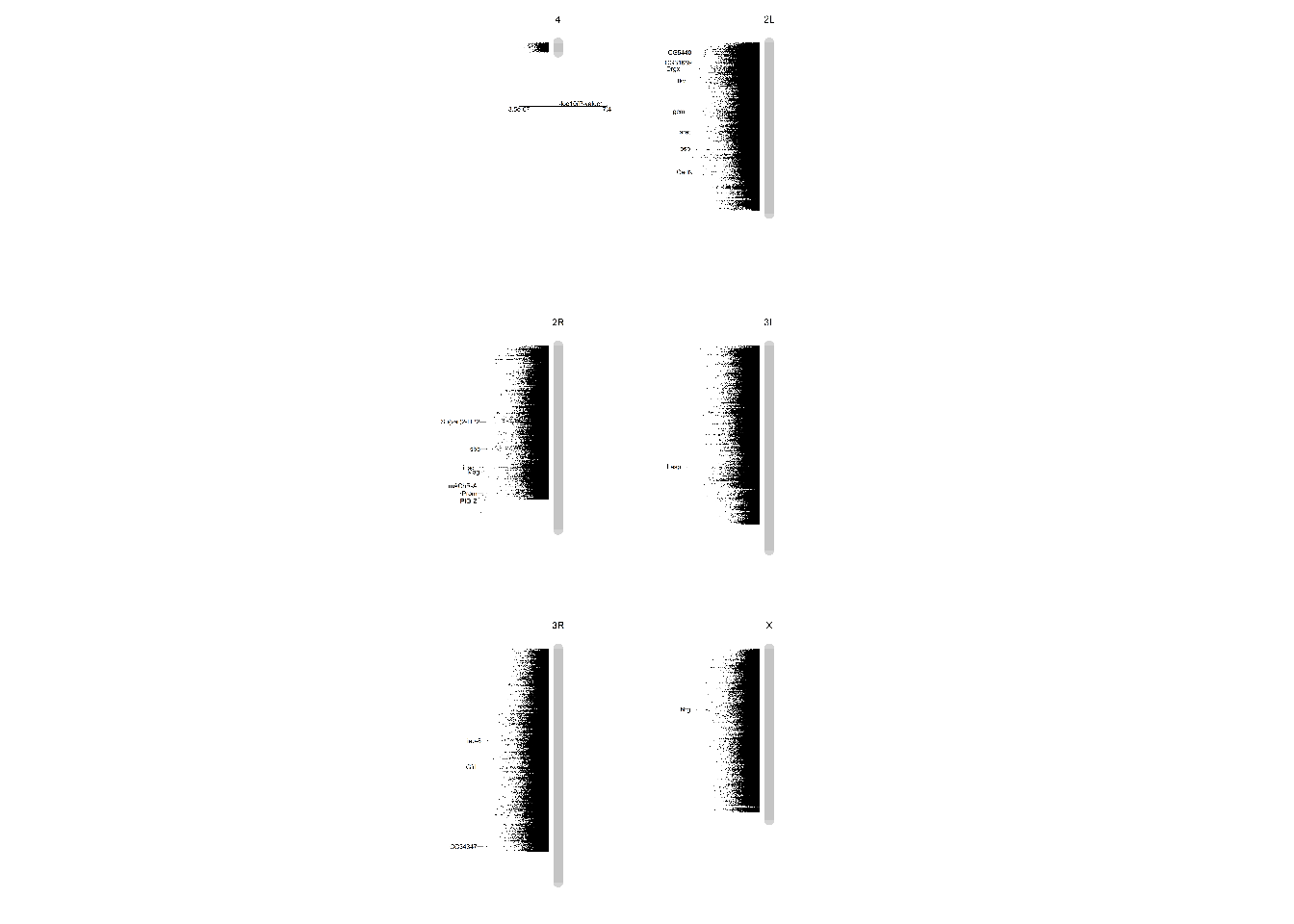

Supplement: Supplementary file 2. [file elife-64125-supp2.zip › Bonfini_script_GutPlasticity_diet_files/figure-html/Figure 1S3A-1.png]

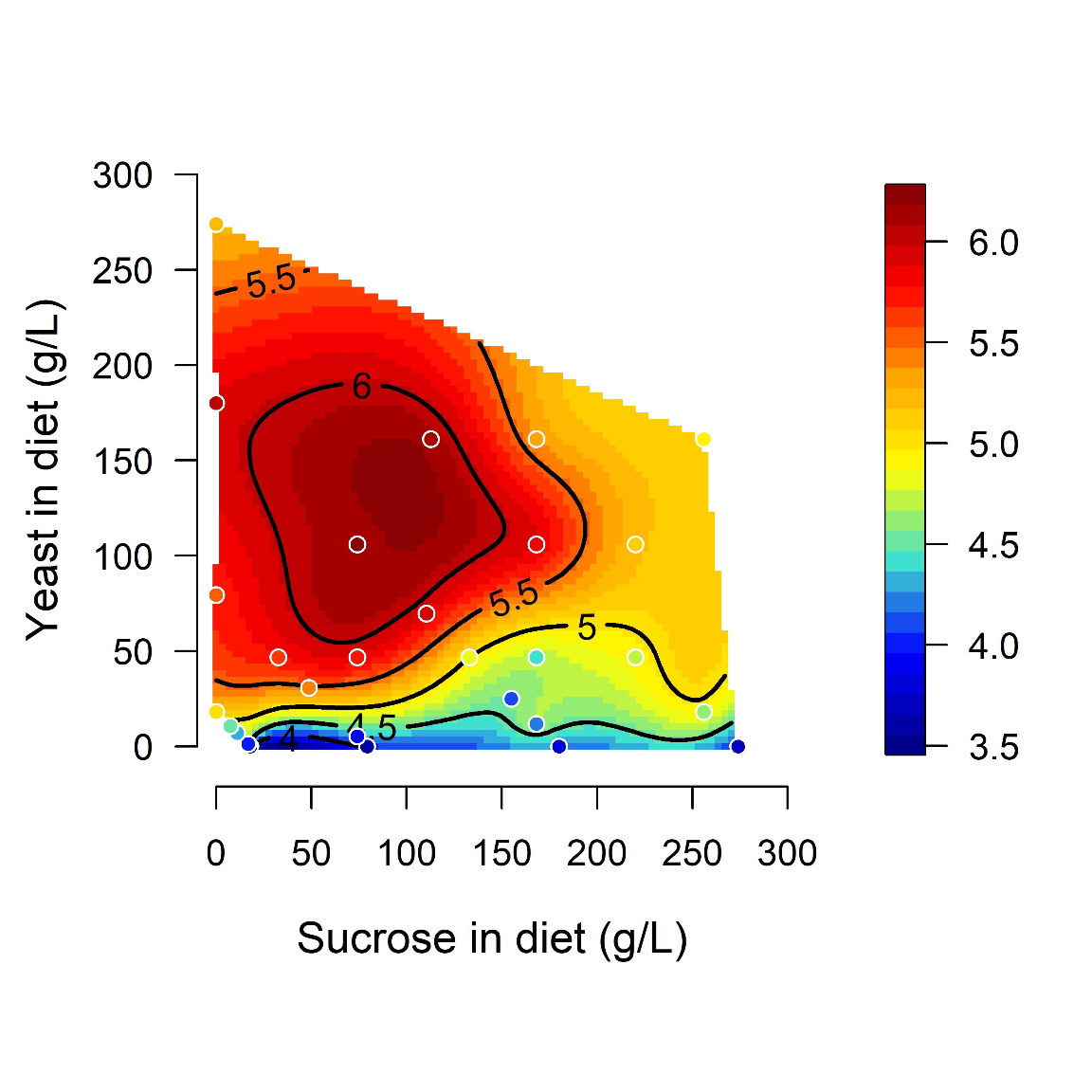

Supplement: Supplementary file 2. [file elife-64125-supp2.zip › Bonfini_script_GutPlasticity_diet_files/figure-html/Figure 2A-1.png]

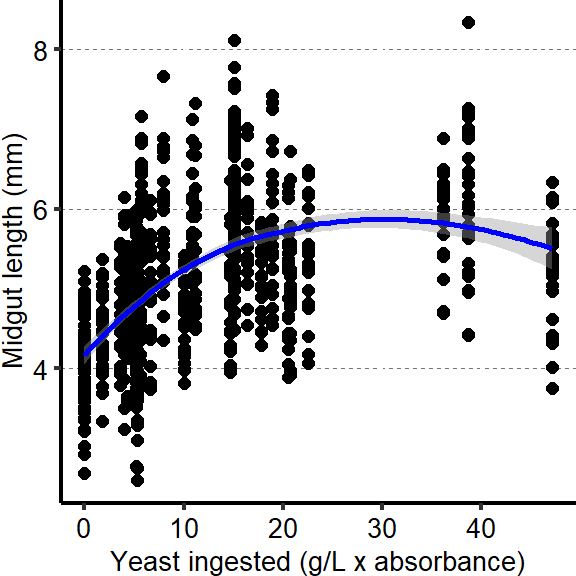

Supplement: Supplementary file 2. [file elife-64125-supp2.zip › Bonfini_script_GutPlasticity_diet_files/figure-html/Figure 2B-1.png]

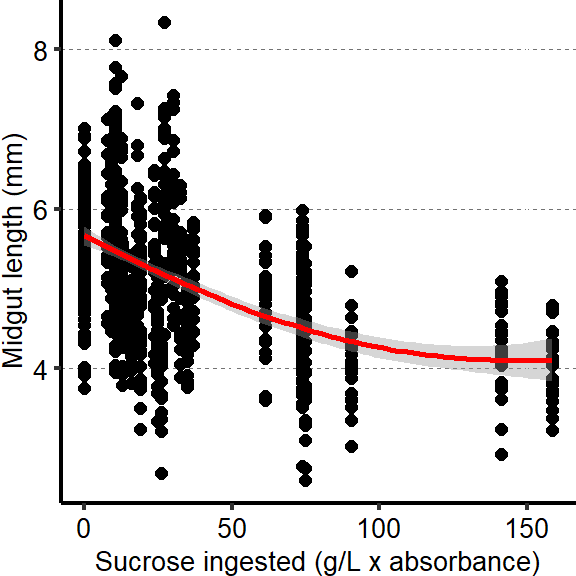

Supplement: Supplementary file 2. [file elife-64125-supp2.zip › Bonfini_script_GutPlasticity_diet_files/figure-html/Figure 2C-1.png]

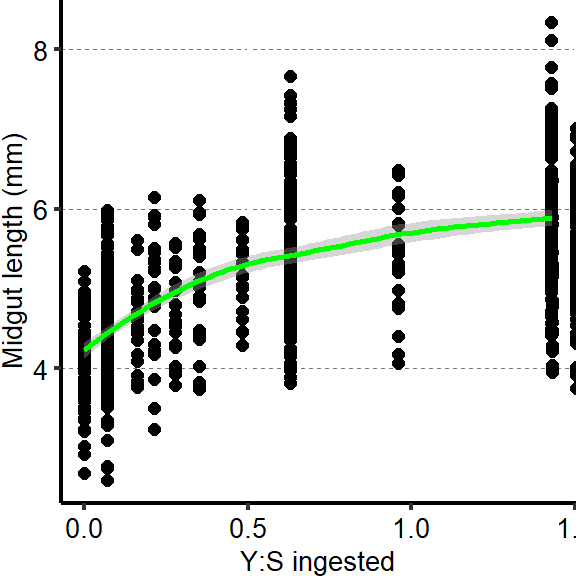

Supplement: Supplementary file 2. [file elife-64125-supp2.zip › Bonfini_script_GutPlasticity_diet_files/figure-html/Figure 2D-1.png]

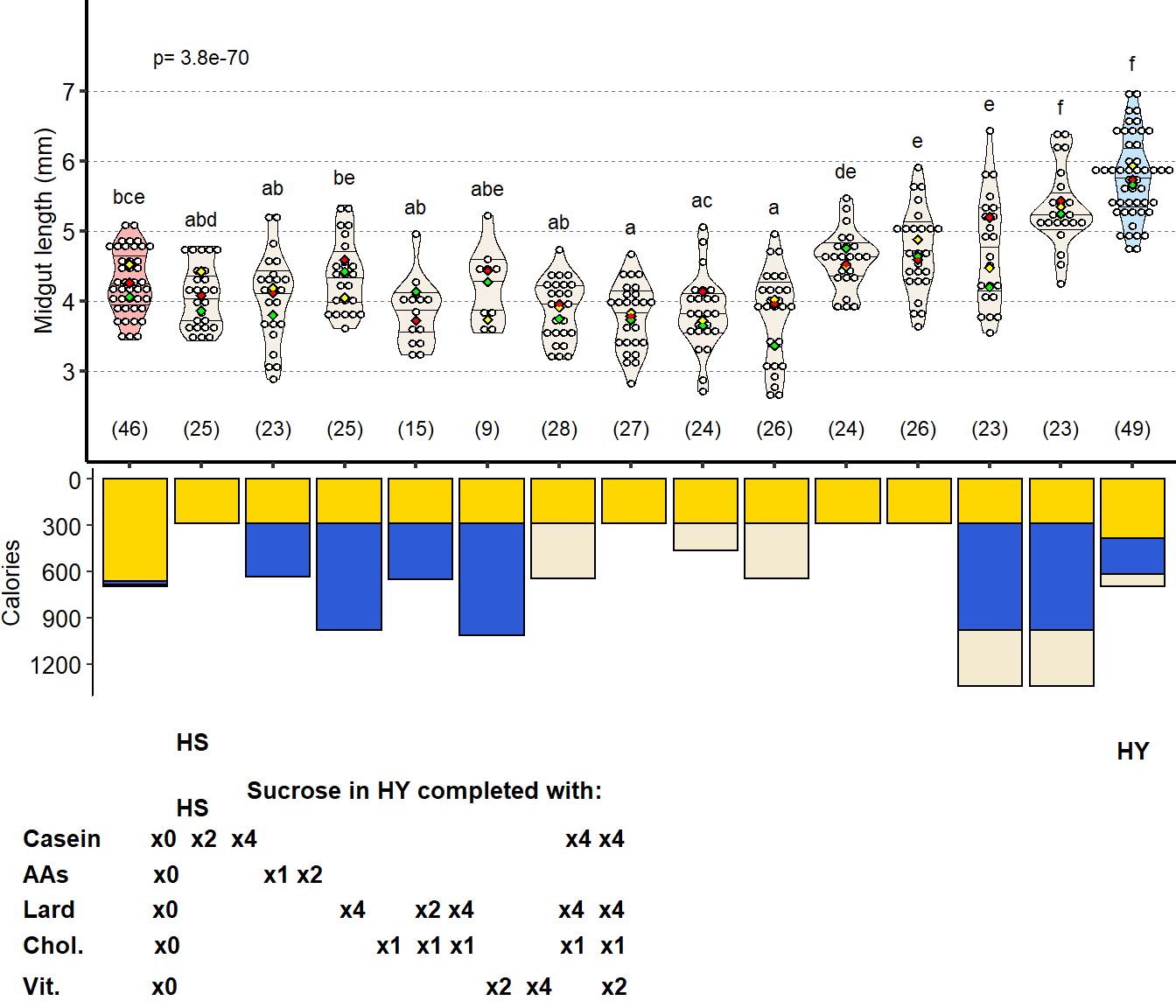

Supplement: Supplementary file 2. [file elife-64125-supp2.zip › Bonfini_script_GutPlasticity_diet_files/figure-html/Figure 2E-1.png]

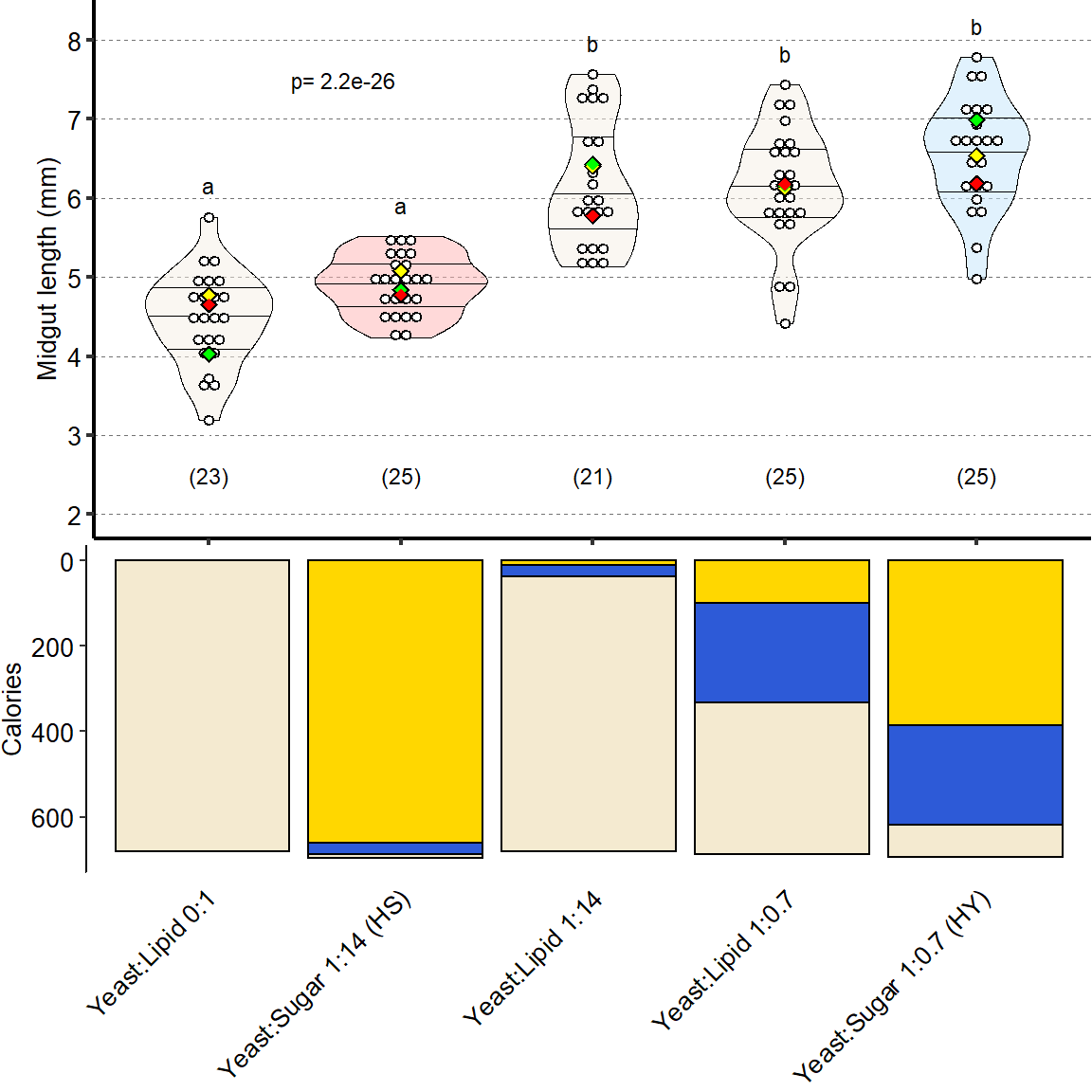

Supplement: Supplementary file 2. [file elife-64125-supp2.zip › Bonfini_script_GutPlasticity_diet_files/figure-html/Figure 2F-1.png]

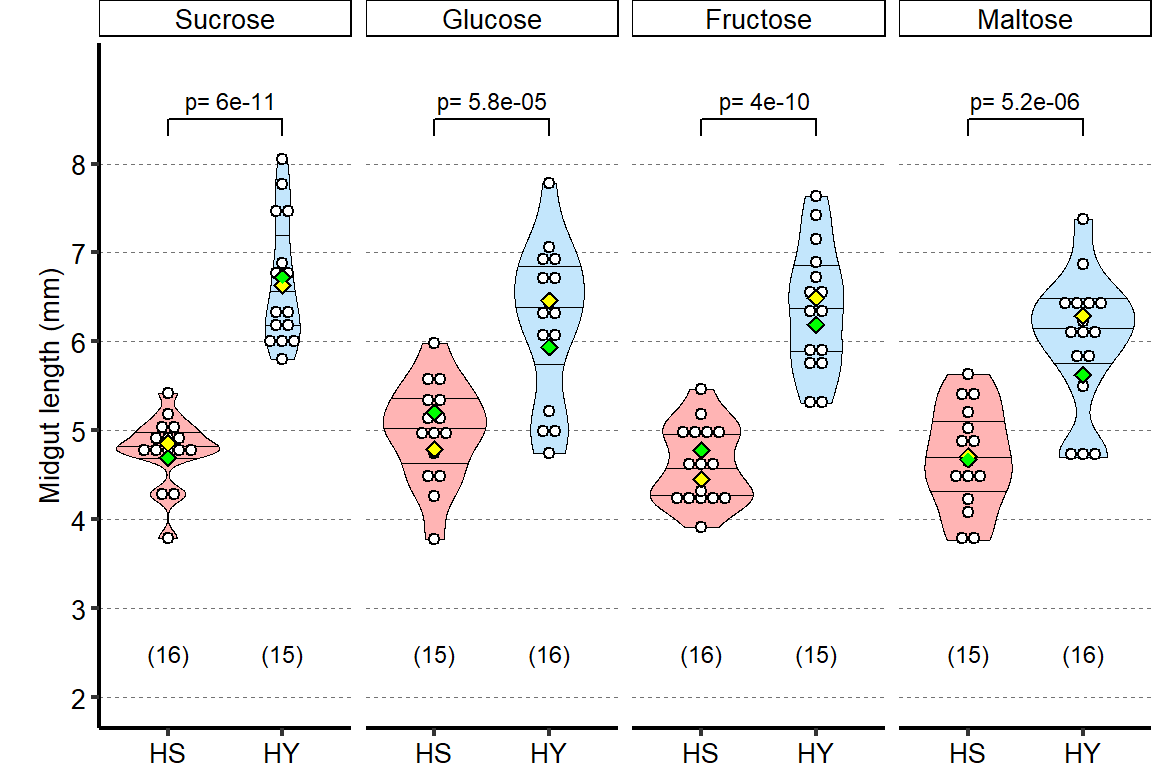

Supplement: Supplementary file 2. [file elife-64125-supp2.zip › Bonfini_script_GutPlasticity_diet_files/figure-html/Figure 2G-1.png]

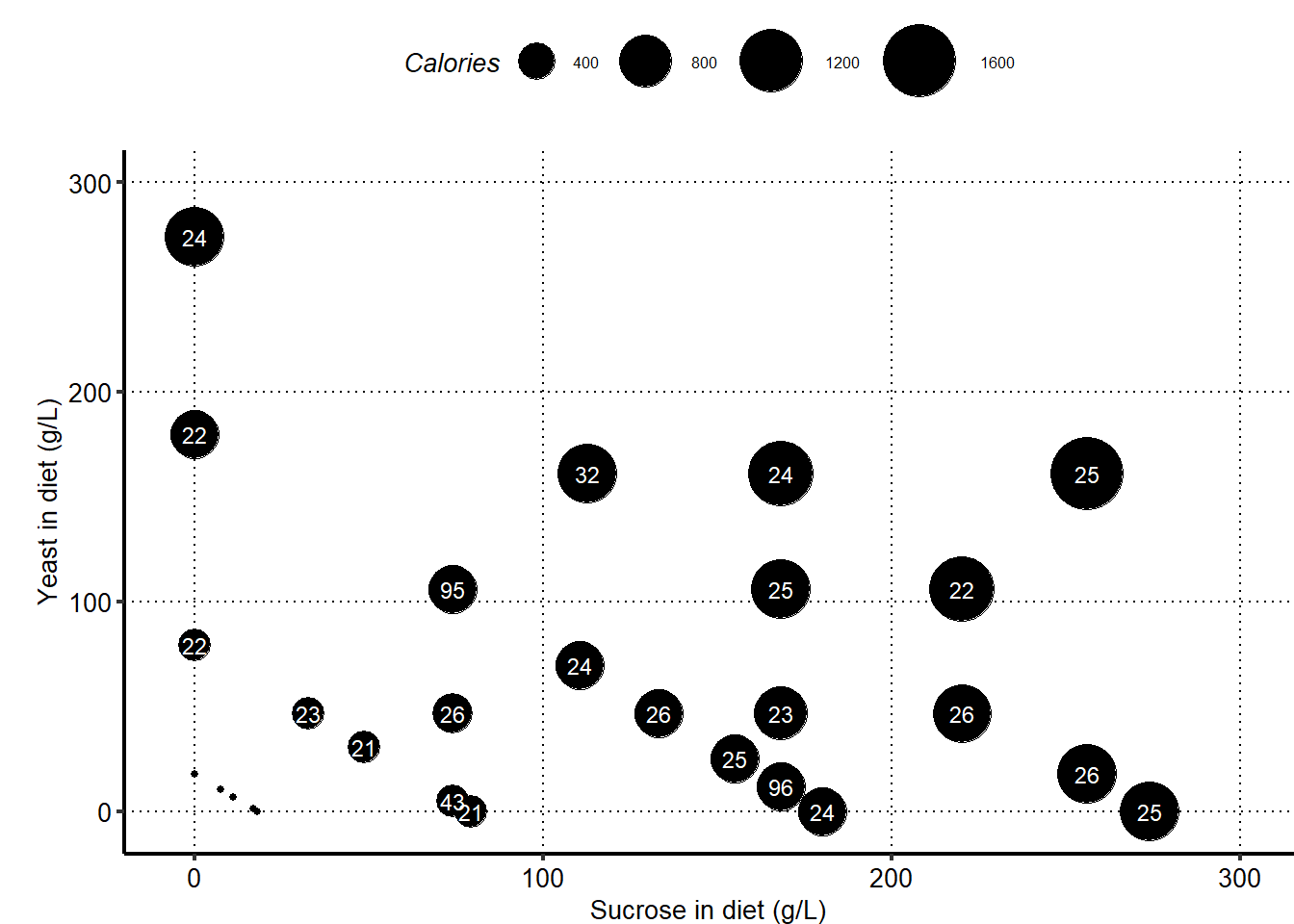

Supplement: Supplementary file 2. [file elife-64125-supp2.zip › Bonfini_script_GutPlasticity_diet_files/figure-html/Figure 2S1A-1.png]

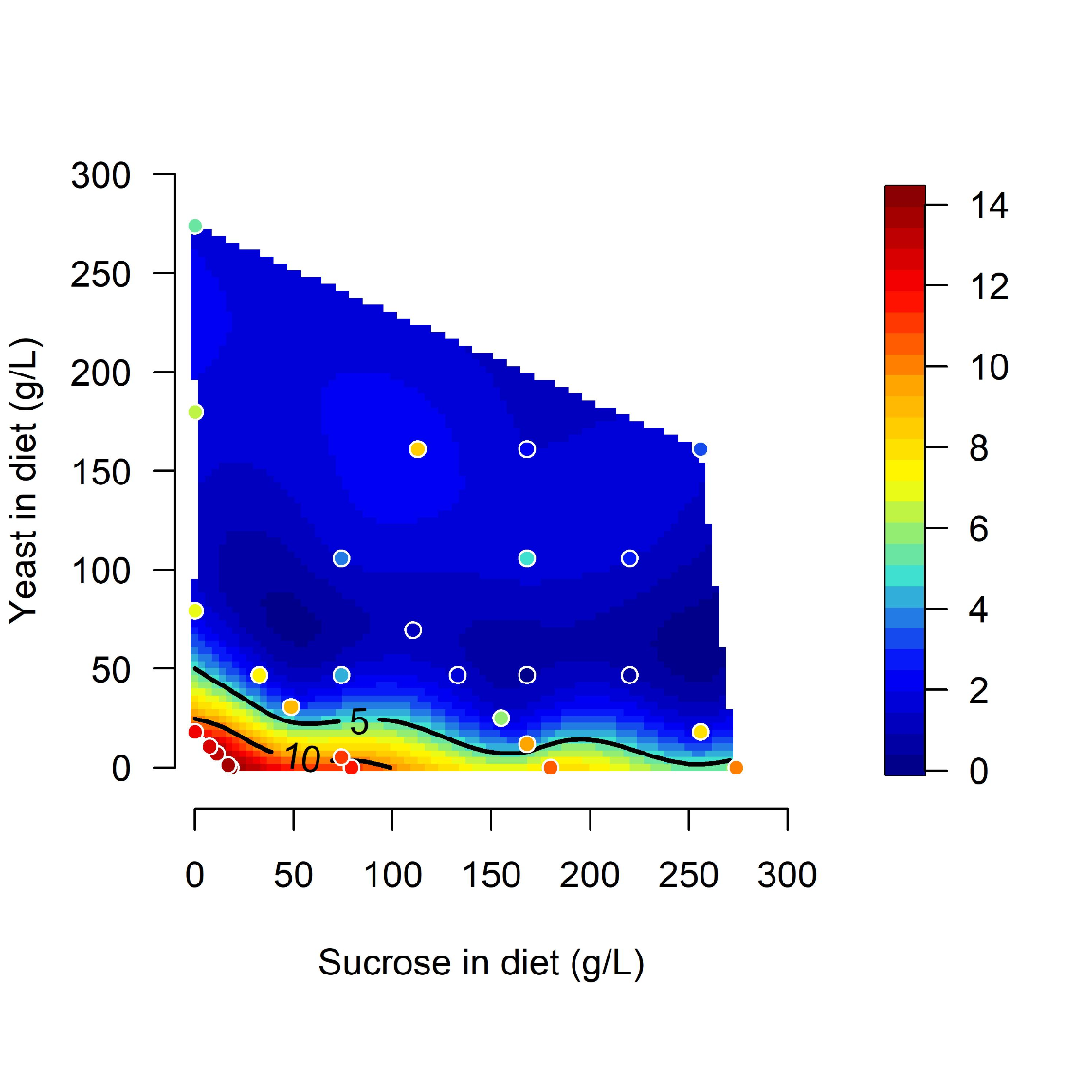

Supplement: Supplementary file 2. [file elife-64125-supp2.zip › Bonfini_script_GutPlasticity_diet_files/figure-html/Figure 2S1B-1.png]

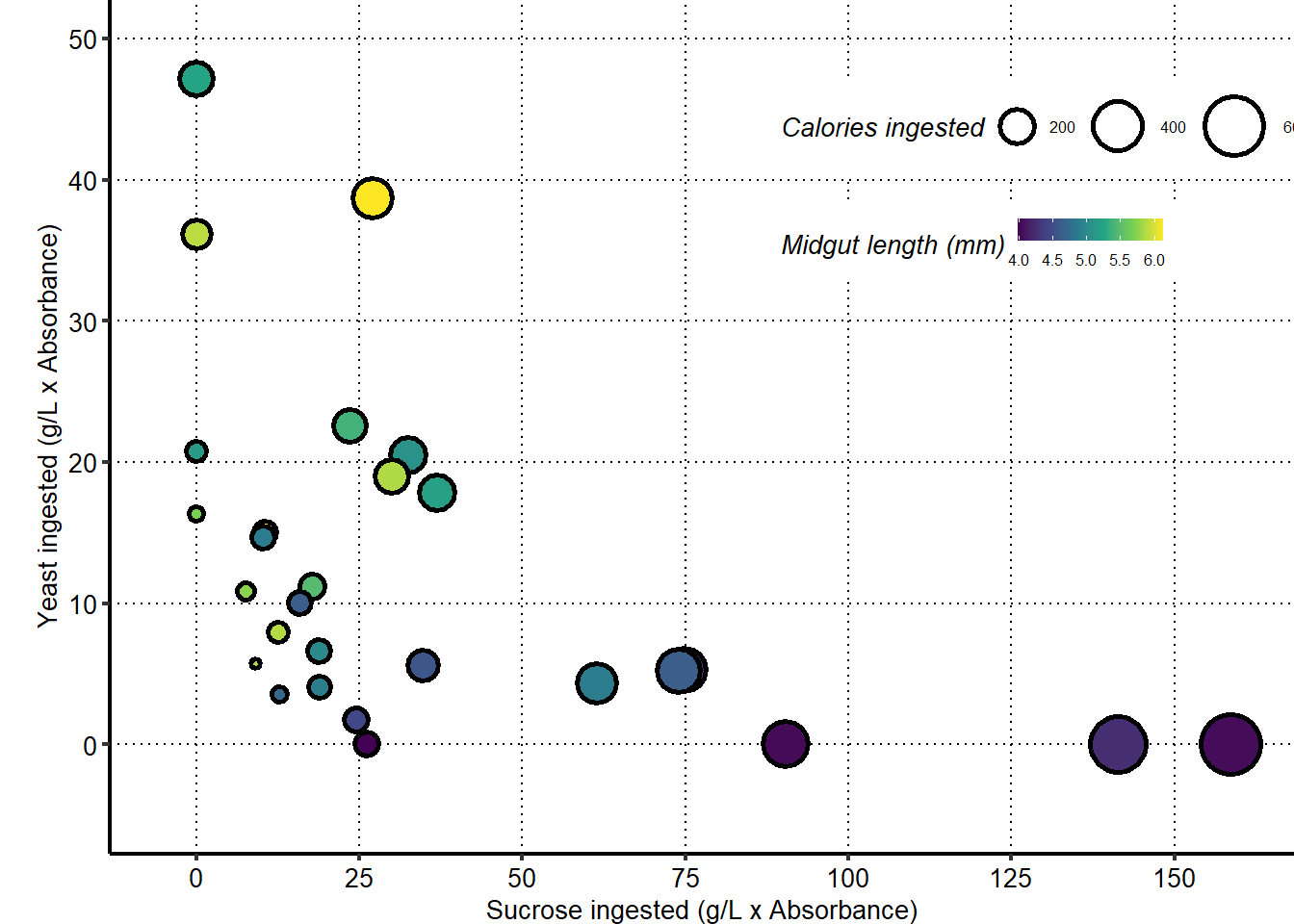

Supplement: Supplementary file 2. [file elife-64125-supp2.zip › Bonfini_script_GutPlasticity_diet_files/figure-html/Figure 2S1C-1.png]

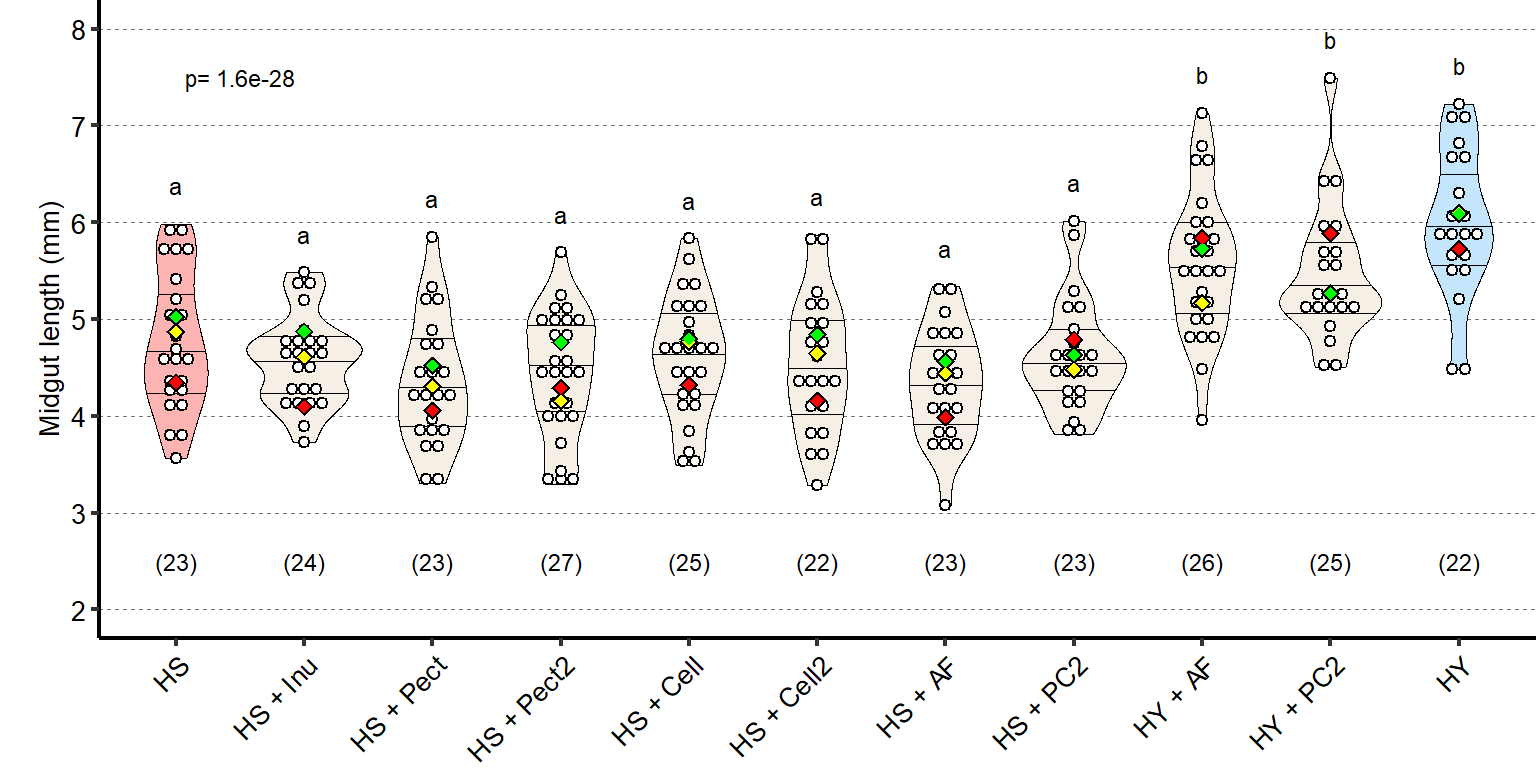

Supplement: Supplementary file 2. [file elife-64125-supp2.zip › Bonfini_script_GutPlasticity_diet_files/figure-html/Figure 2S2A-1.png]

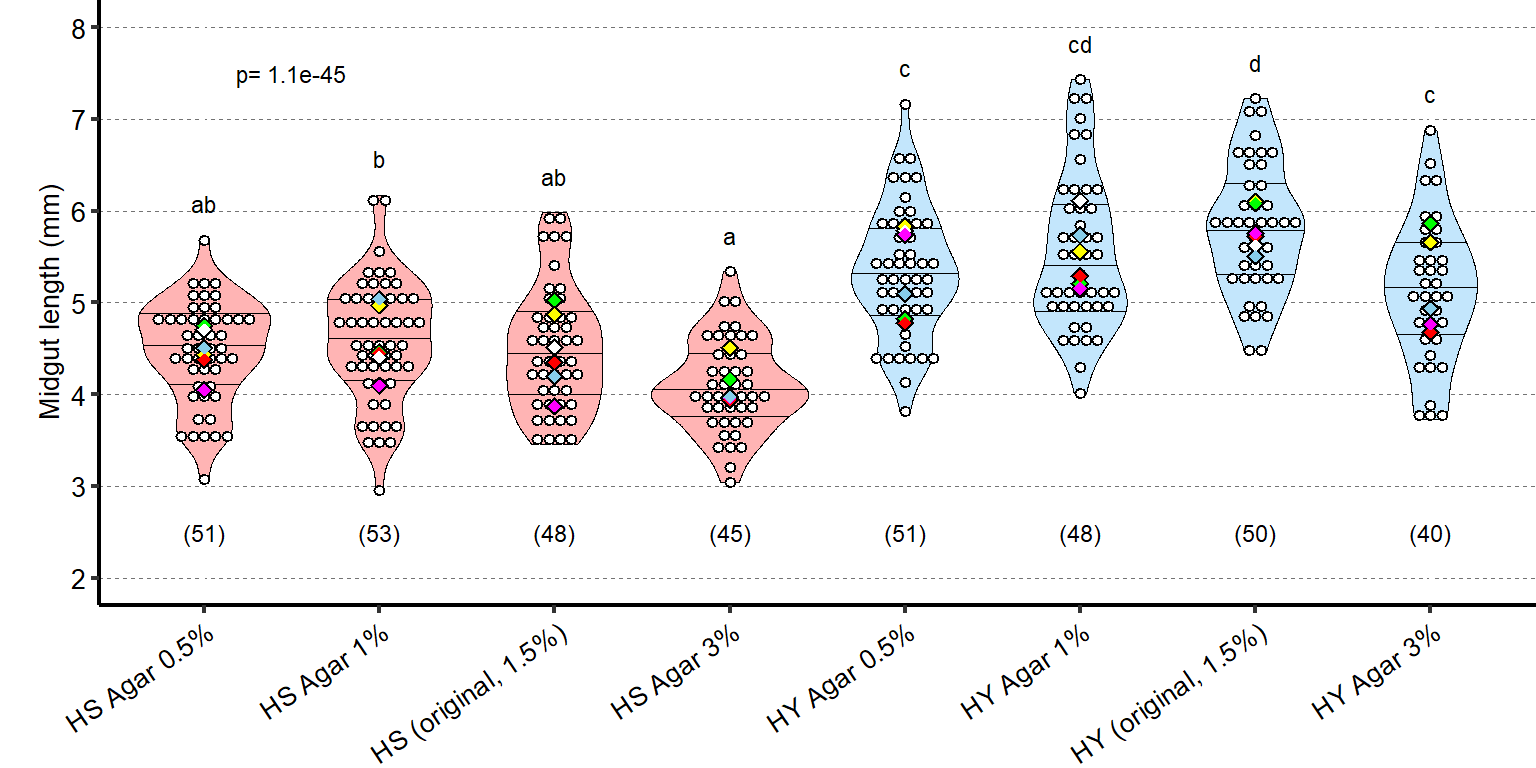

Supplement: Supplementary file 2. [file elife-64125-supp2.zip › Bonfini_script_GutPlasticity_diet_files/figure-html/Figure 2S2B-1.png]

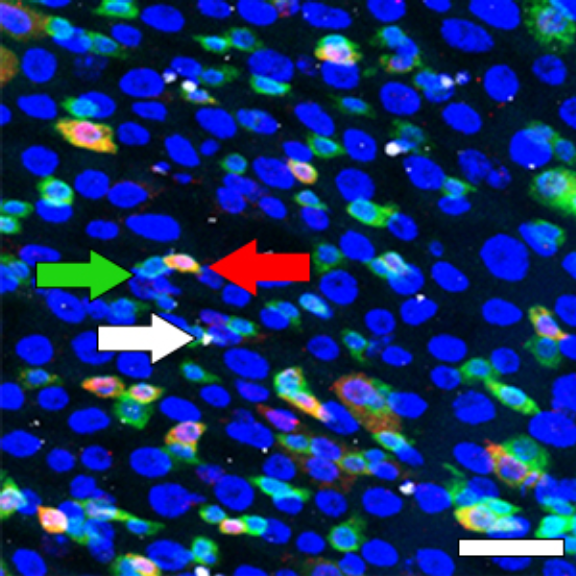

Supplement: Supplementary file 2. [file elife-64125-supp2.zip › Bonfini_script_GutPlasticity_diet_files/figure-html/Figure 3A-1.png]

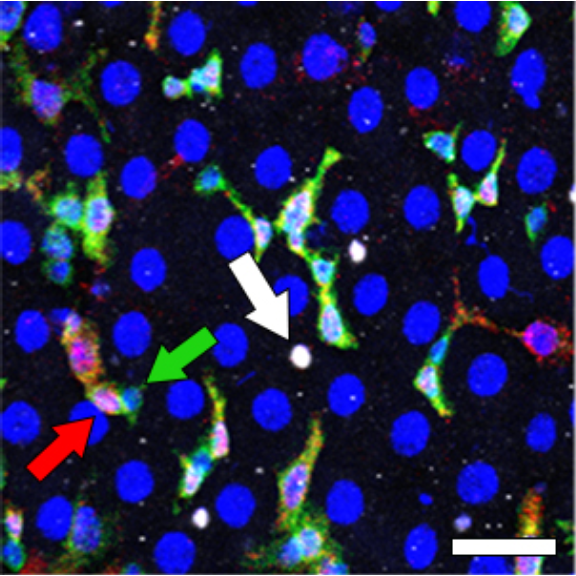

Supplement: Supplementary file 2. [file elife-64125-supp2.zip › Bonfini_script_GutPlasticity_diet_files/figure-html/Figure 3B-1.png]

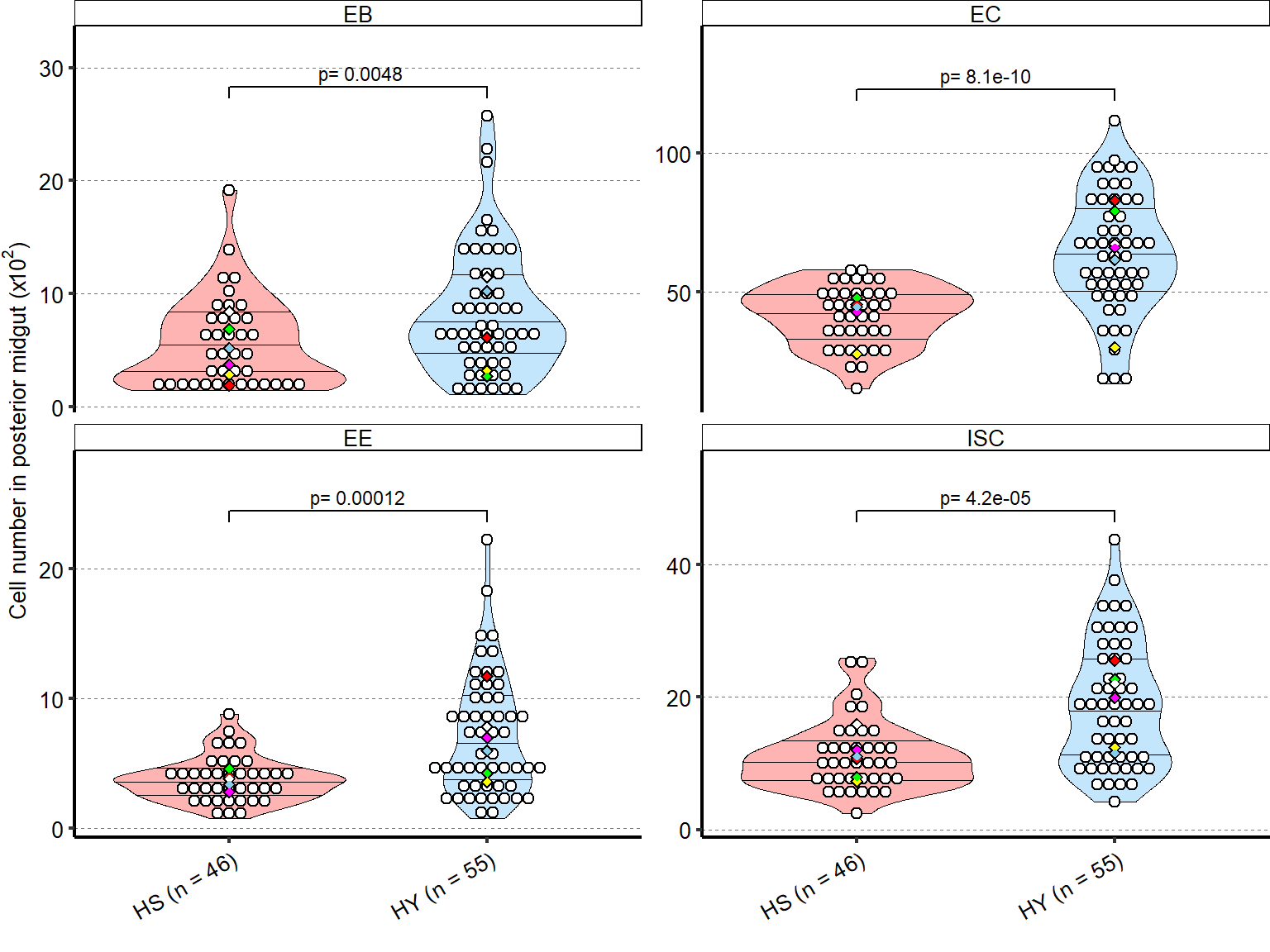

Supplement: Supplementary file 2. [file elife-64125-supp2.zip › Bonfini_script_GutPlasticity_diet_files/figure-html/Figure 3C-1.png]

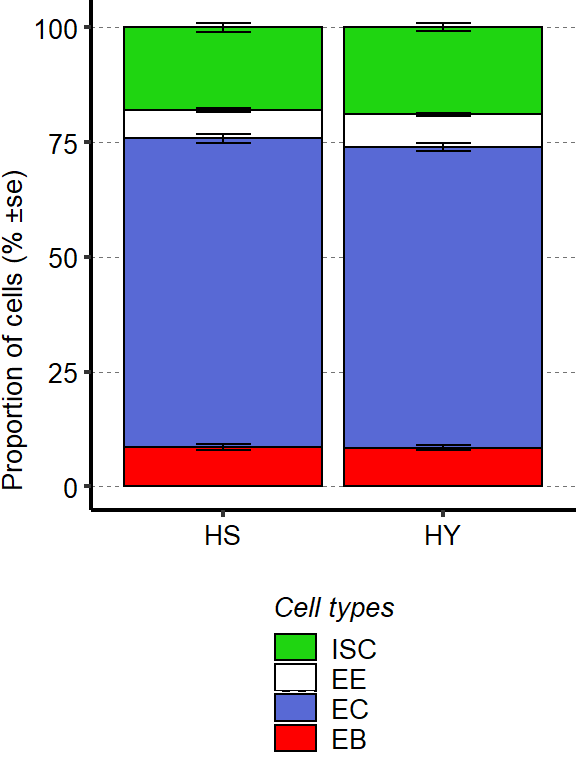

Supplement: Supplementary file 2. [file elife-64125-supp2.zip › Bonfini_script_GutPlasticity_diet_files/figure-html/Figure 3D-1.png]

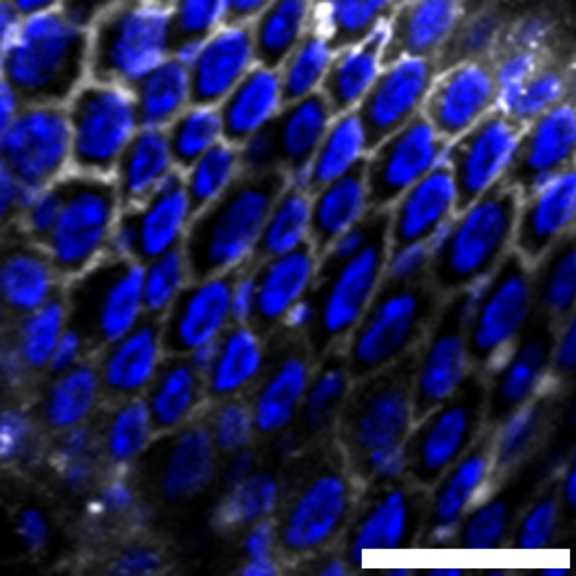

Supplement: Supplementary file 2. [file elife-64125-supp2.zip › Bonfini_script_GutPlasticity_diet_files/figure-html/Figure 3E-1.png]

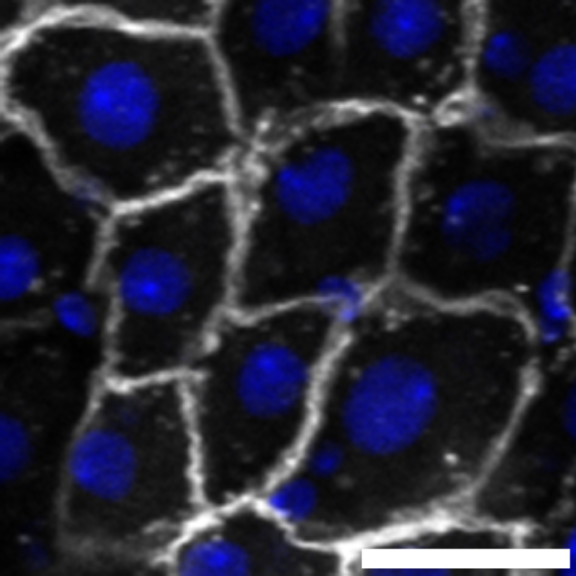

Supplement: Supplementary file 2. [file elife-64125-supp2.zip › Bonfini_script_GutPlasticity_diet_files/figure-html/Figure 3F-1.png]

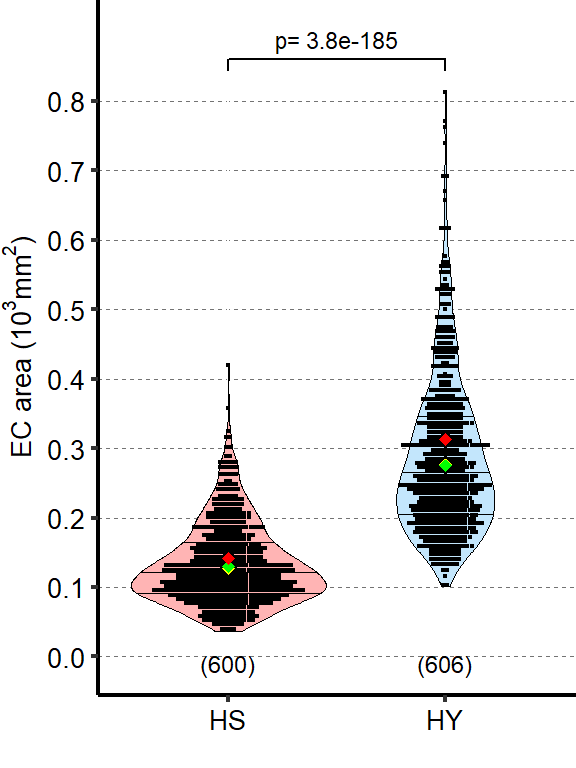

Supplement: Supplementary file 2. [file elife-64125-supp2.zip › Bonfini_script_GutPlasticity_diet_files/figure-html/Figure 3G-1.png]

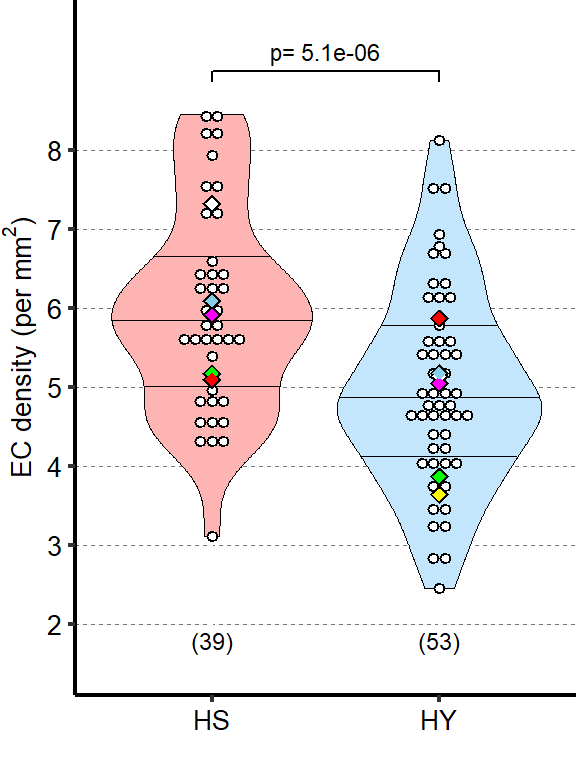

Supplement: Supplementary file 2. [file elife-64125-supp2.zip › Bonfini_script_GutPlasticity_diet_files/figure-html/Figure 3S1A-1.png]

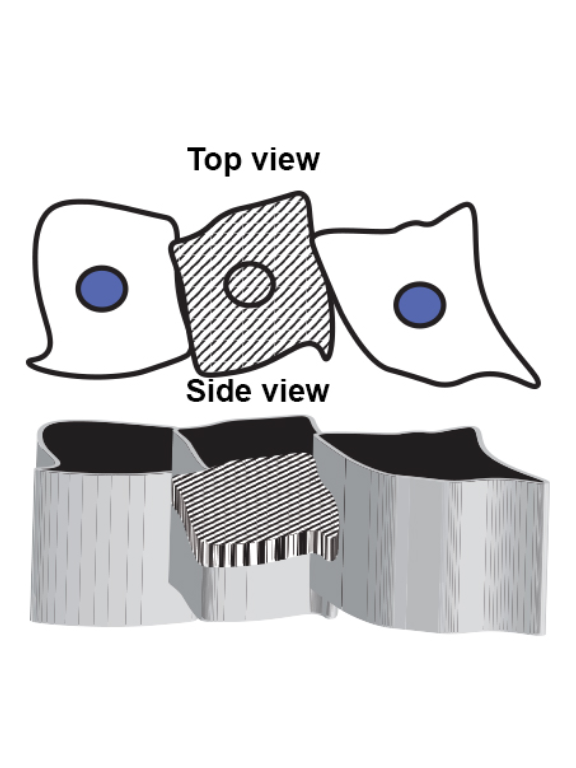

Supplement: Supplementary file 2. [file elife-64125-supp2.zip › Bonfini_script_GutPlasticity_diet_files/figure-html/Figure 3S1B-1.png]

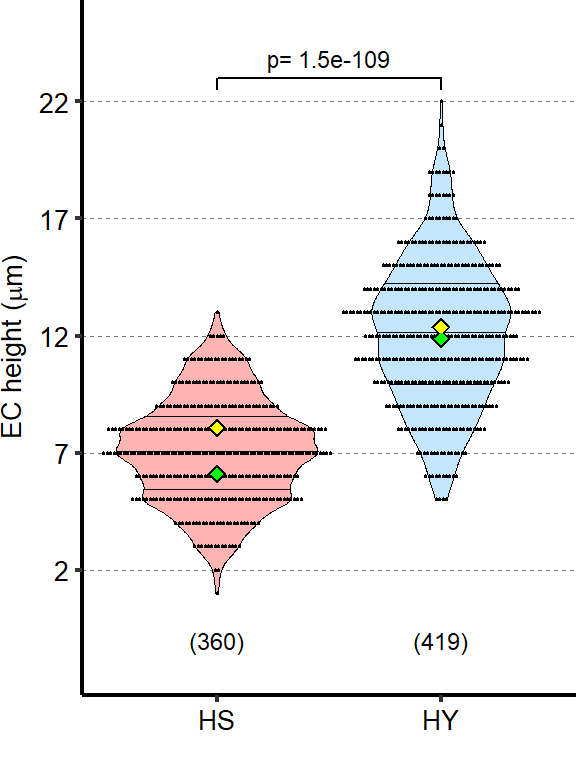

Supplement: Supplementary file 2. [file elife-64125-supp2.zip › Bonfini_script_GutPlasticity_diet_files/figure-html/Figure 3S1C-1.png]

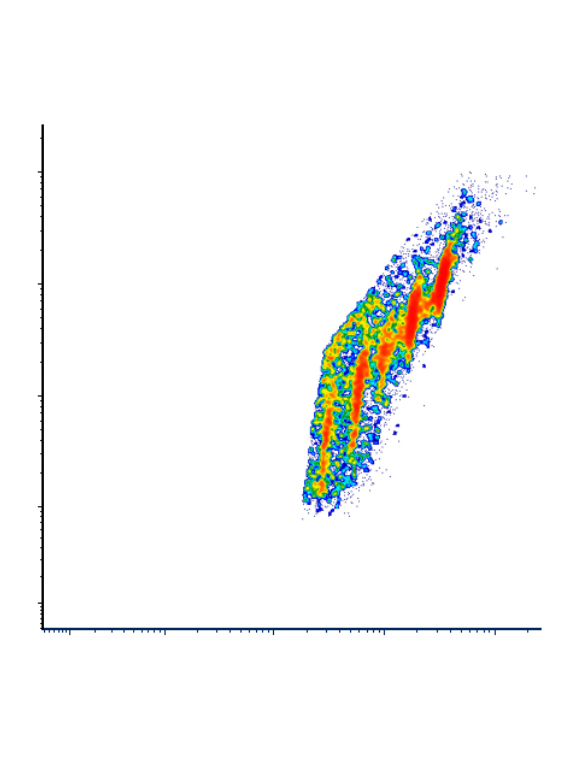

Supplement: Supplementary file 2. [file elife-64125-supp2.zip › Bonfini_script_GutPlasticity_diet_files/figure-html/Figure 3S1D-1.png]

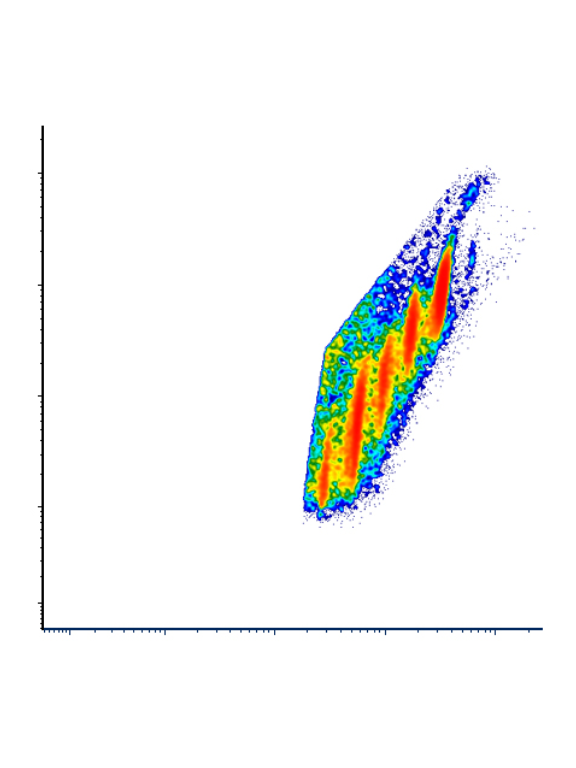

Supplement: Supplementary file 2. [file elife-64125-supp2.zip › Bonfini_script_GutPlasticity_diet_files/figure-html/Figure 3S1E-1.png]

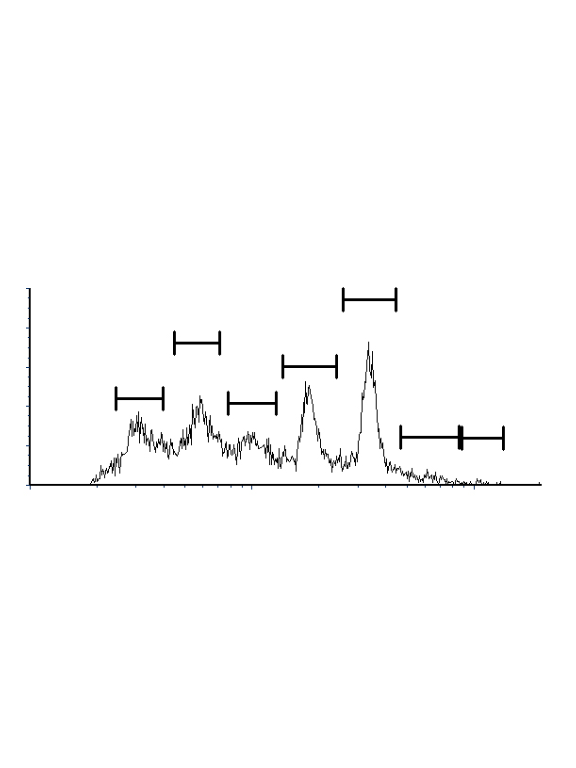

Supplement: Supplementary file 2. [file elife-64125-supp2.zip › Bonfini_script_GutPlasticity_diet_files/figure-html/Figure 3S1F-1.png]

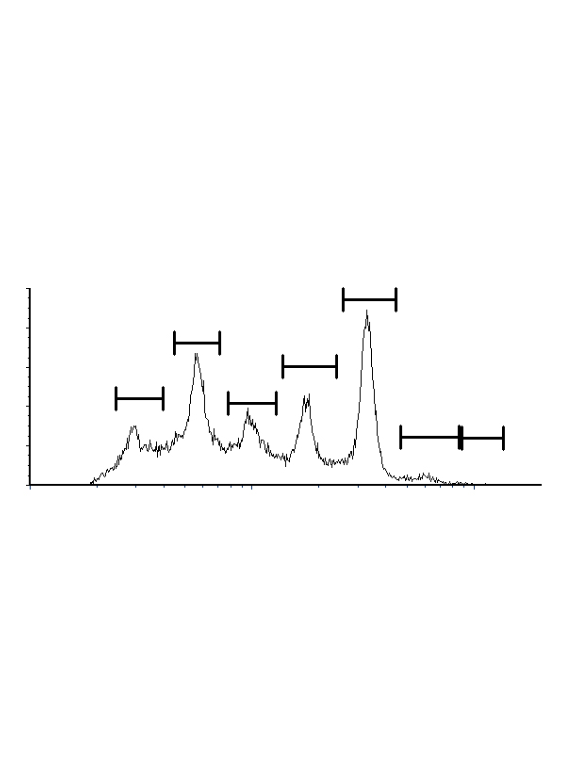

Supplement: Supplementary file 2. [file elife-64125-supp2.zip › Bonfini_script_GutPlasticity_diet_files/figure-html/Figure 3S1G-1.png]

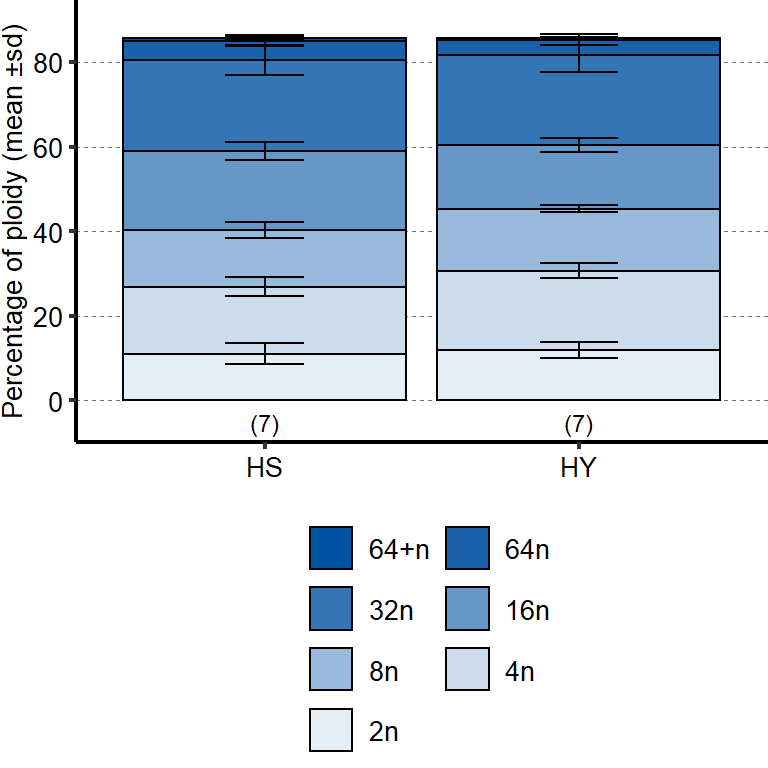

Supplement: Supplementary file 2. [file elife-64125-supp2.zip › Bonfini_script_GutPlasticity_diet_files/figure-html/Figure 3S1H-1.png]

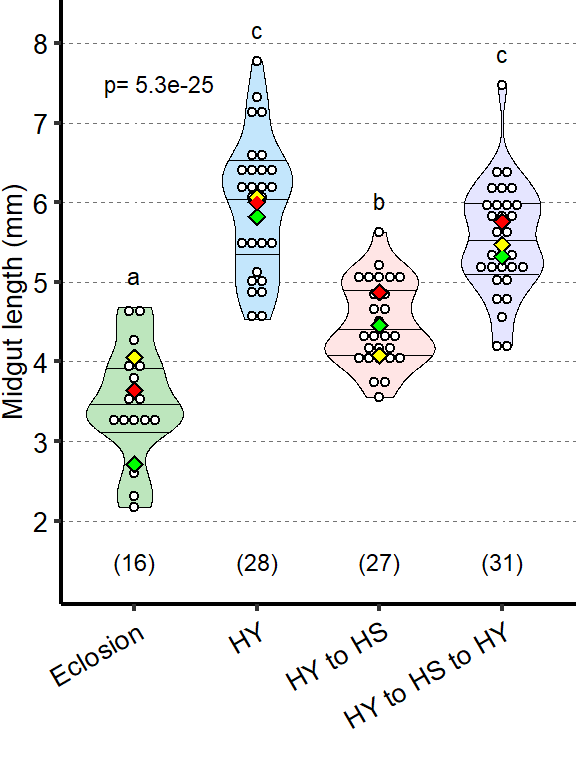

Supplement: Supplementary file 2. [file elife-64125-supp2.zip › Bonfini_script_GutPlasticity_diet_files/figure-html/Figure 4A-1.png]

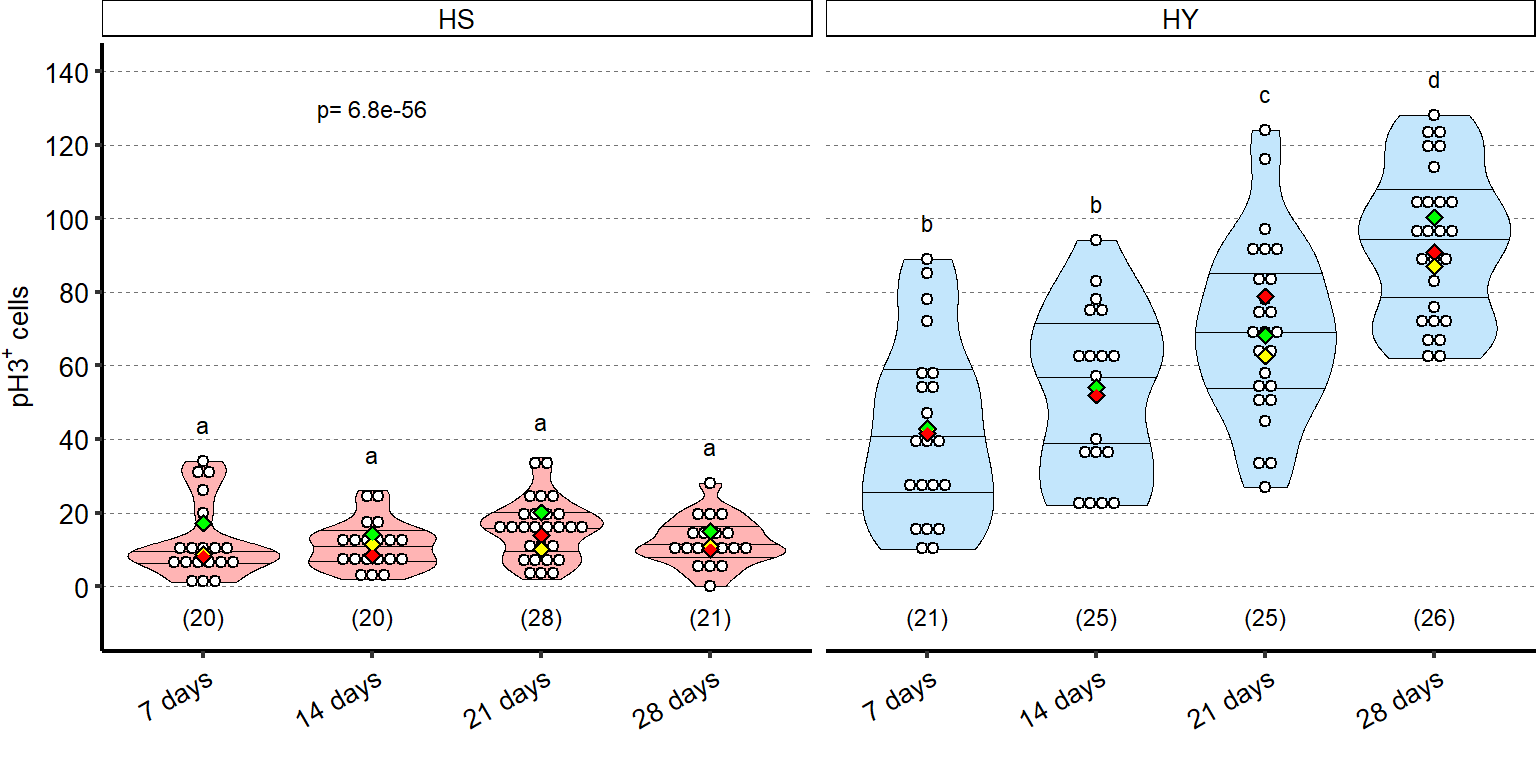

Supplement: Supplementary file 2. [file elife-64125-supp2.zip › Bonfini_script_GutPlasticity_diet_files/figure-html/Figure 4B-1.png]

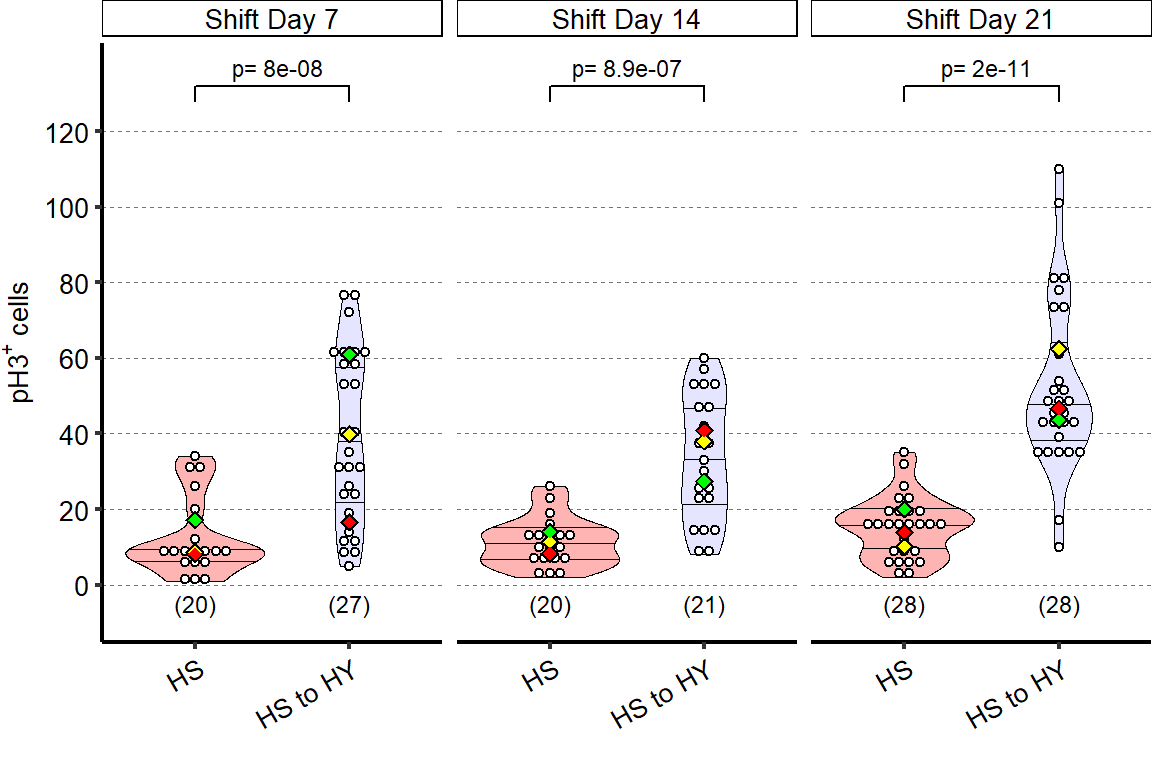

Supplement: Supplementary file 2. [file elife-64125-supp2.zip › Bonfini_script_GutPlasticity_diet_files/figure-html/Figure 4C-1.png]

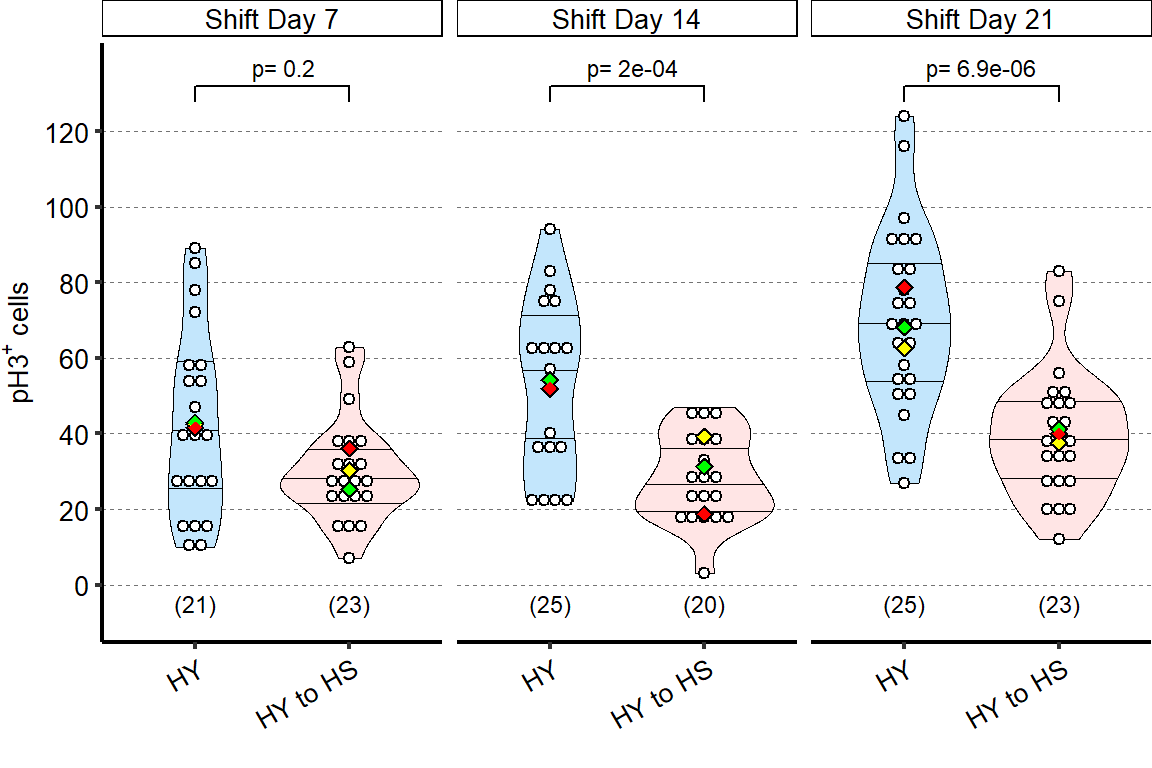

Supplement: Supplementary file 2. [file elife-64125-supp2.zip › Bonfini_script_GutPlasticity_diet_files/figure-html/Figure 4D-1.png]

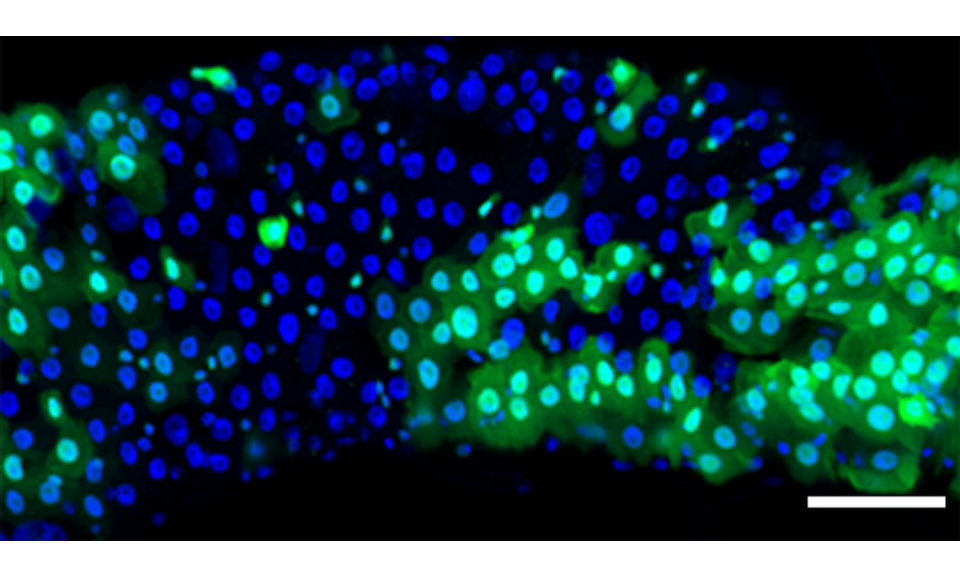

Supplement: Supplementary file 2. [file elife-64125-supp2.zip › Bonfini_script_GutPlasticity_diet_files/figure-html/Figure 4E-1.png]

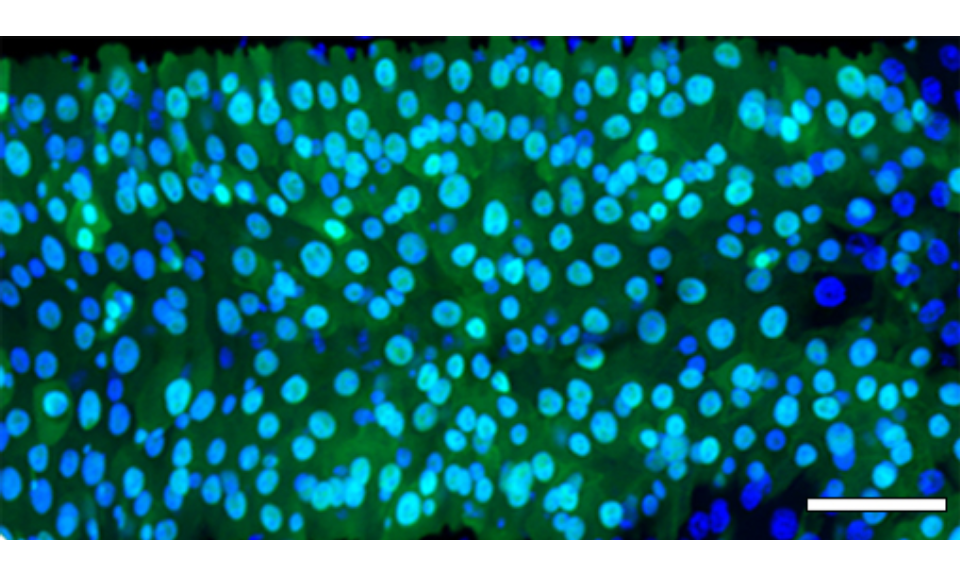

Supplement: Supplementary file 2. [file elife-64125-supp2.zip › Bonfini_script_GutPlasticity_diet_files/figure-html/Figure 4F-1.png]

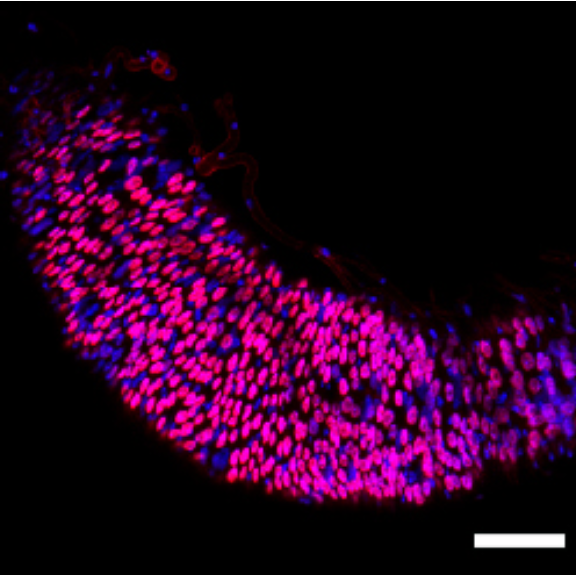

Supplement: Supplementary file 2. [file elife-64125-supp2.zip › Bonfini_script_GutPlasticity_diet_files/figure-html/Figure 4G-1.png]

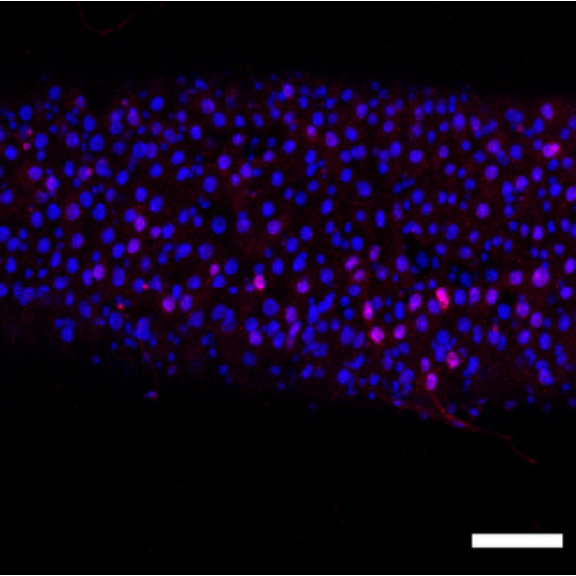

Supplement: Supplementary file 2. [file elife-64125-supp2.zip › Bonfini_script_GutPlasticity_diet_files/figure-html/Figure 4H-1.png]

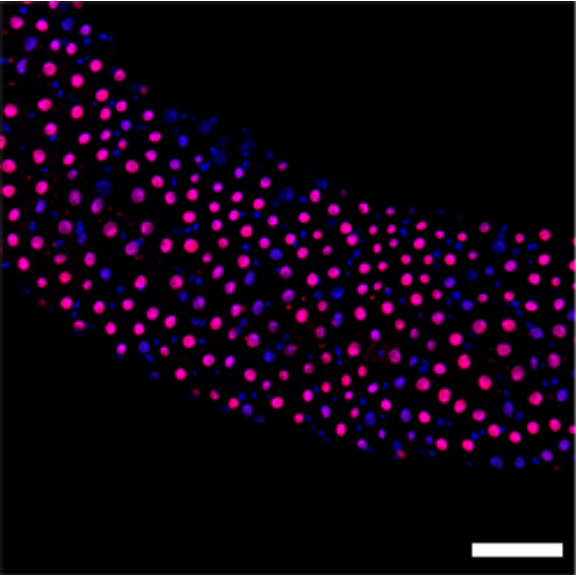

Supplement: Supplementary file 2. [file elife-64125-supp2.zip › Bonfini_script_GutPlasticity_diet_files/figure-html/Figure 4I-1.png]

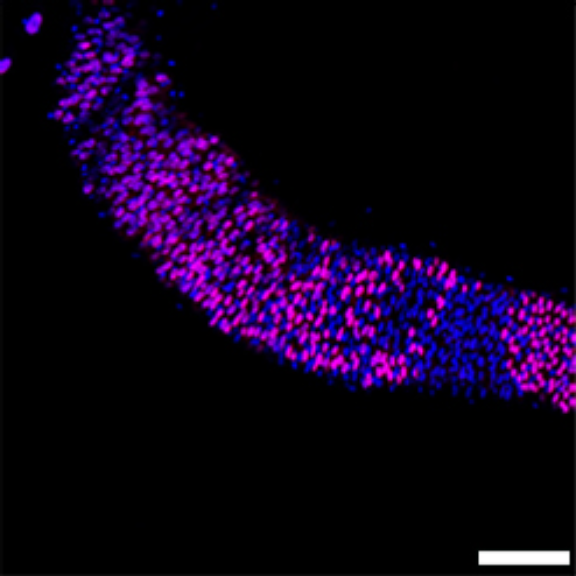

Supplement: Supplementary file 2. [file elife-64125-supp2.zip › Bonfini_script_GutPlasticity_diet_files/figure-html/Figure 4J-1.png]

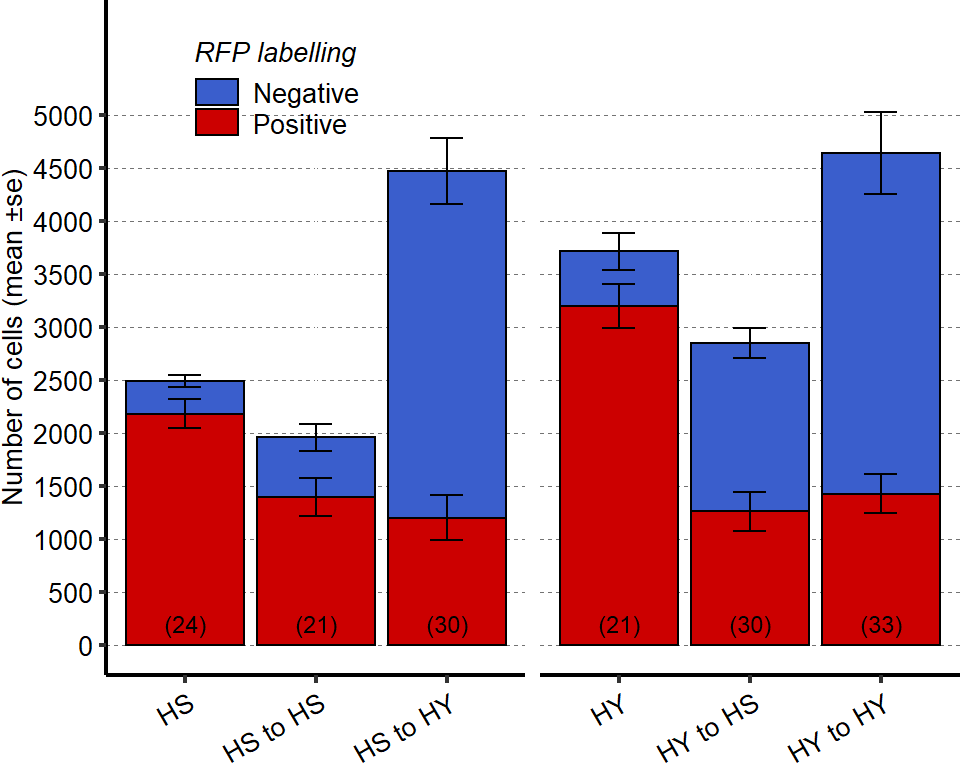

Supplement: Supplementary file 2. [file elife-64125-supp2.zip › Bonfini_script_GutPlasticity_diet_files/figure-html/Figure 4K-1.png]

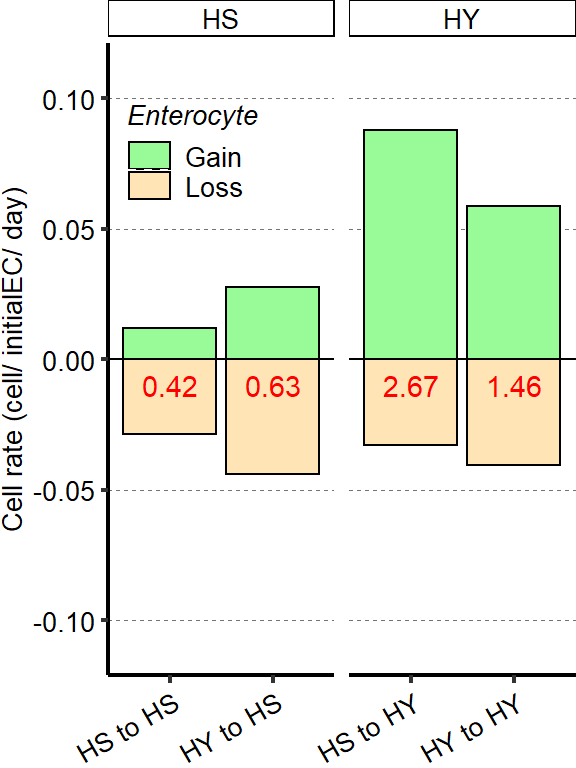

Supplement: Supplementary file 2. [file elife-64125-supp2.zip › Bonfini_script_GutPlasticity_diet_files/figure-html/Figure 4L-1.png]

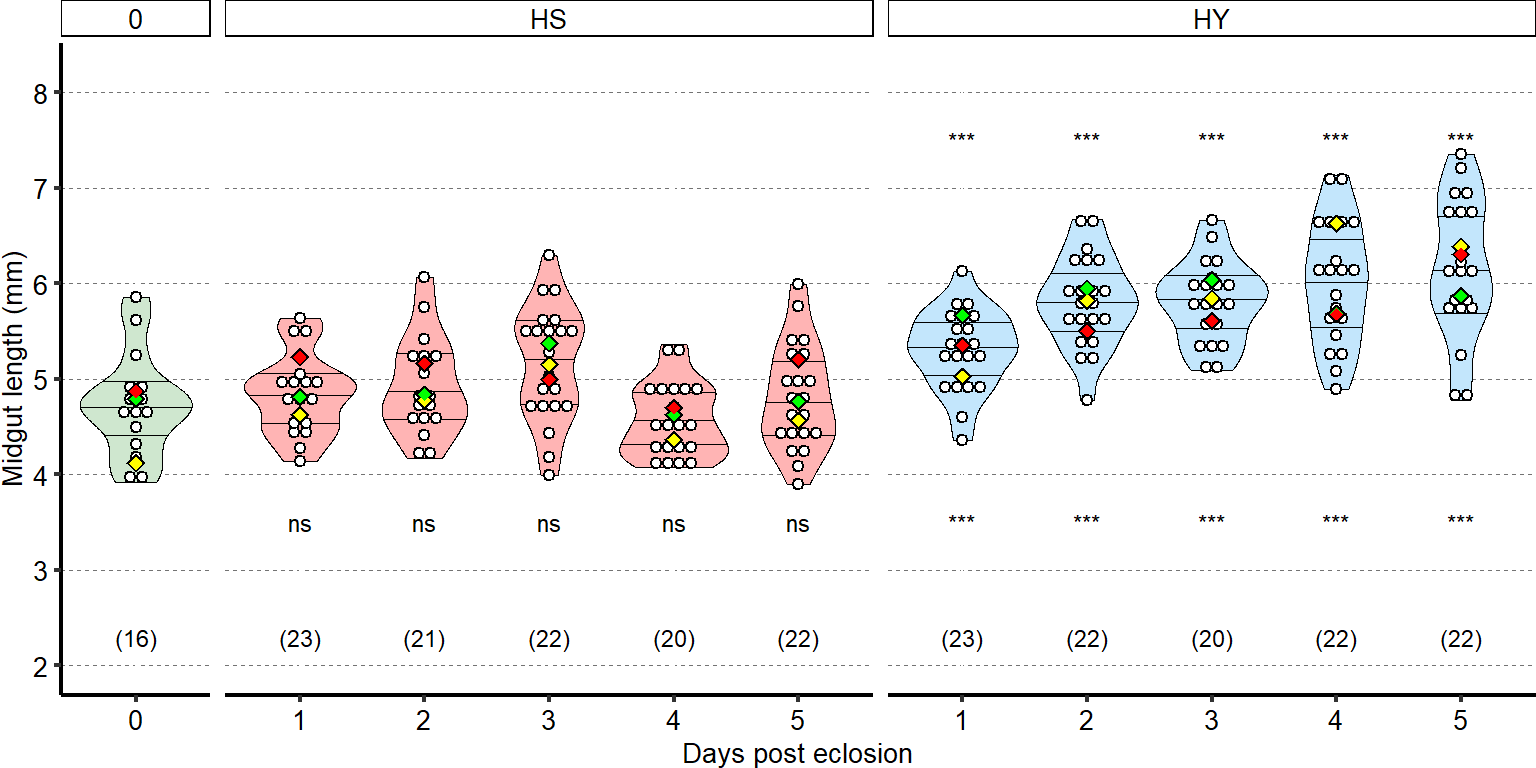

Supplement: Supplementary file 2. [file elife-64125-supp2.zip › Bonfini_script_GutPlasticity_diet_files/figure-html/Figure 4S1A-1.png]

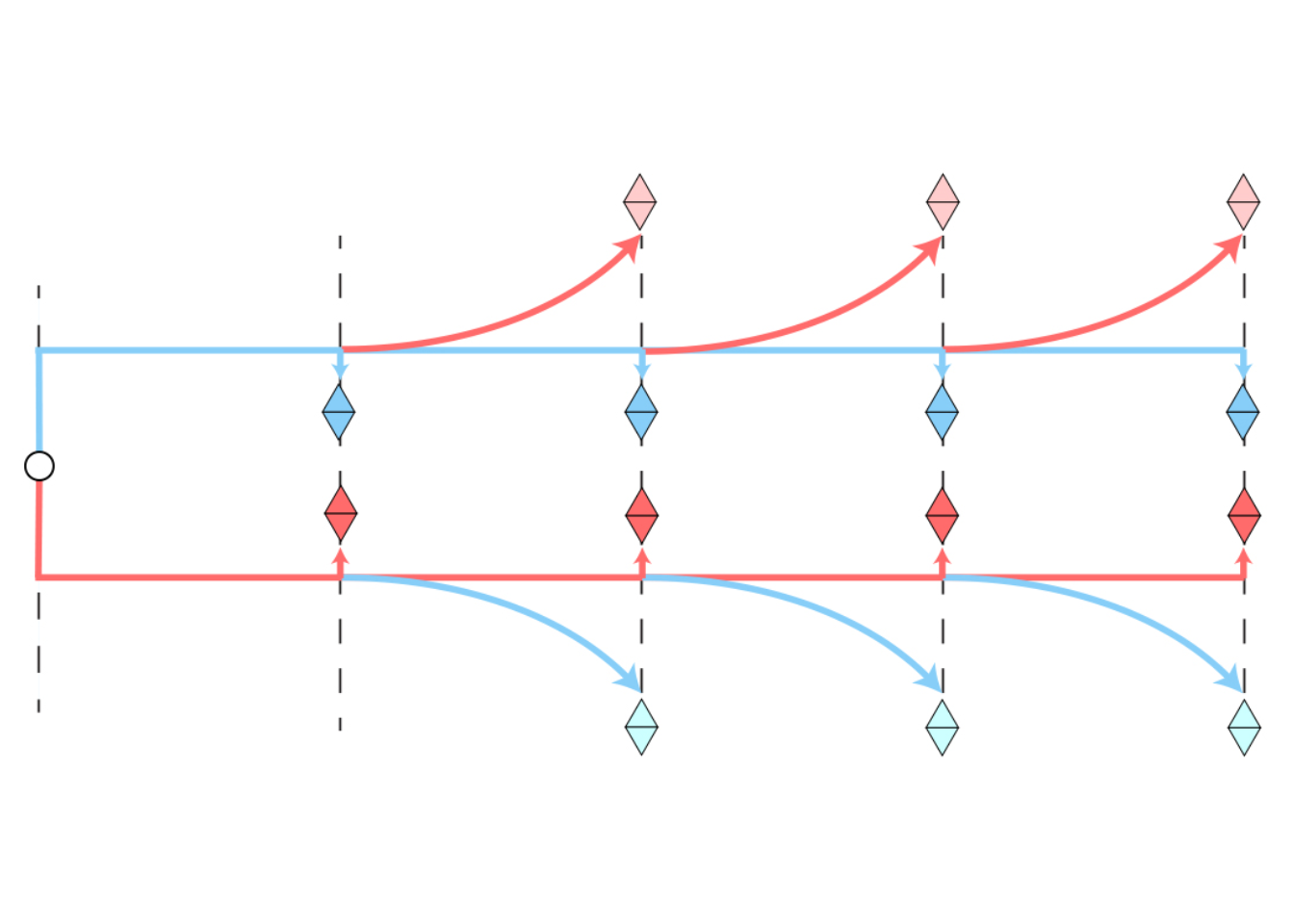

Supplement: Supplementary file 2. [file elife-64125-supp2.zip › Bonfini_script_GutPlasticity_diet_files/figure-html/Figure 4S1B-1.png]

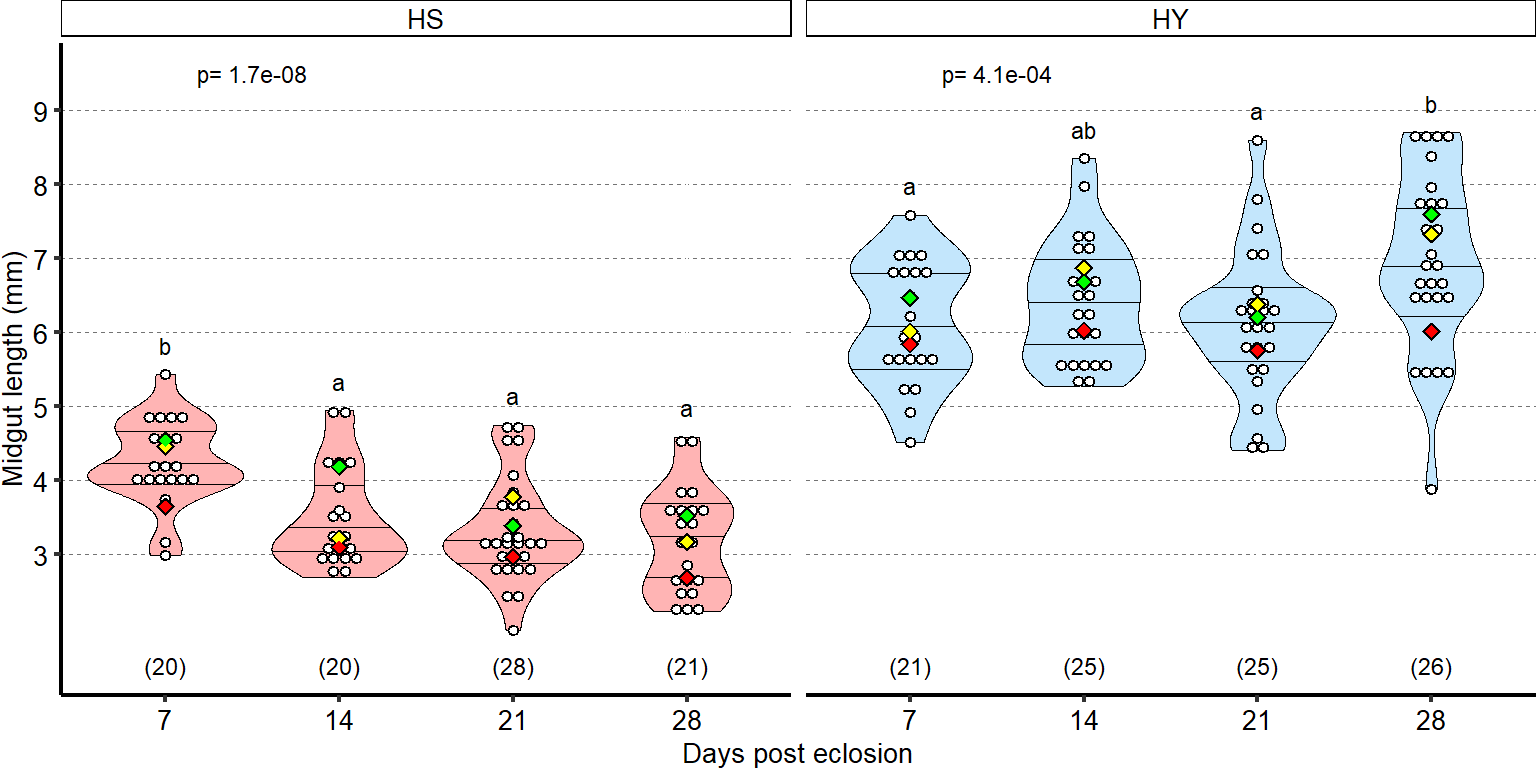

Supplement: Supplementary file 2. [file elife-64125-supp2.zip › Bonfini_script_GutPlasticity_diet_files/figure-html/Figure 4S1C-1.png]

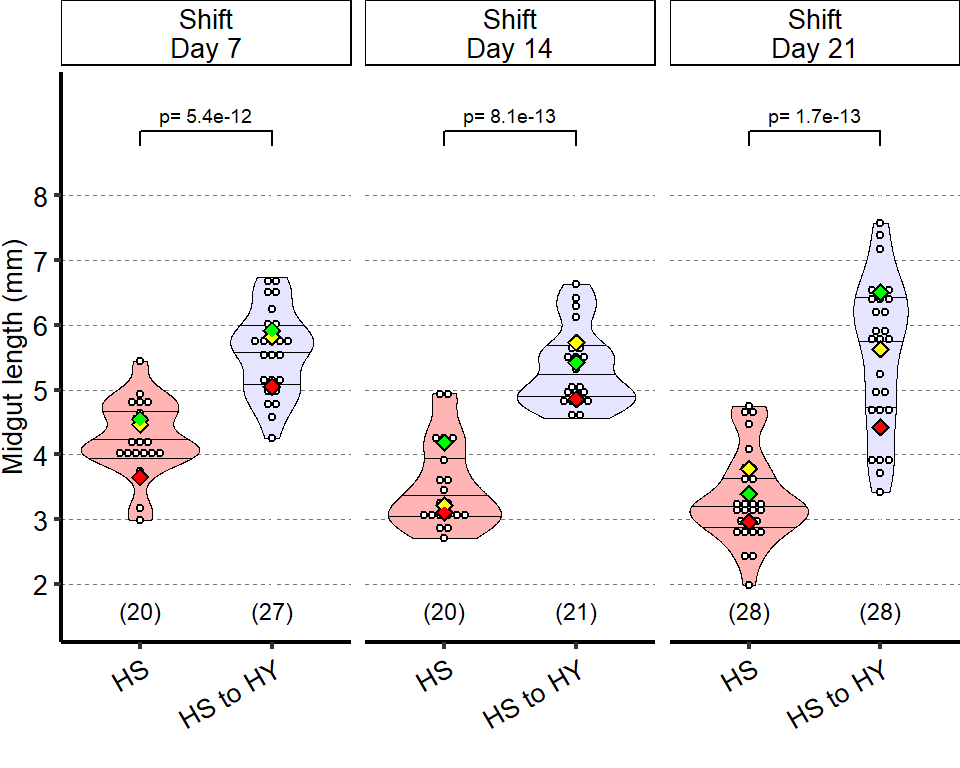

Supplement: Supplementary file 2. [file elife-64125-supp2.zip › Bonfini_script_GutPlasticity_diet_files/figure-html/Figure 4S1D-1.png]

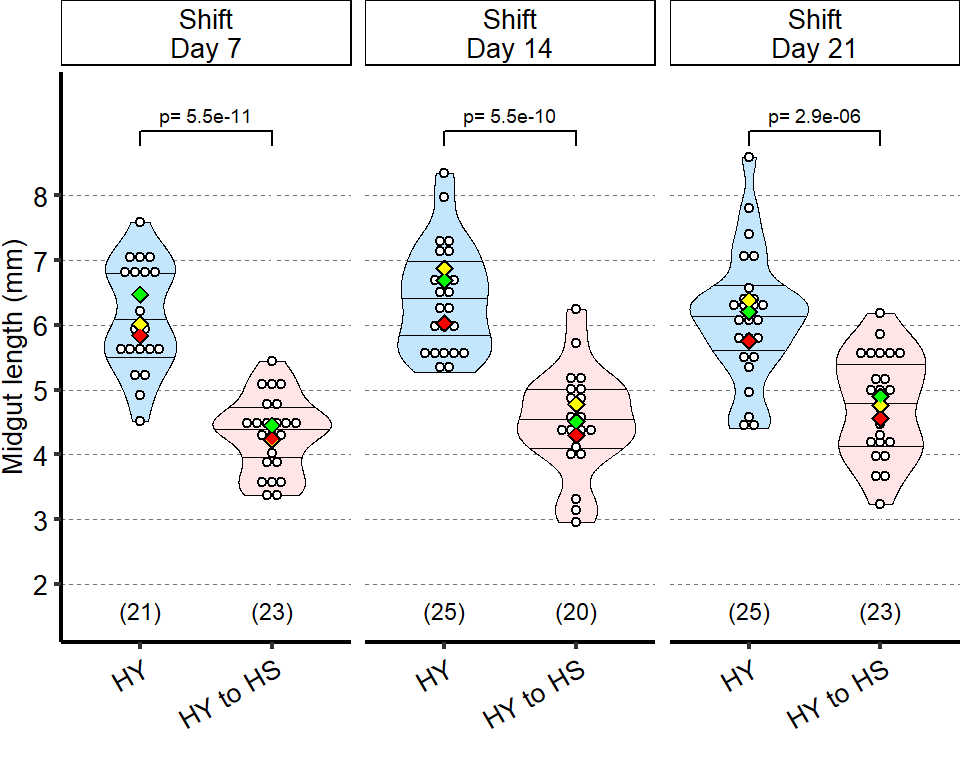

Supplement: Supplementary file 2. [file elife-64125-supp2.zip › Bonfini_script_GutPlasticity_diet_files/figure-html/Figure 4S1E-1.png]

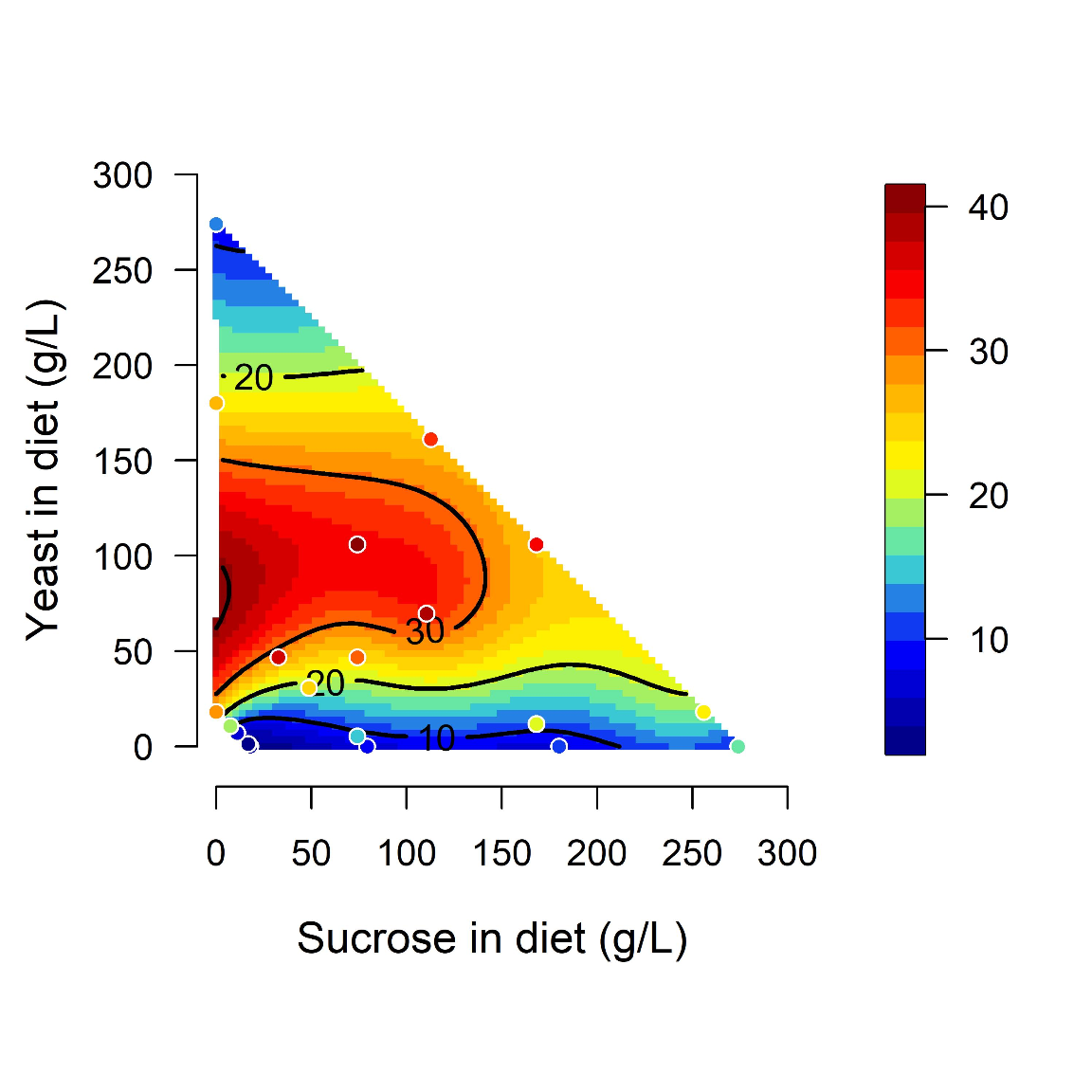

Supplement: Supplementary file 2. [file elife-64125-supp2.zip › Bonfini_script_GutPlasticity_diet_files/figure-html/Figure 4S1F-1.png]

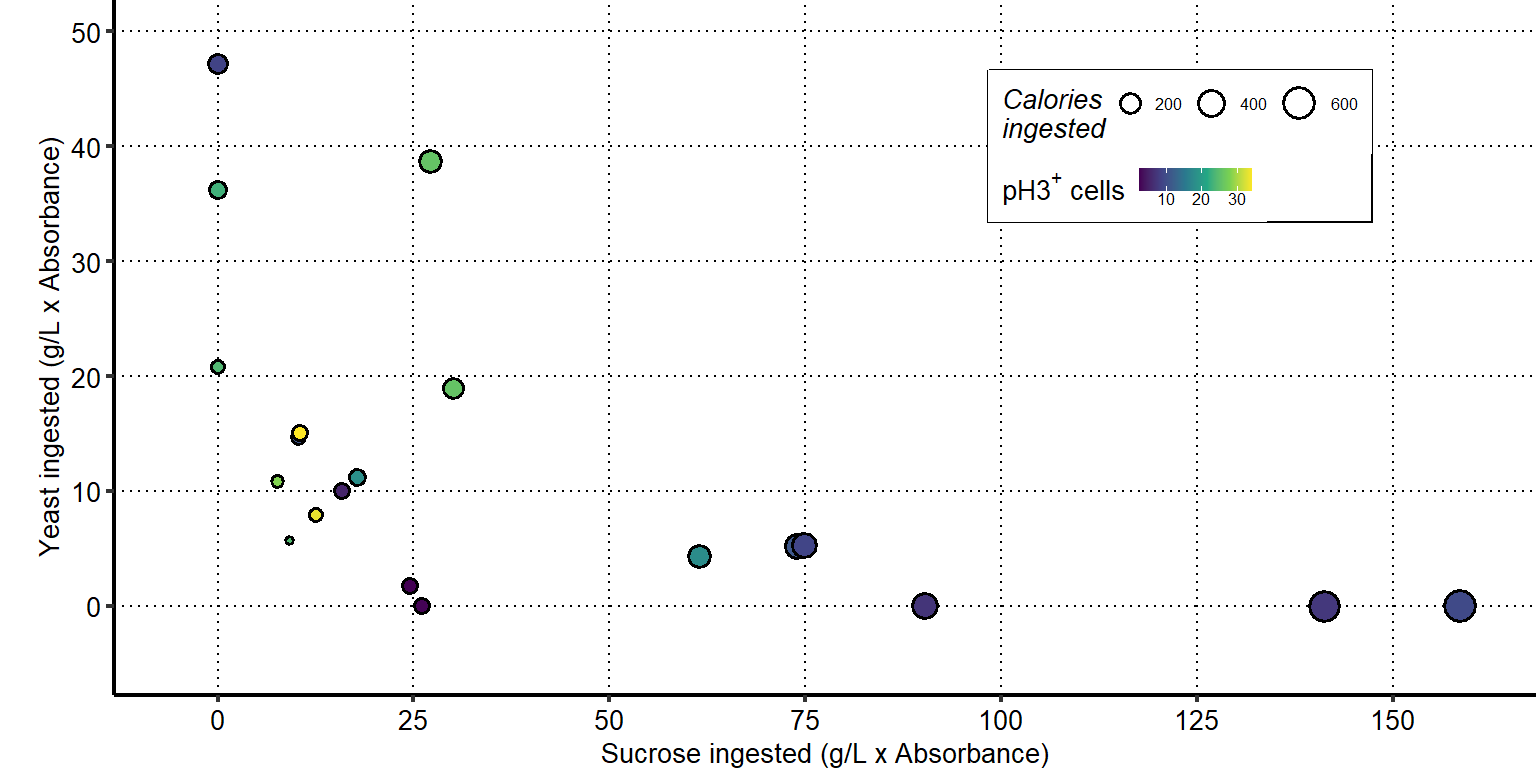

Supplement: Supplementary file 2. [file elife-64125-supp2.zip › Bonfini_script_GutPlasticity_diet_files/figure-html/Figure 4S1G-1.png]

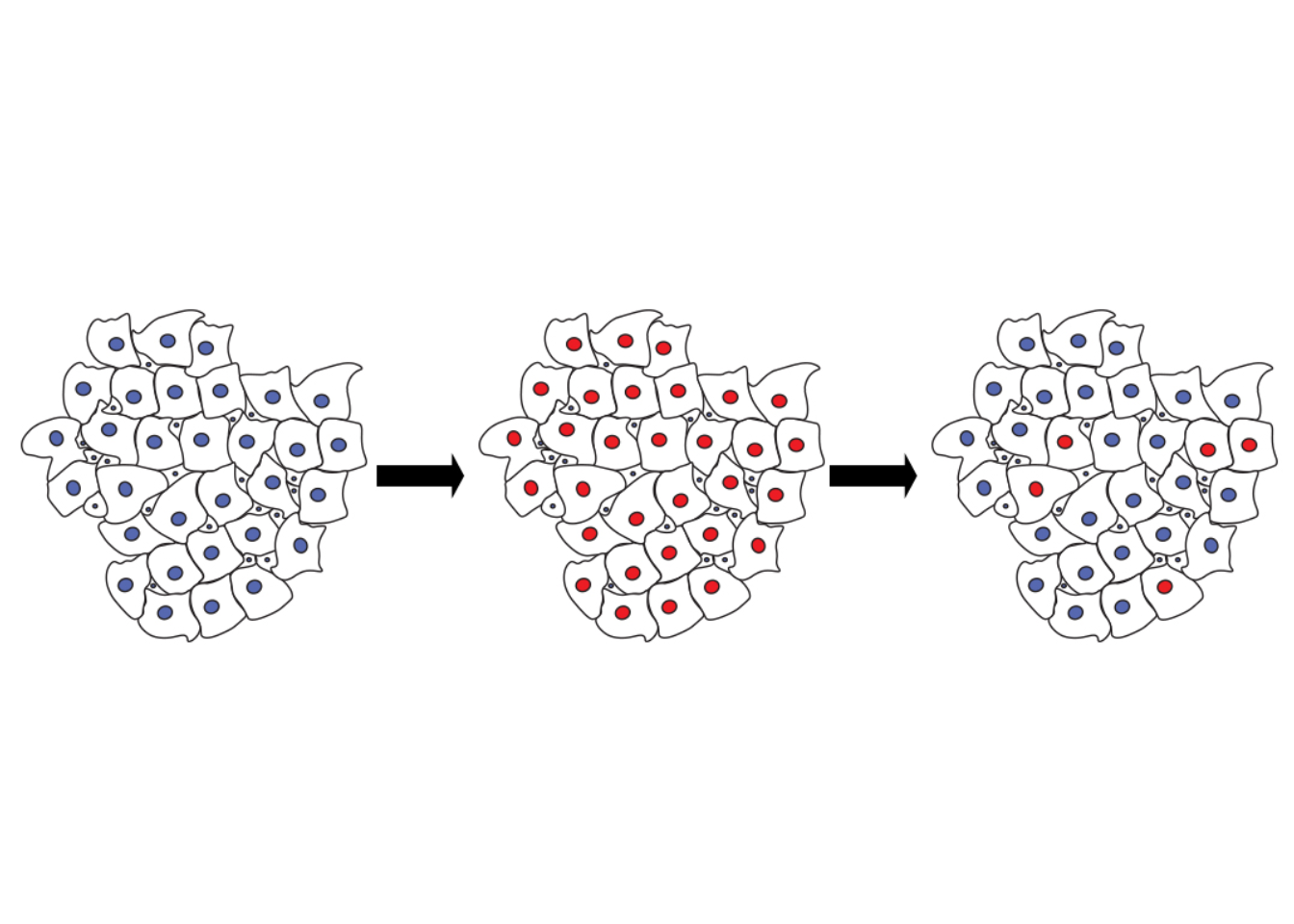

Supplement: Supplementary file 2. [file elife-64125-supp2.zip › Bonfini_script_GutPlasticity_diet_files/figure-html/Figure 4S2A-1.png]

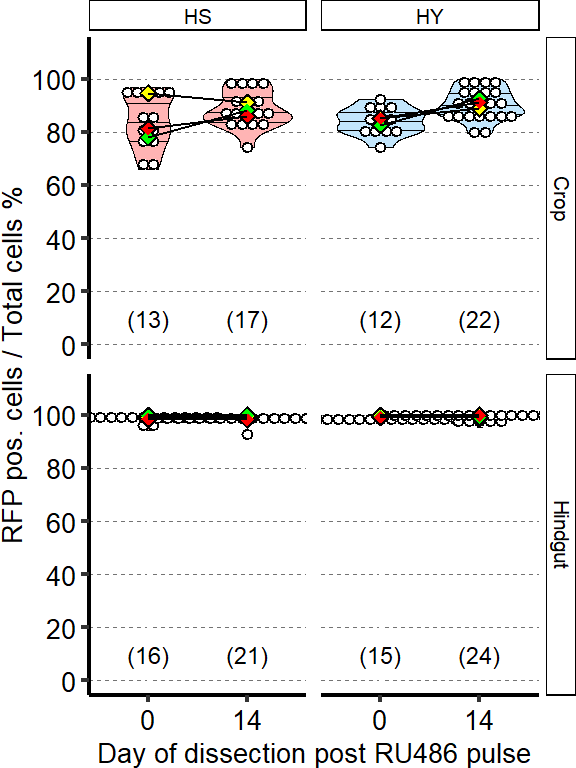

Supplement: Supplementary file 2. [file elife-64125-supp2.zip › Bonfini_script_GutPlasticity_diet_files/figure-html/Figure 4S2B-1.png]

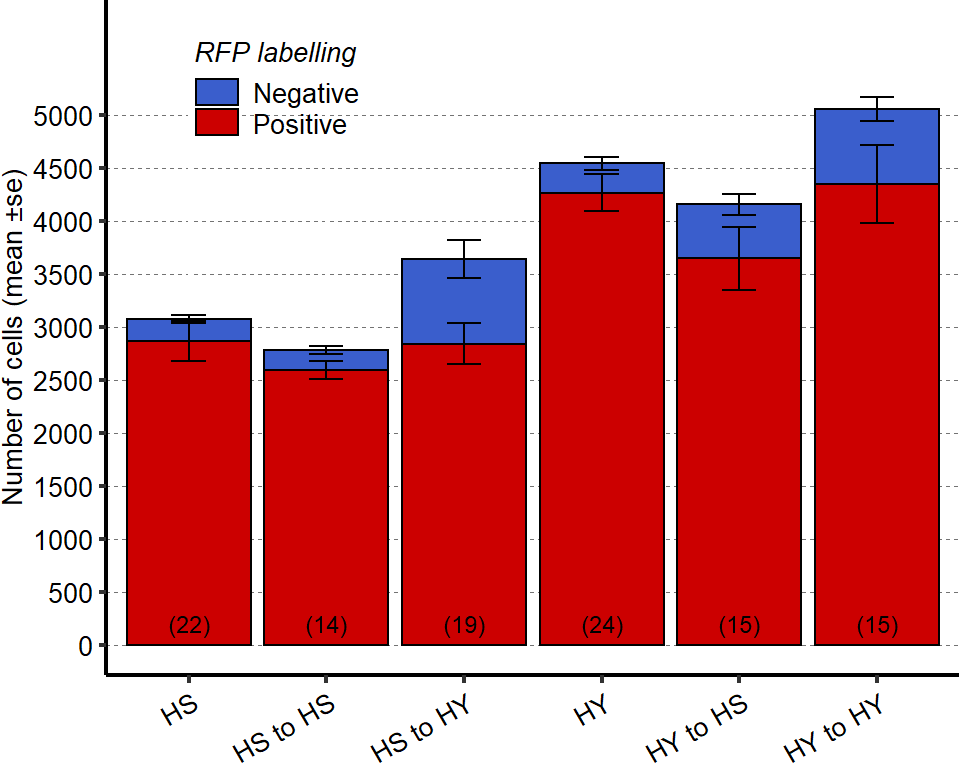

Supplement: Supplementary file 2. [file elife-64125-supp2.zip › Bonfini_script_GutPlasticity_diet_files/figure-html/Figure 4S2C-1.png]

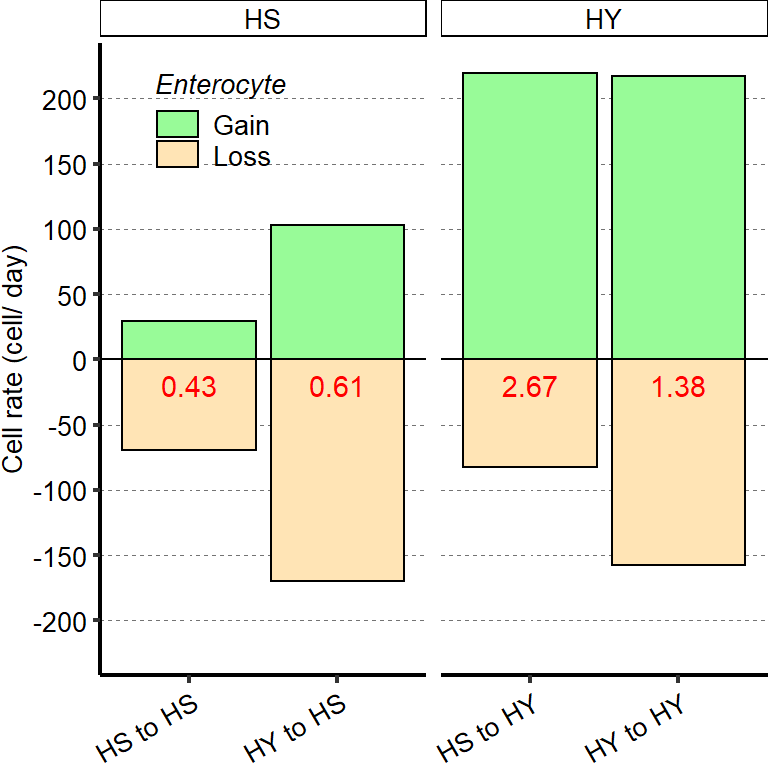

Supplement: Supplementary file 2. [file elife-64125-supp2.zip › Bonfini_script_GutPlasticity_diet_files/figure-html/Figure 4S2D-1.png]

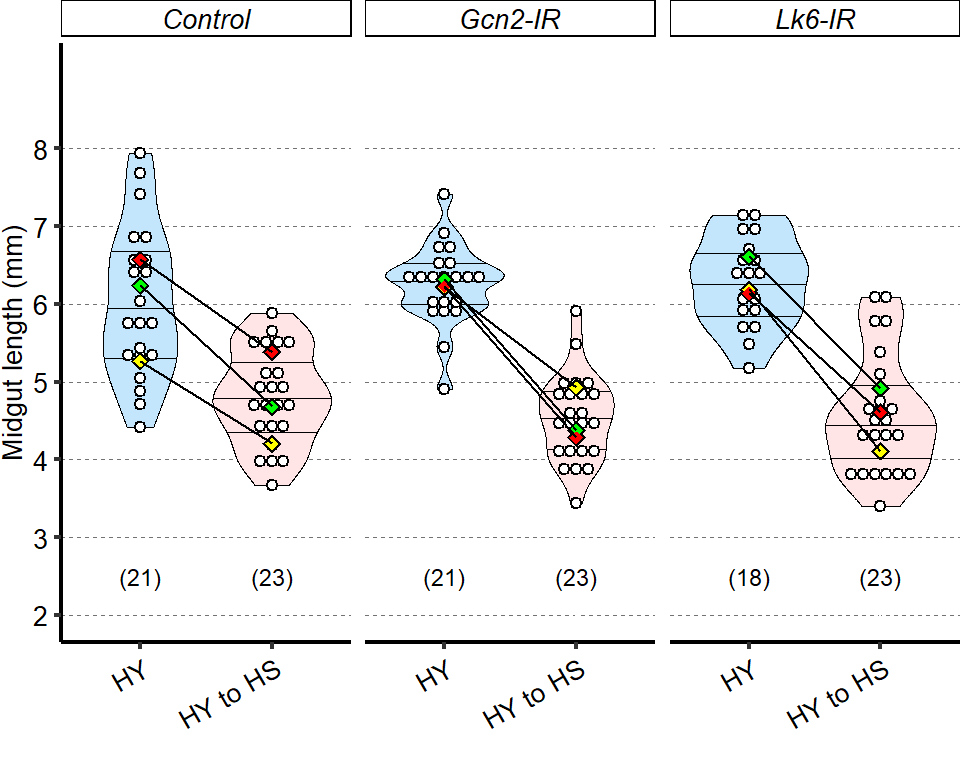

Supplement: Supplementary file 2. [file elife-64125-supp2.zip › Bonfini_script_GutPlasticity_diet_files/figure-html/Figure 55S2F-1.png]

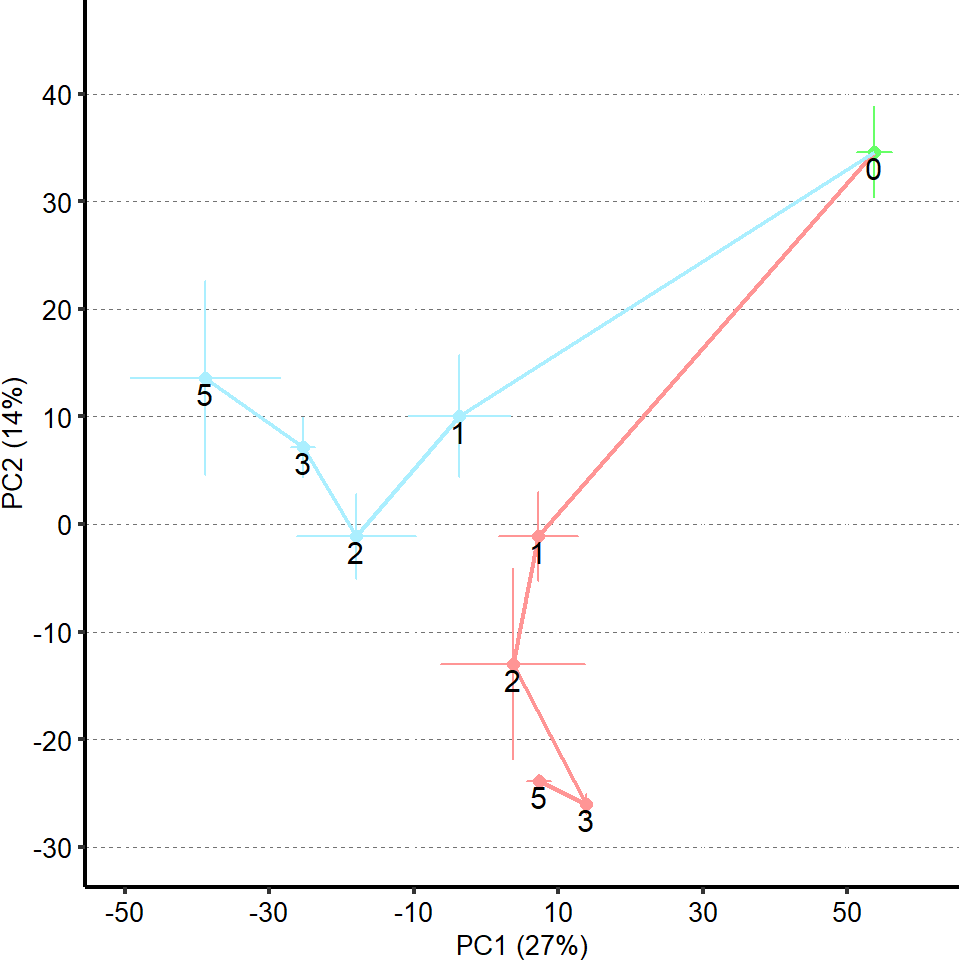

Supplement: Supplementary file 2. [file elife-64125-supp2.zip › Bonfini_script_GutPlasticity_diet_files/figure-html/Figure 5A-1.png]

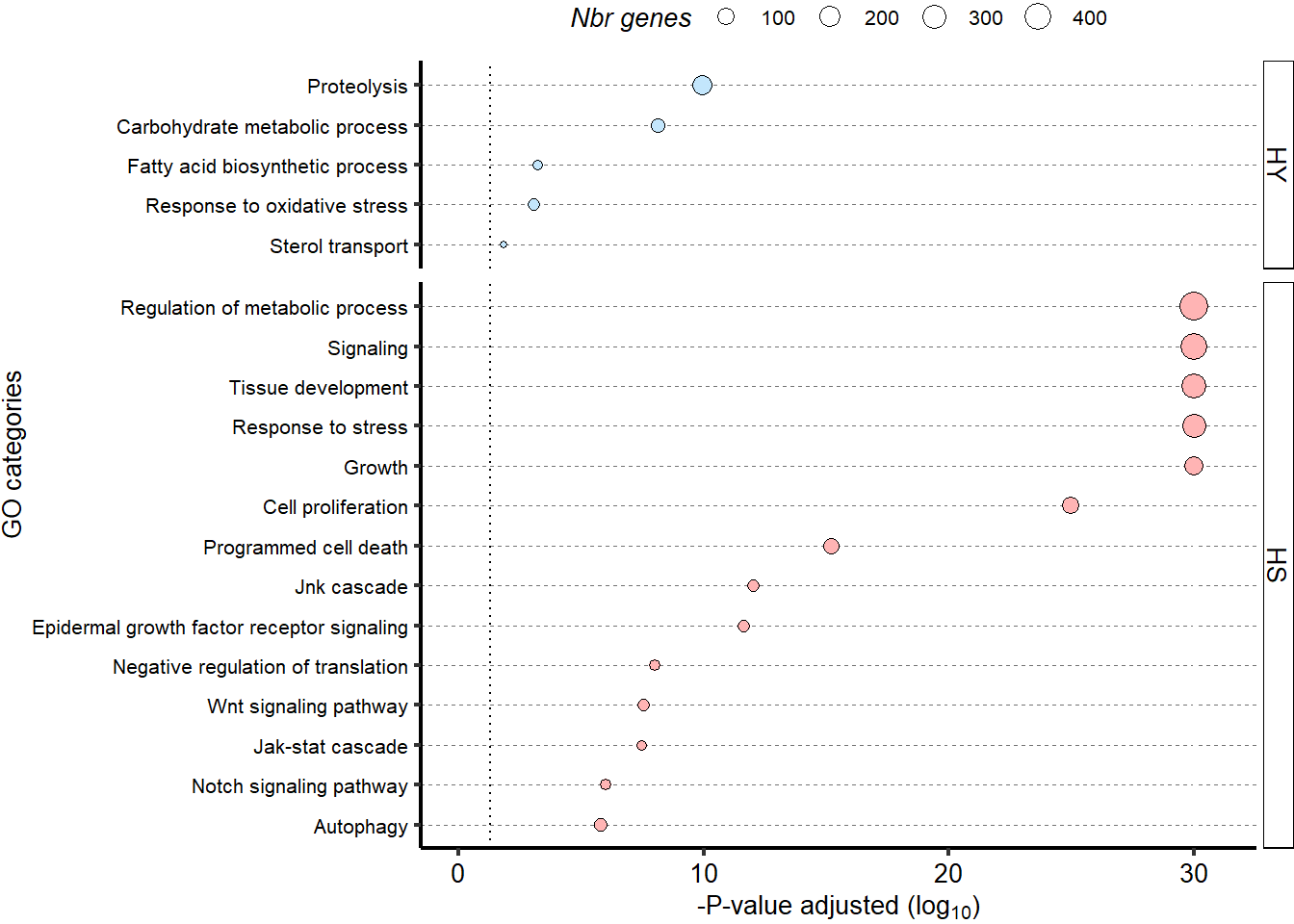

Supplement: Supplementary file 2. [file elife-64125-supp2.zip › Bonfini_script_GutPlasticity_diet_files/figure-html/Figure 5B-1.png]

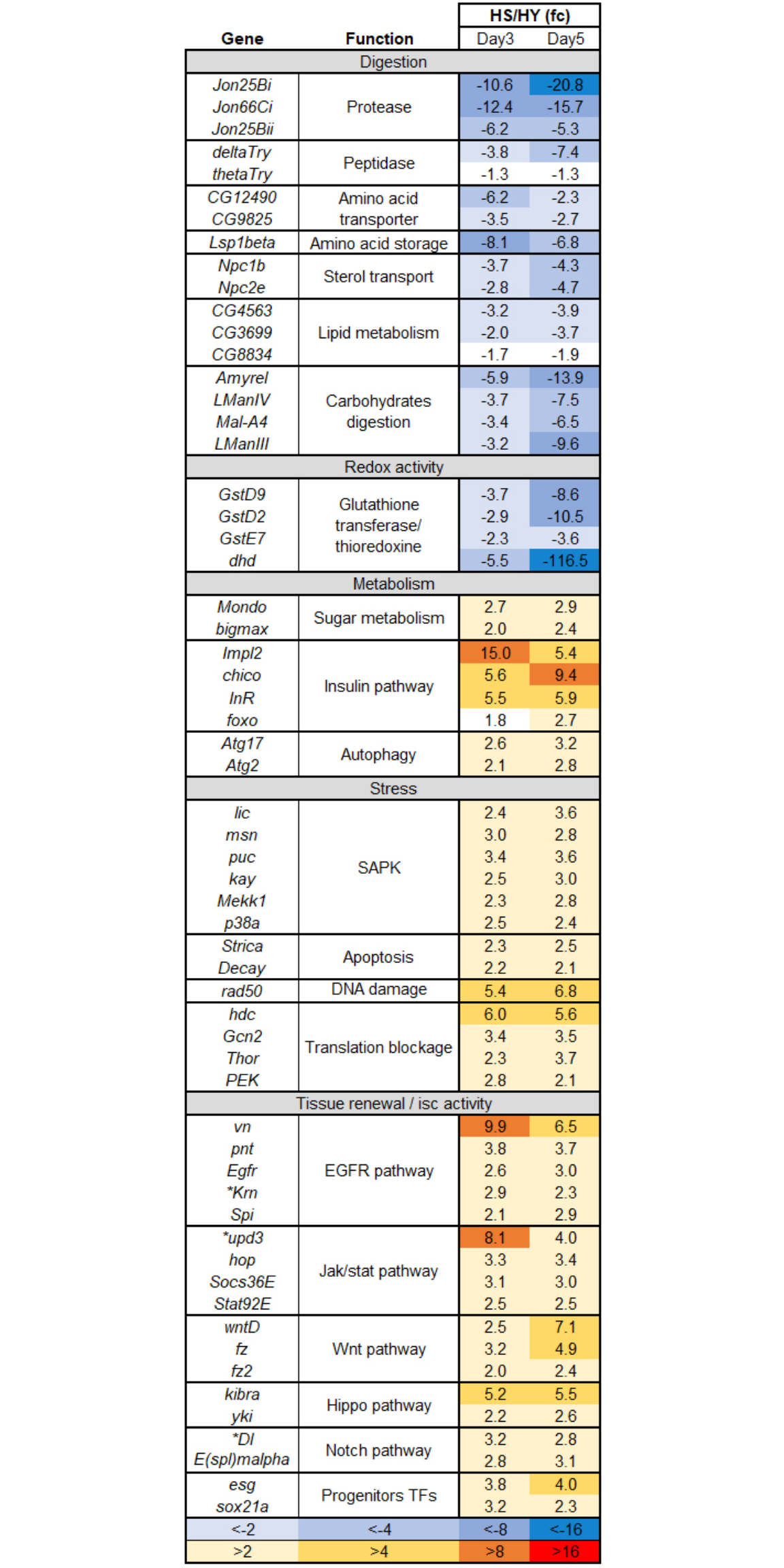

Supplement: Supplementary file 2. [file elife-64125-supp2.zip › Bonfini_script_GutPlasticity_diet_files/figure-html/Figure 5C-1.png]

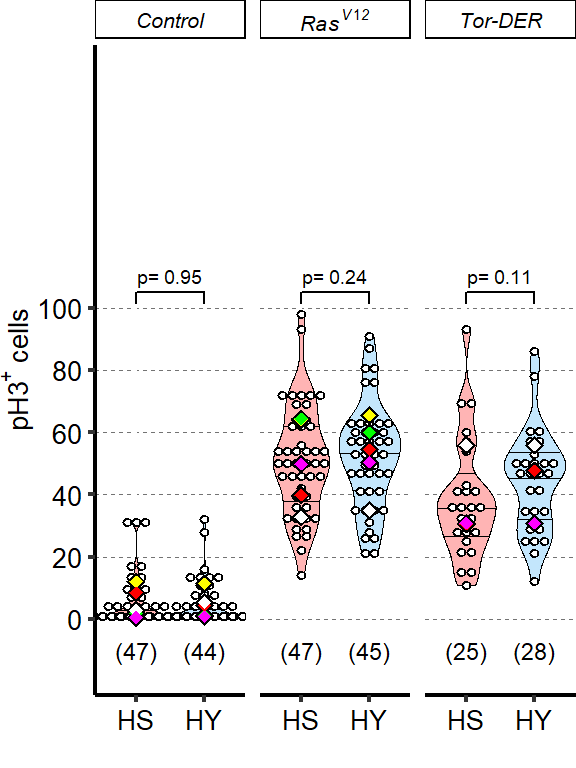

Supplement: Supplementary file 2. [file elife-64125-supp2.zip › Bonfini_script_GutPlasticity_diet_files/figure-html/Figure 5D-1.png]

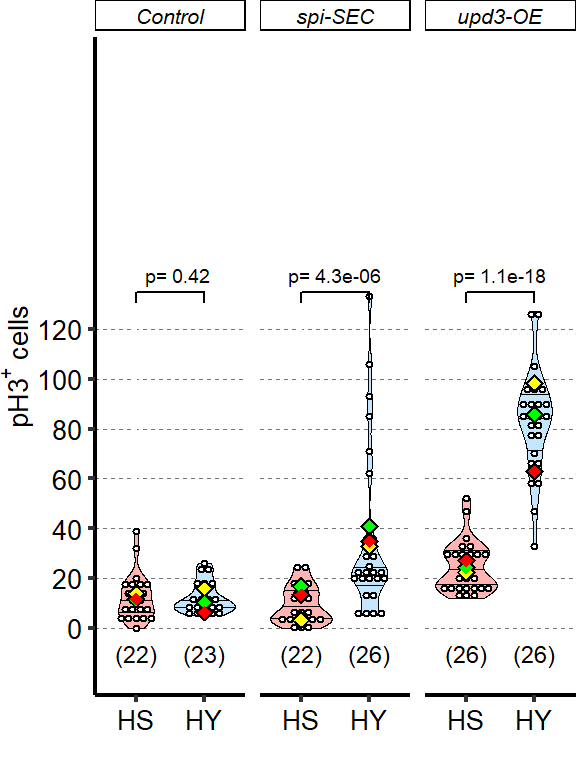

Supplement: Supplementary file 2. [file elife-64125-supp2.zip › Bonfini_script_GutPlasticity_diet_files/figure-html/Figure 5E-1.png]

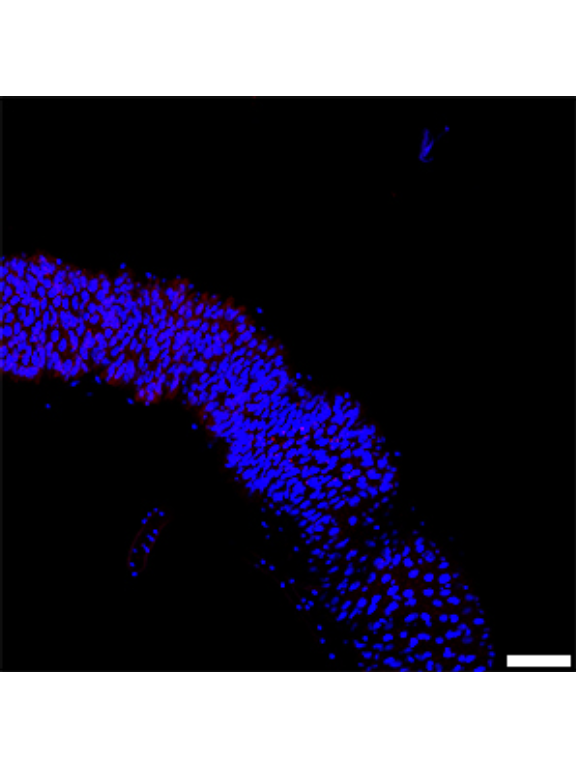

Supplement: Supplementary file 2. [file elife-64125-supp2.zip › Bonfini_script_GutPlasticity_diet_files/figure-html/Figure 5F1-1.png]

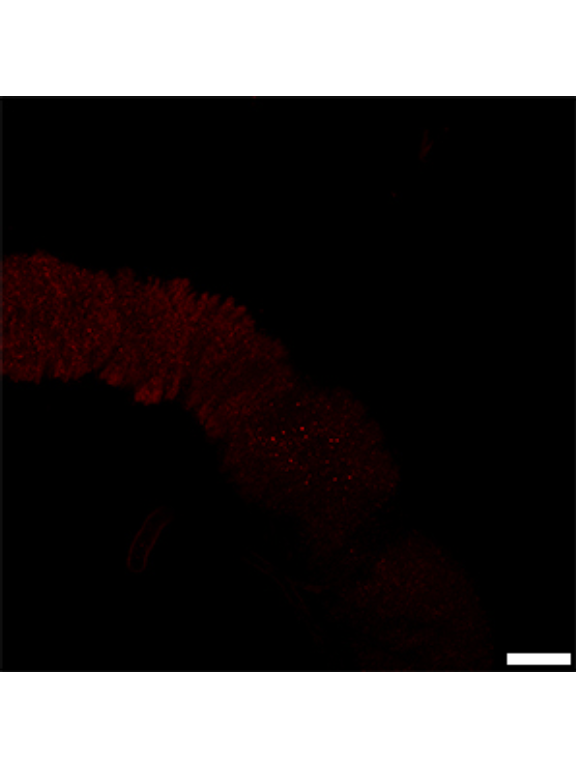

Supplement: Supplementary file 2. [file elife-64125-supp2.zip › Bonfini_script_GutPlasticity_diet_files/figure-html/Figure 5F2-1.png]

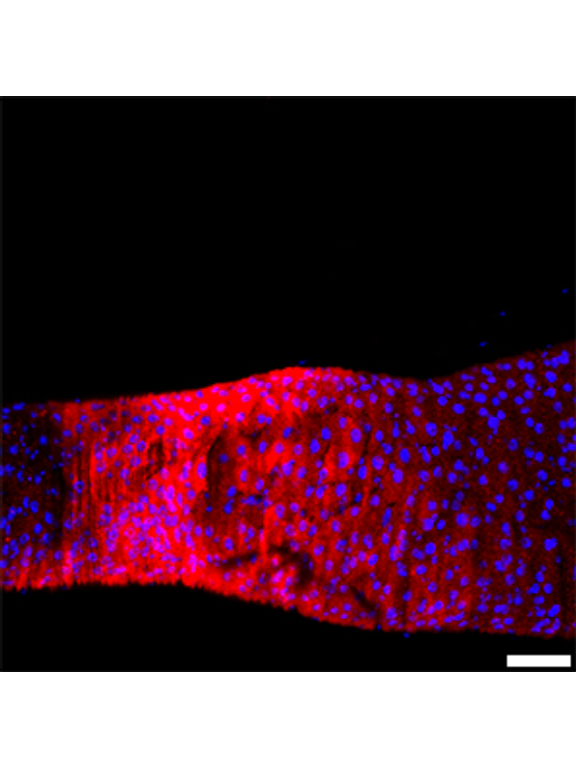

Supplement: Supplementary file 2. [file elife-64125-supp2.zip › Bonfini_script_GutPlasticity_diet_files/figure-html/Figure 5G1-1.png]

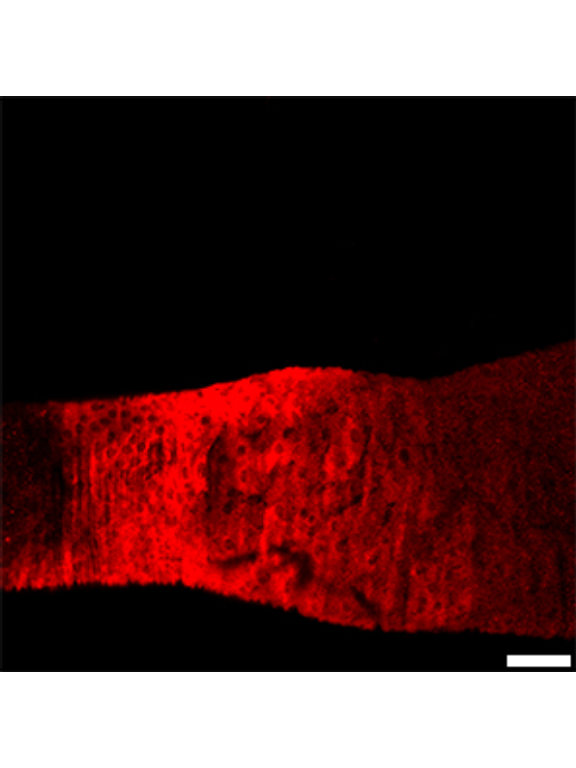

Supplement: Supplementary file 2. [file elife-64125-supp2.zip › Bonfini_script_GutPlasticity_diet_files/figure-html/Figure 5G2-1.png]

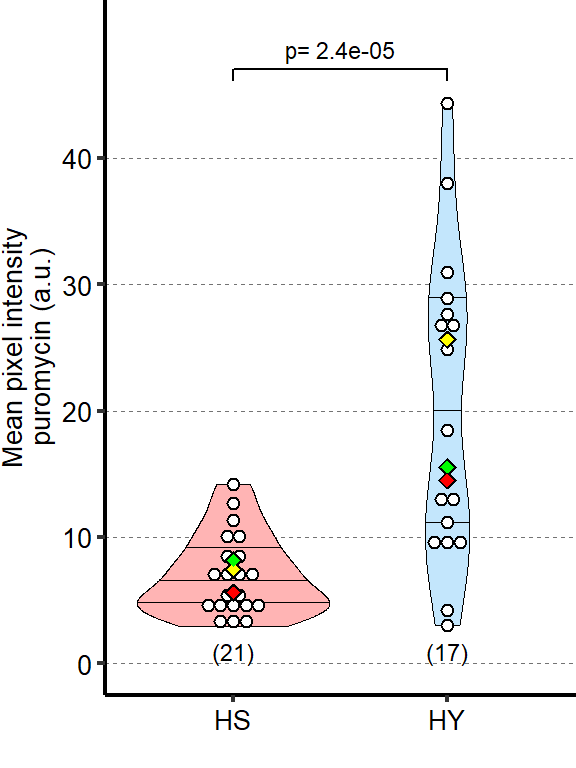

Supplement: Supplementary file 2. [file elife-64125-supp2.zip › Bonfini_script_GutPlasticity_diet_files/figure-html/Figure 5H-1.png]

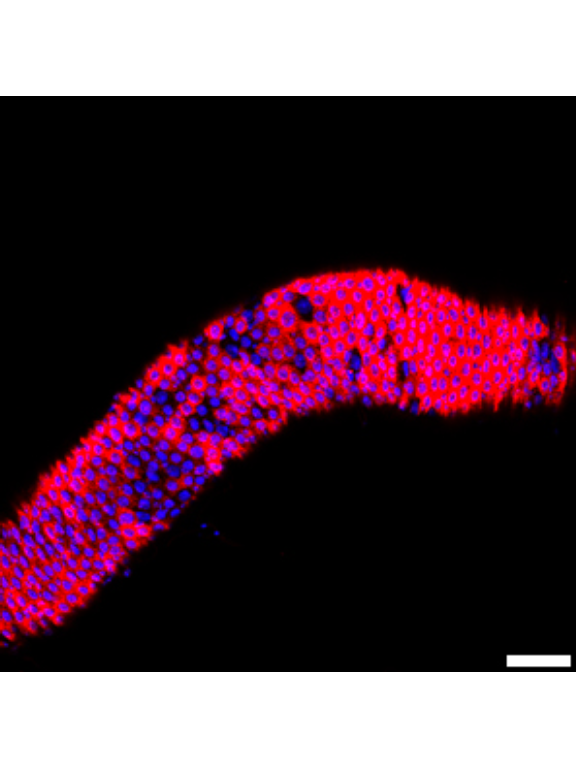

Supplement: Supplementary file 2. [file elife-64125-supp2.zip › Bonfini_script_GutPlasticity_diet_files/figure-html/Figure 5I1-1.png]

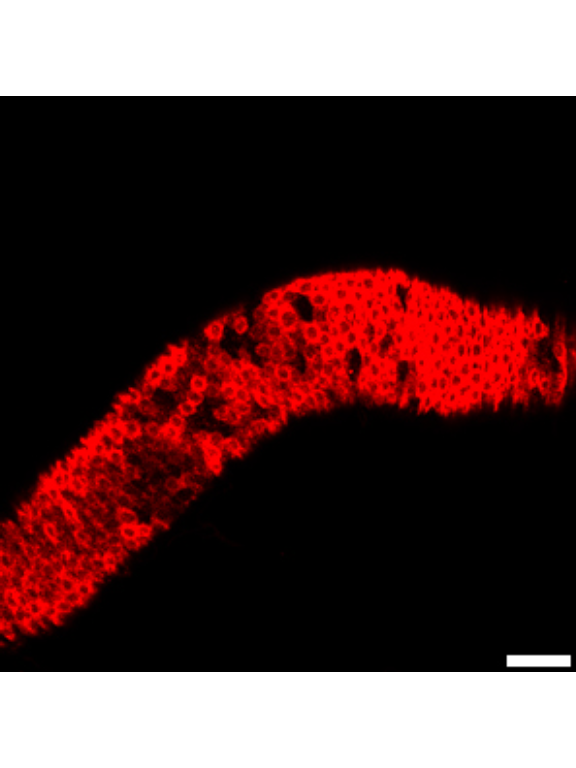

Supplement: Supplementary file 2. [file elife-64125-supp2.zip › Bonfini_script_GutPlasticity_diet_files/figure-html/Figure 5I2-1.png]

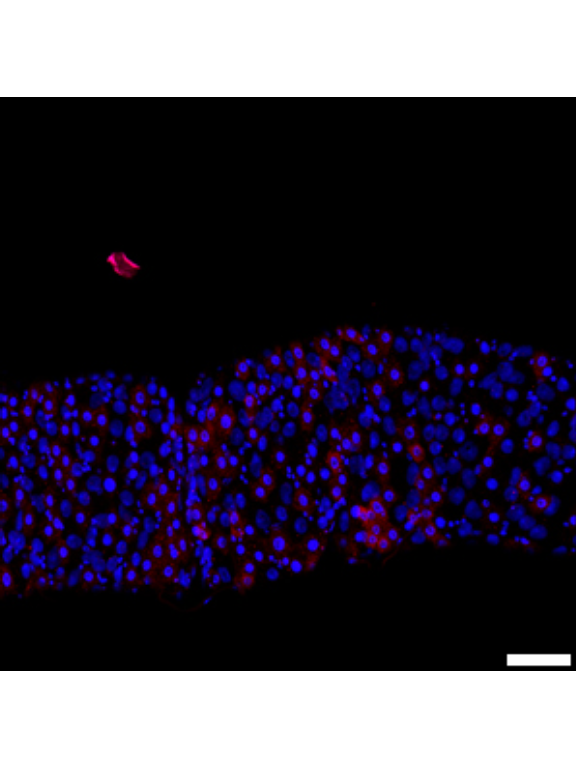

Supplement: Supplementary file 2. [file elife-64125-supp2.zip › Bonfini_script_GutPlasticity_diet_files/figure-html/Figure 5J1-1.png]

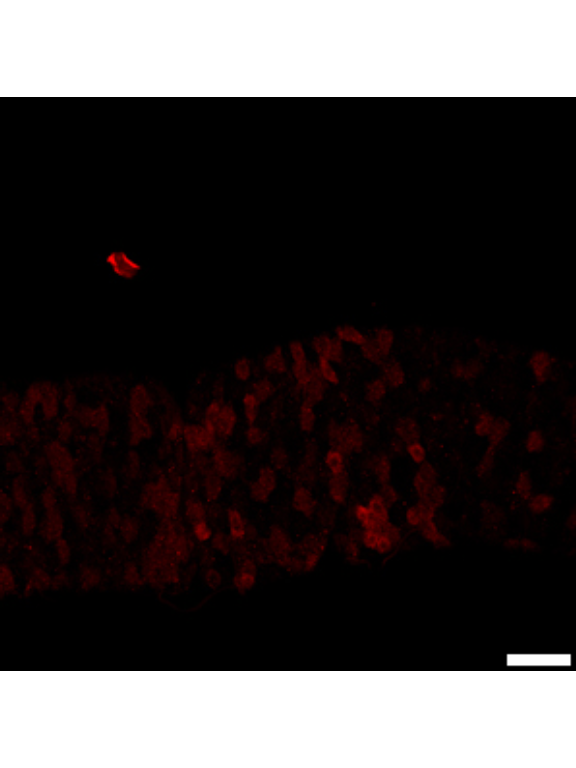

Supplement: Supplementary file 2. [file elife-64125-supp2.zip › Bonfini_script_GutPlasticity_diet_files/figure-html/Figure 5J2-1.png]

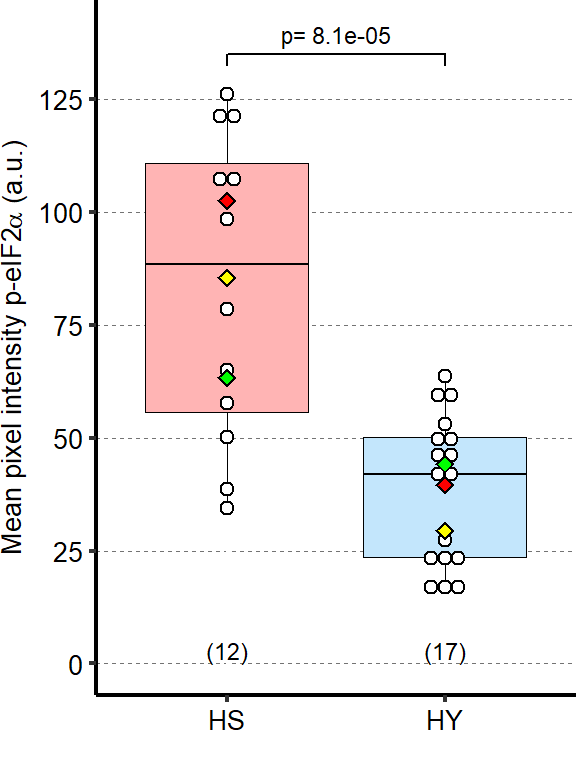

Supplement: Supplementary file 2. [file elife-64125-supp2.zip › Bonfini_script_GutPlasticity_diet_files/figure-html/Figure 5K-1.png]

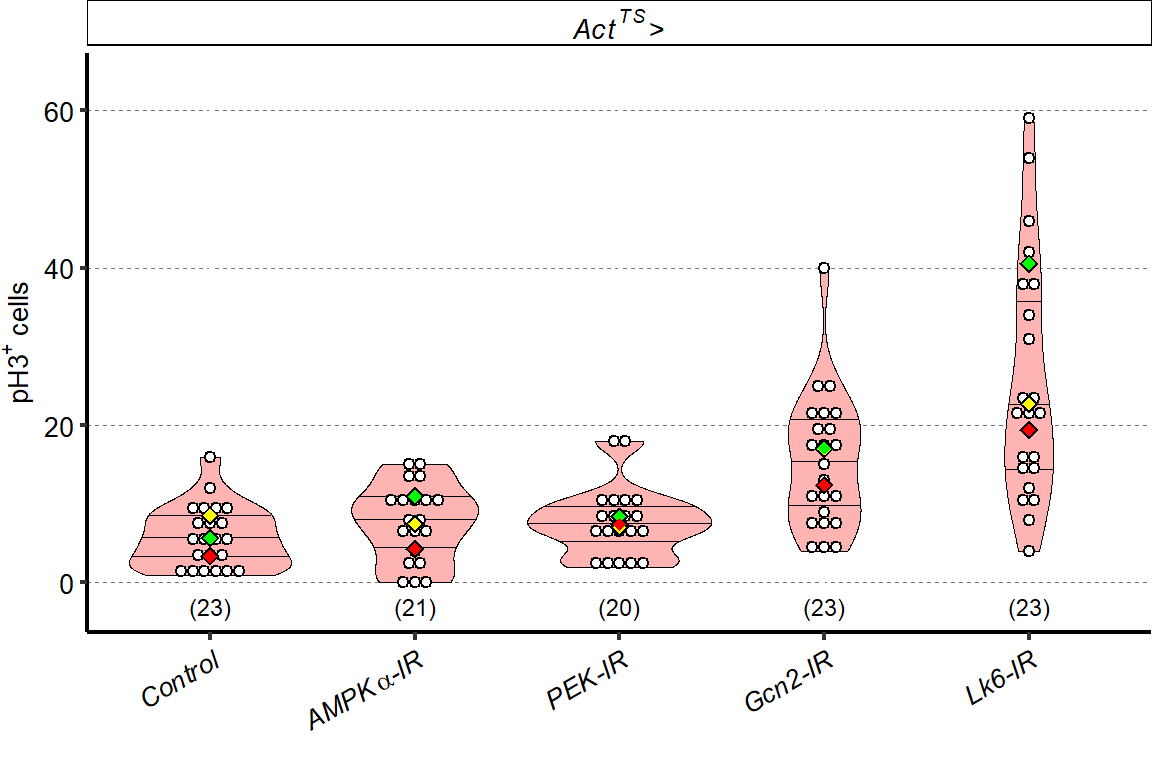

Supplement: Supplementary file 2. [file elife-64125-supp2.zip › Bonfini_script_GutPlasticity_diet_files/figure-html/Figure 5L-1.png]

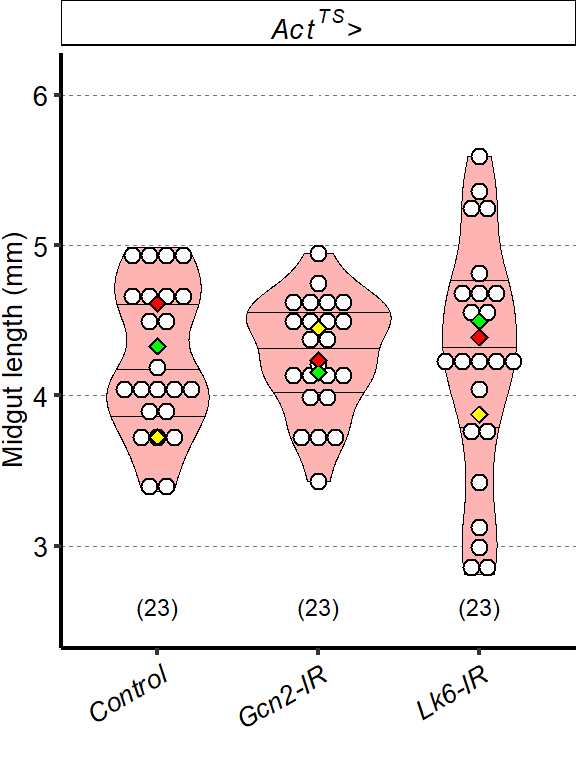

Supplement: Supplementary file 2. [file elife-64125-supp2.zip › Bonfini_script_GutPlasticity_diet_files/figure-html/Figure 5M-1.png]

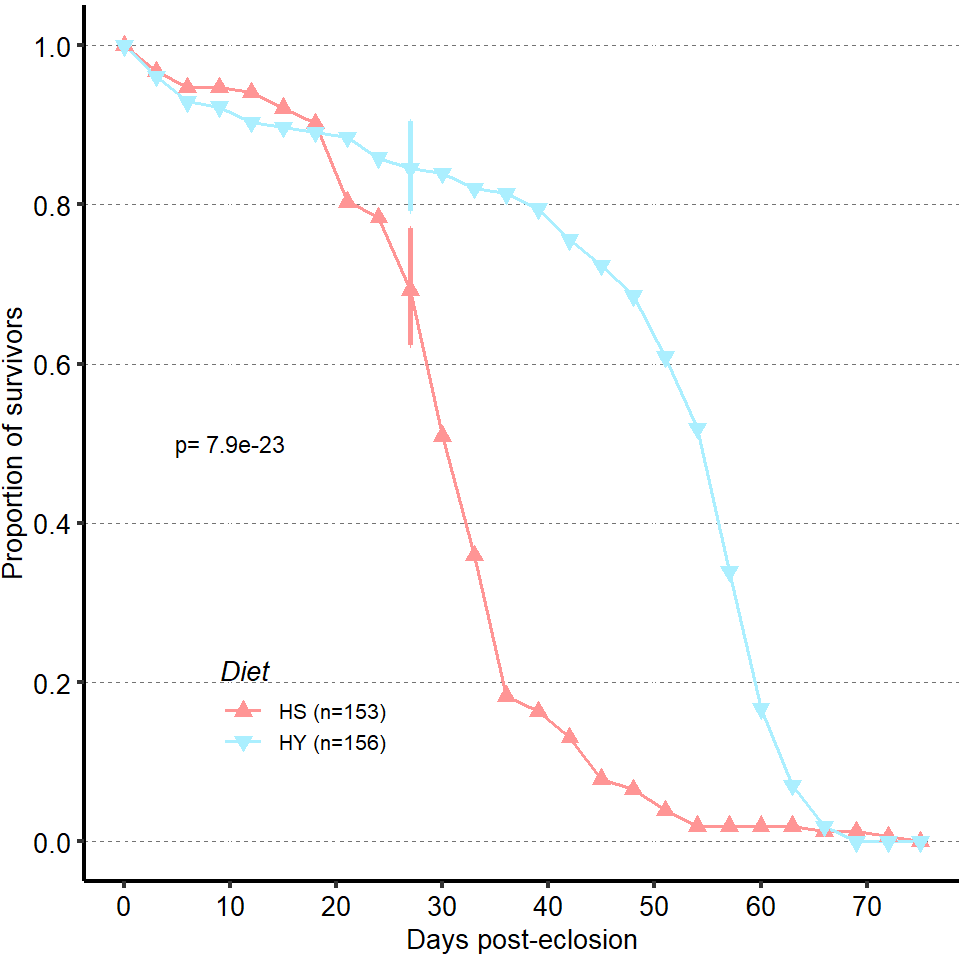

Supplement: Supplementary file 2. [file elife-64125-supp2.zip › Bonfini_script_GutPlasticity_diet_files/figure-html/Figure 5S1A-1.png]

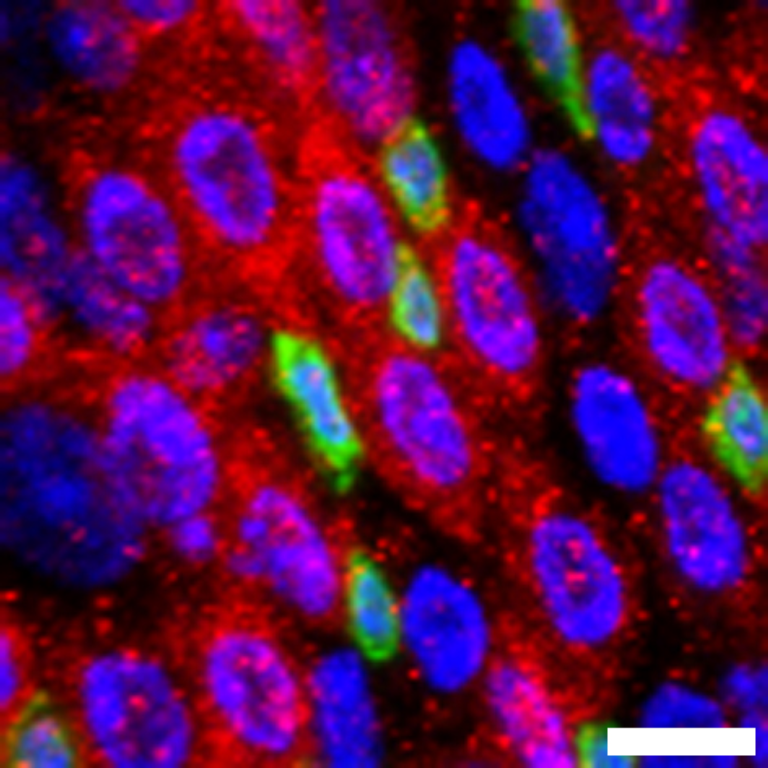

Supplement: Supplementary file 2. [file elife-64125-supp2.zip › Bonfini_script_GutPlasticity_diet_files/figure-html/Figure 5S2A-1.png]

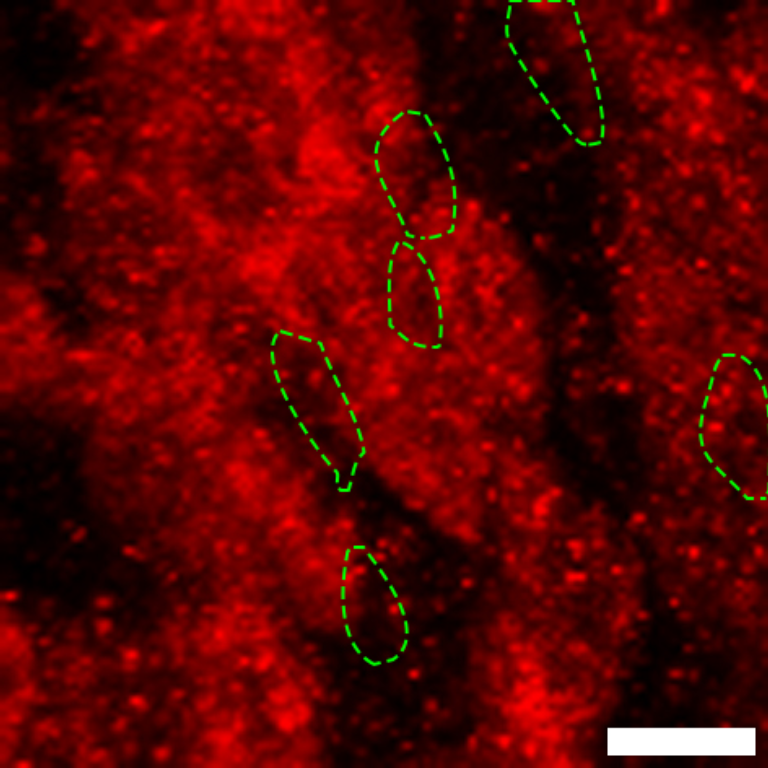

Supplement: Supplementary file 2. [file elife-64125-supp2.zip › Bonfini_script_GutPlasticity_diet_files/figure-html/Figure 5S2A1-1.png]

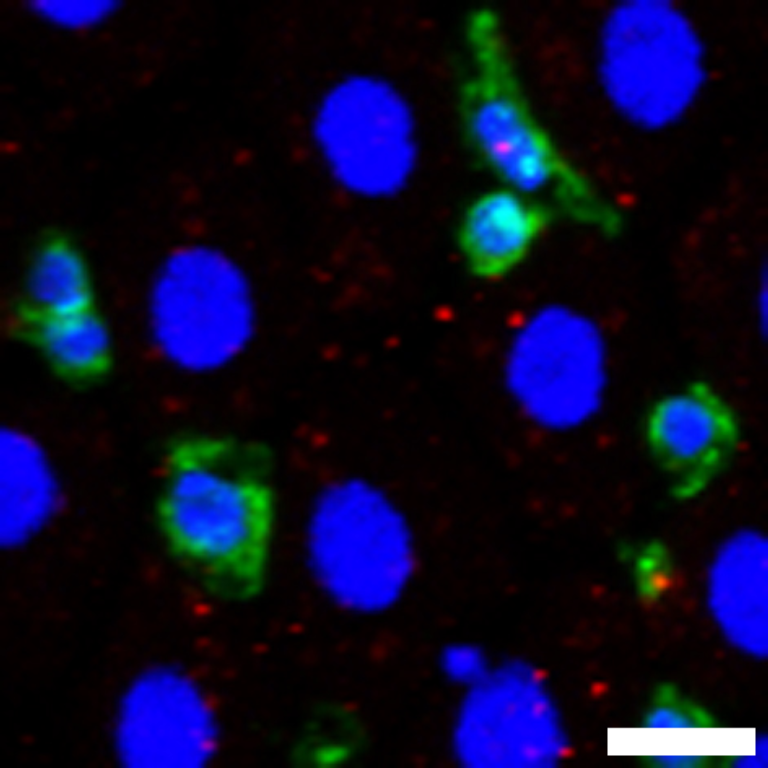

Supplement: Supplementary file 2. [file elife-64125-supp2.zip › Bonfini_script_GutPlasticity_diet_files/figure-html/Figure 5S2B-1.png]

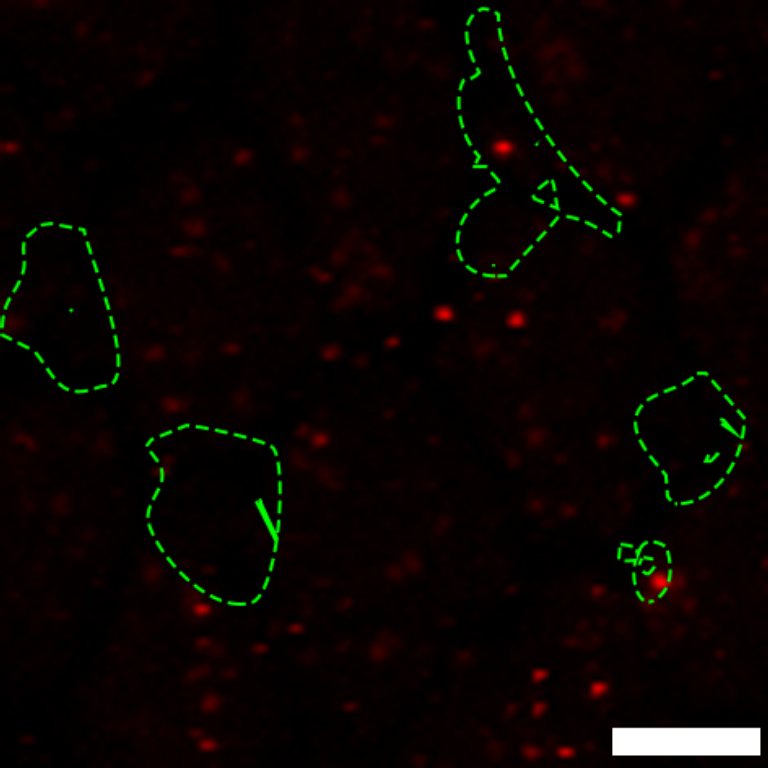

Supplement: Supplementary file 2. [file elife-64125-supp2.zip › Bonfini_script_GutPlasticity_diet_files/figure-html/Figure 5S2B1-1.png]

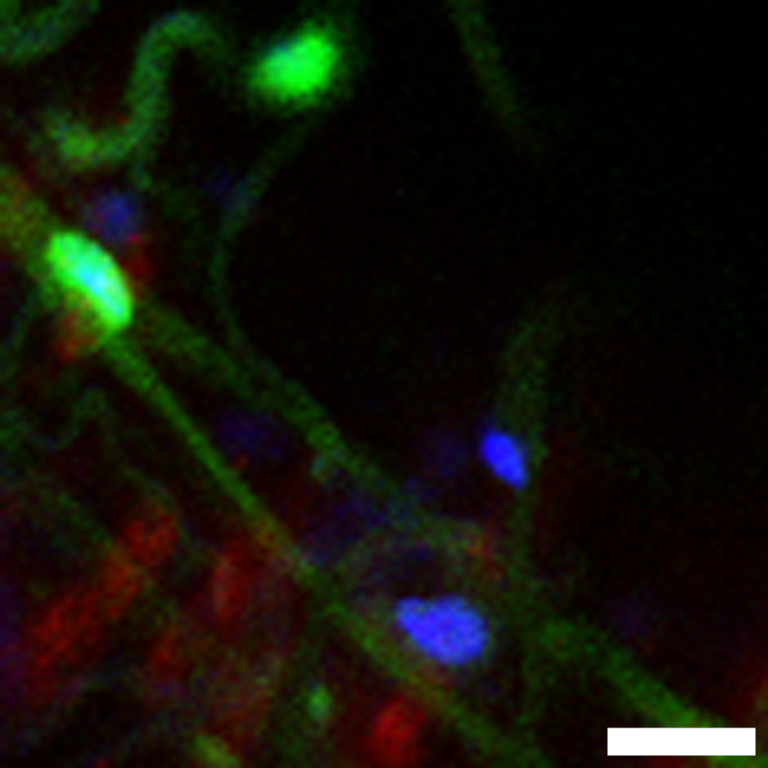

Supplement: Supplementary file 2. [file elife-64125-supp2.zip › Bonfini_script_GutPlasticity_diet_files/figure-html/Figure 5S2C-1.png]

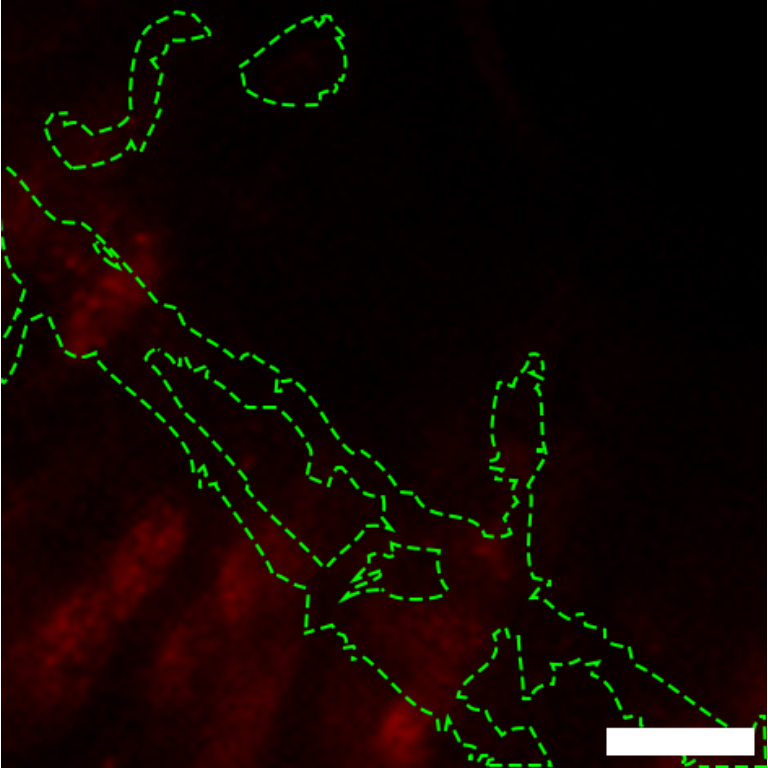

Supplement: Supplementary file 2. [file elife-64125-supp2.zip › Bonfini_script_GutPlasticity_diet_files/figure-html/Figure 5S2C1-1.png]

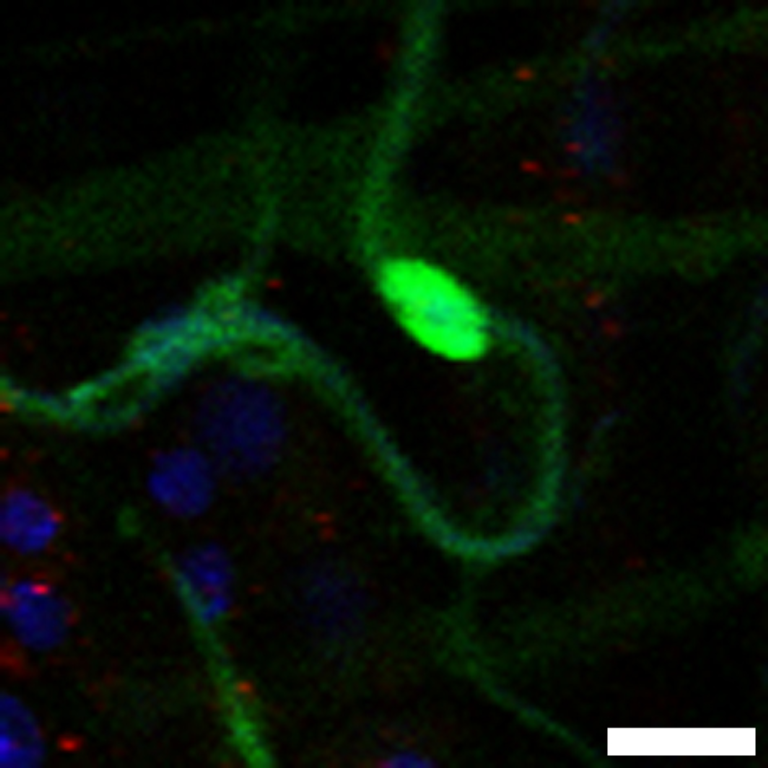

Supplement: Supplementary file 2. [file elife-64125-supp2.zip › Bonfini_script_GutPlasticity_diet_files/figure-html/Figure 5S2D-1.png]

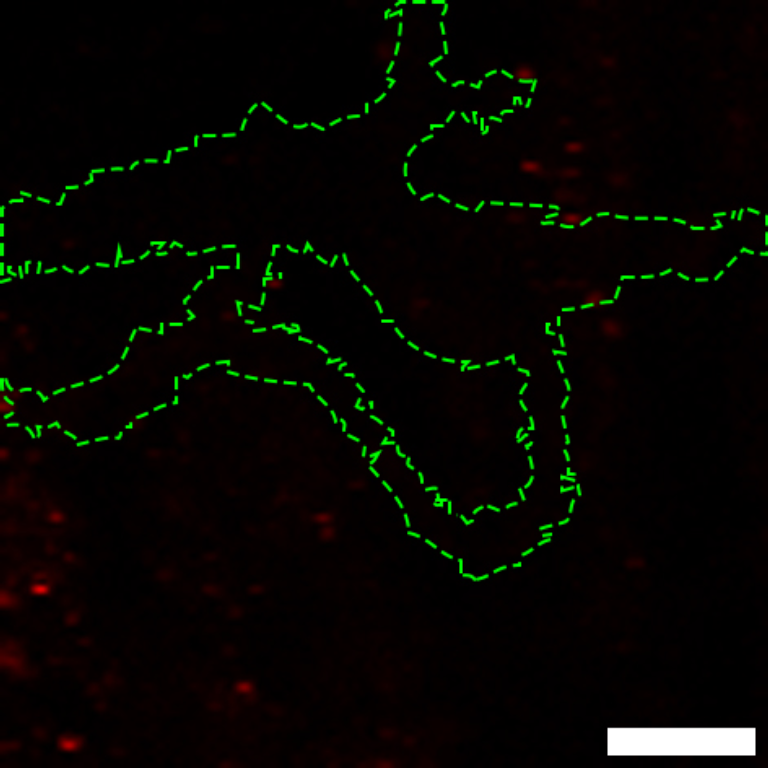

Supplement: Supplementary file 2. [file elife-64125-supp2.zip › Bonfini_script_GutPlasticity_diet_files/figure-html/Figure 5S2D1-1.png]

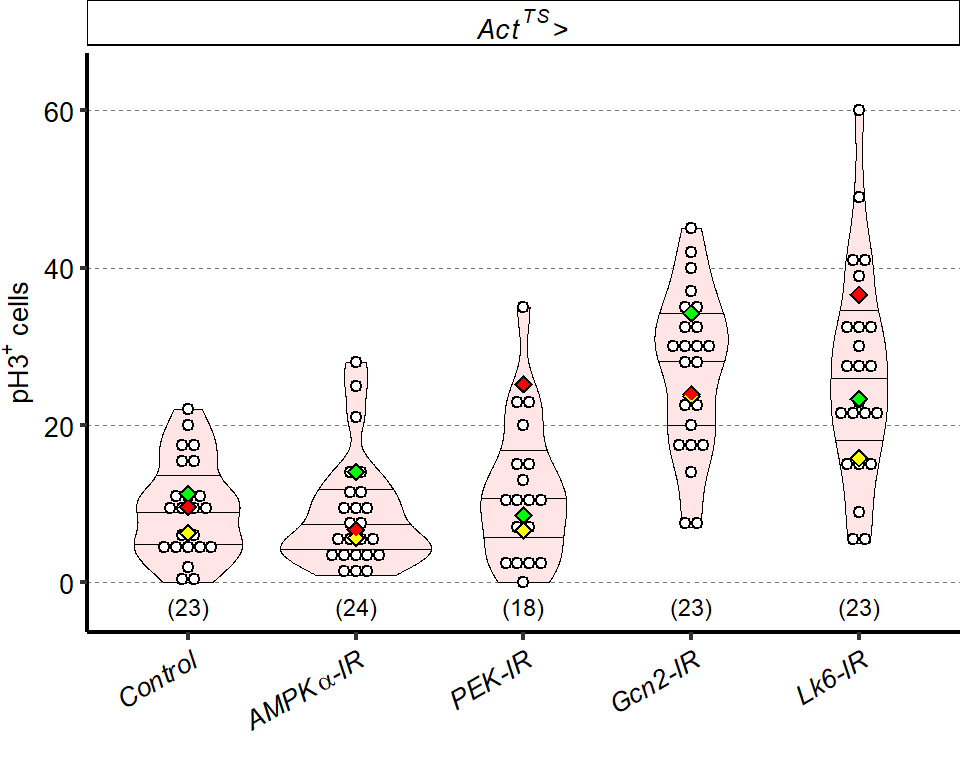

Supplement: Supplementary file 2. [file elife-64125-supp2.zip › Bonfini_script_GutPlasticity_diet_files/figure-html/Figure 5S2E-1.png]

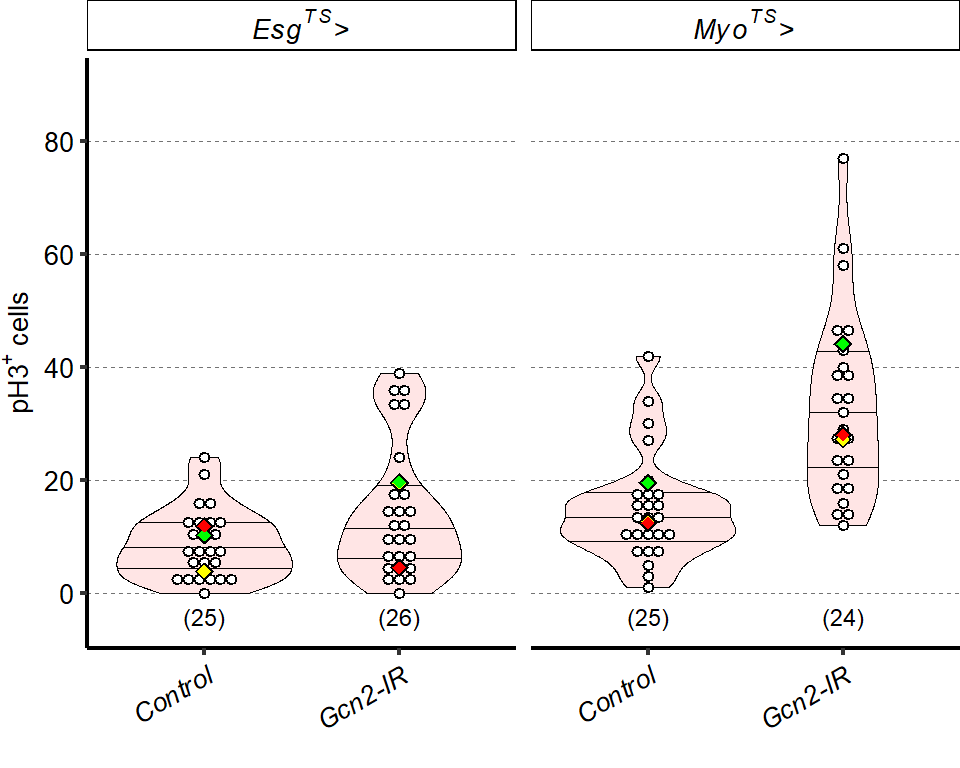

Supplement: Supplementary file 2. [file elife-64125-supp2.zip › Bonfini_script_GutPlasticity_diet_files/figure-html/Figure 5S2G-1.png]

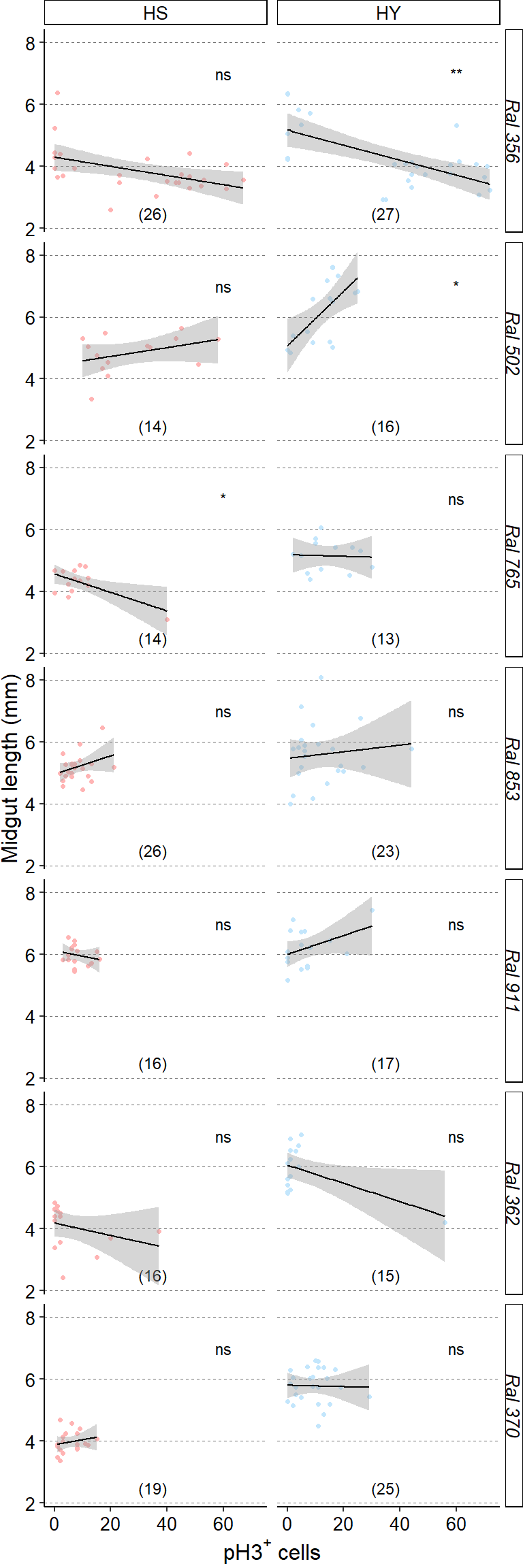

Supplement: Supplementary file 2. [file elife-64125-supp2.zip › Bonfini_script_GutPlasticity_diet_files/figure-html/Figure 6A-1.png]

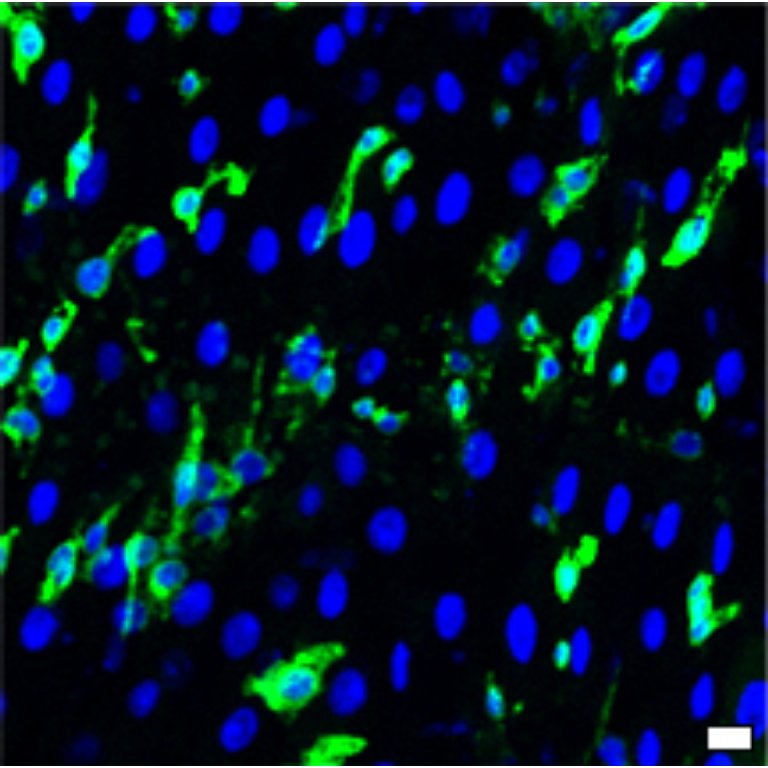

Supplement: Supplementary file 2. [file elife-64125-supp2.zip › Bonfini_script_GutPlasticity_diet_files/figure-html/Figure 6B-1.png]

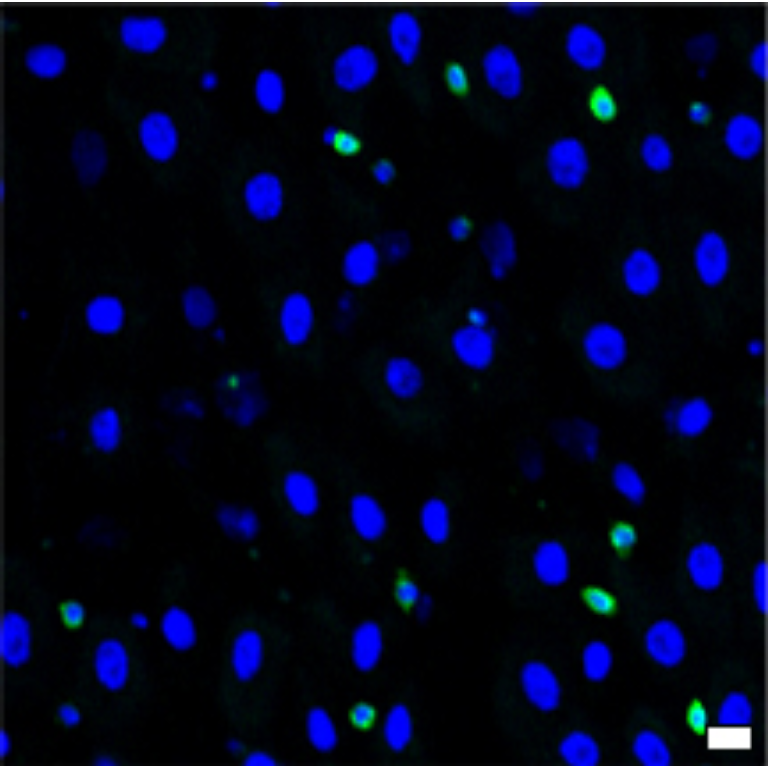

Supplement: Supplementary file 2. [file elife-64125-supp2.zip › Bonfini_script_GutPlasticity_diet_files/figure-html/Figure 6C-1.png]

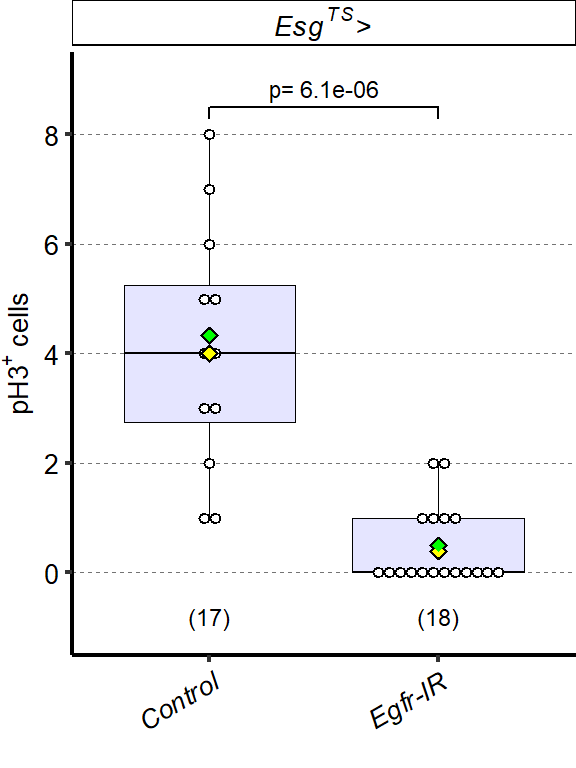

Supplement: Supplementary file 2. [file elife-64125-supp2.zip › Bonfini_script_GutPlasticity_diet_files/figure-html/Figure 6D-1.png]

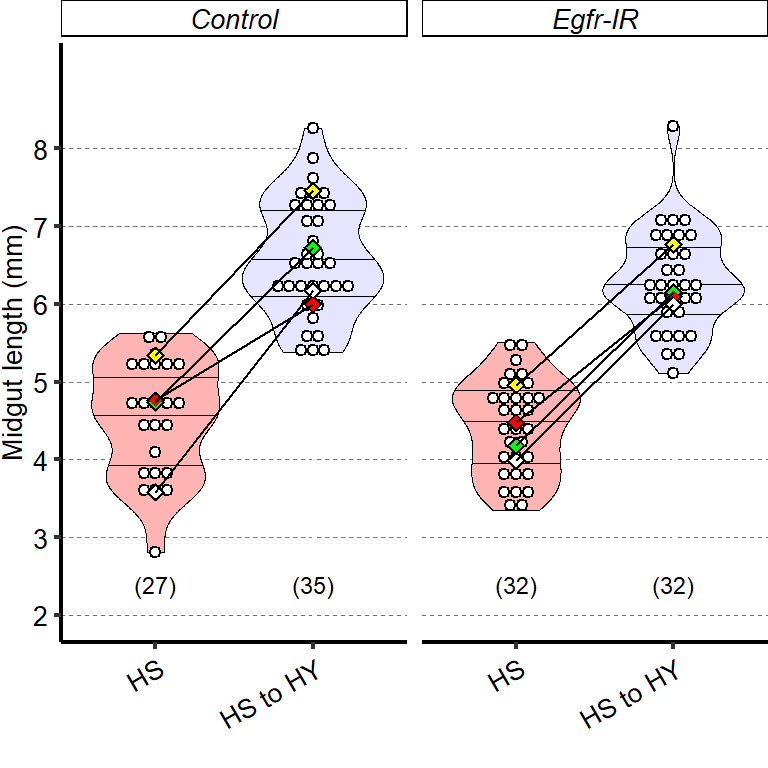

Supplement: Supplementary file 2. [file elife-64125-supp2.zip › Bonfini_script_GutPlasticity_diet_files/figure-html/Figure 6E-1.png]

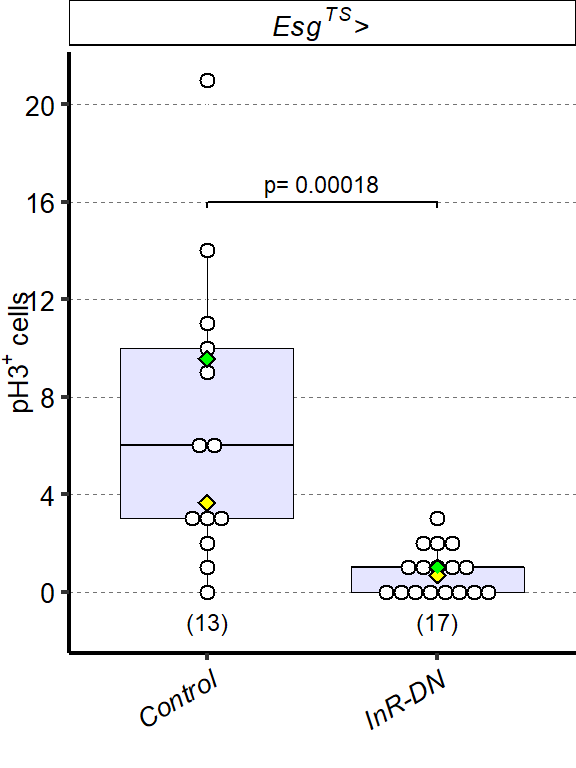

Supplement: Supplementary file 2. [file elife-64125-supp2.zip › Bonfini_script_GutPlasticity_diet_files/figure-html/Figure 6F-1.png]
