# Supplementary material for: Multiscale analysis reveals that diet-dependent midgut plasticity emerges from alterations in both stem cell niche coupling and enterocyte size
Source: eLife. 2021 Sep 23;10:e64125. doi: 10.7554/eLife.64125 (PMC8528489; doi:10.7554/eLife.64125)
Supplement: Transparent reporting form [file elife-64125-transrepform1.pdf]

eLIFE

1st Floor
24 Hills Road
Cambridge CB2 1JP, UK

P 01223 855340
W elifesciences.org
T @elife

eLife's transparent reporting form

We encourage authors to provide detailed information *within their submission* to facilitate the interpretation and replication of experiments. Authors can upload supporting documentation to indicate the use of appropriate reporting guidelines for health-related research (see EQUATOR Network), life science research (see the BioSharing Information Resource), or the ARRIVE guidelines for reporting work involving animal research. Where applicable, authors should refer to any relevant reporting standards documents in this form.

If you have any questions, please consult our Journal Policies and/or contact us: editorial@elifesciences.org.

Sample-size estimation

- You should state whether an appropriate sample size was computed when the study was being designed
- You should state the statistical method of sample size computation and any required assumptions
- If no explicit power analysis was used, you should describe how you decided what sample (replicate) size (number) to use

Please outline where this information can be found within the submission (e.g., sections or figure legends), or explain why this information doesn't apply to your submission:

To ensure the validity of our conclusions we adhered (and often surpassed) the standard of the field. As drosophila allows us to study multiple biological replicates, we performed at least 3 independent biological replicates for each experiment (up to 6 for some experiment, only 2 in few instances), with 4-10 guts for repeats for imaging experiments and 50 guts per repeat for sequencing experiments.

Replicates

- You should report how often each experiment was performed
- You should include a definition of biological versus technical replication
- The data obtained should be provided and sufficient information should be provided to indicate the number of independent biological and/or technical replicates
- If you encountered any outliers, you should describe how these were handled
- Criteria for exclusion/inclusion of data should be clearly stated
- High-throughput sequence data should be uploaded before submission, with a private link for reviewers provided (these are available from both GEO and ArrayExpress)

Please outline where this information can be found within the submission (e.g., sections or figure legends), or explain why this information doesn't apply to your submission:

- We used violin plots combined with dot plots do give a full representation of each measurement (also reported in parenthesis under each chart). We include a mean of each independent repeat directly on the chart (number of lozangez on the chart = number of biological replicates).
- We did not employ technical repeats but only biological repeats (experiments done at separate times (days/weeks) and fly generated by different parents).
- We omitted few single outliers situated completely outside the distribution.
- We submitted our sequencing data to NCBI Sequence Read Archive. Accession currently pending. We can share the data if requested by the reviewers.

Statistical reporting

- Statistical analysis methods should be described and justified
- Raw data should be presented in figures whenever informative to do so (typically when N per group is less than 10)
- For each experiment, you should identify the statistical tests used, exact values of N, definitions of center, methods of multiple test correction, and dispersion and precision measures (e.g., mean, median, SD, SEM, confidence intervals; and, for the major substantive results, a measure of effect size (e.g., Pearson's r, Cohen's d)
- Report exact p-values wherever possible alongside the summary statistics and 95% confidence intervals. These should be reported for all key questions and not only when the p-value is less than 0.05.

Please outline where this information can be found within the submission (e.g., sections or figure legends), or explain why this information doesn't apply to your submission:

- We give an outline of the statistics used in the methods section (under the statistical analysis section). Additionally, we have prepared a "R markdown" containing the exact formula used for each statistical test and all of the data utilized to make the figures. We will publish the markdown together with the publication, and will share it ahead of time with the reviewers.
- Additionally, all of our charts (with few exceptions) show the single measurements as a dot plot directly on the chart.
- We provide the information for point 3 on the charts (exact N number, mean, se or sd, Pearson's etc). For statistical methods, see response to point 1. For the N numbers for the nutritional geometry, the n number are found in figure 2S1A).
- We report the exact p-value directly on the chart.

(For large datasets, or papers with a very large number of statistical tests, you may upload a single table file with tests, Ns, etc., with reference to sections in the manuscript.)

Group allocation

- Indicate how samples were allocated into experimental groups (in the case of clinical studies, please specify allocation to treatment method); if randomization was used, please also state if restricted randomization was applied
- Indicate if masking was used during group allocation, data collection and/or data analysis

Please outline where this information can be found within the submission (e.g., sections or figure legends), or explain why this information doesn't apply to your submission:

- Group of flies for the same experiment were collected together and divided randomly (but keeping same numbers for each vial) into different experimental vials. In case where flies were split after a common treatment, flies were split randomly in the new treatment (dietary shifts experiments) coming from the same initial group.
- Upon dissection, different genotypes were marked with numbers, which were re-associated to the sample's name in the excel sheet after the measurements.

Additional data files ("source data")

- We encourage you to upload relevant additional data files, such as numerical data that are represented as a graph in a figure, or as a summary table
- Where provided, these should be in the most useful format, and they can be uploaded as "Source data" files linked to a main figure or table
- Include model definition files including the full list of parameters used
- Include code used for data analysis (e.g., R, MatLab)
- Avoid stating that data files are "available upon request"

Please indicate the figures or tables for which source data files have been provided:

-As previously mentioned, most of our data are already represented as dot plot in the image. Additionally, we are happy to provide the "R-markdown" containing all the raw data and statistical model used, and code for the charts. We will upload the R markdown upon acceptance of the paper.
